# Engineering N-site isomerism in donor-acceptor covalent organic frameworks for efficient Fenton-like water purification

**DOI:** 10.1038/s41467-026-76345-2

**Published:** 2026-08-07

**Authors:** Chengming Xiao, Mengjia He, Wenbo Yang, Yuxin Tian, Junwen Qi, Zhigao Zhu, Yujun Zhou, Yue Yang, Xiaoguang Duan, Shaobin Wang, Jiansheng Li

**Affiliations:** 1 Jiangsu Key Laboratory of Chemical Pollution Control and Resources Reuse, School of Environmental and Biological Engineering, Nanjing University of Science & Technology, Nanjing, People’s Republic of China; 2 School of Chemical Engineering, Adelaide University, Adelaide, SA Australia

**Keywords:** Pollution remediation, Heterogeneous catalysis, Porous materials

## Abstract

Covalent organic frameworks (COFs) have emerged as promising candidates for singlet oxygen (^1^O_2_) generation via peroxymonosulfate (PMS) activation, yet their structure-property-activity relationships remain poorly understood. Herein, three constitutionally isomeric donor-acceptor COFs (TF-22Bpy, TA-22Bpy and TA-33Bpy) were constructed via N-site isomeric engineering, which involved precisely modulation of the imine and pyridine nitrogen positions within the skeleton. This systematically structural engineering was undertaken to unravel the fundamental effects of regioisomerism on both the electronic structure and subsequent Fenton-like catalytic activity. Among the isomers, TA-33Bpy showed the best catalytic activity for PMS activation, exhibiting an observed rate constant (*k*_obs_) of 0.165 min^−1^. This value is substantially higher than those of TF-22Bpy (0.013 min^−1^) and TA-22Bpy (0.052 min^−1^) by factors of 12.7 and 3.2, respectively. Mechanistic investigations indicate that direct PMS-COF interaction induces charge polarization within the donor-acceptor framework, generating localized electron-deficient and electron-enriched domains that promote the coupled redox steps required for selective ¹O₂ generation. This work identifies PMS-triggered charge polarization as a key determinant of PMS activation and offers a design principle for high-performance COF catalysts for water purification.

## Abstract (web summary)

Covalent organic frameworks (COFs) emerged as promising candidates for singlet oxygen generation, yet their structure-property-activity relationships remain poorly understood. Here the authors construct three constitutionally isomeric donor-acceptor COFs which involves precise modulation of the imine and pyridine nitrogen positions within the skeleton.

## Introduction

Global water crisis is increasingly exacerbated by water pollution of persistent microcontaminants, such as pharmaceuticals residues and endocrine-disrupting chemicals, which pose significant health risks due to their chronic toxicity, environmental persistence, and recalcitrance to biodegradation^1–3^. Heterogeneous peroxymonosulfate (PMS) activation has emerged as a promising advanced oxidation platform for water decontamination, demonstrating great catalytic efficiency, tunable generation of reactive oxygen species (ROSs), and cost-effectiveness^4–6^. Among various ROSs, singlet oxygen (^1^O_2_) offers unique advantages for selective decontamination due to its moderate oxidation potential, extended half-life, and pronounced electrophilic character, which enables preferential attack on electron-rich organic pollutants^7–10^. However, due to the inherent instability of the asymmetric O–O bond, PMS is easily activated to generate multiple ROSs, including hydroxyl (•OH), sulfate (SO_4_^•‒^), and superoxide radicals (O_2_^•‒^) as well as ^1^O_2_^5,11,12^. Multiple ROSs in complex aqueous matrices, along with mass transfer limitations in a heterogeneous system, often compromises the practical applicability^13,14^. While metal-based catalysts risk ion leaching and secondary pollution, metal-free alternatives typically suffer from insufficient activity, poorly defined active sites, and limited structural tunability^15–18^. Therefore, rational design of heterogeneous catalysts for the precise control and efficient generation of ^1^O_2_ from PMS remains a significant challenge.

In metal-free systems, ^1^O_2_ generation from PMS generally proceeds via a two-step electron-transfer pathway: (i) single-electron oxidation of PMS to form SO_5_^•‒^ intermediates (Eq. 1), followed by (ii) two-electron reduction of two SO_5_^•‒^ to yield ^1^O_2_ and SO_4_^2‒^ (Eq. 2)^19,20^. The complete pathway requires both oxidation and reduction steps, which are difficult to achieve simultaneously at a single active site. This mechanistic insight underscores the need for bifunctional catalysts with spatially separated sites to respectively drive PMS oxidation and SO_5_^•‒^ reduction.

1 $${{{\rm{Active}}}}\,{{{\rm{site}}}}+{{{{\rm{HSO}}}}}_{5}^{-}{\to }^{\ast }{{{{\rm{SO}}}}}_{5}^{\bullet -}+{{{{\rm{H}}}}}^{+}+{{{{\rm{e}}}}}^{-}({{{\rm{Active}}}}\,{{{\rm{site}}}})$$

2 $$2{{{{\rm{e}}}}}^{-}({{{\rm{Active}}}}\,{{{\rm{site}}}}){+}^{\ast }{{{{\rm{SO}}}}}_{5}^{\bullet -}+{{{{\rm{SO}}}}}_{5}^{\bullet -}\to 2{{{{\rm{SO}}}}}_{4}^{2-}{+}^{1}{{{{\rm{O}}}}}_{2}$$

Covalent organic frameworks (COFs) are crystalline porous materials constructed from organic building blocks linked via strong covalent bonds^21–23^. Their structural regularity, high porosity, and chemical stability make them versatile platforms^24,25^ for diverse applications ranging from energy storage^26–28^ and molecular separation^29–31^ to catalysis^32–36^. In multipath catalytic reactions, the structural homogeneity of active sites in COFs enables precise control over reaction selectivity^37^. Furthermore, through rational ligand design and functional group customization, the electronic configurations of these catalytic centers can be systematically modulated to optimize their intrinsic activity. In particular, two-dimensional (2D) COFs with periodic columnar π-arrays exhibit efficient charge-carrier mobility and long-live excited states. Inspired by donor-acceptor (D-A) heterojunction in photovoltaics, integrating spatially ordered D and A units into 2D COFs has proven effective in promoting electron-hole separation and directional charge transport^38,39^, leading to enhanced performance across various reactions^40–43^. For example, Liu et al. designed a D-A COF that separately catalyzes water oxidation at donor sites and oxygen reduction at acceptor sites for efficient H_2_O_2_ photosynthesis from water and air^43^. The donor-acceptor columnar π-arrays function dually as both charge transport channels and catalytic centers, facilitating four-electron water oxidation at the donor sites and two-electron oxygen reduction at the acceptor moieties. Notably, ^1^O_2_ generation from PMS shares mechanistic similarities with such coupled redox processes, suggesting that rationally designed D-A COFs could likewise enable simultaneous PMS oxidation and SO_5_^•‒^ reduction for selective ^1^O_2_ generation. Despite several reports on pristine COFs for PMS activation^44,45^, for example, Weng et al. demonstrated the introduction of pyridine-N atoms could provide additional adsorption sites for the terminal H atom of PMS, thereby promoting S-O bond cleavage to generate ^1^O_2_, the deliberate use of D-A architectures to control ROS speciation in advanced oxidation processes (AOPs) remains unexplored.

Herein, we report the design of three constitutionally isomeric D-A COFs (TF-22Bpy, TA-22Bpy and TA-33Bpy) sharing the same topology but differ in the positions of imine and pyridine nitrogen atoms within the framework. Through N-site isomeric engineering, we systematically investigate the effect of regioisomerism on the electronic and Fenton-like performance. Among them, TA-33Bpy exhibits the best activity in activating PMS for bisphenol A (BPA) degradation, with an observed rate constant (*k*_obs_) of 0.165 min^−1^, representing 12.7-fold and 3.2-fold enhancements over TF-22Bpy (0.013 min^−1^) and TA-22Bpy (0.052 min^−1^), respectively. More importantly, we elucidate for the first time a PMS-triggered charge polarization mechanism, in which direct PMS–COF interaction induces electronic redistribution across the donor (D^+^) and acceptor (A^‒^) units, thereby cooperatively promoting PMS oxidation and SO_5_^•‒^ reduction toward predominant ^1^O_2_ generation. This work not only demonstrates the critical role of PMS-triggered charge polarization on PMS activation for ^1^O_2_ generation but also establishes a new design paradigm for developing high-performance COFs, advancing the development of catalysts toward efficient Fenton-like water purification.

## Results

### Molecular design and theoretical calculation

**Fig. 1:**
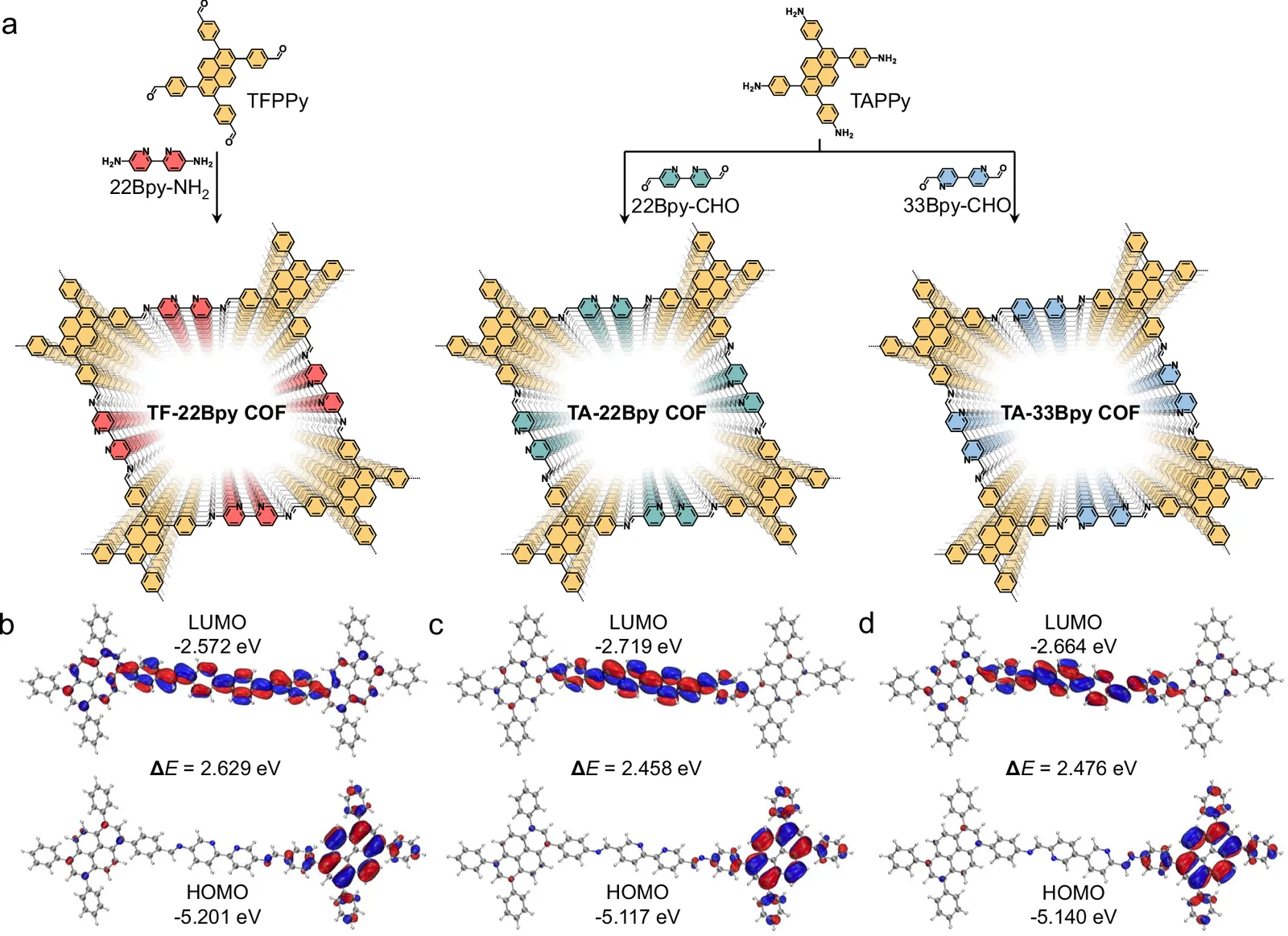
Molecular design of isomeric COFs. **a** Synthetic routes and corresponding chemical structures of TF-22Bpy, TA-22Bpy and TA-33Bpy COFs. Kohn–Sham LUMOs and HOMOs of **b** TF-22Bpy, **c** TA-22Bpy, and **d** TA-33Bpy COFs.

For construction of D-A COFs with precisely modulated electronic structures, the design of isomeric building blocks capable of fine-tuning electron distribution is essential. In this work, tetraphenylpyrene, which possesses a highly symmetric structure and extended π-conjugated system, was selected as the electron-donating knot, while electron-deficient pyridine served as the acceptor linker^46,47^. By strategically varying the relative position of imine and pyridine nitrogen atoms within the skeleton, we constructed three constitutionally isomeric COFs: TF-22Bpy, TA-22Bpy, and TA-33Bpy COFs (Fig. 1a). Specifically, the imine-N is oriented closer to the bipyridine linker in TF-22Bpy, whereas it is closer to the pyrene knot in TA-22Bpy and TA-33Bpy. Furthermore, while the pyridine-N is adjacent in TA-22Bpy and TF-22Bpy, it adopts a diagonally opposite configuration in TA-33Bpy. The adjacent pyridine-N motif in TF-22Bpy and TA-22Bpy is derived from the 2,2’-bipyridyl unit, whose protonated form preferentially adopts a cis conformation under acidic solvothermal conditions because of the intramolecular [N − H^+^···N] hydrogen bond between the two pyridine N atoms. Similarly, TA-33Bpy shows a pyridine-like N environment stabilized by an intramolecular [N − H^+^···N] hydrogen bond between the protonated pyridine-N and the neighboring imine-N^48^. All three COFs share an identical tetrahedral topology, with a theoretical pore size of ~2.53 nm. To elucidate the influence of N-site isomerism on the electron distribution within the COF skeletons, we performed electrostatic potential (ESP) analysis. As illustrated in Supplementary Fig. 1, the higher electronegativity of nitrogen relative to carbon draws electron density towards the N sites, rendering these regions negatively charged in all three COFs. A distinct charge localization pattern emerges in TA-33Bpy: the spatial proximity between the imine and pyridine nitrogen atoms creates a bipyridine-like structure that concentrates negative charge at this combined site. In contrast, the negative charges in TF-22Bpy and TA-22Bpy remain separately localized at the pyridine and imine nitrogen sites. These results clearly demonstrate that precise positional engineering of N-sites enables targeted control over intramolecular electron distribution, providing a molecular-lever strategy to tailor the electronic structure of D-A COFs.

To further elucidate how N-site position governs the electronic structure of the COFs, we conducted the frontier orbital analysis. As shown in Fig. 1b–d, the highest occupied molecular orbitals (HOMO) of all three isomers are predominantly localized on the electron-rich pyrene knot, while the lowest occupied molecular orbitals (LUMO) reside mainly on the electron-deficient bipyridine linker. This clear spatial separation of frontier orbitals is the characteristic of a donor-acceptor architecture and is intrinsically favorable for intermolecular charge polarization and electron transfer^42,49^. Notably, the HOMO and LUMO energy gaps of TA-22Bpy (ΔE = 2.458 eV) and TA-33Bpy (ΔE = 2.476 eV) are similar and significantly smaller than that of TF-22Bpy (ΔE = 2.629 eV). A narrower bandgap generally facilitates more efficient charge excitation and transfer, which is a key electronic descriptor for enhancing the catalytic activity^38,49^. Together, these results demonstrate that precise control over N-site isomerism provides an effective strategy to modulate the frontier orbital energetics and spatial distribution in COFs, thereby validating the proposed design principle for tailoring their electronic and catalytic properties.

### Material synthesis and characterizations

Three isomeric D-A COFs (TF-22Bpy, TA-22Bpy and TA-33Bpy) were synthesized by polycondensation of pyrene knots and bipyridine linkers under optimized solvothermal conditions. Specifically, TF-22Bpy was obtained by reacting 1,3,6,8-tetrakis(4-formylphenyl)pyrene (TFPPy) with 2,2’-bipyridine-5,5’-diamine (22Bpy-NH_2_) in a 1:1 (v/v) mixture of n-butyl alcohol (BuOH) and 1,2-dichlorobenzene (o-DCB) using 6 M acetic acid (HAc) as a catalyst at 120 °C for 72 h. TA-22Bpy and TA-33Bpy were prepared analogously by reacting 1,3,6,8-tetra(aminophenyl)pyrene (TAPPy) with 2,2’-bipyridyl-5,5’-dialdehyde (22Bpy-CHO) and 6,6’-diformyl-3,3’-bipyridine (33Bpy-CHO), respectively, in a 1:1 (v/v) mixture of 1,4-dioxane (Diox) and mesitylene (TMB) under the same catalytic and thermal conditions.

**Fig. 2:**
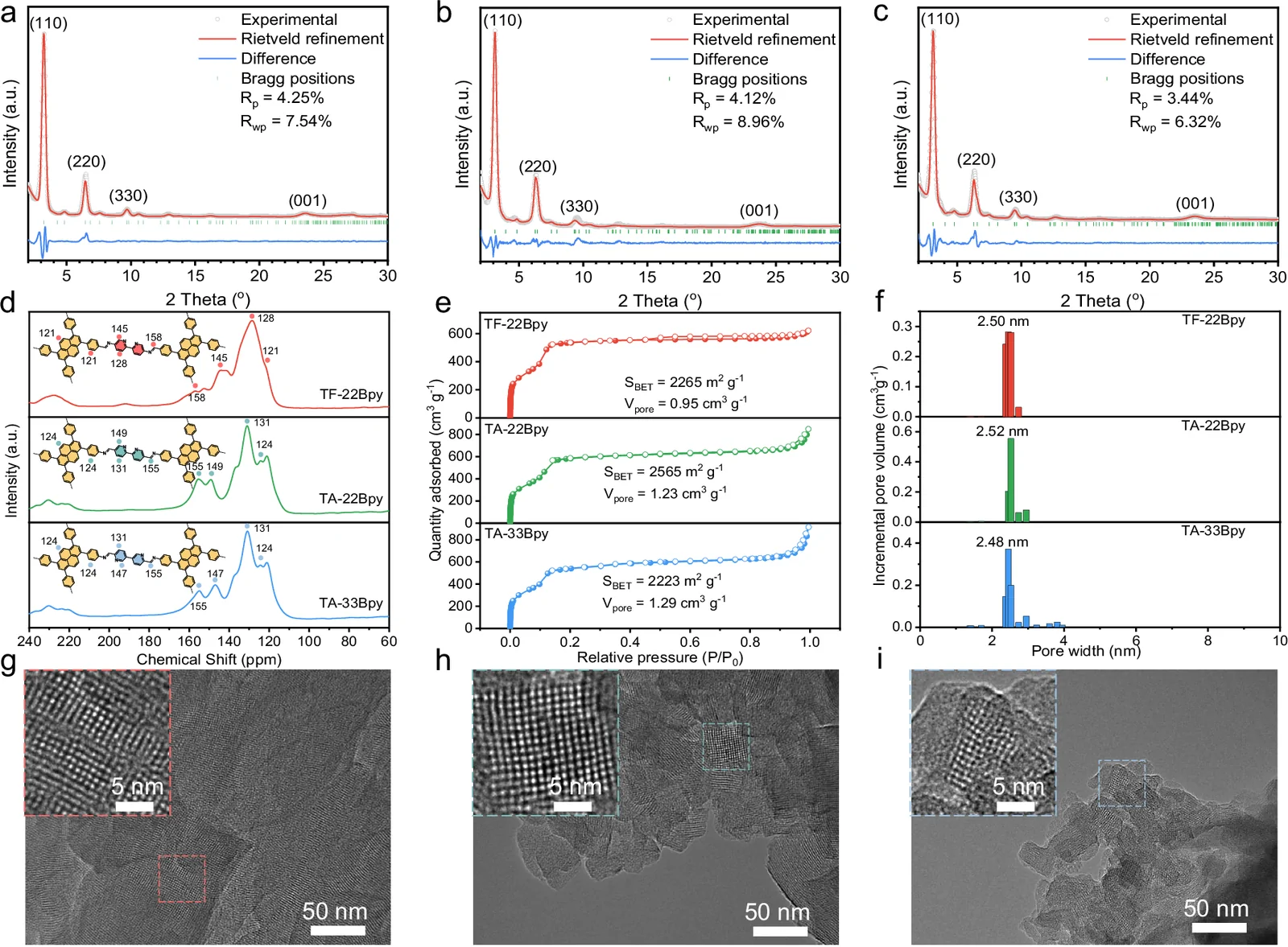
Characterizations of isomeric COFs. Experimentally obtained PXRD patterns (grey circle), Pawley refined patterns (red curves), their differences (blue curves) and Bragg positions (green verticals) of **a** TF-22Bpy, **b** TA-22Bpy, and **c** TA-33Bpy COF. (Insets: Top and side views of COFs with AA-stacking). **d** Solid-state ^13^C CP-MAS NMR spectra, **e** N_2_ adsorption-desorption isotherms, and **f** pore size distributions of isomeric COFs. TEM and HRTEM images of (**g**) TF-22Bpy, **h** TA-22Bpy, and **i** TA-33Bpy COFs.

Powder X-ray diffraction (PXRD) patterns verified the high crystallinity of the three isomeric COFs (Fig. 2a-c). To determine the stacking mode with higher confidence, we compared the experimental PXRD data with simulated patterns based on inclined AA-, AB-, and ABC-stacking models. In all three cases, the inclined AA-stacking model reproduced the experimental diffraction features most satisfactorily, whereas the AB- and ABC-stacking models showed obvious mismatches in peak positions and relative intensities (Supplementary Figs. 2–4). These results indicate that TF-22Bpy, TA-22Bpy, and TA-33Bpy adopt an inclined AA-stacking mode despite their distinct regioisomeric configurations. Based on these validated stacking models, Rietveld refinements were performed using monoclinic structural models to yield good agreement between the observed and calculated PXRD profiles. All three COFs were satisfactorily refined in the monoclinic CM space group, with low residual factors and reasonable GOF values, confirming the reliability of the structural models. The refined lattice parameters further reveal subtle isomer-dependent variations in framework geometry, while preserving the overall topology and inclined AA stacking.

The successful formation of imine linkages in the three COFs was confirmed by Fourier Transform Infrared (FT-IR) spectroscopy. As shown in Supplementary Fig. 5, the emergence of characteristic C = N bond stretching vibration at 1624 (TF-22Bpy), 1627 (TA-22Bpy), and 1608 cm^-1^ (TA-33Bpy) was accompanied by the disappearance of the primary amine (-NH_2_) and aldehyde (-CHO) signals of the corresponding monomers, indicating complete Schiff-base condensation. ^13^C solid-state nuclear magnetic resonance (NMR) spectroscopy provides further structural verification (Fig. 2d). The spectra of all three COFs exhibited clear signals corresponding to imine carbon (C = N) and pyridine rings carbons, with the characteristic peaks appearing at 158, 145, 128 ppm for TF-22Bpy; 155, 149, 131 ppm for TA-22Bpy; and 155, 147, 131 ppm for TA-33Bpy. Notably, the subtle but systematic variations in chemical shift among the isomers reflect the distinct electronic environments induced by the different positions of imine and pyridine nitrogen atoms, confirming that the chemical structure was precisely tuned through N-site isomerism. X-ray photoelectron spectroscopy (XPS) survey scans revealed comparable signals for C 1 *s*, O 1 *s*, and N 1 *s* among the isomeric COFs, confirming their similar chemical composition (Supplementary Figs. 6–8). High-resolution N 1 *s* spectra could be deconvoluted into two components corresponding to imine-N and pyridine-N. The binding energies for these two species were 398.8 and 399.3 eV in TF-22Bpy, 399.4 and 400.3 eV in TA-22Bpy, and 399.3 and 400.0 eV in TA-33Bpy. The consistent presence of both N species, along with their varying binding energies across the isomers, further corroborates the successful incorporation of bipyridine linkers and imine bonds, as well as the distinct electronic modulation achieved by N-site isomerism.

The porous structure of the as-synthesized COFs was evaluated by N_2_ adsorption-desorption measurements. TF-22Bpy, TA-22Bpy, and TA-33Bpy demonstrated high Brunauer–Emmett–Teller (BET) surface areas (2265, 2565, and 2223 m^2^ g^-1^, respectively) and pore volume (0.95, 1.23, and 1.29 cm^3 ^g^-1^, respectively), indicating their excellent crystalline and porosity (Fig. 2e). Pore size distribution derived from the adsorption branches revealed a dominant pore size of ~2.50 nm for each COF, which aligns well with the theoretical value (2.53 nm) predicted from the structure models (Fig. 2f). Morphology and structural details were further examined by scanning electron microscopy (SEM) and transmission electron microscopy (TEM). SEM images showed that TF-22Bpy adopts an irregular stick-like morphology, TA-22Bpy forms agglomerated particles, and TA-33Bpy exhibits a distinctive sea-urchin-like architecture (Supplementary Figs. 9–11). Despite these morphological differences, high-resolution TEM images of all isomeric COFs display well-defined lattice fringes with interplanar spacing of about 1.83 nm (Fig. 2g-h), which corresponds to the (020) plane in COFs, consistent with the PXRD results. Collectively, these structural and textural characterizations confirm the successful synthesis of three constitutionally isomeric COFs that share the same tetragonal topology but differ in the precise positioning of nitrogen sites within the framework.

### Physicochemical property and Fenton-like performance

**Fig. 3:**
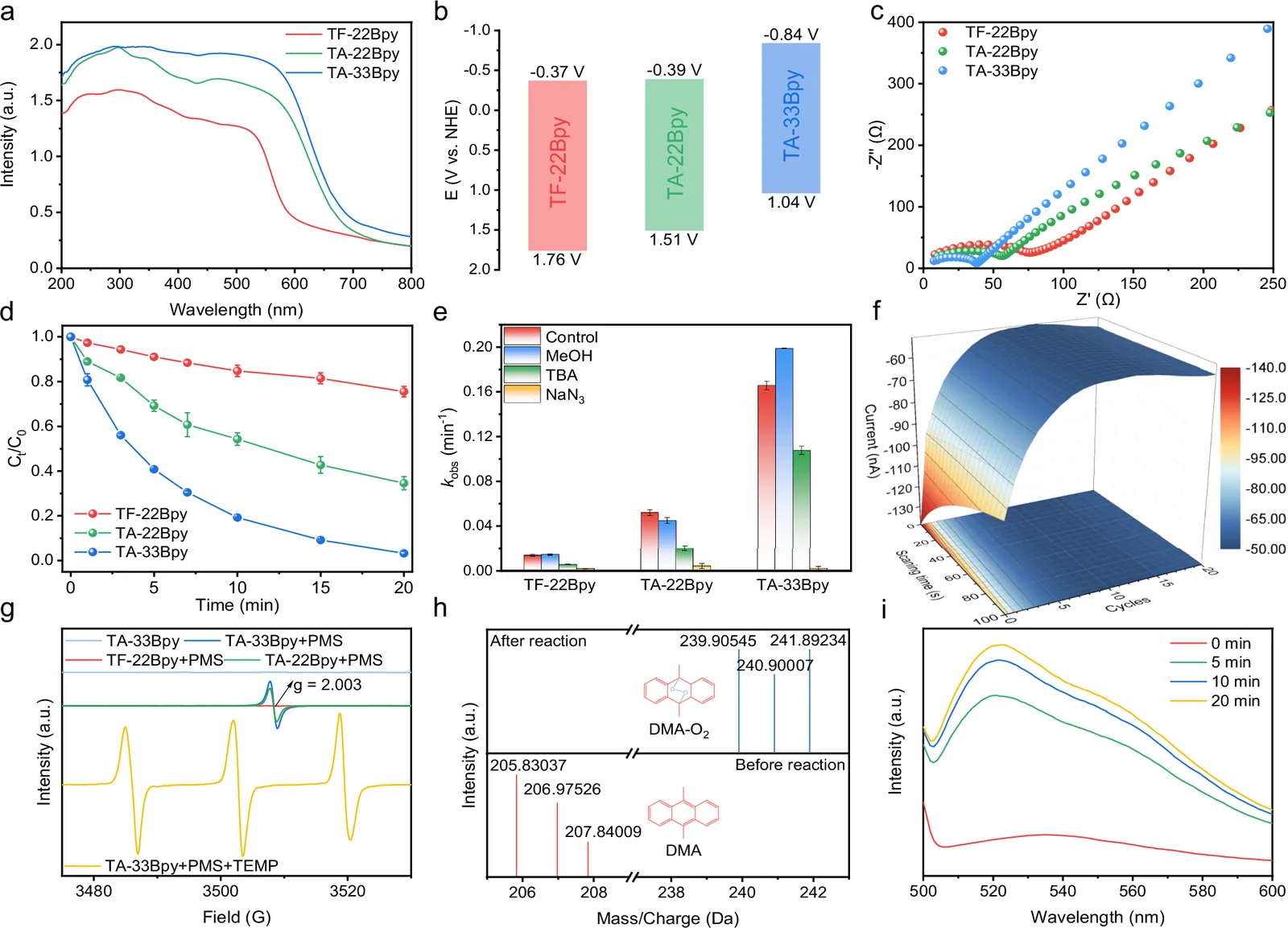
Physicochemical properties and Fenton-like performance. **a** UV–vis–NIR diffuse reflectance spectra, **b** electronic band structures, and **c** electrochemical impedance spectra of the isomeric COFs. **d** BPA removal and **e** quenching experiments of all COFs/PMS system. **f** Scanning electrochemical microscopic analysis of TA-33Bpy/PMS systems. **g** EPR spectra of TA-33Bpy/PMS system using TEMP as trapping agents. **h** HR-MS chromatogram of typical DMA-O_2_ from DMA oxidation in TA-33Bpy/PMS system. **i** Fluorescence spectra of SOSG-EP in TA-33Bpy/PMS system. Data for (**d**, **e**) are presented as mean values ± SD (*n* = 3).

To elucidate the influences of N-site isomerism on the electronic and band structures of the COFs, we carried out a comprehensive exploration of the physicochemical properties. The UV/Visible diffuse reflectance spectroscopy (UV/Vis DRS) revealed distinct light-adsorption profiles of three isomers (Fig. 3a). TA-33Bpy exhibited the most red-shifted absorption edges at 723 nm, compared with 701 nm for TA-22Bpy and 660 nm for TF-22Bpy. This broader visible-light response can be attributable to its more extended π-conjugation and enhanced electron delocalization. The degree of electron delocalization was further probed by solid-state electronic paramagnetic resonance (EPR) spectroscopy (Supplementary Fig. 12). Under identical measurement conditions, all three COFs showed a characteristic EPR signal with the g factor of 2.003, corresponding to the delocalized electrons in the aromatic frameworks^50,51^. Notably, the signal intensity followed an order TA-33Bpy > TA-22Bpy > TF-22Bpy, indicating progressively stronger electron delocalization from TF-22Bpy to TA-33Bpy. The Tauc plots derived from the UV-Vis DRS data showed that TA-33Bpy possesses the narrowest band gap energies (*E*_*g*_ = 1.88 eV), compared with 1.90 eV for TA-22Bpy and 2.13 eV for TF-22Bpy (Supplementary Fig. 13a). A smaller band gap generally favors charge polarization and transfer.

To obtain the electronic band positions, Mott−Schottky (M-S) measurements were carried out (Supplementary Fig. 13b-d). All samples displayed positive slopes in their M-S plots, confirming the n-type semiconductor behavior. It is well known that the conduction-band (CB) minimum position is close to its flat band potential for an n-type semiconductor^52^. Consequently, the CB potentials were thus determined to be −0.84 V vs NHE for TA-33Bpy, −0.37 V vs NHE for TF-22Bpy, and −0.39 V vs NHE for TA-22Bpy. From these values and optical band gaps, the valence band (VB) potentials were calculated as 1.04, 1.76 and 1.51 V vs NHE for TA-33Bpy, TF-22Bpy, and TA-22Bpy, respectively. The resulting band-edge alignments are summarized in Fig. 3b. These results clearly demonstrated that N-site isomerism exerts a pronounced effect on the electronic structure of the COFs. In particular, the position of imine-N appears to primarily modulate the band-gap width, whereas the arrangement of pyridine-N strongly influences the absolute energy levels of both CB and VB. In addition, electrochemical impedance spectroscopy (EIS) was employed to evaluate the interfacial charge-transfer kinetics (Fig. 3c). The Nyquist plot of TA-33Bpy displayed a smaller semicircle in the high-frequency region and a steeper slope in the low-frequency region than those of TF-22Bpy and TA-22Bpy, indicating a lower charge-transfer resistance and more efficient charge transport in TA-33Bpy.

The PMS activation performance of the isomeric COFs was evaluated by monitoring the oxidative degradation of BPA as a model reaction, which allows us to directly correlate the catalytic activity with the modulated electronic structures of the COFs. In the absence of PMS, BPA adsorption capacities of TF-22Bpy, TA-22Bpy, and TA-33Bpy were 32.8%, 39.0%, and 44.7%, respectively (Supplementary Fig. 14a). Upon addition of PMS, TA-33Bpy exhibited a markedly better BPA removal performance compared to the other two isomers (Fig. 3d). Specifically, the TA-33Bpy/PMS system achieved 97.5% BPA removal within 20 min, far exceeding the efficiencies of TF-22Bpy/PMS (25.5%) and TA-22Bpy/PMS (65.4%). The reaction kinetics were further analyzed using a pseudo-first-order model. The observed rate constant (*k*_obs_) for TA-33Bpy/PMS system was 0.165 min^−1^, representing a 12.7-fold and 3.2-fold enhancements compared to the TF-22Bpy/PMS (0.013 min^−1^) and TA-22Bpy/PMS (0.052 min^-1^) systems, respectively (Supplementary Fig. 14b). Moreover, the mineralization efficiency, assessed by total organic carbon (TOC) removal, reached approximately 48.6% in the TA-33Bpy/PMS system. This value is about 2.53 and 1.3 times higher than those achieved by TF-22Bpy/PMS (19.2%) and TA-22Bpy/PMS (37.4%), respectively (Supplementary Fig. 15). To further isolate the contribution of surface area, the *k*_obs_ values were standardized based on the specific surface areas of three COFs. The resulting area-normalized *k*_obs_ values were 7.42 × 10^-5^, 2.03 × 10^-5^ and 0.57 × 10^-5^ for TA-33Bpy, TA-22Bpy and TF-22Bpy, respectively. This result indicated that the improvement in performance mainly originated from the intrinsic effect of the N-site isomerism in regulating the electronic structure. In order to conduct a more comprehensive comparison with various systems, a standardized kinetic model (k-value) was adopted, taking into account of the amount of catalyst, oxidant and pollutant concentrations. According to Supplementary Table 8, the k-value of TA-33Bpy exceeds that of the majority of metal-based and non-metal-based catalysts, highlighting the efficiency of TA-33Bpy/PMS system.

To identify the dominated reactive oxygen species (ROSs) responsible for BPA degradation, we conducted a series of quenching experiments and probe analyses. Methanol (MeOH), tert-butanol (TBA) and NaN_3_ were employed as scavengers of •OH/SO_4_^•‒^, •OH, and ^1^O_2_, respectively. As shown in Fig. 3e, the addition of MeOH slightly enhanced the degradation kinetics in TF-22Bpy/PMS (0.014 min^−1^) and TA-33Bpy/PMS systems (0.198 min^-1^), while slightly inhibiting the reaction in TA-22Bpy/PMS (0.045 min^−1^), suggesting a negligible contribution from •OH and SO_4_^•‒^ radicals. The inhibition observed upon TBA addition in all COFs/PMS systems is likely due to the hydrophobic nature of TBA, which may interfere with catalyst dispersion rather than effectively quenching •OH. Further evidence for the absence of free radicals was obtained using benzoic acid (BA) as a radical-specific probe. No significant BA removal was detected in any of the COFs/PMS systems (Supplementary Fig. 16), confirming that •OH and SO_4_^•‒^ were not generated. Similarly, nitro-tetrazolium blue chloride (NBT) tests showed no characteristic absorption at 530 nm, ruling out the presence of O_2_^•‒^ in three COFs/PMS systems (Supplementary Fig. 17)^53^. To assess the potential role of direct electron-transfer processes, open circuit potential and galvanic oxidation process (GOP) were conducted for TA-33Bpy/PMS system. The potential increased significantly upon PMS addition but remained unchanged after BPA introduction (Supplementary Fig. 18a). In the GOP test, the current increased to 4.02 mA upon PMS addition and stabilized at 3.04 mA after 200 min, whereas the BPA removal was negligible (Supplementary Fig. 18b-c), indicating that electron-transfer pathways did not contribute significantly to BPA degradation. Importantly, the addition of NaN_3_, a specific ^1^O_2_ scavenger, almost completely suppressed BPA degradation in all three systems (Fig. 3e). Moreover, replacing 10% (v/v) of the reaction solvent with D_2_O, which extends the half-life time of ^1^O_2_ from 2.9–4.6 μs in H_2_O to 22–70 μs, consistently enhanced the degradation efficiency (Supplementary Fig. 19). These results collectively confirmed ^1^O_2_ is the primary ROS governing BPA oxidation in all COFs/PMS systems.

To quantitatively verify ^1^O_2_ generation in the heterogeneous PMS system, we further conducted PMS consumption, ABDA-based ^1^O_2_ quantification, and FFA kinetic measurements. Within 20 min, the PMS consumption reached 50.2%, 23.8%, and 16.4% for TA-33Bpy, TA-22Bpy, and TF-22Bpy, respectively (Supplementary Fig. 20). Consistently, ABDA quantification confirmed ^1^O_2_ production of 0.122, 0.104, and 0.082 mM, respectively (Supplementary Fig. 21a). The corresponding PMS utilization efficiencies were calculated to be 37.25%, 67.35%, and 76.86%, indicating that the slower-reacting system used PMS more efficiently (Supplementary Fig. 21b). FFA kinetics further showed that the steady-state concentration (C*ss*) of ^1^O_2_ followed an order of TA-33Bpy (1.60 × 10^-12^M) > TA-22Bpy (1.00 × 10^-12^M) > TF-22Bpy (0.82 × 10^−12^M), in good agreement with the ABDA results (Supplementary Fig. 21c)^54,55^. Notably, the opposite trends in ^1^O_2_ productivity and PMS utilization efficiency suggest that more efficient oxidant use does not necessarily lead to higher apparent activity.

To gain deeper insight into the catalytic performance of the isomeric COFs for ^1^O_2_ generation via PMS activation, we quantitatively assessed their activity using scanning electrochemical microscopy (SECM) in a three-electrode configuration. This system consisted of a platinum ultrafine electrode (tip, diameter = 10 μm) as the working electrode, a Pt wire as the counter electrode, and an Ag/AgCl/3 M KCl electrode as the reference. The working electrode, immersed in a Na_2_SO_4_ electrolyte containing uniformly dispersed COFs, served as the cathode to detect the oxidative current arising from the electroactive ^1^O_2_ generated upon PMS introduction. As illustrated in Fig. 3f and Supplementary Fig. 22, the TA-33Bpy/PMS system exhibited the highest initial current response (−137 nA), surpassing TF-22Bpy/PMS (−60 nA) and TA-22Bpy/PMS (−125 nA). This higher current response directly correlates with a greater flux of generated ^1^O_2_, confirming the better efficiency of TA-33Bpy in activating PMS to produce ^1^O_2_. Over time, the current progressively declined and eventually reached a plateau, indicating that the system approaching a steady-state reaction regime.

EPR spin-trapping tests were employed to monitor the occurrence of ROSs in TA-33Bpy/PMS system. Taking 2,2,6,6-tetramethyl-4-piperidinol (TEMP) as the specific trapping agent of ^1^O_2_, a typical triplet signal corresponding to TEMP-^1^O_2_ was clearly detected, signifying the existence of ^1^O_2_ in TA-33Bpy/PMS system (Fig. 3g). In contrast, when 5,5-dimethyl-1-pyrroline N-oxide (DMPO) was employed to capture •OH and SO_4_^•‒^, only 5,5-dimethyl-2-pyrrolidone-N-oxyl (DMPOX) was observed (Supplementary Fig. 23a). The formation of DMPOX is known to arise from the oxidation of DMPO on the catalyst surface, indicating the absence of free •OH and SO_4_^•‒^ in the bulk solution and suggesting a surface-confined, non-radical oxidation pathway. Similarly, when DMPO was used to trap O_2_^•‒^ in the presence of 50% MeOH (v/v), only the DMPOX signal was observed, further confirming that no generation of O_2_^•‒^ (Supplementary Fig. 23b). These EPR findings were further supported by chemical trapping using 9,10-dimethylanthracene (DMA) and Singlet Oxygen Sensor Green (SOSG), two selective probes for ^1^O_2_. UV-vis spectra showed a clear decrease in the DMA adsorption peak, and high-resolution mass spectrometry (HR-MS) identified the corresponding oxidation product DMA-O_2_ (Fig. 3h and Supplementary Fig. 24)^20^. Moreover, a clear fluorescence signal of SOSG-EP was observed in Fig. 3i, showing an increased intensity with reaction time. Together, these results provide direct and complementary evidence that ^1^O_2_ is the predominant ROSs in the TA-33Bpy/PMS system.

### Origin of ^1^O_2_ generation and theoretical calculation

The interaction between PMS and COF catalysts was first examined by EPR method. Notably, a strong EPR signal at *g* = 2.003 was observed only when TA-33Bpy was brought into contact with PMS, whereas no signal was detected for pure PMS or TA-33Bpy suspension alone (Fig. 3g), indicating the generation of abundant unpaired electrons in the TA-33Bpy/PMS system. Similar EPR responses have been reported for chemically oxidized COFs treated with electrophilic oxidants such as iodine, where the oxidant acts as an electron acceptor and induces electron delocalization within the conjugated framework^56,57^. Considering the electrophilic nature of PMS, we therefore attribute the EPR signal to a PMS-triggered ground-state charge polarization within the donor-acceptor COF. Specifically, PMS is proposed to interact with the COF as an electron acceptor, withdrawing electron density from the conjugated skeleton and generating localized electron-deficient donor domains (D^+^) together with electron-enriched PMS-associated acceptor domains (A^–^). Consistent with this interpretation, the EPR intensity followed the order TA-33Bpy/PMS > TA-22Bpy/PMS > TF-22Bpy/PMS under identical conditions, indicating progressively stronger PMS-COF electronic interaction from TF-22Bpy to TA-33Bpy. Moreover, the signal intensity increased significantly with increasing COF dosage but remained nearly unchanged with increasing PMS concentration (Supplementary Figs. 25–26), confirming that the unpaired electrons mainly originated from the COF framework rather than from PMS itself.

To further probe the electronic consequence of PMS binding, we performed time-resolved photoluminescence (TR-PL) decay plots, transient absorption spectroscopy (TAS) and photocurrent tests were performed in the absence and presence of PMS. Although these techniques are frequently used in photocatalytic systems, here they serve as probes of interfacial electron transfer and carrier extraction induced by PMS. Upon PMS addition, both the amplitude-averaged and intensity-averaged fluorescence lifetimes were prolonged, and the contribution of the long-lived decay component increased markedly, indicating substantial perturbation of the excited-state relaxation pathway (Supplementary Fig. 27). Consistently, femtosecond TAS revealed pronounced changes in the decay kinetics after PMS introduction, whereas the photocurrent was markedly suppressed with little change in the electrochemical impedance (Supplementary Fig. 28-29). This combination indicates that PMS efficiently extracts electrons from the COF framework, rather than simply altering its conductivity. These results therefore support the role of PMS as an electron acceptor upon association with the COF.

**Fig. 4:**
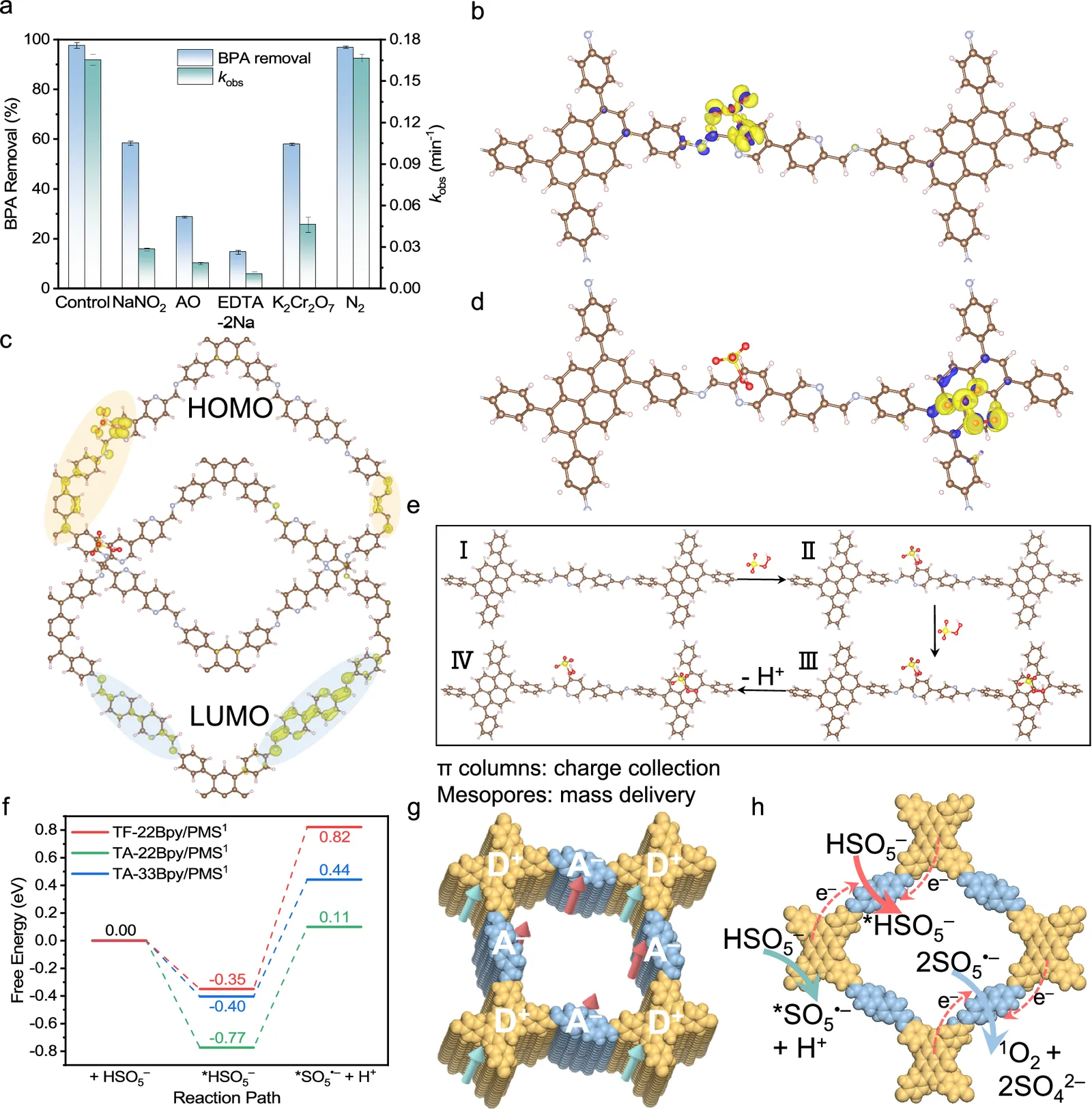
Origin of ^1^O_2_ generation. **a** Quenching experiments of electron-deficient sites, electron-enriched sites, SO_5_^•‒^ and DO in TA-33Bpy/PMS system. **b** EDDs of PMS^1^ adsorption model on TA-33Bpy. **c** HOMO and LUMO of TA-33Bpy/PMS^1^ system. **d** EDDs of PMS^2^ adsorption model on TA-33Bpy/PMS^1^ system. **e** The pathway and optimized atomic structures for the main process of the PMS activation on TA-33Bpy. **f** Free energy diagram for PMS^2^ activation in all COFs/PMS^1^ systems. **g** Ordered donor–acceptor π-columns (shown in one quadrangle of fourteen layers), illustrating the PMS-induced charge polarization within the framework, with acceptor columns (blue) associated with electron-enriched (A^‒^) regions and donor columns (orange) associated with electron-deficient (D^+^) regions. **h** Overall reaction scheme and one-layer square TA-33Bpy COF to depict the PMS-induced charge polarization (red arrow) from the D knot to A linker; HSO_5_^‒^ oxidation at D^+^ domains (green arrow); and SO_5_^•‒^ reduction to produce ^1^O_2_ at A^‒^ regions (blue arrow). Data for (**a**) are presented as mean values ± SD (*n* = 3).

To reveal the origin of ^1^O_2_ generation in COFs/PMS systems, we introduced NaNO_2_ as a specific scavenger of SO_5_^•‒^ intermediate^58^, which significantly suppressed BPA degradation (Fig. 4a). Combined with the earlier NBT probe results, this inhibition indicates that ^1^O_2_ is predominantly generated through the conversion of SO_5_^•‒^ rather than from O_2_^•‒^. To further explore the role of PMS-induced charge polarization in the process, we conducted quenching experiments using ammonium oxalate (AO) and EDTA-2Na as scavengers of electron-deficient sites, and K_2_Cr_2_O_7_ as a scavenger of electron-enriched sites. As depicted in Fig. 4a, the presence of AO, EDTA-2Na and K_2_Cr_2_O_7_ significantly reduced the BPA removal efficiency and *k*_obs_ to 28.7% (0.018 min⁻¹), 14.4% (0.010 min⁻¹), and 57.9% (0.046 min⁻¹), respectively. These results confirm that both electron-deficient donor (D^+^) and electron-rich acceptor (A⁻) regions actively participate in PMS activation for BPA degradation. Based on the above results, to separate PMS adsorption from PMS activation, we used EDTA-2Na to suppress PMS conversion, allowing the intrinsic PMS uptake by the COF frameworks to be evaluated. The PMS adsorption capacity was determined to be 23%, 8%, and 4.75% for TA-33Bpy, TA-22Bpy, and TF-22Bpy, respectively (Supplementary Fig. 20a-c). Moreover, the adsorption isotherms were well described by the Langmuir model, affording *q*_*m*_ values of 7868, 7188, and 7369 mg g^-1^, and *K*_*L*_ values of 0.466, 0.366, and 0.247 L g^-1^, respectively (Supplementary Fig. 30). Importantly, the *K*_*L*_ values show the same trend as the catalytic rates, with *k*_obs_ decreasing from 0.165 to 0.052 and 0.013 min^-1^ for TA-33Bpy, TA-22Bpy, and TF-22Bpy, respectively. This positive correlation indicates that stronger PMS affinity favors interfacial oxidant enrichment and contributes to the enhanced catalytic activity. Control experiments under N_2_ bubbling showed nearly unchanged catalytic activity, suggesting that dissolved oxygen (DO) makes a negligible contribution to ^1^O_2_ generation. The generation of these charge carriers was directly probed using 2,2,6,6-tetramethylpiperidine-1-oxyl (TEMPO) as a spin-trapping agent. TEMPO exhibits a characteristic triplet EPR signal, which diminishes upon reaction with polarized charges to form the spin-silent adduct^59,60^. In the TA-33Bpy/PMS system, the intensity of TEMPO signal decreased significantly, accompanied by the emergence of an unpaired electron signal attributable to the COF/PMS interaction (Supplementary Fig. 31). Together, these results support a mechanism in which PMS first associates with the COF framework, then induces interfacial charge polarization, and finally undergoes selective activation toward ^1^O_2_ generation.

To gain a deeper insight into the relationship between structure, electronic properties and catalytic activity, we carried out DFT calculations based on the ideal framework structures. Firstly, the electronic structure changes of the system were calculated when the first PMS molecule (PMS^1^) was adsorbed on COFs. In the optimized configuration, PMS^1^ preferentially adsorbs near the bipyridine (acceptor) units in all three systems. Electron density differences (EDDs) analysis revealed net charge transfer from COF framework to PMS^1^ (Fig. 4b and Supplementary Figs. 32–34). Notably, for TA-22Bpy and TA-33Bpy, the pyrene (donor) units also exhibited significant electron depletion, indicating that PMS induces framework-wide electronic polarization across the entire D-A framework. Further analysis of the frontier molecular orbitals in the COFs/PMS^1^ systems provided deeper insights into this polarized electronic state. In TA-33Bpy/PMS^1^, the HOMO remained localized on the pyrene unit and PMS-associated region, while the LUMO resided on the bipyridine linker, preserving a well-defined donor-acceptor orbital arrangement that is favorable for PMS-induced charge polarization (Fig. 4c). A similar but less distinct separation was observed in TA-22Bpy/PMS^1^, where the HOMO and LUMO were primarily located on the pyrene and bipyridine units, respectively, yet exhibited slight orbital overlap at the pyrene site (Supplementary Fig. 33c-d). In contrast, for the TF-22Bpy/PMS^1^ system, the HOMO was distributed over pyrene, bipyridine and PMS, while the LUMO was confined to PMS (Supplementary Fig. 34c-d). This stronger orbital overlap is less favorable for maintaining a polarized interfacial electronic state, consistent with the weaker EPR signal experimentally observed for TF-22Bpy/PMS^61^.

Bader charge analysis further quantified the PMS-induced charge polarization. The acceptor bipyridine channels in TA-33Bpy/PMS^1^ and TA-22Bpy/PMS^1^ carried net positive charges of +0.88 and +0.81, respectively. The larger extent of charge polarization in TA-33Bpy suggests a stronger electronic interaction with PMS and a greater driving force for interfacial electron redistribution. Besides, in-situ Raman and FT-IR analysis were employed to monitor the surface interaction between TA-33Bpy and PMS. The pristine PMS solution exhibited characteristic peaks of HSO_5_^‒^ at 882 and 1060 cm^-1^ and of SO_4_^2‒^ at 982 cm^-1^ (Supplementary Fig. 35). Upon addition of TA-33Bpy, a new peak emerged at 834 cm^-1^, attributed to surface-adsorbed PMS* species, directly confirming the catalyst-oxidant interaction at the solid-liquid interface^45^. As shown in Supplementary Fig. 36, with the addition of PMS, the characteristic peaks of the pyridine ring (728 cm^-1^) and the aromatic ring (821 and 833 cm^-1^) shifted and their intensities changed, indicating that PMS was adsorbed around the linkage.

Next, the adsorption of the second PMS molecule (PMS^2^) within the electron-deficient region of the COF/PMS^1^ system and its subsequent oxidation to SO_5_^•‒^ were simulated. In the optimized configuration, PMS^2^ in all three systems induced electron transfer from the pyrene (donor) unit to itself, primarily driven by the high electronegativity of PMS (Fig. 4d and Supplementary Figs. 37–S39)^44^. Calculation showed that the adsorption of the second PMS to form PMS^2*^ is spontaneous in all three COF/PMS^1^ systems, with adsorption free energies of -0.35 eV for TF-22Bpy/PMS^1^, -0.77 eV for TA-22Bpy/PMS^1^, and −0.40 eV for TA-33Bpy/PMS^1^ (Fig. 4f). The subsequent dehydrogenation step to generate SO_5_^•‒^ exhibited the lowest energy barrier in the TA-33Bpy/PMS^1^ system (+0.84 eV), compared with TF-22Bpy/PMS^1^ ( + 1.17 eV) and TA-22Bpy/PMS^1^ ( + 0.88 eV), indicating that the TA-33Bpy/PMS^1^ system is more favorable for SO_5_^•‒^ formation. Once formed, SO_5_^•‒^ rapidly undergoes self-reaction to yield SO_4_^2‒^ and ^1^O_2_. Importantly, the PMS-induced polarized electronic state in TA-33Bpy/PMS^1^ enables the coupled redox steps required for both PMS oxidation and SO_5_^•‒^ conversion, thereby promoting selective ^1^O_2_ generation. This is fully consistent with quenching experiments presented earlier.

Based on these computational and experimental findings, we propose the following mechanism for ^1^O_2_ generation in the TA-33Bpy/PMS system (Fig. 4e and Supplementary Figs. 40–41). (i) Electrophilic PMS interacts with the donor-acceptor framework and induces charge polarization, generating localized electron-deficient (D^+^) and electron-enriched (A^‒^) domains; (ii) Additional HSO_5_^‒^ molecules were oxidized at the electron-deficient region to form SO_5_^•‒^; (iii) SO_5_^•‒^ was reduced at the electron-enriched region to produce ^1^O_2_. Structurally, the donor (pyrene) and acceptor (bipyridine) units in the TA-33Bpy are orderly arranged within the x-y plane, which is favorable for establishing PMS-induced charge polarization, while their columnar stacking along the z direction supports efficient interfacial electronic communication (Fig. 4g)^43,61,62^. Furthermore, the one-dimensional mesoporous channels of TA-33Bpy enhance mass transport and provide a confined microenvironment for pollutants degradation by ^1^O_2_. Within this architecture, the D^+^ domains preferentially participate in HSO_5_^‒^ oxidation (Fig. 4h, green arrow), while A^‒^ domains promote ^1^O_2_ formation via SO_5_^•‒^ reduction (Fig. 4h, blue arrow).

### Fenton-like performance of TA-33Bpy/PMS system

The influence of COF loading on BPA removal was systematically investigated (Supplementary Fig. 42). Increasing the catalyst dosage from 0.05 to 0.2 g L^-1^ led to a corresponding increase in the reaction rate constant from 0.09 to 0.33 min⁻¹. To further evaluate the environmental adaptability of the TA-33Bpy/PMS system, we examined the effects of common water constituents and solution pH on BPA degradation. As shown in Supplementary Fig. 43, the presence of NO_3_^-^, Cl^-^, HCO_3_^-^, SO_4_^2-^ and HPO_4_^2-^ exerted a negligible influence on both BPA removal efficiency and *k*_obs_. Interestingly, in the presence of Br^-^, BPA was rapidly removed within 1 min even without catalyst addition, which is likely related to the relatively high nucleophilicity of bromide species. Moreover, complete BPA removal could still be achieved within 1 min in mixed systems containing Br^-^, Cl^-^ and NO_3_^-^, while HPLC-MS analysis confirmed the absence of typical halogenated byproducts. To investigate the influence of natural organic matter, we further evaluated the catalytic performance in the presence of different concentrations of humic acid (HA). As depicted in Supplementary Fig. 44, BPA removal efficiency and reaction kinetics gradually decreased with an increasing HA concentration, likely due to competitive ROS consumption and partial blockage of catalytic active sites by HA adsorption. The effect of solution pH was also examined. Acidic conditions favored BPA degradation, whereas alkaline conditions suppressed the reaction (Supplementary Fig. 45a). Zeta-potential measurements showed that the surface of TA-33Bpy became progressively more negatively charged with increasing pH (Supplementary Fig. 45b). Such deprotonation-induced surface charge variation likely enhanced the electrostatic repulsion between TA-33Bpy and anionic HSO_5_^‒^ species, thereby reducing the interfacial PMS activation efficiency. Despite this, TA-33Bpy maintained effective catalytic activity across a wide pH range from acidic to weakly alkaline solutions.

The universality of the TA-33Bpy/PMS system was further evaluated using a variety of representative pollutants, including rhodamine B (RhB), methyl orange (MO), Congo red (CR), sulfanilamide (SA), sulfamethoxazole (SMX), carbamazepine (CBZ), and acetaminophen (AcP). As shown in Supplementary Fig. 46, MO, CR, and AcP were rapidly degraded, while sulfonamides and RhB exhibited relatively lower removal efficiencies. In contrast, only 7.0% of CBZ was removed under the same conditions. These results indicate that the TA-33Bpy/PMS system possesses broad applicability and shows preferential reactivity toward electron-rich contaminants that are neutral or negatively charged under acidic conditions. In addition, BPA degradation was further examined in real water matrices, including a secondary effluent and surface water. As shown in Supplementary Fig. 47, a slight inhibition of BPA removal was observed in both cases, which is likely attributable to the presence of coexisting constituents in the water matrix that compete for reactive species or interfere with PMS activation. To further validate its practical potential, we evaluated the long-term performance of TA-33Bpy in a continuous-flow column setup. A solution containing 5 ppm BPA and 0.65 mM PMS was passed through the catalyst bed at a flow rate of 2.2 mL/min, corresponding to a hydraulic retention time of 9.1 min. After 48 h of continuous operation (total treated water volume: 6.336 L), BPA removal efficiency remained above 73% (Supplementary Fig. 48). Collectively, the TA-33Bpy/PMS system exhibits excellent environmental adaptability, robust stability, and sustained catalytic performance, confirming its strong potential for practical water purification applications.

To elucidate the degradation mechanism and environmental safety of this process, we identified the degradation products of BPA by high-performance liquid chromatography-mass spectrometry (HPLC-MS), and proposed a plausible degradation pathway (Supplementary Fig. 49). The identified intermediates suggest that BPA undergoes sequential ^1^O_2_-driven oxidation and ring-cleavage reactions, ultimately yielding small organic acids and, eventually, CO_2_ and H_2_O. Meanwhile, analysis of the methanol-eluted fraction from the spent catalyst surface revealed BPA oligomers, indicating that part of BPA follows a parallel surface-confined oligomerization/polymerization pathway. This interpretation is consistent with recent studies showing that selective nonradical oxidation in persulfate-based AOPs, including ^1^O_2_-mediated pathways, can convert phenolic substrates into phenoxy-radical-type intermediates that further couple on catalyst surfaces^63^. The accumulation of these surface-bound products may facilitate initial pollutant removal, but persistent deposition could progressively block active sites and impede interfacial mass/electron transfer, thereby lowering catalyst stability and long-term catalytic efficiency. Furthermore, the ecological toxicity of the detected aqueous intermediates was evaluated using ECOSAR software (version 2.2) based on quantitative structure-activity relationship (QSAR) models. As shown in Supplementary Fig. 50, both acute and chronic toxicity endpoints decreased along the degradation pathway, indicating effective detoxification during the treatment process.

The stability of the catalyst was evaluated through a comprehensive characterization of TA-33Bpy after the continuous-flow reaction. PXRD patterns confirmed that the crystalline framework remained largely intact, with no obvious loss of long-range order (Supplementary Fig. 51). HR-TEM further revealed well-resolved lattice fringes, indicating preserved local crystallinity (Supplementary Fig. 52). N_2_ adsorption-desorption results showed that the specific surface area, pore volume and pore size decreased from 2223, 1.29 and 2.48 to 777 m²/g, 0.50 cm^3^/g and 2.38 nm, respectively (Supplementary Fig. 53). This reduction can be attributed to partial framework distortion and pore blocking by adsorbed reaction products, such as BPA oligomers. FT-IR spectroscopy revealed the disappearance of the characteristic imine (C = N) stretching vibration at 1610 cm^−1^ and the emergence of a new band at 1655 cm^−1^, which is assignable to the carbonyl (C = O) stretch of amide linkages (Supplementary Fig. 54)^64,65^. This transformation was corroborated by XPS spectroscopy. The survey scan indicated an increase in oxygen content after reaction. Furthermore, the high-resolution N 1 *s* and O 1 *s* peaks shifted from 399.4 and 532.6 eV to 399.7 and 533.2 eV, respectively (Supplementary Fig. 55), consistent with the transformation from imine to amide functional groups.

Furthermore, recycling experiments were performed, and the catalysts recovered after each cycle were systematically characterized to track their structural evolution during operation. As shown in Supplementary Fig. 56, the reaction rate gradually decreased with increasing cycle number, accompanied by slight reductions in specific surface area and pore volume, whereas the pore size remained essentially unchanged (Supplementary Fig. 57). In addition, the PXRD patterns showed negligible changes in crystallinity throughout the cycling process (Supplementary Fig. 58). These results suggest that the decrease in surface area and partial pore blockage mainly originated from the accumulation of BPA-derived polymeric products during continuous-flow operation, which could be largely removed by methanol washing. To minimize the influence of surface-area variation, the observed rate constants (*k*_obs_) were further normalized by the specific surface area for each cycle (Supplementary Table 9). The area-normalized kinetic constant began to decline noticeably after the fourth cycle, indicating a gradual decrease in the intrinsic catalytic activity. To further probe the chemical evolution of the framework, we conducted XPS analyses on the catalysts collected after different cycles. As shown in Supplementary Fig. 59, the amide-related signal progressively increased, while the imine-related signal gradually weakened with increasing cycle number. Notably, the imine species nearly disappeared after the fourth cycle, consistent with the decline in the area-normalized activity, suggesting that the imine linkage plays a critical role in PMS activation (Supplementary Table 10). The gradual loss of catalytic activity was mainly attributed to the limited stability of imine linkages under prolonged oxidative conditions. Accordingly, periodic regeneration through organic-solvent washing followed by mild drying, together with post-synthetic conversion of imine linkages into more robust heterocyclic linkages while preserving their protonatable character for PMS interaction, may provide effective strategies for improving the long-term durability of TA-33Bpy.

## Discussion

In summary, we have developed a N-site isomeric strategy to fine-tune the electronic structures of D-A COFs by precisely modulating the positions of imine and pyridine nitrogen atoms. This structural control directly translates to enhanced Fenton-like catalytic activity, with TA-33Bpy exhibiting the highest performance in activating PMS. The observed rate constant *k*_obs_ (0.165 min^-1^) for BPA degradation represents a 12.7-fold and 3.2-fold improvement over TF-22Bpy (0.013 min^-1^) and TA-22Bpy (0.052 min^-1^), respectively. By combining experimental investigations with density functional theory calculations, we reveal that direct PMS–COF interaction induces charge polarization along the donor–acceptor π-columns for the first time. This polarized electronic configuration enables coupled PMS oxidation and SO_5_^•‒^ reduction, resulting in predominant generation of singlet oxygen (^1^O_2_). This work highlights the decisive role of PMS-triggered charge polarization in mediating PMS activation and establishes, for the first time, N-site isomeric engineering as an isomerism-guided design strategy for COF-based PMS activation, with potential applicability to broader nitrogen-containing COFs and catalytic reactions.

## Methods

### Synthesis of TF-22Bpy COF

In a typical synthesis process, TFPPy (30 mg, 0.048 mmol), 22Bpy-NH_2_ (17.8 mg, 0.096 mmol), o-DCB (1 mL), n-BuOH (1 mL) and HAc (6 M, 0.2 mL) were added to a 10 mL ampoule. After sonication for 10 –15 min, the ampoule was sealed after degassing by three freeze-pump-thaw cycles, followed by heating at 120 °C for 3 d. After cooling down to room temperature, the precipitate was collected by filtration and washed with DMF, THF and EtOH. The purified COFs were further dried at 60 °C under vacuum to yield yellow powder.

### Synthesis of TA-22Bpy COF

In a typical synthesis process, TAPPy (42.5 mg, 0.075 mmol), 22Bpy-CHO (32 mg, 0.15 mmol), Diox (1.5 mL), TMB (1.5 mL) and HAc (3 M, 0.5 mL) were added to a 10 mL ampoule. After sonication for 10 – 15 min, the ampoule was sealed after degassing by three freeze-pump-thaw cycles, followed by heating at 120 °C for 3 d. After cooling down to room temperature, the precipitate was collected by filtration and washed with DMF, THF and EtOH. The purified COFs were further dried at 60 °C under vacuum to yield yellow powder.

### Synthesis of TA-33Bpy COF

In a typical synthesis process, TAPPy (42.5 mg, 0.075 mmol), 33Bpy-CHO (32 mg, 0.15 mmol), Diox (1.5 mL), TMB (1.5 mL) and HAc (3 M, 0.5 mL) were added to a 10 mL ampoule. After sonication for 10 – 15 min, the ampoule was sealed after degassing by three freeze-pump-thaw cycles, followed by heating at 120 °C for 3 d. After cooling down to room temperature, the precipitate was collected by filtration and washed with DMF, THF and EtOH. The purified COFs were further dried at 60 °C under vacuum to yield orange powder.

## Supplementary information

Supplementary Information

Transparent Peer Review file

## Source data

Source Data Figure 2

Source Data Figure 3

Source Data Figure 4

## Data Availability

The authors declare that the data generated in this study are provided in the Supplementary Information and Source Data file. Source data are provided with this paper. All data are available from the corresponding author upon request. Source data are provided with this paper.
