# Supplementary material for: Engineering N-site isomerism in donor-acceptor covalent organic frameworks for efficient Fenton-like water purification
Source: Nat Commun. 2026 Aug 7;17:9492. doi: 10.1038/s41467-026-76345-2 (PMC13542144; doi:10.1038/s41467-026-76345-2)
Supplement: Supplementary file 1 [file 41467_2026_76345_MOESM1_ESM.pdf]

Supplementary Information

Engineering N-site isomerism in donor-acceptor covalent organic frameworks for efficient Fenton-like water purification

Chengming Xiao[1], Mengjia He[1], Wenbo Yang[1], Yuxin Tian[1], Junwen Qi[1], Zhigao Zhu[1], Yujun Zhou[1], Yue Yang[1], Xiaoguang Duan[2], Shaobin Wang[2*], Jiansheng Li[1*]

[1]*Jiangsu Key Laboratory of Chemical Pollution Control and Resources Reuse, School of Environmental and Biological Engineering, Nanjing University of Science & Technology, Nanjing 210094, People's Republic of China*

[2]*School of Chemical Engineering, Adelaide University, Adelaide, South Australia 5005, Australia*

E-mail: shaobin.wang@adelaide.edu.au; lijsh@njust.edu.cn

Materials

All chemicals were purchased from commercial sources and used without further treatment. OXONE ($KHSO_5 \cdot 0.5KHSO_4 \cdot 0.5K_2SO_4$) and 2,2,6,6-tetramethyl-4-piperidone (TEMP) were purchased from Sigma Aldrich Chemicals. 2,2'-Bipyridine-5,5'-diamine (22Bpy-NH2), 1,3,6,8-tetra(4-formylphenyl)pyrene (TFPPy), 4,4',4'',4'''-(Pyrene-1,3,6,8-tetrayl)tetraaniline (TAPPy), [2,2'-Bipyridine]-5,5'-dicarbaldehyde (22Bpy-CHO) and [3,3-Bipyridine]-6,6-dicarboxaldehyde (33Bpy-CHO) were purchased from Jilin Chinese Academy of Sciences-Yanshen Technology Co., Ltd. Nitro-tetrazolium blue chloride (NBT), deuterium oxide (D_2O, 99.9 atom% D), 5,5-dimethyl-1-pyrroline N-oxide (DMPO), benzoic acid (BA), 1,4-dioxane (Diox), mesitylene (TMB), 1,2-dichlorobenzene (o-DCB), n-butyl alcohol (n-BuOH) and acetic acid (HAc) were purchased from Shanghai Aladdin Biochemical Co., Ltd. Ethanol (EtOH), methanol (MeOH), tert butyl alcohol (TBA), N,N-dimethylformamide (DMF), sodium hydroxide (NaOH), sodium chloride (NaCl), sodium sulfate (Na_2SO_4), sodium nitrite ($NaNO_2$), sodium nitrate ($NaNO_3$), sodium bicarbonate ($NaHCO_3$) and sodium dihydrogen phosphate (NaH_2PO_4) were purchased from Nanjing Chemical Reagent Co., Ltd. Singlet Oxygen Sensor Green (SOSG) were purchased from Shanghai Adamas Reagent Co., Ltd.

Characterizations

Scanning electron microscopy (SEM) and transmission electron microscopy (TEM) were conducted on JEOL JSM 7800F and JEM F200, respectively. Powder X-ray diffraction (PXRD) patterns were obtained on a Bruker D8 instrument with Cu Kα radiation (λ = 1.54 Å) operating at 40 kV and 40 mA. The PXRD patterns were collected at a scanning speed of 0.02° s^{-1} in the range of 2°–30°. Fourier transform infrared spectroscopy (FT-IR) analyses of the samples were performed on a Nicolet iS5 spectrometer. N_2 adsorption and desorption measurements were conducted on a Micromeritics ASAP-2020 instrument with degassing temperature of 120 °C for 12 h before the measurement. The specific surface areas were calculated by using the Brunauer–Emmett–Teller (BET) calculations and the pore size distributions were obtained from the adsorption branch of isotherms by the non-localized density functional theory (NLDFT) model. Scanning electrochemical microscopy (SECM) analysis was conducted on a Princeton

VersaSCAN. UV-Vis diffuse reflectance spectra (UV-Vis DRS) were recorded on a UV-visible spectrophotometer (UV-2600, Shimadzu Corp., Japan), with an integrating sphere attachment and $BaSO_4$ reference. The analysis of degradation products was measured by ultra-high performance liquid chromatography-quadrupole time-of-flight mass spectrometry (UHPLC-Q-TOF MS, AB SCIEX Triple TOF 5600+). The measurement of electron paramagnetic resonance (EPR) was conducted on a Bruker A300.

Electrochemical tests

All electrochemical experiments (electrochemical impedance spectroscopy (EIS) and open circuit potential (OCP)) were carried out using a CHI760E three electrochemical workstation. For the preparation of working electrodes, 5 mg of catalyst was dispersed into a mixture of 0.45 mL of ethanol and 0.05 mL of Nafion® solution, followed by sonication for 3 h. The suspension (2 μL) was loaded onto a glassy carbon electrode and repeated dropwise two to three times for preparing a COFs-coated glassy carbon electrode (COFs GCE). Pt and silver/silver chloride (Ag/AgCl) electrodes were used as a counter electrode and a reference electrode, respectively. The electrolyte was 0.5 M Na_2SO_4 aqueous solution (pH 6.8).

(1) EIS was measured at open potential with a scan frequency in the range from 10^5 to 10^{-1} Hz.

(2) OCP of COFs GCE was monitored by chronopotentiometry analysis. The changing potential was monitored after adding PMS or BPA.

Mott-Schottky curves

Indium tin oxide (ITO) glasses, platinum plate electrode and saturated calomel electrode (SCE) were used as working electrode, assistant electrode and reference electrode, respectively. For the preparation of working electrodes, 5 mg of catalyst was dispersed into a mixture of 0.45 mL of ethanol and 0.05 mL of Nafion® solution, followed by sonication for 3 h. The suspension (50 μL) was loaded onto a ITO electrode and repeated dropwise two times for preparing a COFs-coated ITO electrode. Mott-Schottky curves were obtained by immersing COFs PPE into a 0.5 M Na_2SO_4 aqueous solution. The transformation among SCE, NHE and RHE is showed below:

$$E(\text{RHE}) = E(\text{SCE}) + 0.0591 \times \text{pH} + 0.241 \quad (1)$$

$$E(\text{NHE}) = E\ (\text{SCE}) + 0.241 \quad (2)$$

Transient photocurrent response

Indium tin oxide (ITO) glasses, platinum plate electrode and saturated calomel electrode (SCE) were used as working electrode, assistant electrode and reference electrode, respectively. For the preparation of working electrodes, 5 mg of catalyst was dispersed into a mixture of 0.45 mL of ethanol and 0.05 mL of Nafion® solution, followed by sonication for 3 h. The suspension (50 μL) was loaded onto a glassy carbon electrode and repeated dropwise two times for preparing a COFs-coated platinum plate electrode (COFs PPE). A 300 W xenon lamp was used as the light source, and the transient photocurrent with a bias of 0 V was tested over time during the on/off light cycle.

Galvanic oxidation process

For the preparation of working electrodes, 5 mg of catalyst was dispersed into a mixture of 0.45 mL of ethanol and 0.05 mL of Nafion® solution, followed by sonication for 3 h. The suspension (200 μL) was uniformly coated onto a graphite plate with dimensions of 20 mm × 20 mm × 3 mm. The plate was then dried in a 60 ºC oven. The two graphite plates were separated and placed in two electrolyte chambers with 0.5 M Na_2SO_4 containing 20 ppm BPA and 0.1 g L^{-1} PMS, respectively, and connected by agar salt bridges to maintain charge balance during the reaction process. The current changes during the reaction were monitored in real time using the CHI 760E electrochemical workstation.

Biotoxicity assessment

The ECOSAR program (version 2.0) was used to predict the acute and chronic toxicity of BPA and its degradation intermediates to fish, daphnids, and green algae. The LC_{50} (median lethal concentration for fish and daphnid after 96 h and 48 h exposure, respectively) and EC_{50} values (maximal effective concentration for inhibiting 50% growth of green algae after 96 h exposure) represent acute toxicity. Chronic toxicity is expressed by ChV values.

Experimental Procedures

Various conditional experiments were conducted in a 100 mL of beaker. In a typical run, 25 mL of distilled water and 25 mL of BPA (40 ppm) were mixed, and 5 mg of catalyst was

added to reach the adsorption equilibrium. With 10 mg PMS added, at a selected reaction period (0, 1, 3, 5, 7, 10, 15 and 20 min), 0.75 mL of solution was removed from the beaker and added into another 0.75 mL of methanol (MeOH) in a 2 mL tube. An organic phase filter membrane (0.22 μm) was used to remove the catalyst, and the concentration of BPA was detected by high performance liquid chromatography (HPLC). Quenching experiments were performed by adding MeOH ($SO_4^{•-}$, $^•OH$), TBA ($^•OH$), NaN_3 ($^1O_2$), $NaNO_2$ ($SO_5^{•-}$), AO (Electron-deficient site), EDTA-2Na (Electron-deficient site) and $K_2Cr_2O_7$ (Electron-rich sites) in the degradation system as the scavengers. For the influence of co-existing ion and matrix, the concentrations of anions and HA were 5 mM and 5 ppm, respectively. To explore the effect of pH on BPA degradation, we adjusted the pH of solution by adding either NaOH or H_2SO_4. As for recycling experiment, the used catalyst was washed with ethanol to remove the oligomeric products and then dried in a vacuum oven at 60 °C.

Continuous-flow performance

The catalyst (100 mg) was dispersed in a 20 mL flow column. A 10 mg/L BPA solution and a 1.3 M PMS solution were introduced into the reaction column from two different pipelines at a rate of 1.1 mL per second from bottom to top, and the hydraulic retention time was 9.1 min.

Identification of $SO_4^{•-}$, $^•OH$, and $O_2^{•-}$

BA was employed as a probe of $SO_4^{•-}$ and $^•OH$. TA-33Bpy (5 mg) and 10 mg PMS were added into 50 mL BA solution (20 ppm). The concentration of BA was measured by HPLC. Nitro-tetrazolium blue chloride (NBT) was used as a probe to detect $O_2^{•-}$. TA-33Bpy (5 mg) and 10 mg PMS were added into 50 mL NBT solution (1 mM). After a defined time interval, 2 mL sample was taken out from the quartz reactor using a syringe and filtered through a 0.22 μm PTFE filter. The yellow NBT was reduced by $O_2^{•-}$ to blue formazan, which could be measured by UV-Vis-DRS at 530 nm.

9,10-Dimethylanthracene (DMA) measurement

DMA (50.0 μM) was reacted in a 50.0 wt.% acetonitrile-water solution. The decomposition of DMA was monitored using a UV-vis spectrometer. The reaction products were identified using a high-performance liquid chromatography coupled with mass

spectrometry (Orbitrap Fusion, Thermo Fisher Inc., USA). The mass spectrometry signals for DMA and 9,10-dimethylanthracene-endoperoxide (DMA-O_2) were recorded in the mass range of 100- 450 m/z using an electrospray ion source in a positive ion mode. These signals were utilized to determine the retention time for DMA and DMA-O_2. The conversion of DMA to DMA-O_2 was quantified by measuring the absorbance at 258 nm using the molar absorption coefficients for DMA and DMA-O_2 at this wavelength.

SOSG probe measurement

The generation of $^1O_2$ was detected by an SOSG probe measurement. Initially, SOSG (100 μg) was dissolved in 300 μL of MeOH to obtain a SOSG solution with a concentration of 500 μM, which was freshly prepared prior to each use. Catalysts (1 mg) and H_2O (10 mL) were added into a 25 mL glass beaker, followed by sonication for 10 min to ensure that the material was completely dispersed. Afterward, 150 μL of SOSG solution was added into the beaker; then, PMS (100 mM, 100 μL) was added to initiate the reaction. After 5, 10, 15, and 20 min, 1 mL of the sample was withdrawn and added to 1 mL MeOH. The obtained sample was immediately filtered with a PTFE syringe filter (0.22 μm). Finally, the sample was assessed by using a fluorescence spectrometer (Lengguang, F97). The excitation wavelength was set at 488 nm, and the emission wavelength range was 500−600 nm.

Electronic paramagnetic resonance (EPR) measurement

To characterize the charge polarization of COFs induced by PMS, we added 250 μL of PMS aqueous solution (0.4 g L^{-1}) to 250 μL of the COFs dispersion solution (0.2 g L^{-1}), followed by vigorously shaken for 5 seconds to ensure complete mixing. After adsorbing the reaction solution with a capillary, the EPR signal was detected by Bruker A300. Similarly, in order to detect TEMP-$^1O_2$ and DMPO-$SO_4^{\bullet-}$/$^{\bullet}OH$, the COF dispersion solutions were prepared containing 50 mM TEMP and 200 mM DMPO, respectively. As for DMPO-$O_2^{\bullet-}$, the COFs (0.2 g L^{-1}) were dispersed into a methanol solution containing 200 mM DMPO.

Determination of degradation kinetics

The degradation kinetics of the contaminants followed pseudo-first-order kinetics, and the reaction rate constants (k_{obs}) were calculated using the following Eq. 3:

$$\ln\left(\frac{C_t}{C_0}\right) = -k_{obs}t \quad (3)$$

where C_t is the concentration of the contaminant at time t, and C_0 is the initial concentration at time t=0.

DFT Calculations

All COFs structures were fully optimized in vacuum without considering solvent effects. The calculations were performed within the framework of density functional theory (DFT) using the projector augmented-wave (PAW) method, as implemented in the Vienna *ab initio* simulation package (VASP). The Perdew-Burke-Ernzerhof (PBE) functional within the generalized gradient approximation (GGA) was employed to describe the exchange-correlation potential, and long-range van der Waals interactions were included using the DFT-D3 method. The cut-off energy for plane wave is set to be 400 eV. The energy criterion is set to 10^{-5} eV in iterative solution of the Kohn-Sham equation. The Brillouin zone integration is performed at the Gamma point. All the structures are relaxed until the residual forces on the atoms have declined to less than 0.05 eV/Å. The Gibbs free energies are defined as following Eq. 4:

$$G = E + E_{ZPE} - TS \quad (4)$$

Where, E, E_{ZPE}, and TS refer to the total energy of the system, the zero-point energy, and the entropy, respectively.

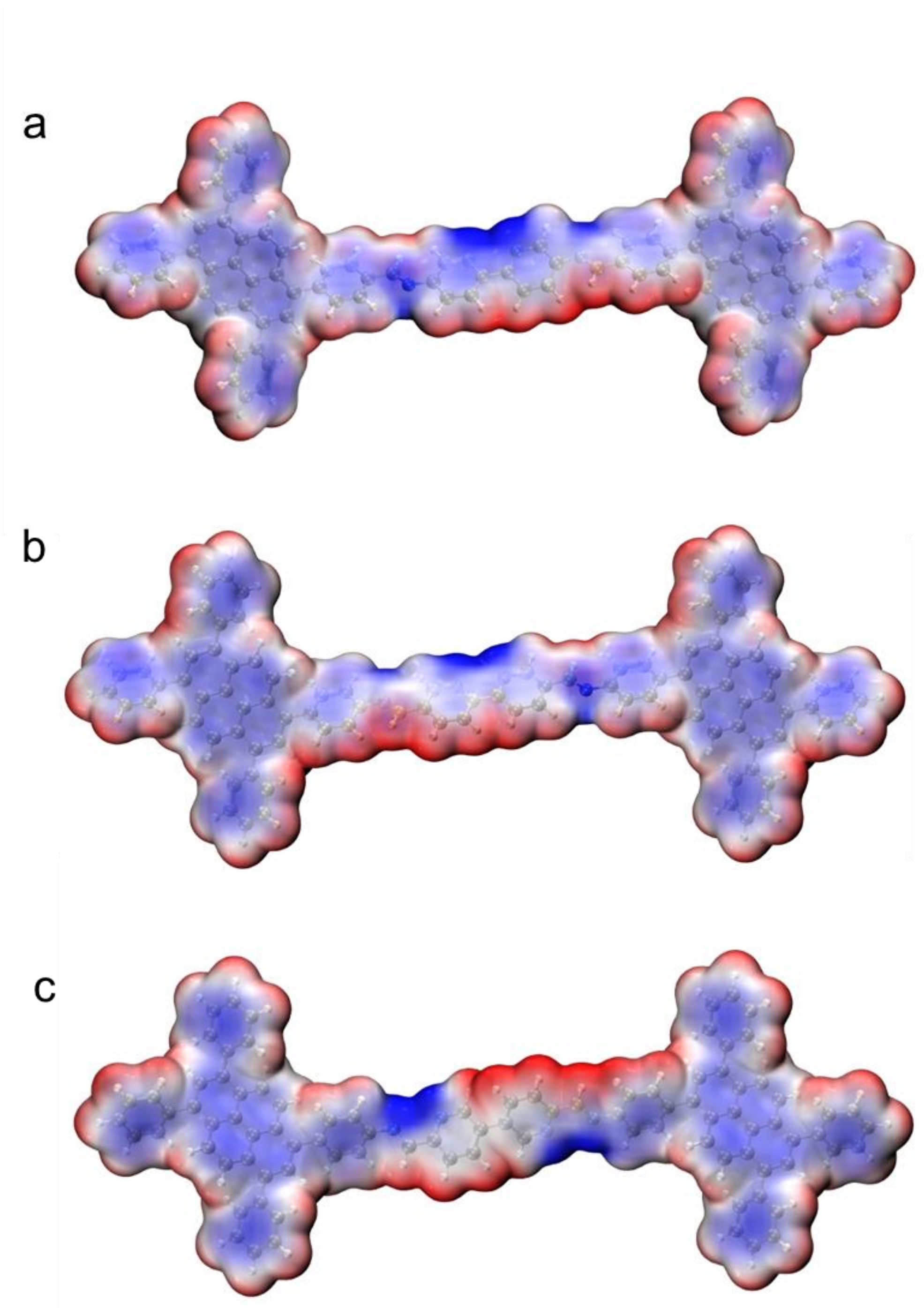

Supplementary Fig. 1 Electrostatic potential profiles of (a) TF-22Bpy, (b) TA-22Bpy and (c) TA-33Bpy COF.

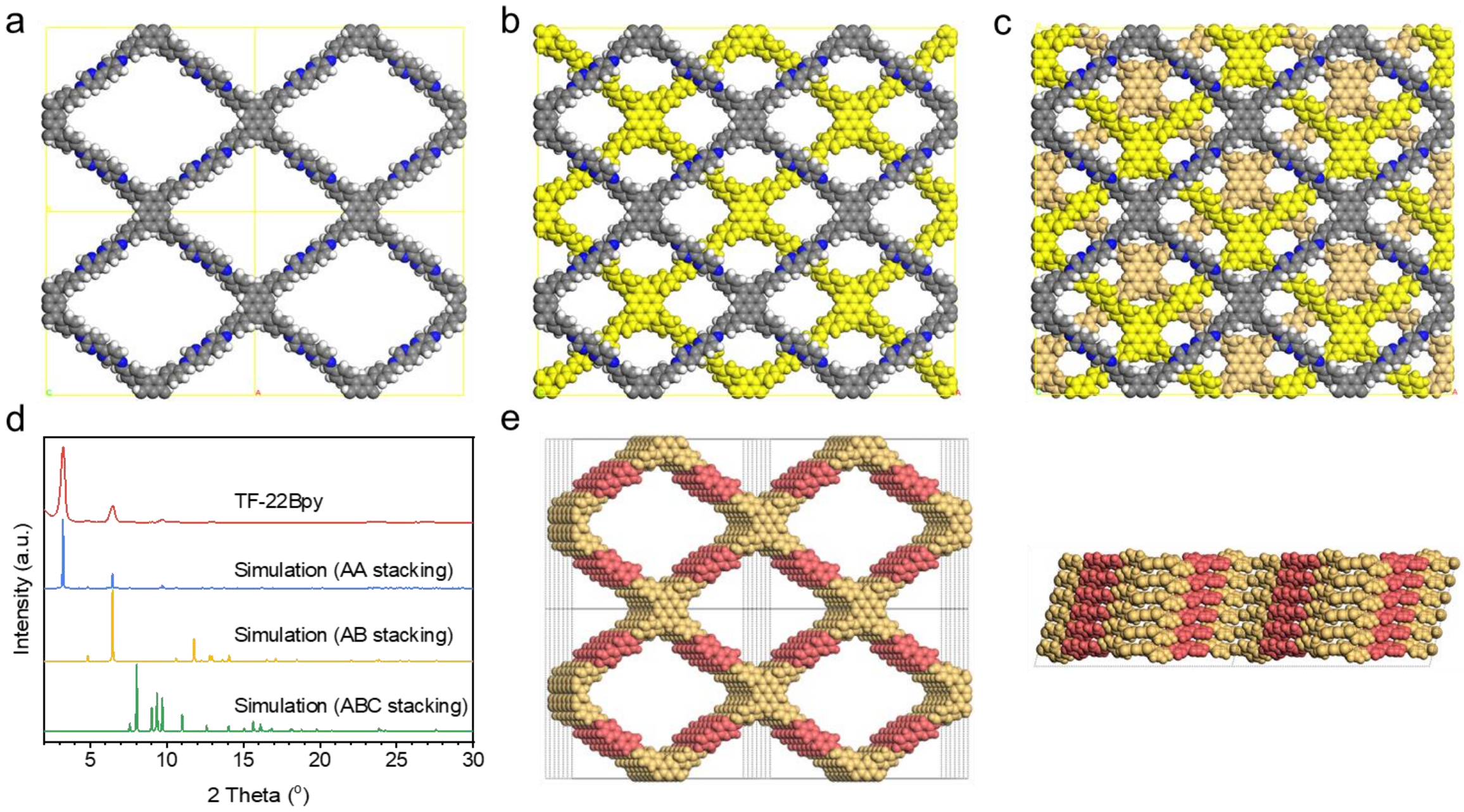

Supplementary Fig. 2 Theoretically modelled inclined (a) AA stacking, (b) AB stacking, (b) ABC stacking models, and (d) corresponding simulated XRD patterns of TF-22Bpy. (e) Top view (left) and side view (right) of the inclined AA stacking model.

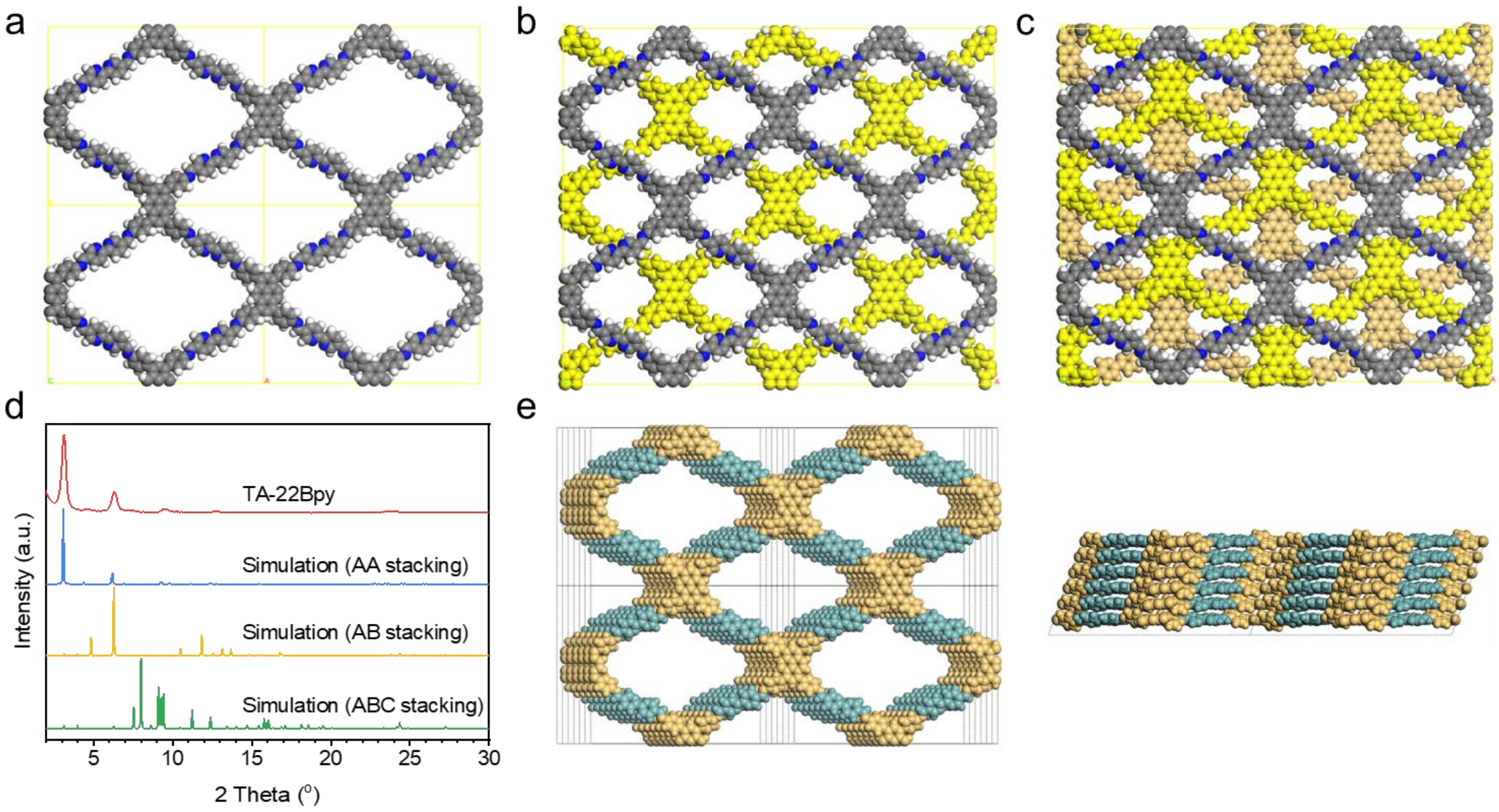

Supplementary Fig. 3 Theoretically modelled inclined (a) AA stacking, (b) AB stacking, (b) ABC stacking models, and (d) corresponding simulated XRD patterns of TA-22Bpy. (e) Top view (left) and side view (right) of the inclined AA stacking model.

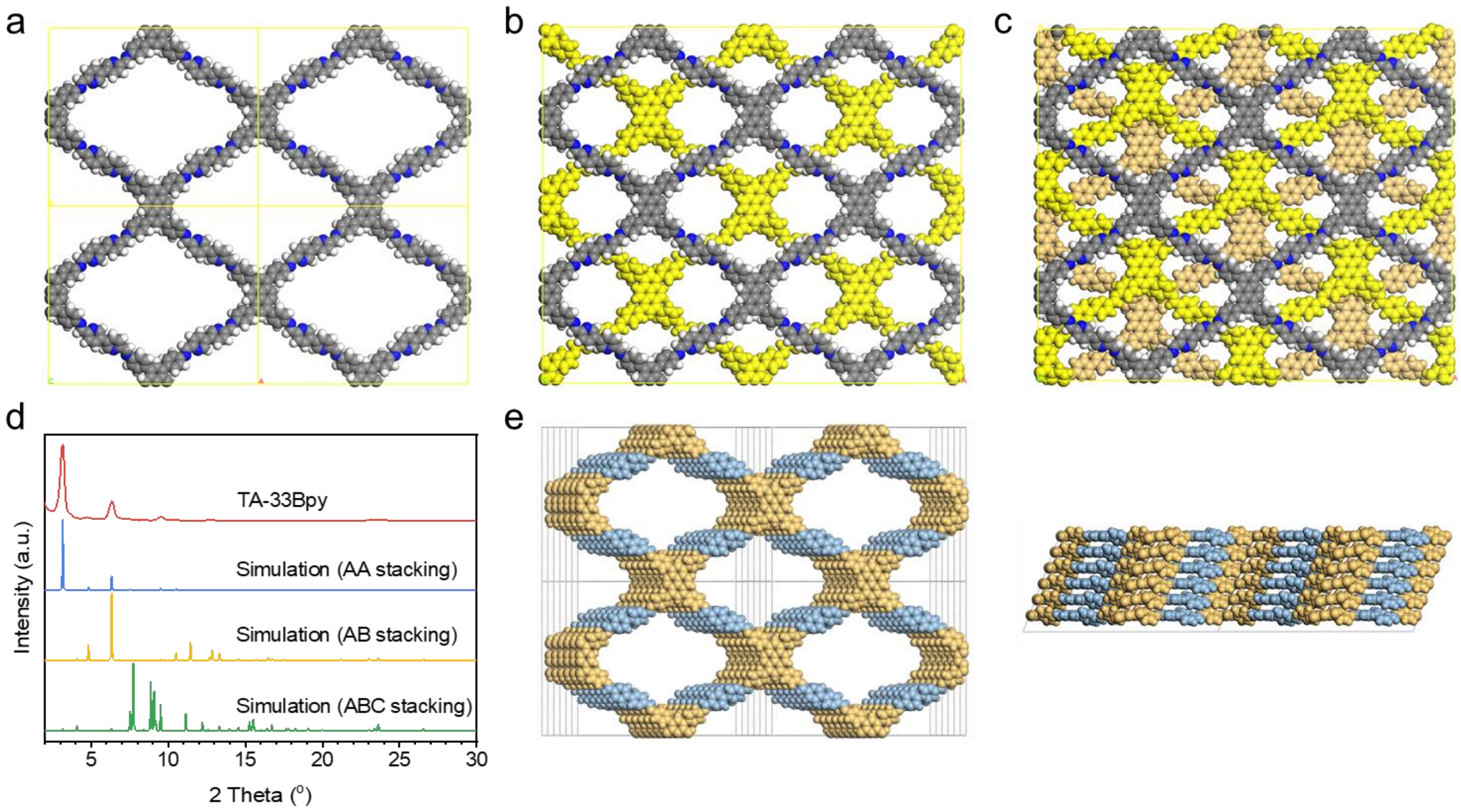

Supplementary Fig. 4 Theoretically modelled inclined (a) AA stacking, (b) AB stacking, (b) ABC stacking models, and (d) corresponding simulated XRD patterns of TA-33Bpy. (e) Top view (left) and side view (right) of the inclined AA stacking model.

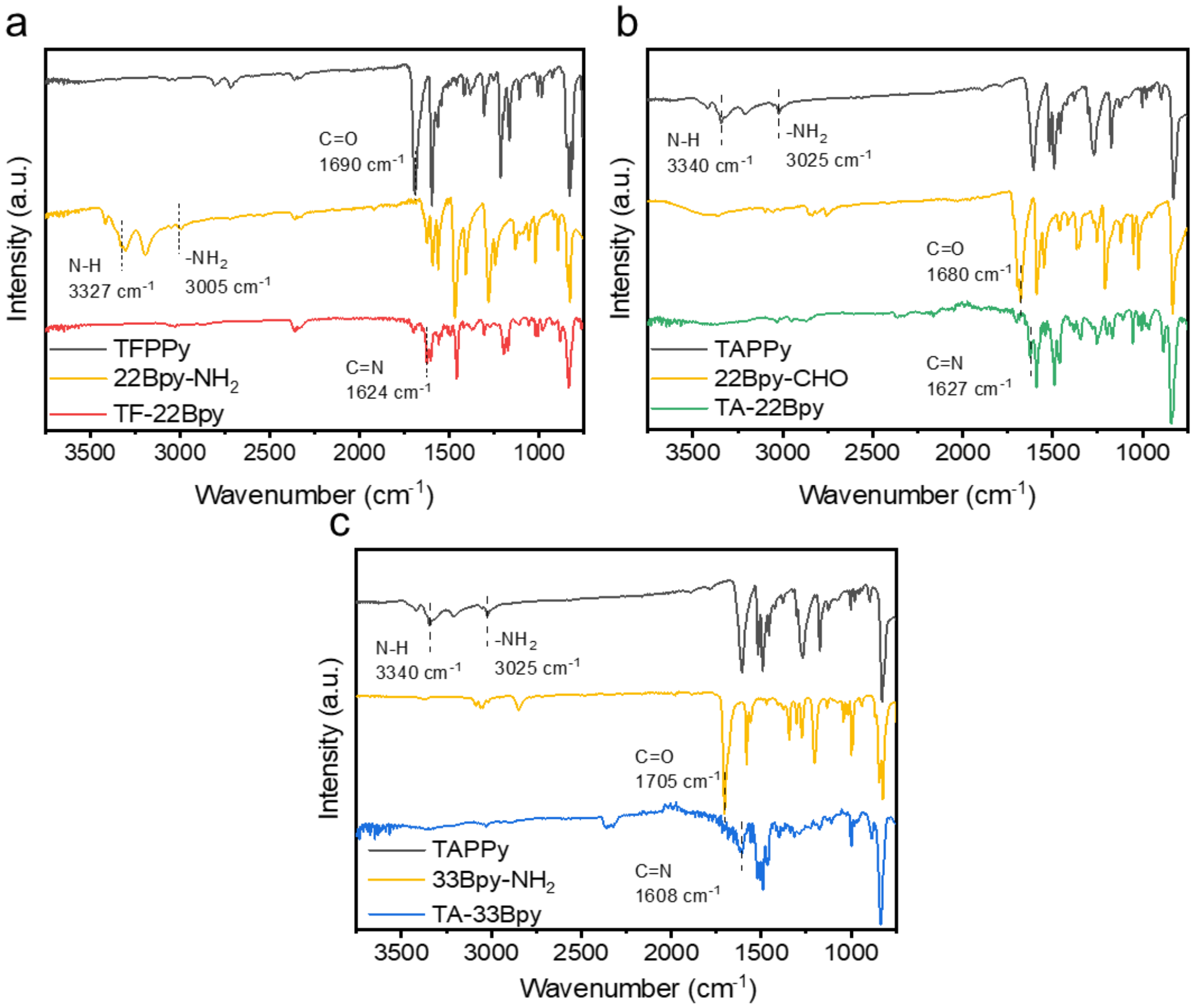

Supplementary Fig. 5 FT-IR spectra of (a) TF-22Bpy, (b) TA-22Bpy, (c) TA-33Bpy, and the corresponding monomers.

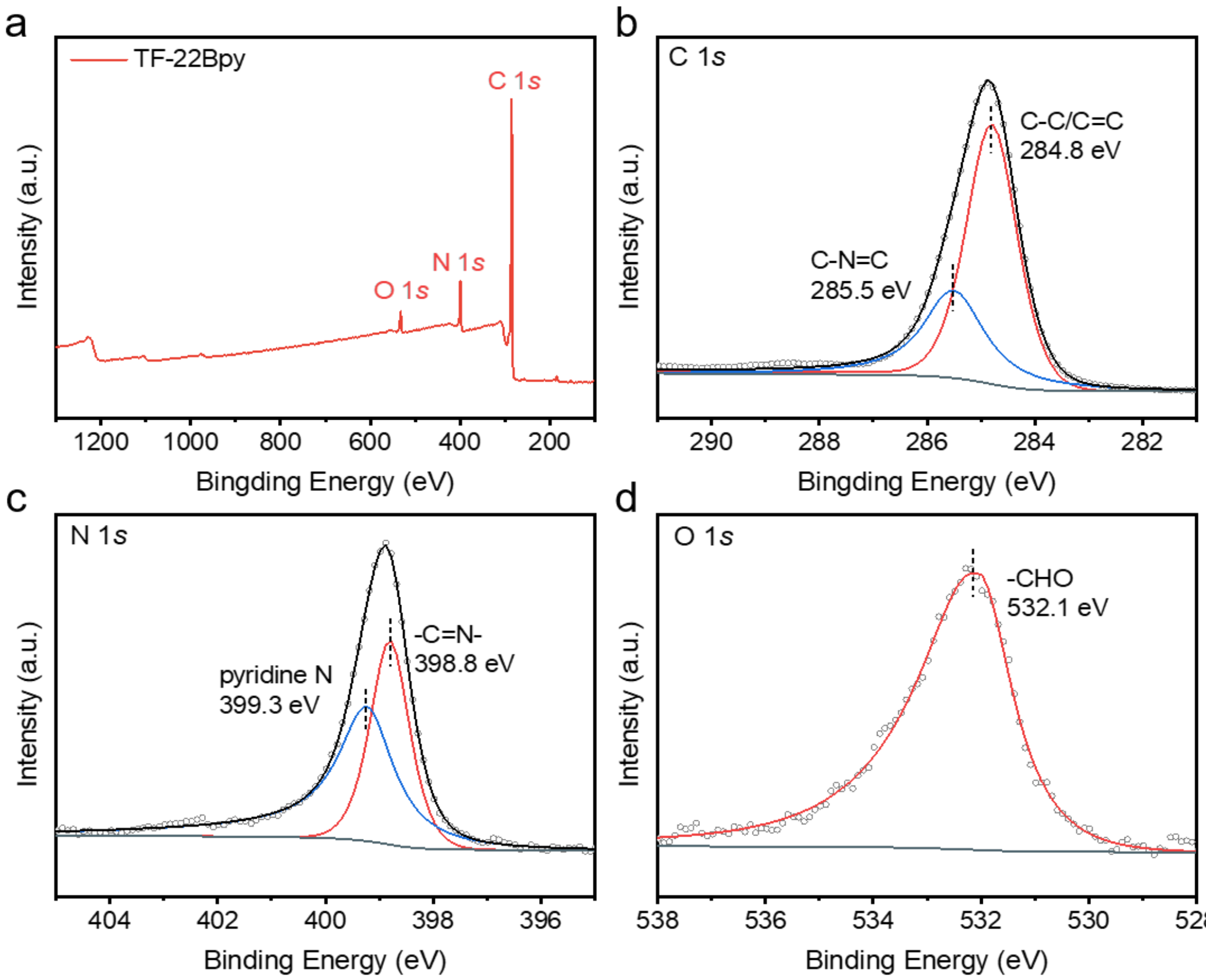

Supplementary Fig. 6 (a) XPS survey spectra, (b) high-resolution C 1*s* spectra, (c) N 1*s* spectra, (d) O 1*s* spectra of TF-22Bpy.

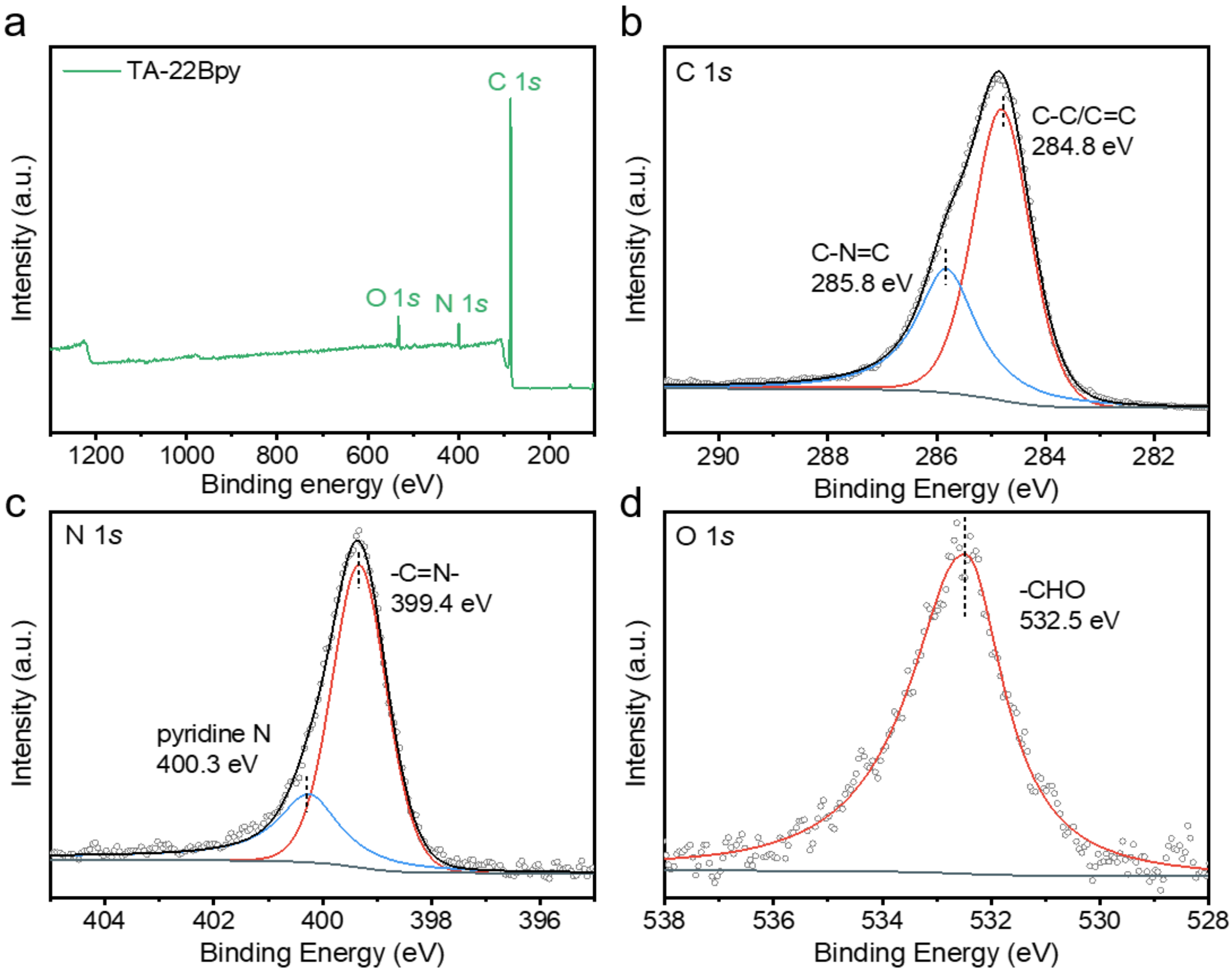

Supplementary Fig. 7 (a) XPS survey spectra, (b) high-resolution C 1*s* spectra, (c) N 1*s* spectra, (d) O 1*s* spectra of TA-22Bpy.

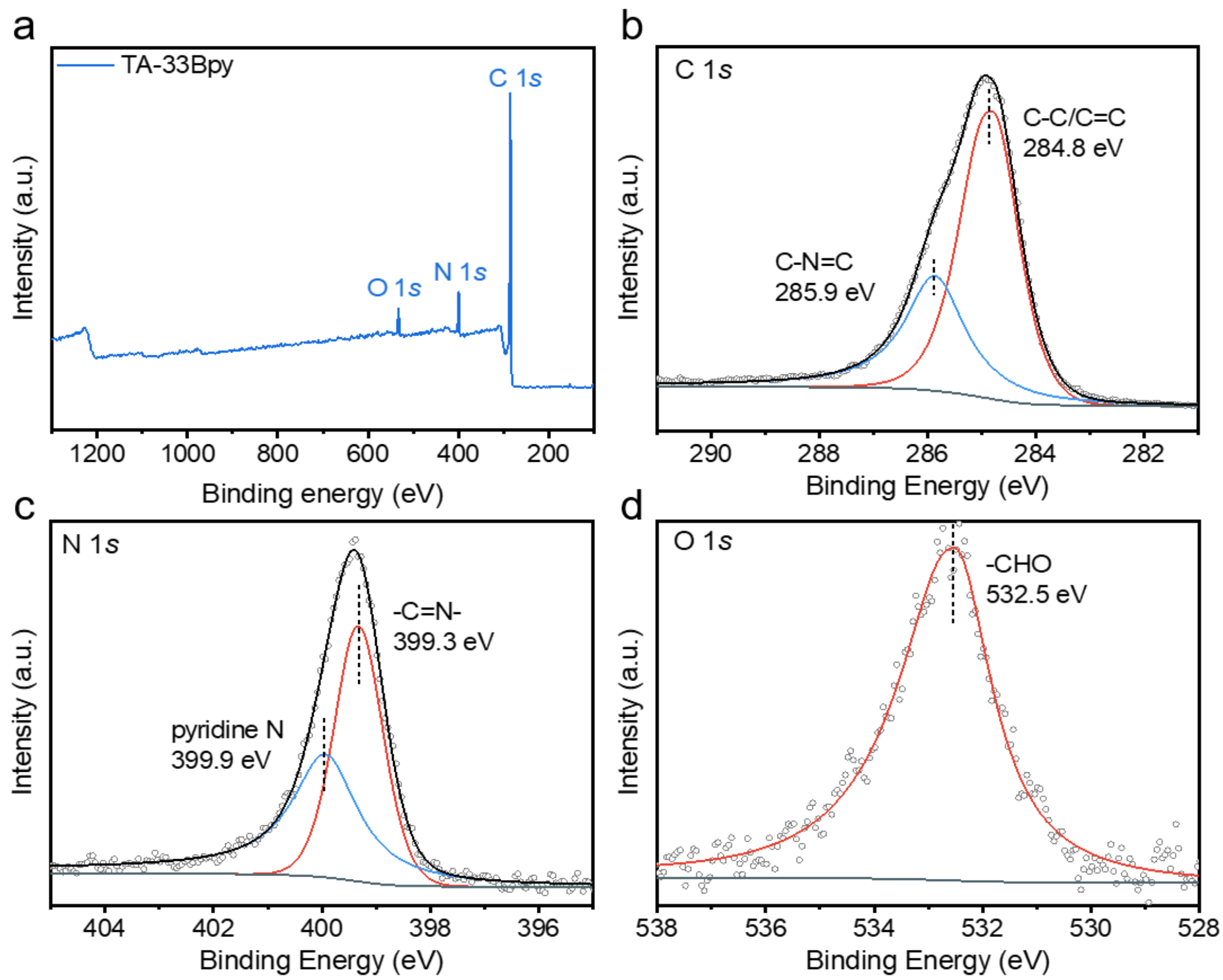

Supplementary Fig. 8 (a) XPS survey spectra, (b) high-resolution C 1*s* spectra, (c) N 1*s* spectra, (d) O 1*s* spectra of TA-33Bpy.

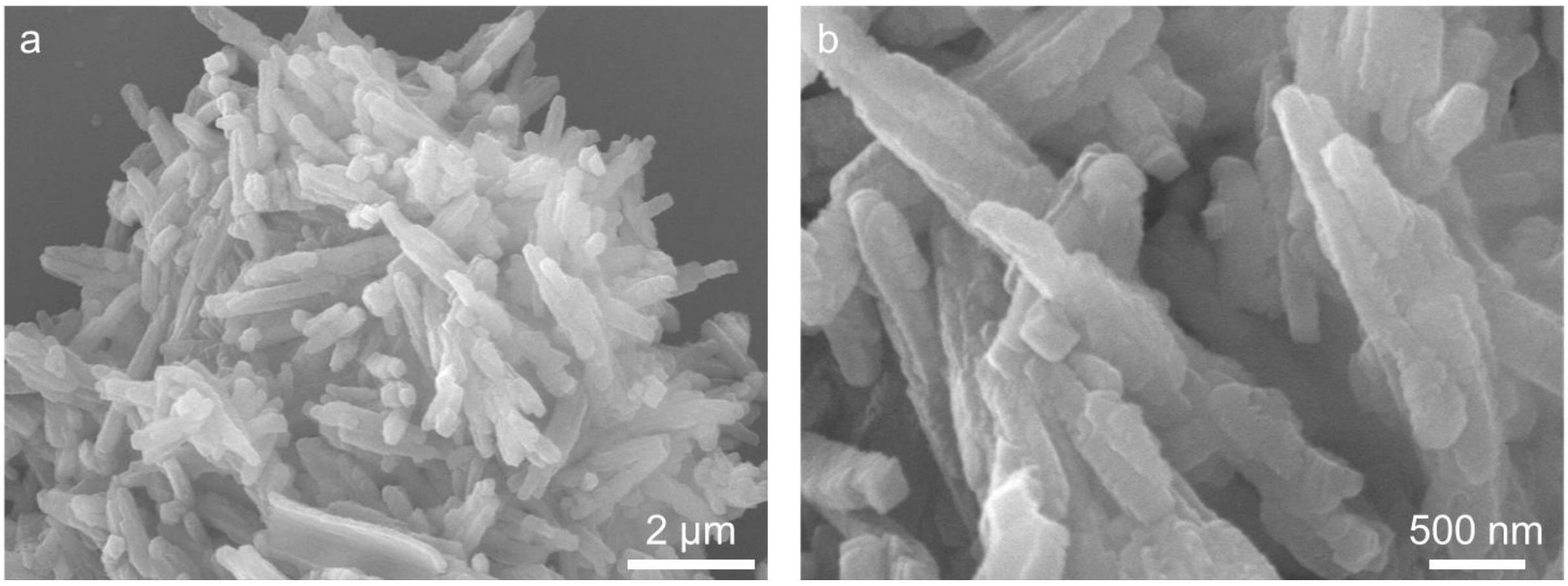

Supplementary Fig. 9 (a) SEM and (b) enlarged SEM images of TF-22Bpy.

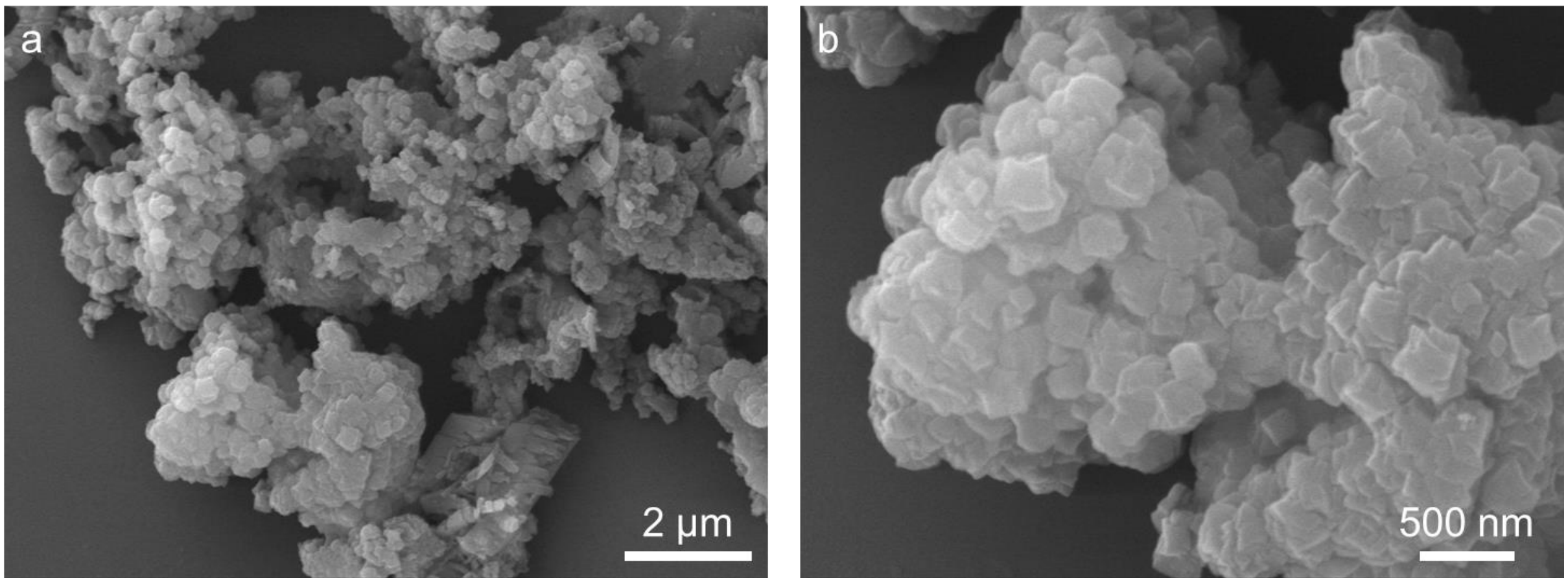

Supplementary Fig. 10 (a) SEM and (b) enlarged SEM images of TA-22Bpy.

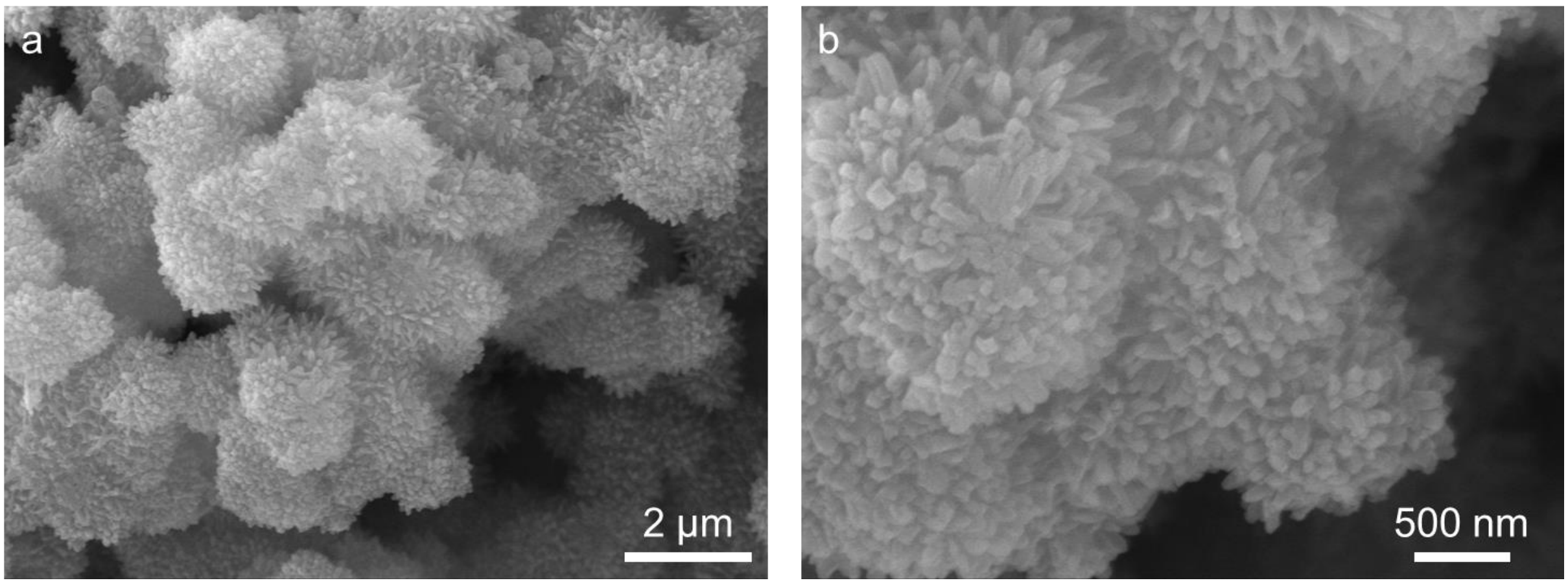

Supplementary Fig. 11 (a) SEM and (b) enlarged SEM images of TA-33Bpy.

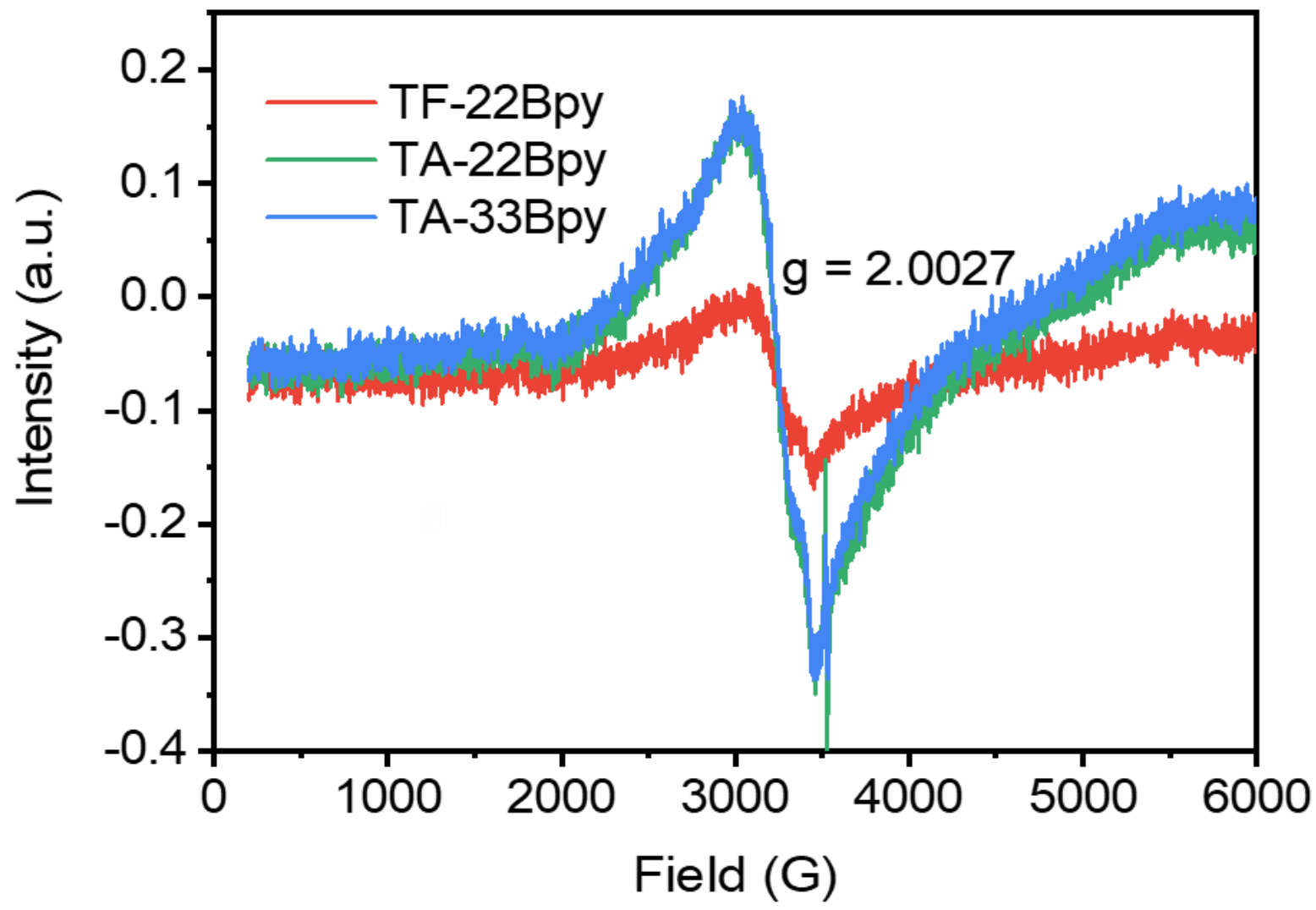

Supplementary Fig. 12 Solid-state EPR spectra of the isomeric COFs.

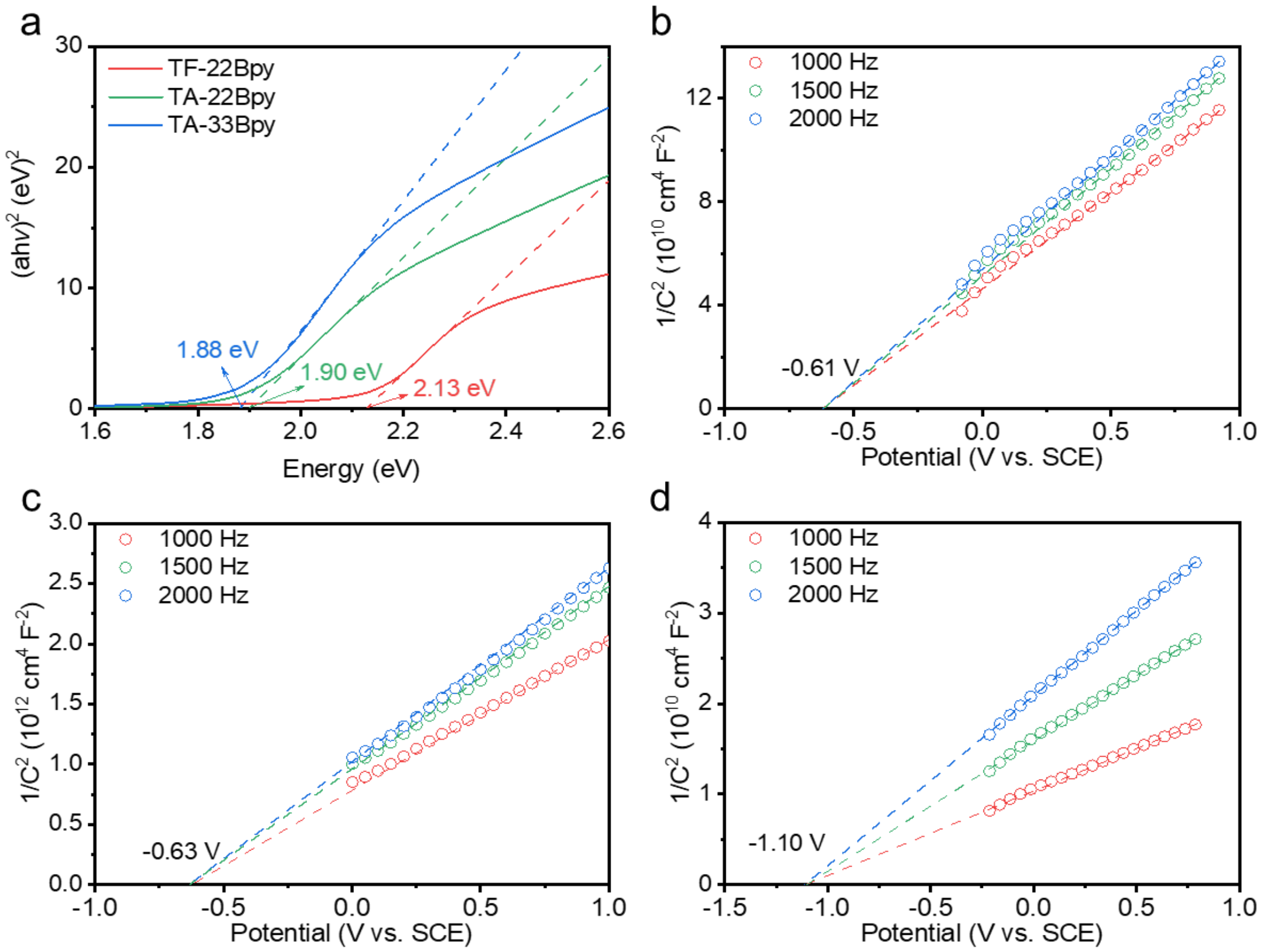

Supplementary Fig. 13 (a) Tauc plots of isomeric COFs. Mott−Schottky plots of (b) TF-22Bpy, (c) TA-22Bpy and (d) TA-33Bpy COF.

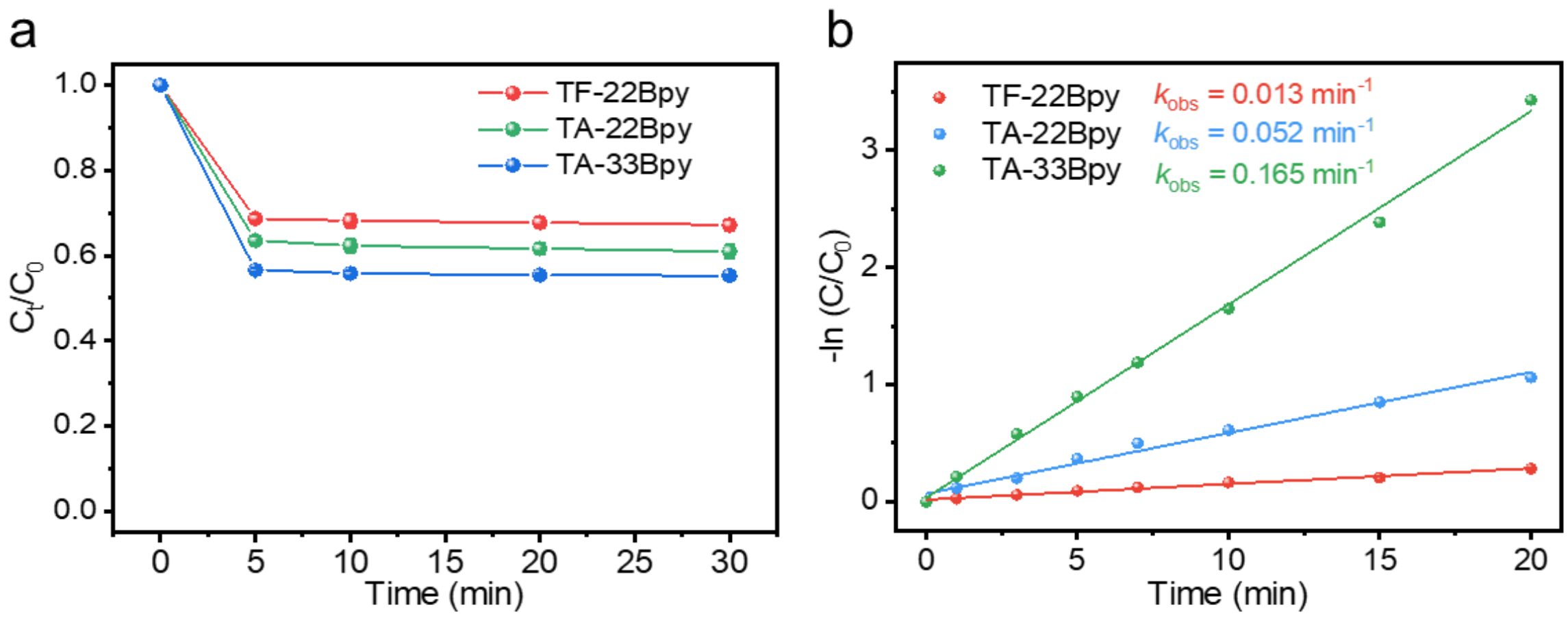

Supplementary Fig. 14 (a) BPA adsorption capacity of the isomeric COFs. (b) Kinetics of BPA degradation in different COFs/PMS systems.

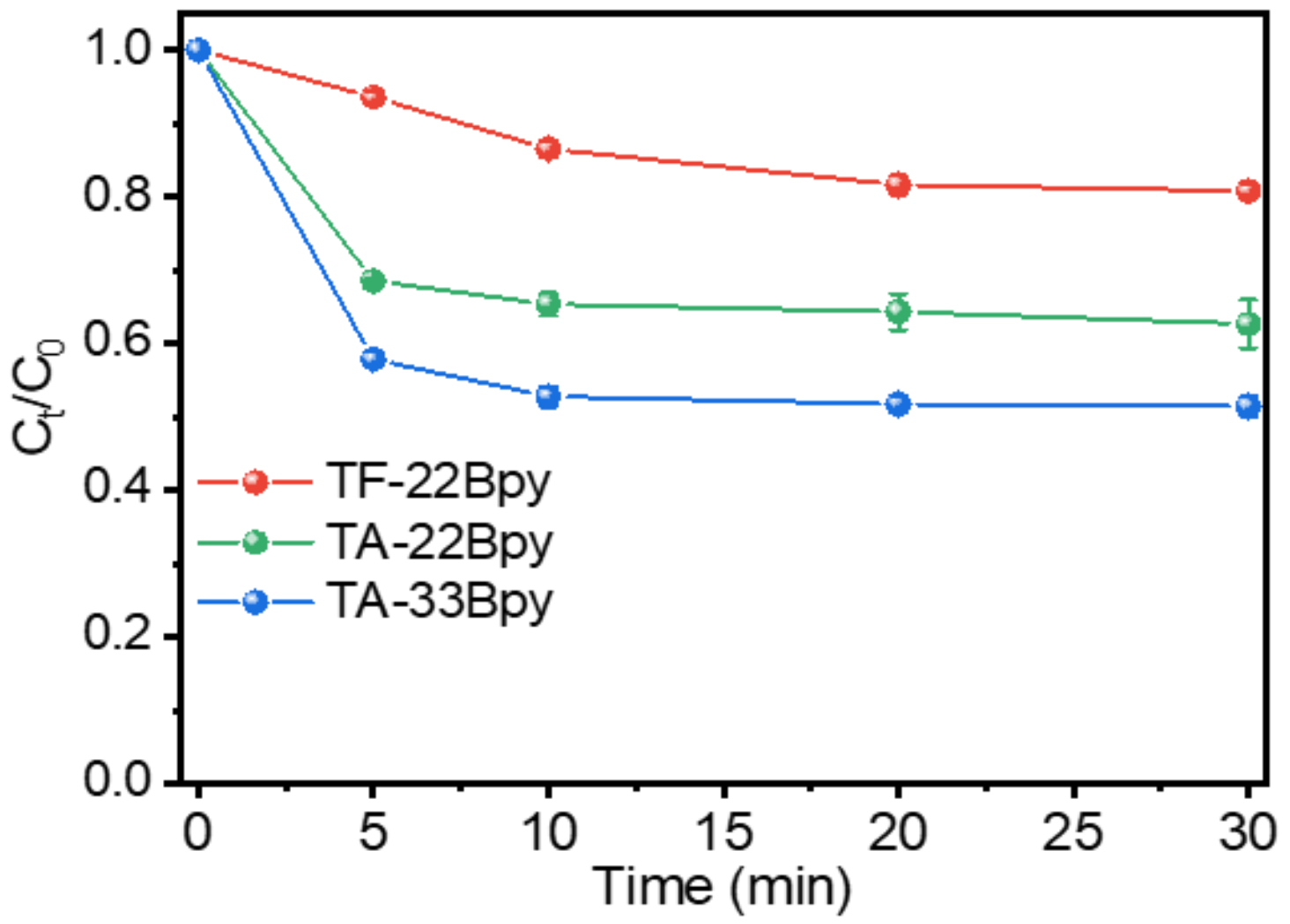

Supplementary Fig. 15 TOC removal in different COFs/PMS systems.

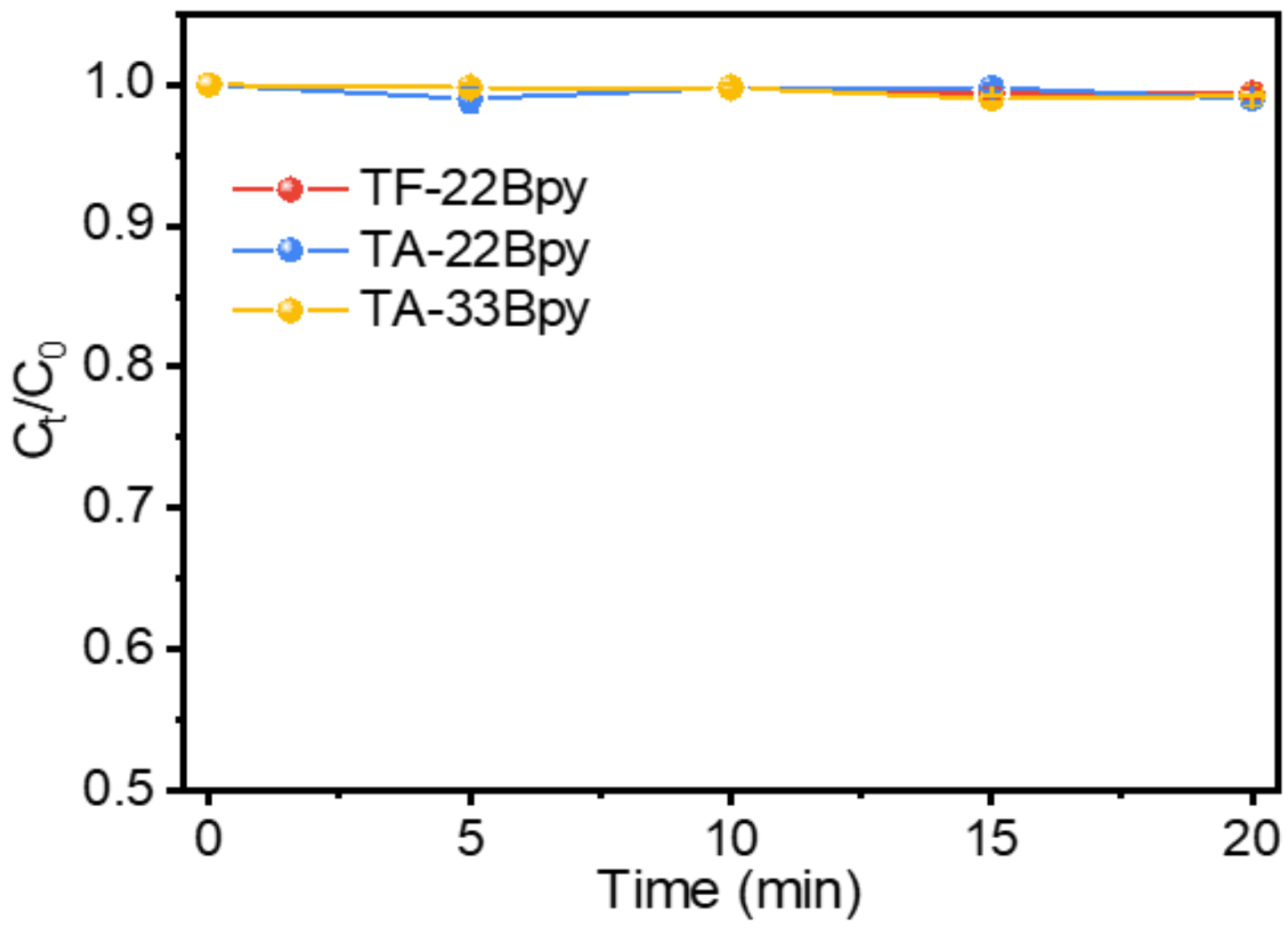

Supplementary Fig. 16 BA removal in different COFs/PMS systems.

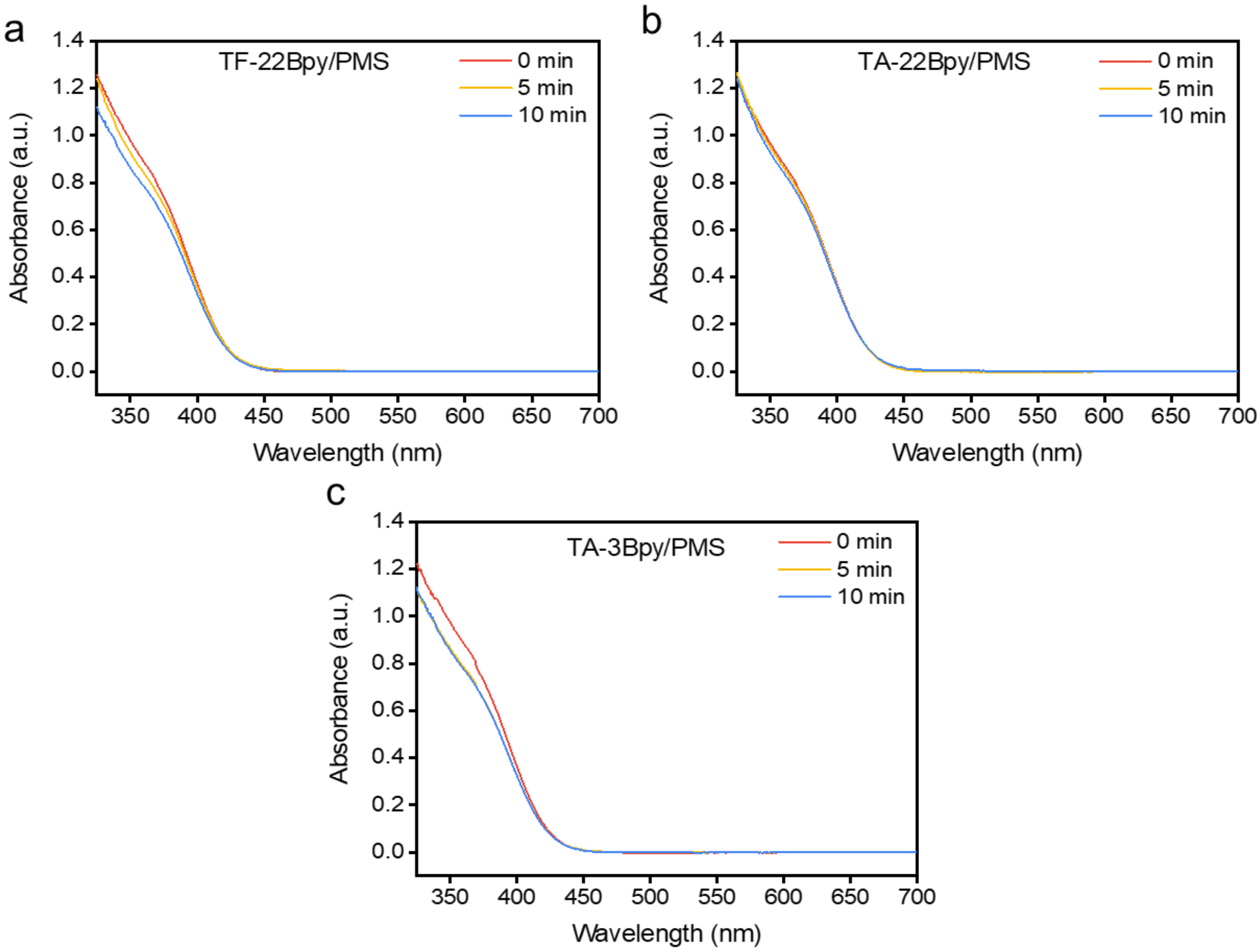

Supplementary Fig. 17 Determination of $O_2^{\bullet-}$ in (a) TF-22Bpy/PMS, (b) TA-22Bpy/PMS, and (c) TA-33Bpy/PMS systems through NBT probing.

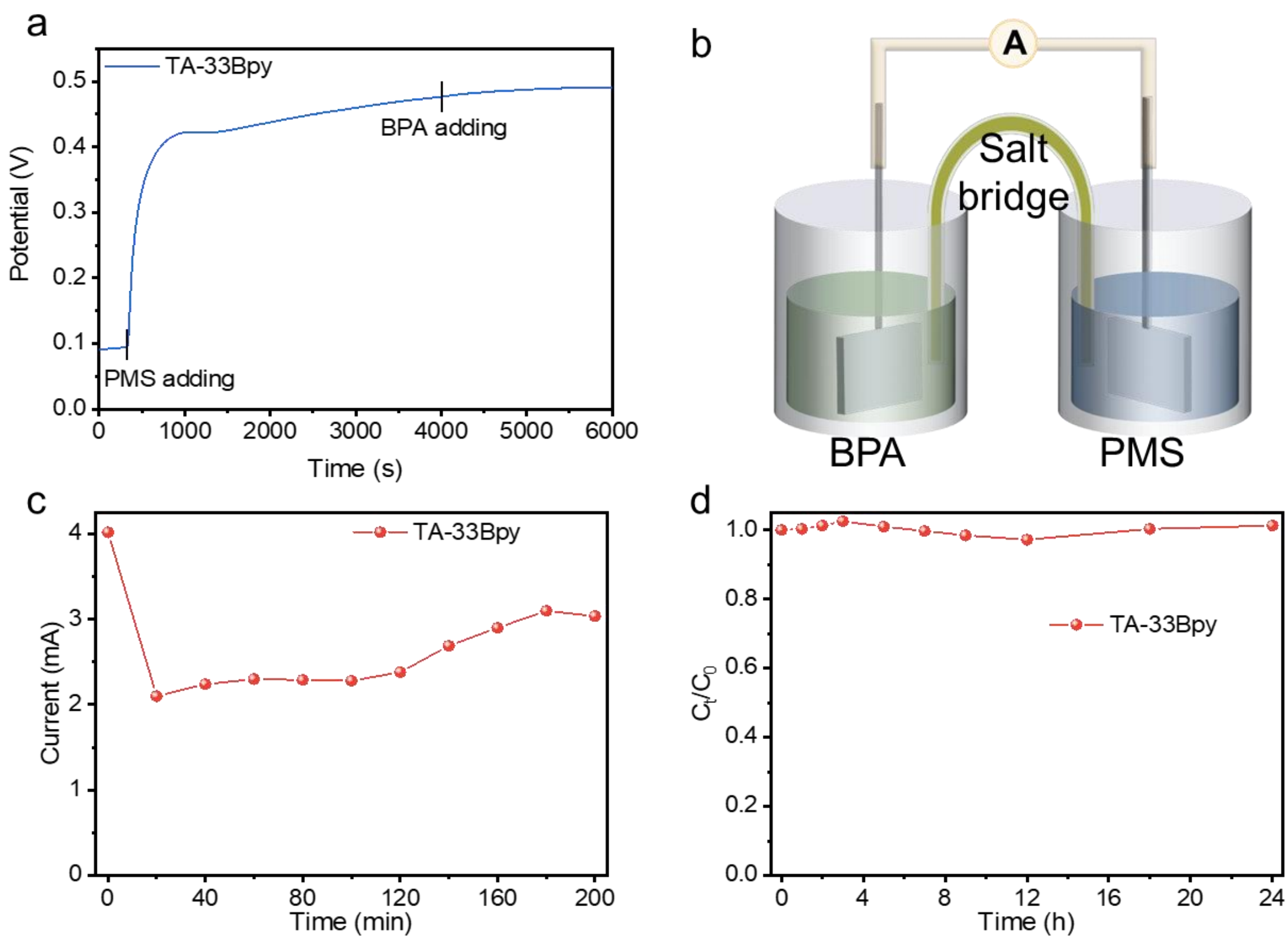

Supplementary Fig. 18 (a) The time-profile of open circuit potential upon addition of PMS and BPA. (b) Schematic diagram of the salt bridge device. (c) Current changes and (d) BPA removal in the GOP.

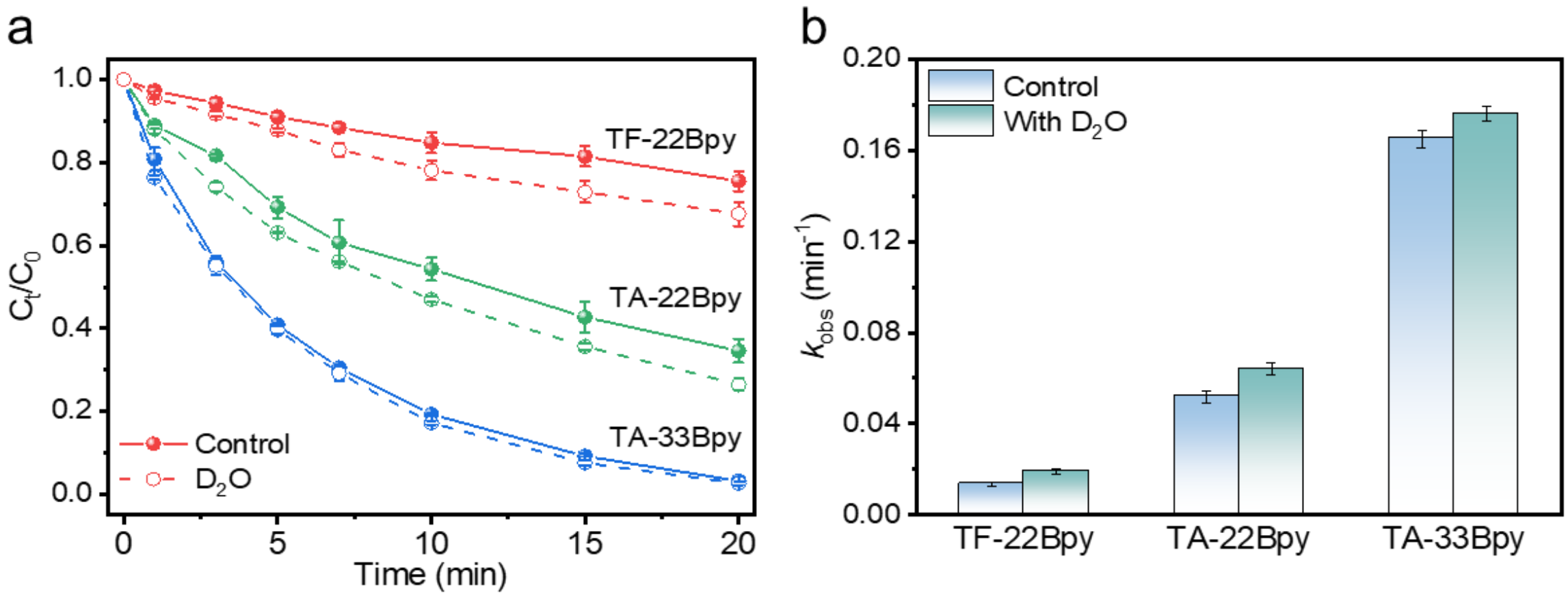

Supplementary Fig. 19 (a) BPA removal with the presence of 10% D_2O (v/v) and (b) corresponding k_{obs} in COFs/PMS systems. Data for (a-b) are presented as mean values ± SD (n = 3).

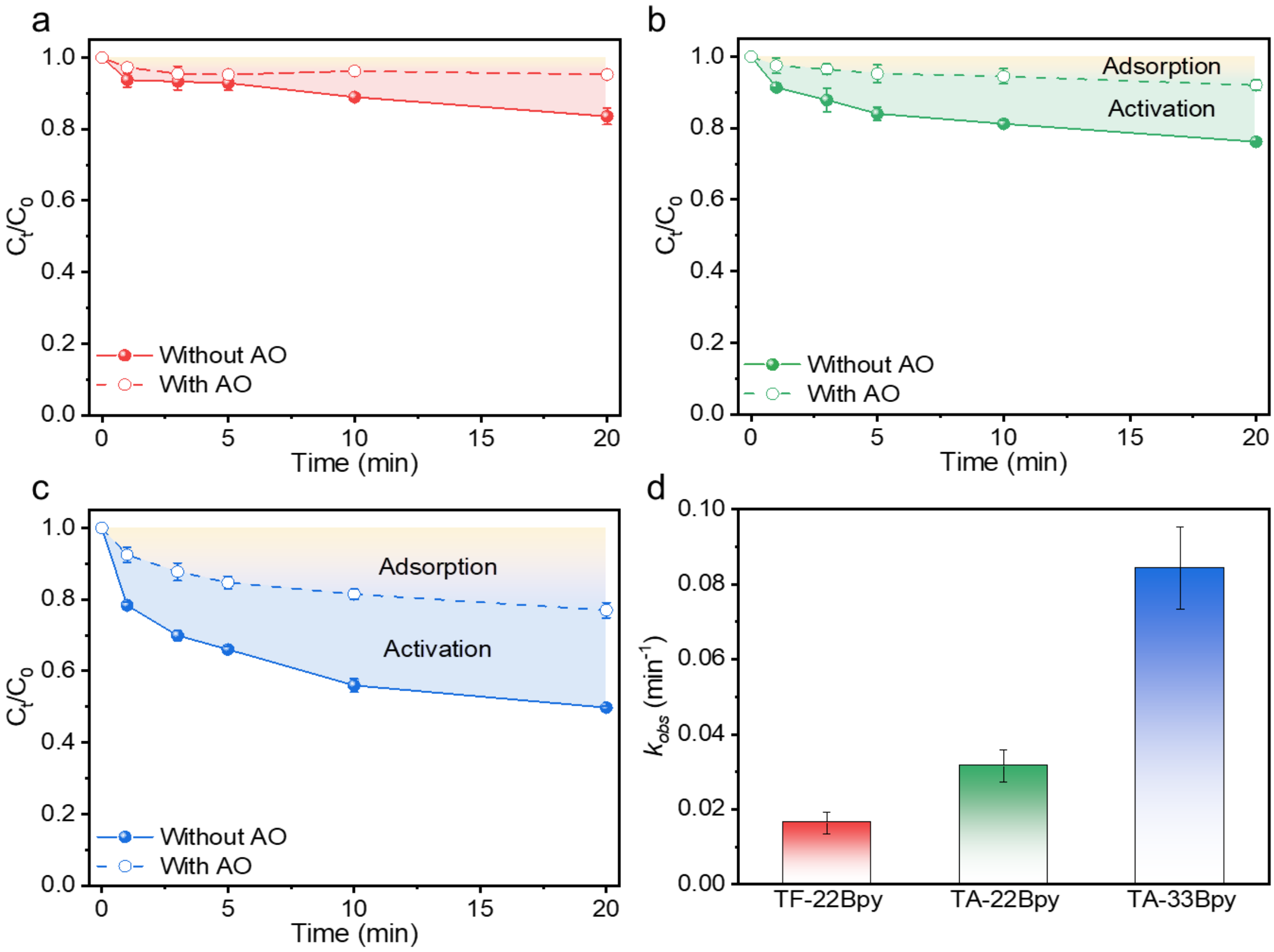

Supplementary Fig. 20 The consumption and adsorption of PMS by (a) TF-22Bpy, (b) TA-22Bpy and (c) TA-33Bpy. (d) PMS consumption kinetics. Data for (a-d) are presented as mean values ± SD (n = 3).

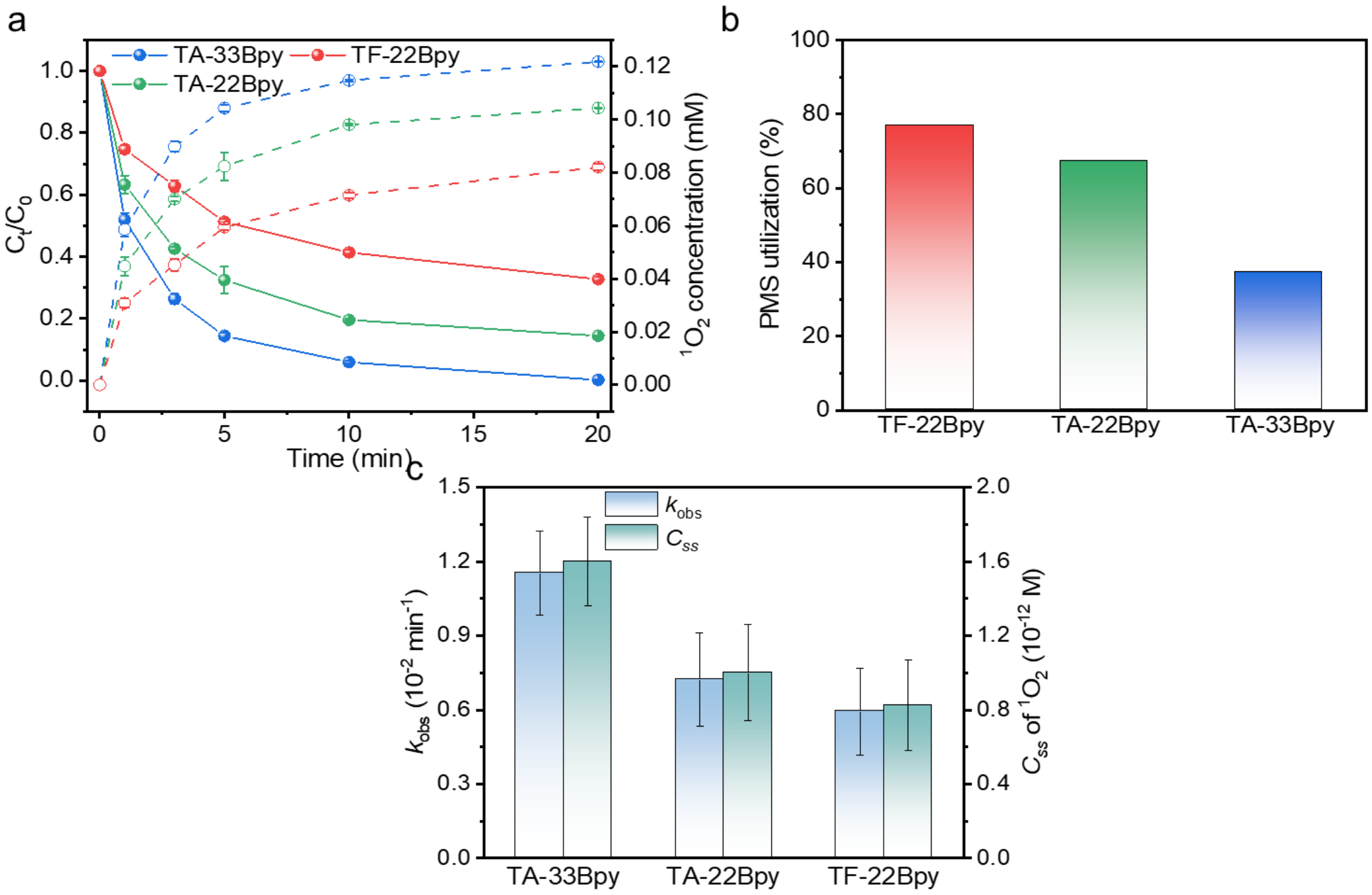

Supplementary Fig. 21 (a) The consumption of ABDA and generation of $^1O_2$ in COFs/PMS system. (b) Efficiency of PMS to $^1O_2$. (c) The k_{obs} of FFA probes and steady-state concentration of $^1O_2$. Data for (a and c) are presented as mean values ± SD (n = 3).

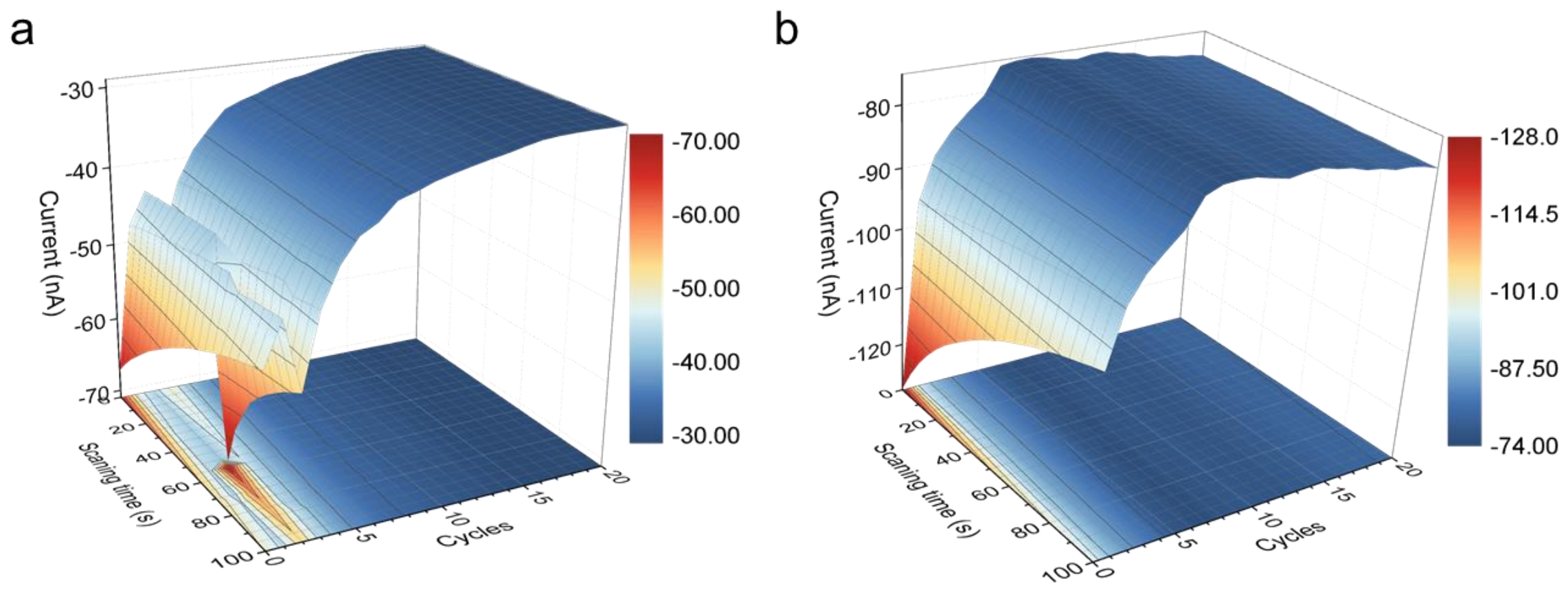

Supplementary Fig. 22 Scanning electrochemical microscopic analysis of (a) TF-22Bpy/PMS, (b) TA-22Bpy/PMS

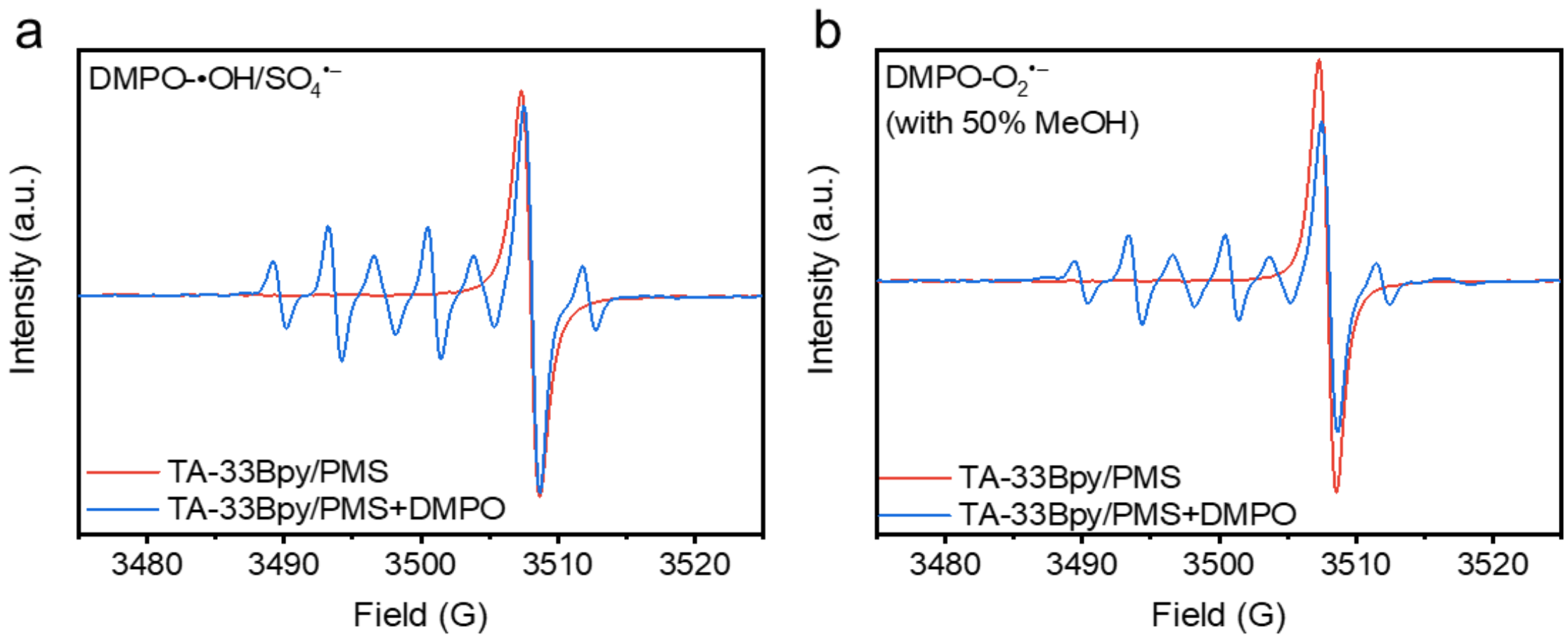

Supplementary Fig. 23 EPR spectra of TA-33Bpy/PMS system using DMPO as trapping agents for (a) $\bullet OH/SO_4^{\bullet-}$, and (b) $O_2^{\bullet-}$.

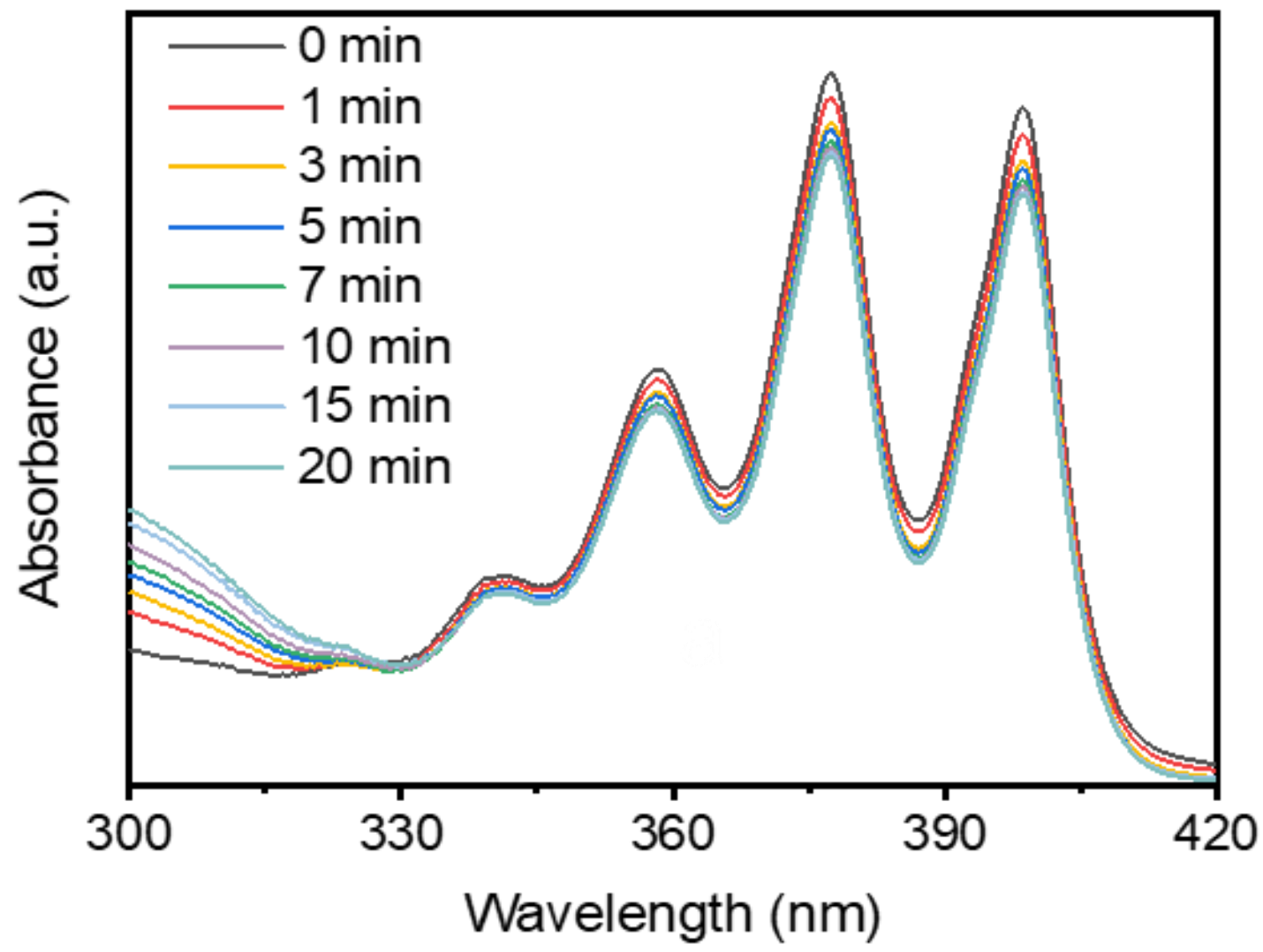

Supplementary Fig. 24 UV-vis absorption spectra of DMA in acetonitrile/H2O (v/v = 50:50) upon treatment with TA-33Bpy/PMS system.

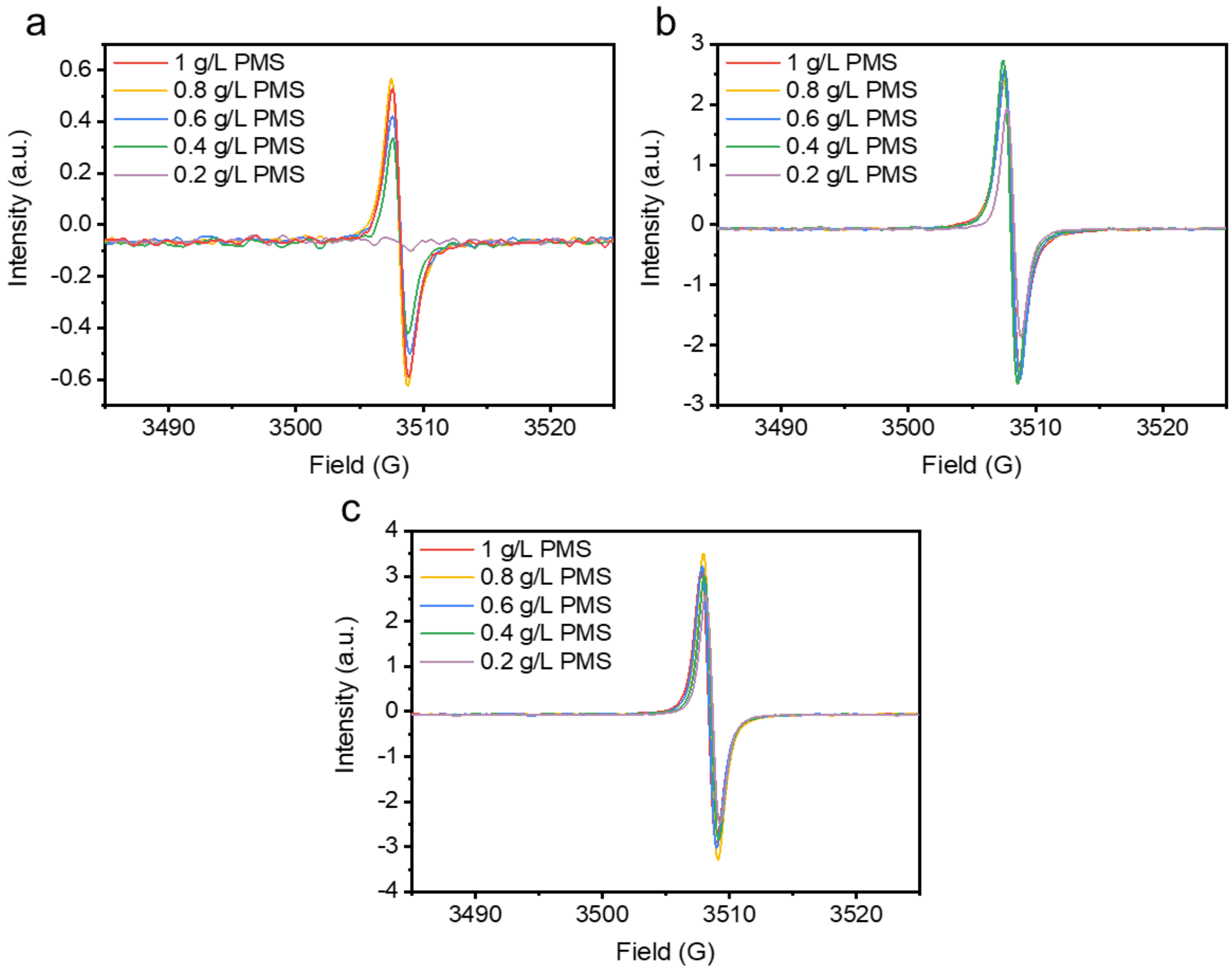

Supplementary Fig. 25 EPR spectra of (a) TF-22Bpy/PMS, (b) TA-22Bpy/PMS, and (c) TA-33Bpy/PMS systems at different PMS concentrations. (C_{COFs} = 0.1 g/L)

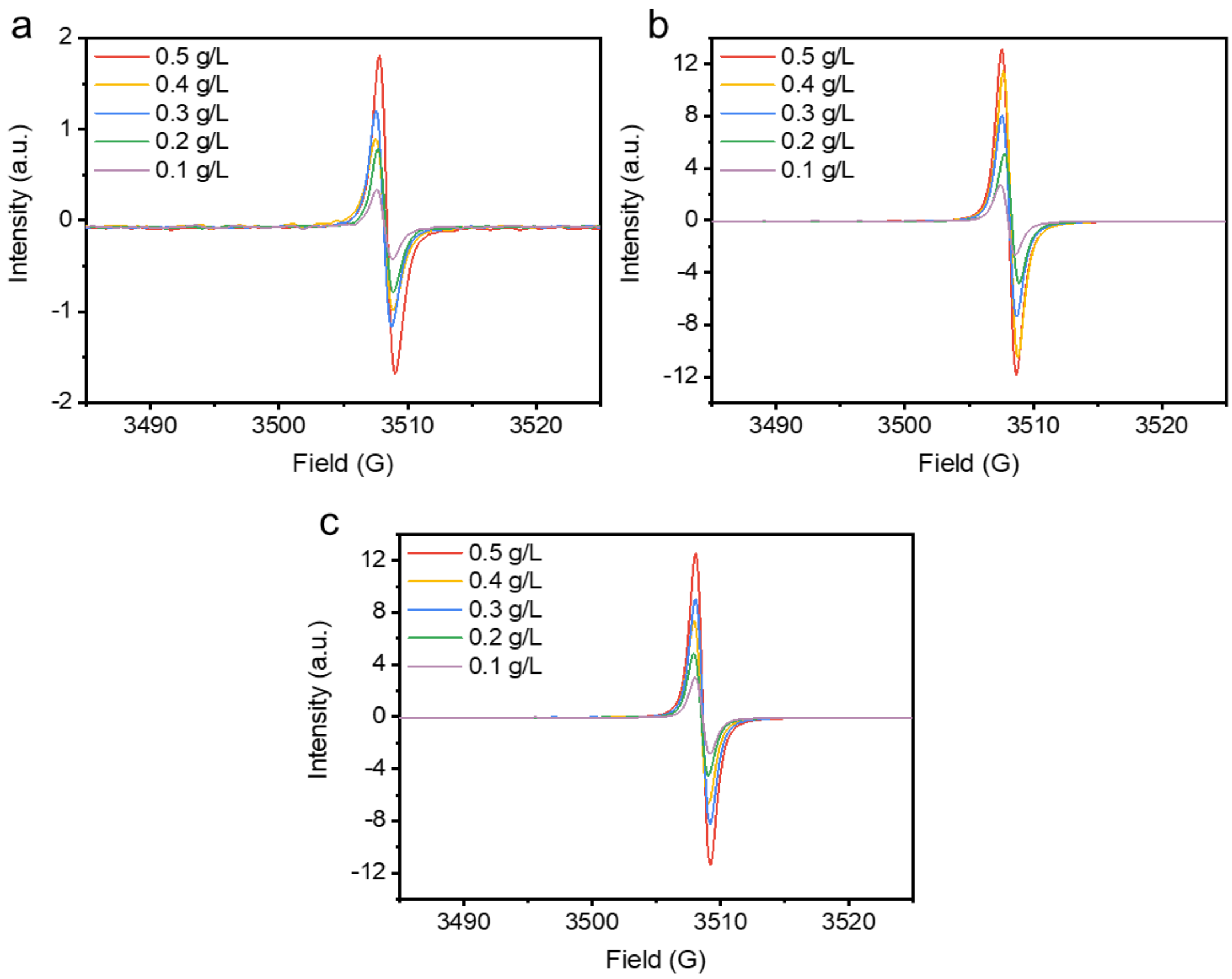

Supplementary Fig. 26 EPR spectra of (a) TF-22Bpy/PMS, (b) TA-22Bpy/PMS, and (c) TA-33Bpy/PMS systems at different PMS concentrations. (C_{PMS} = 0.4 g/L)

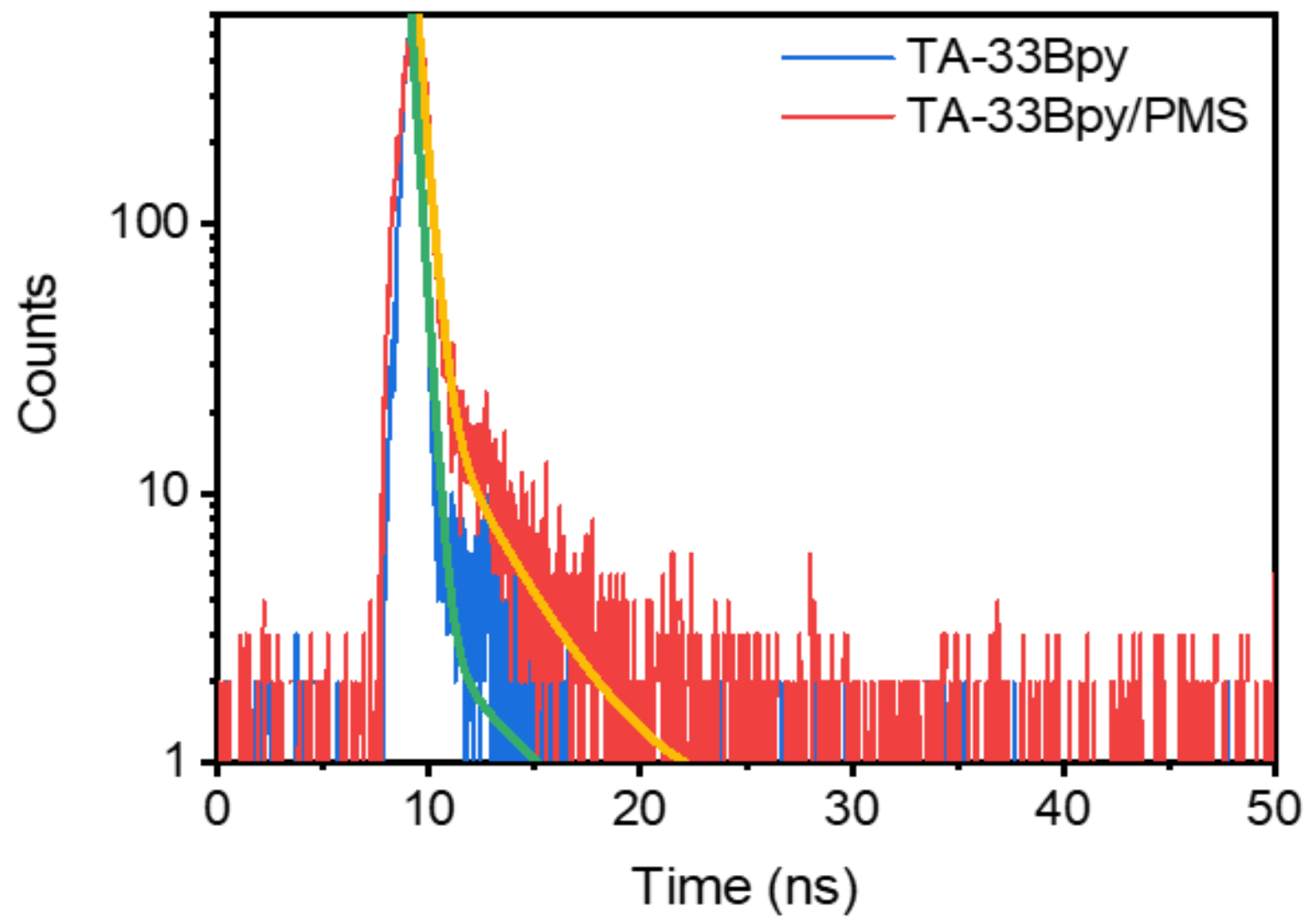

Supplementary Fig. 27 Time-resolved photoluminescence decay plots of TA-33Bpy and TA-33Bpy/PMS.

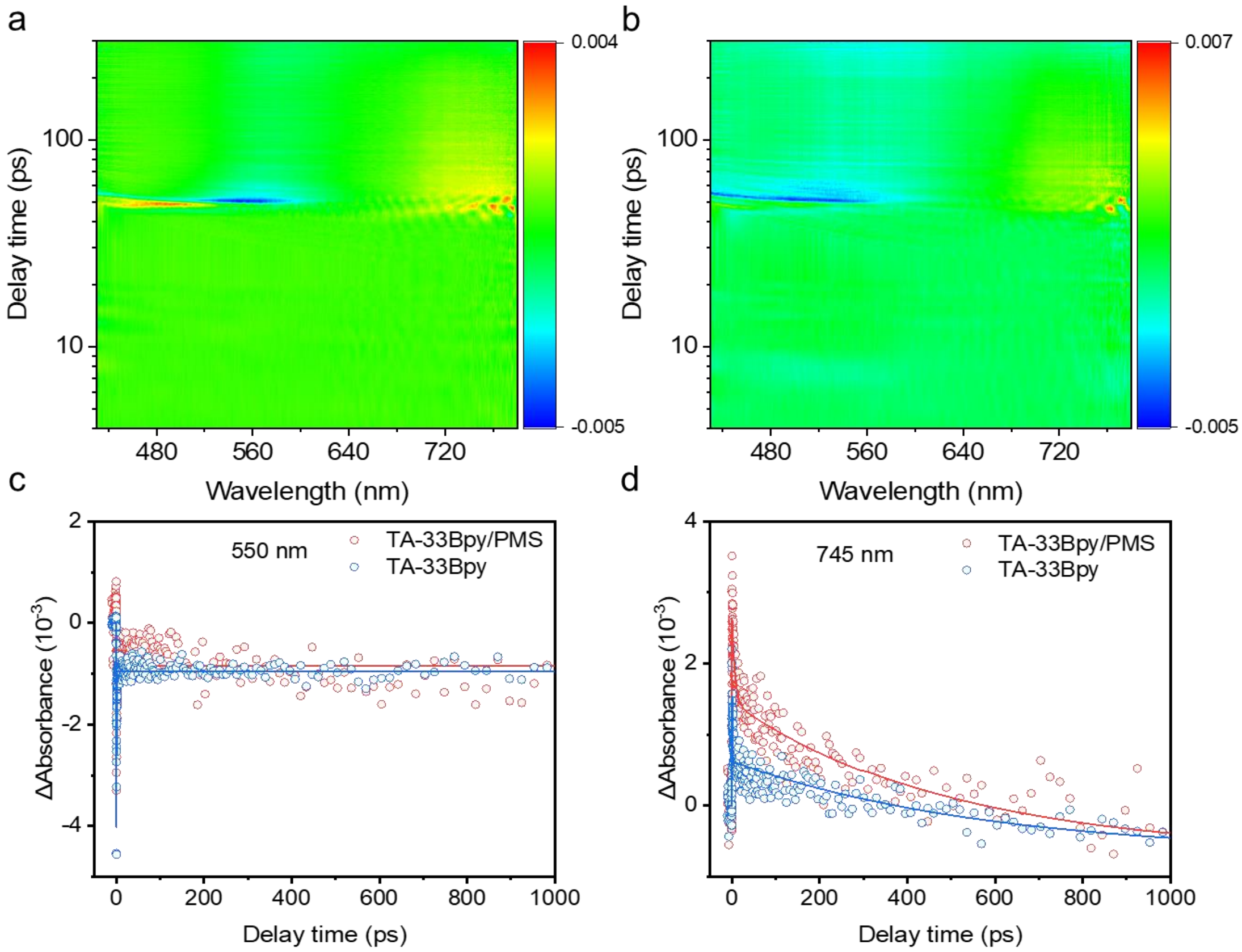

Supplementary Fig. 28 2D mapping TA spectra of (a) TA-33Bpy and (b) TA-33Bpy/PMS. Decay kinetic curves on (c) 550 nm and (d) 745 nm.

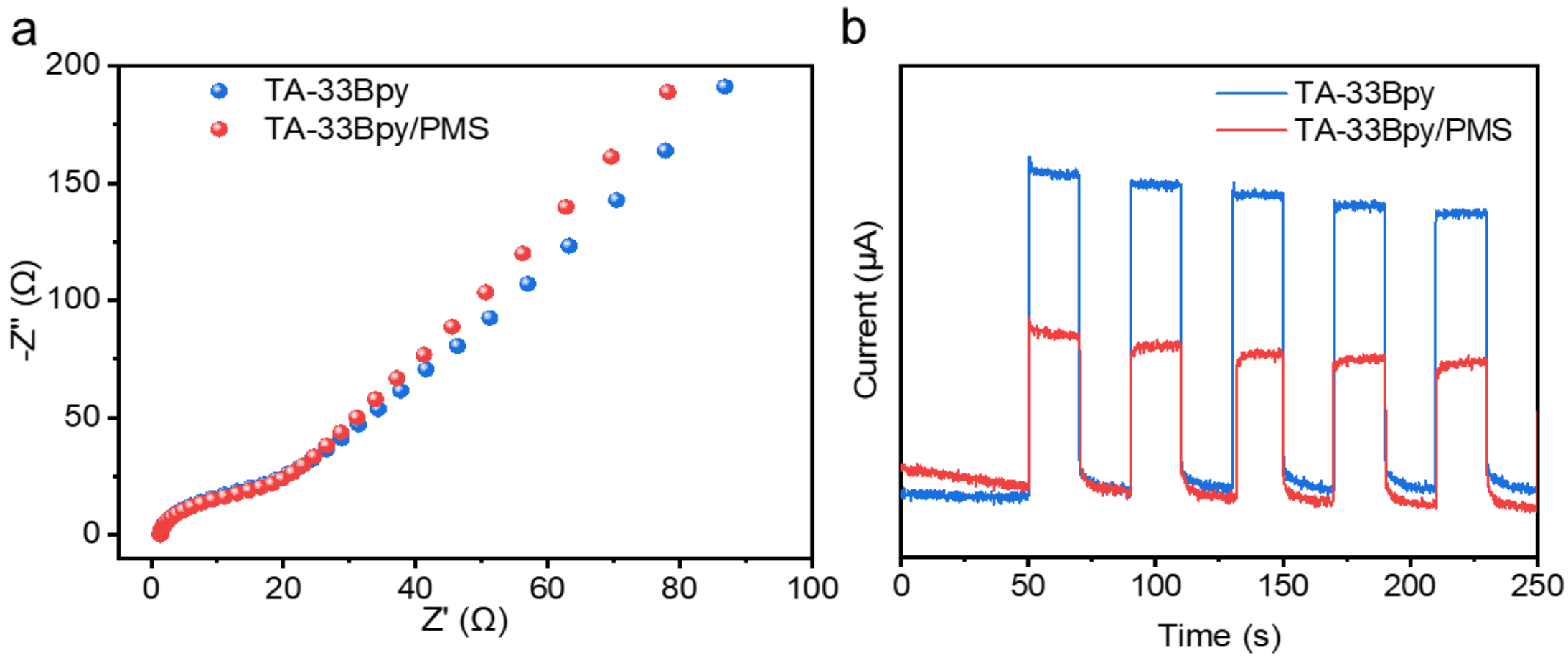

Supplementary Fig. 29 (a) Electrochemical impedance spectra and (b) transient photocurrent responses of TA-33Bpy and TA-33Bpy/PMS.

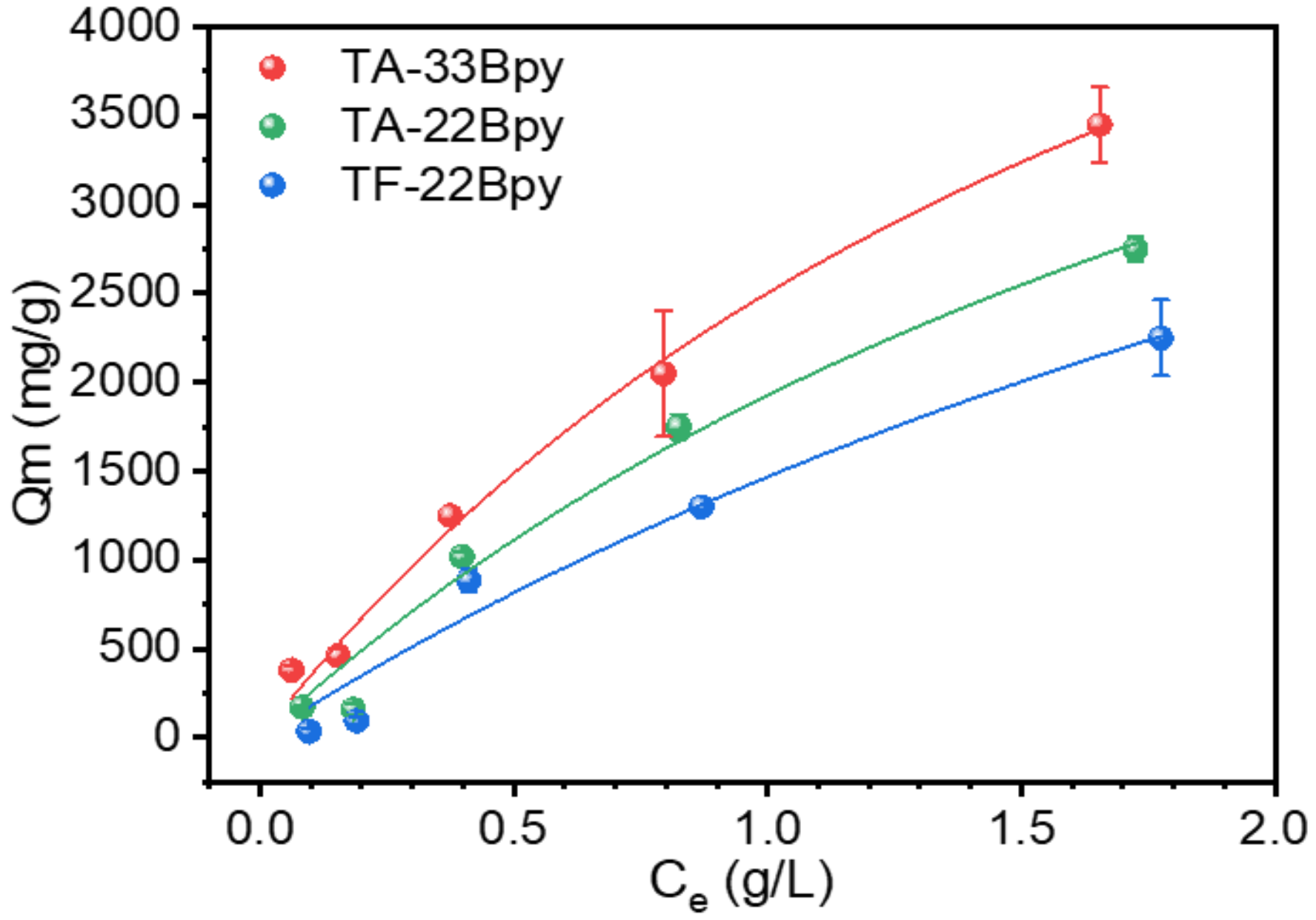

Supplementary Fig. 30 Isothermal adsorption curves of PMS by COFs. Data for this figure is presented as mean values ± SD (n = 3).

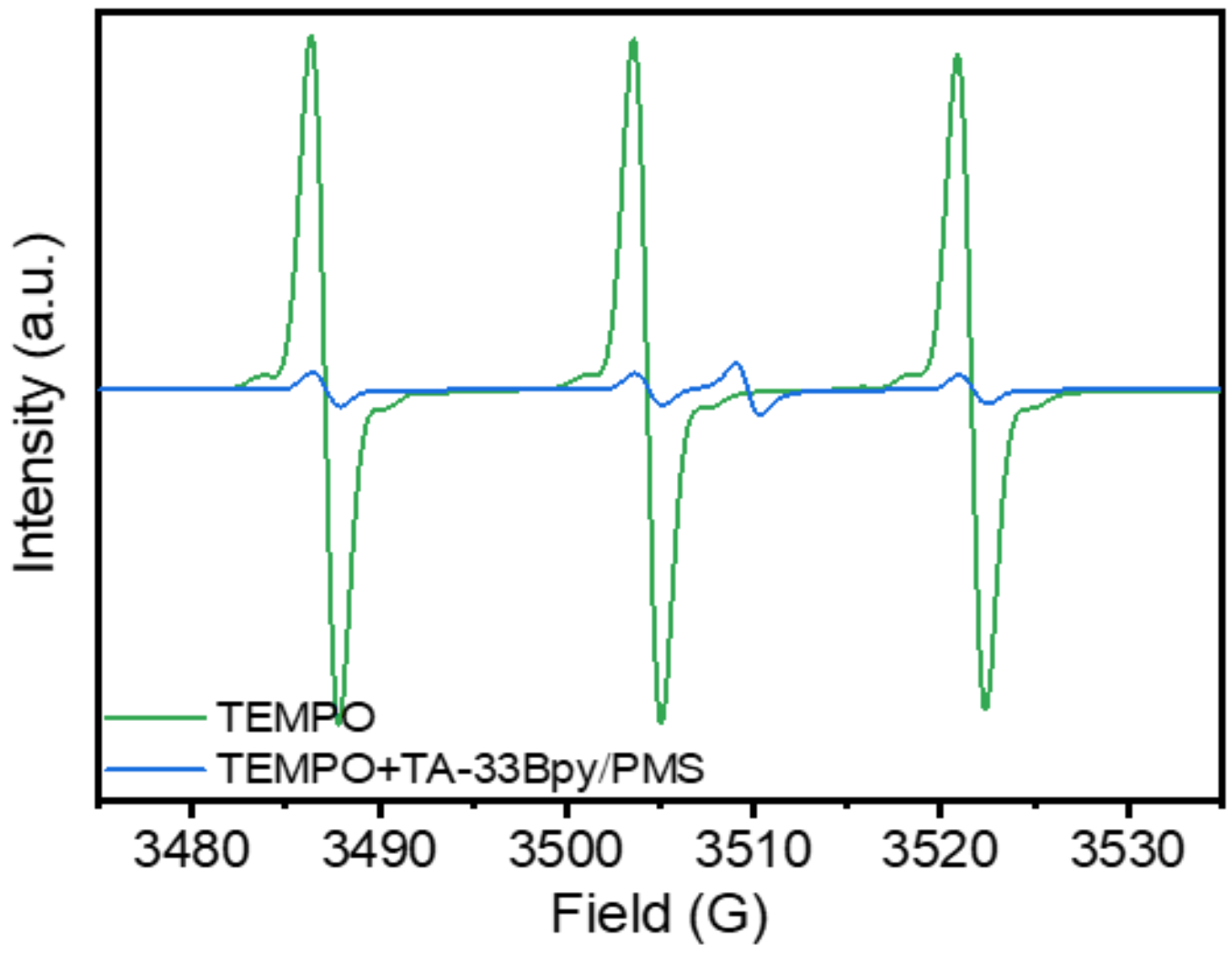

Supplementary Fig. 31 EPR spectra of TEMPO in TA-33Bpy/PMS systems.

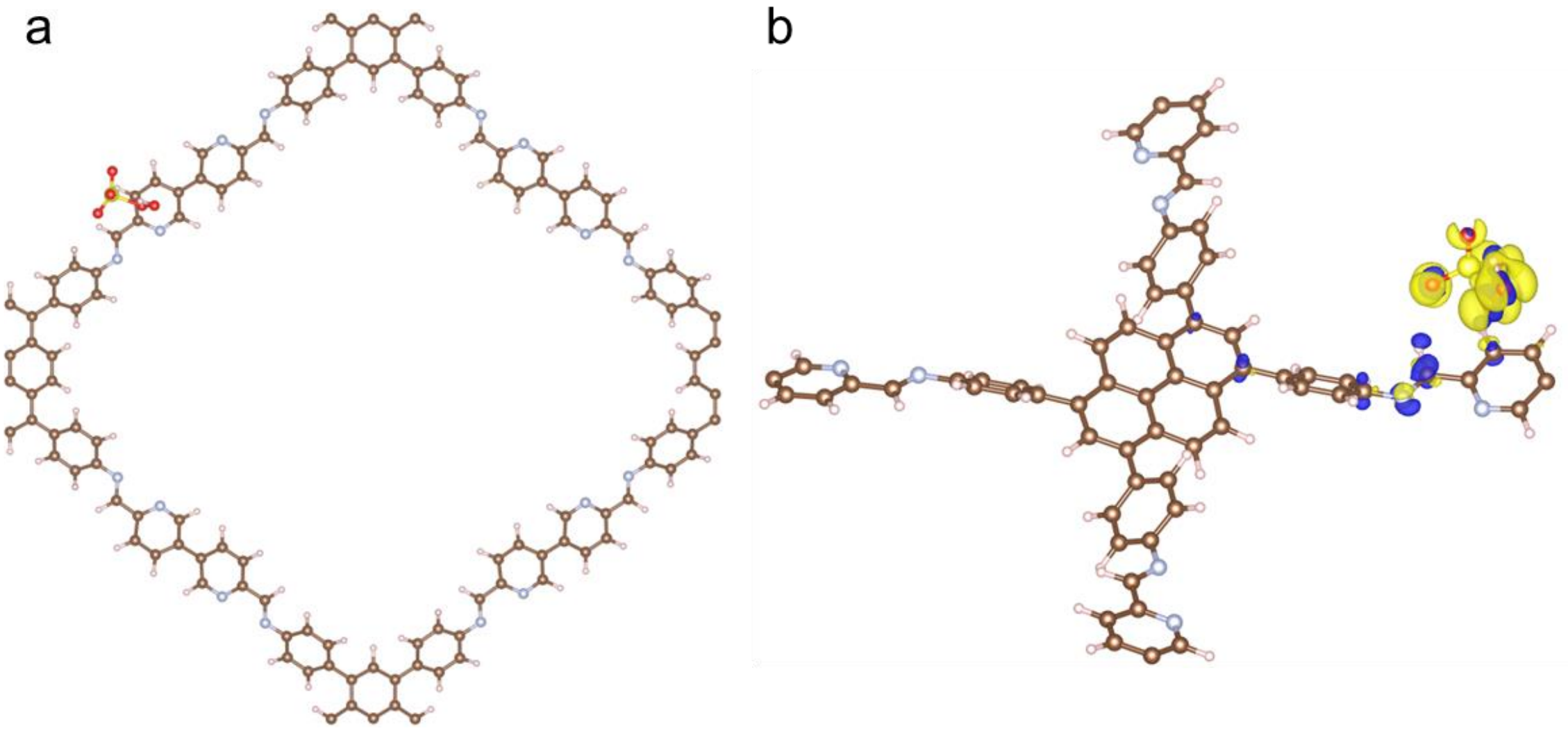

Supplementary Fig. 32 (a) PMS[1] adsorption configurations on TA-33Bpy. (b) Electron density differences (EDDs) of PMS[1] adsorption model on TA-33Bpy system. (Yellow and blue regions represent electron accumulation and electron depletion in (b), respectively.)

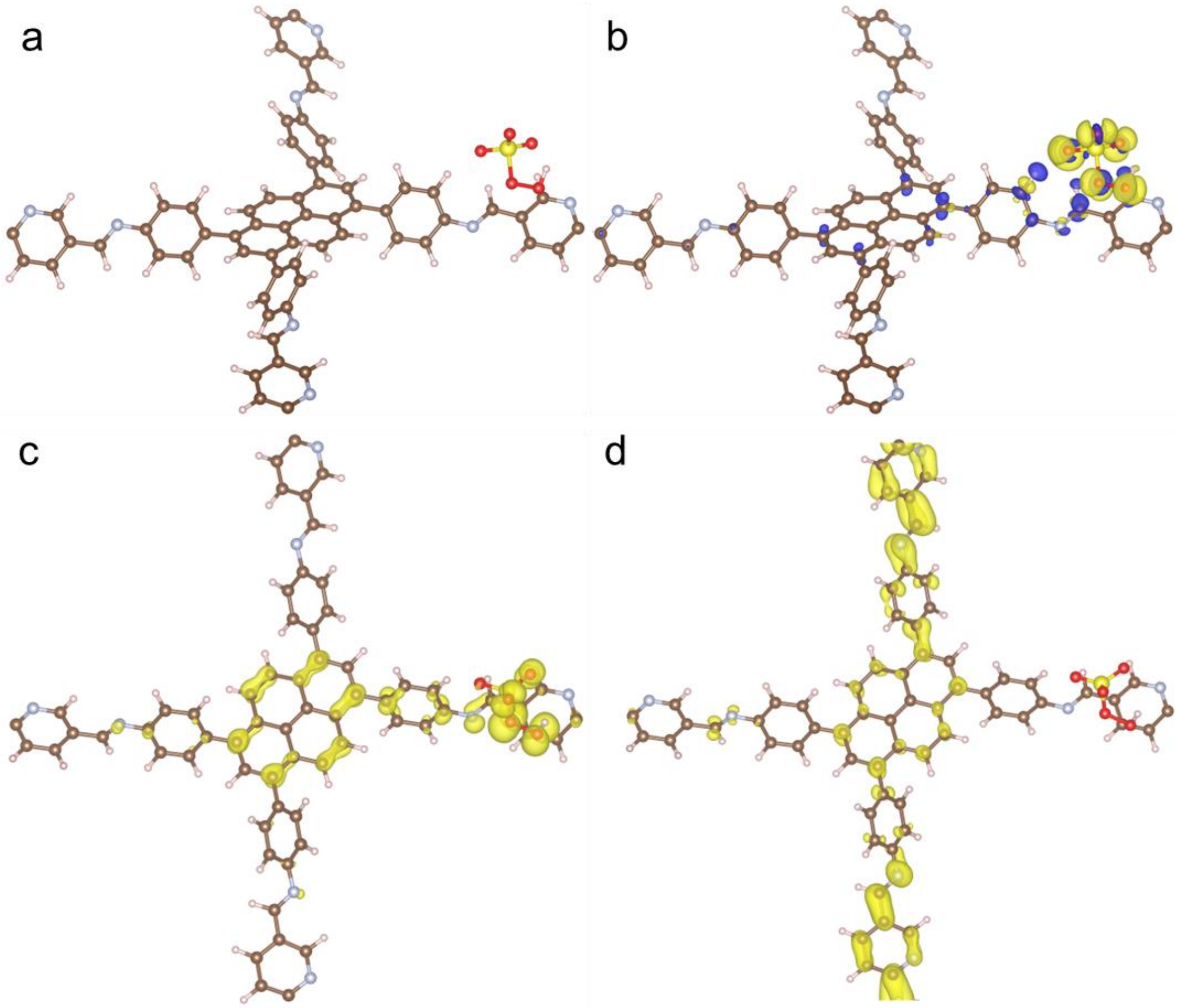

Supplementary Fig. 33 (a) PMS adsorption configurations on TA-22Bpy. (b) EDDs, (c) HOMO and (d) LUMO of TA-22Bpy/PMS[1] system. (Yellow and blue regions represent electron accumulation and electron depletion in (b), respectively.)

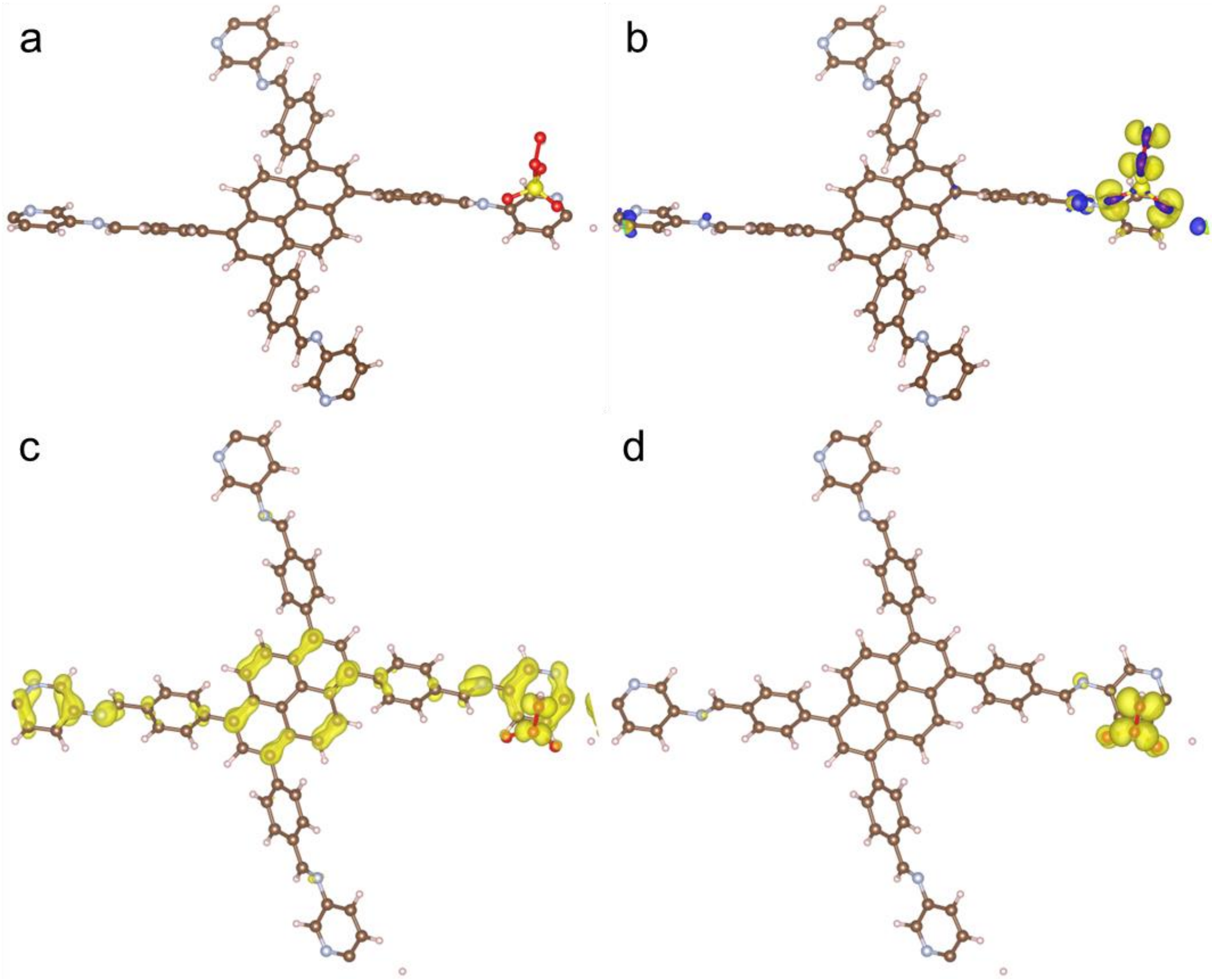

Supplementary Fig. 34 (a) PMS[1] adsorption configurations on TF-22Bpy. (b) EDDs, (c) HOMO and (d) LUMO of TF-22Bpy/PMS[1]/PMS[2] system. (Yellow and blue regions represent electron accumulation and electron depletion in (b), respectively.)

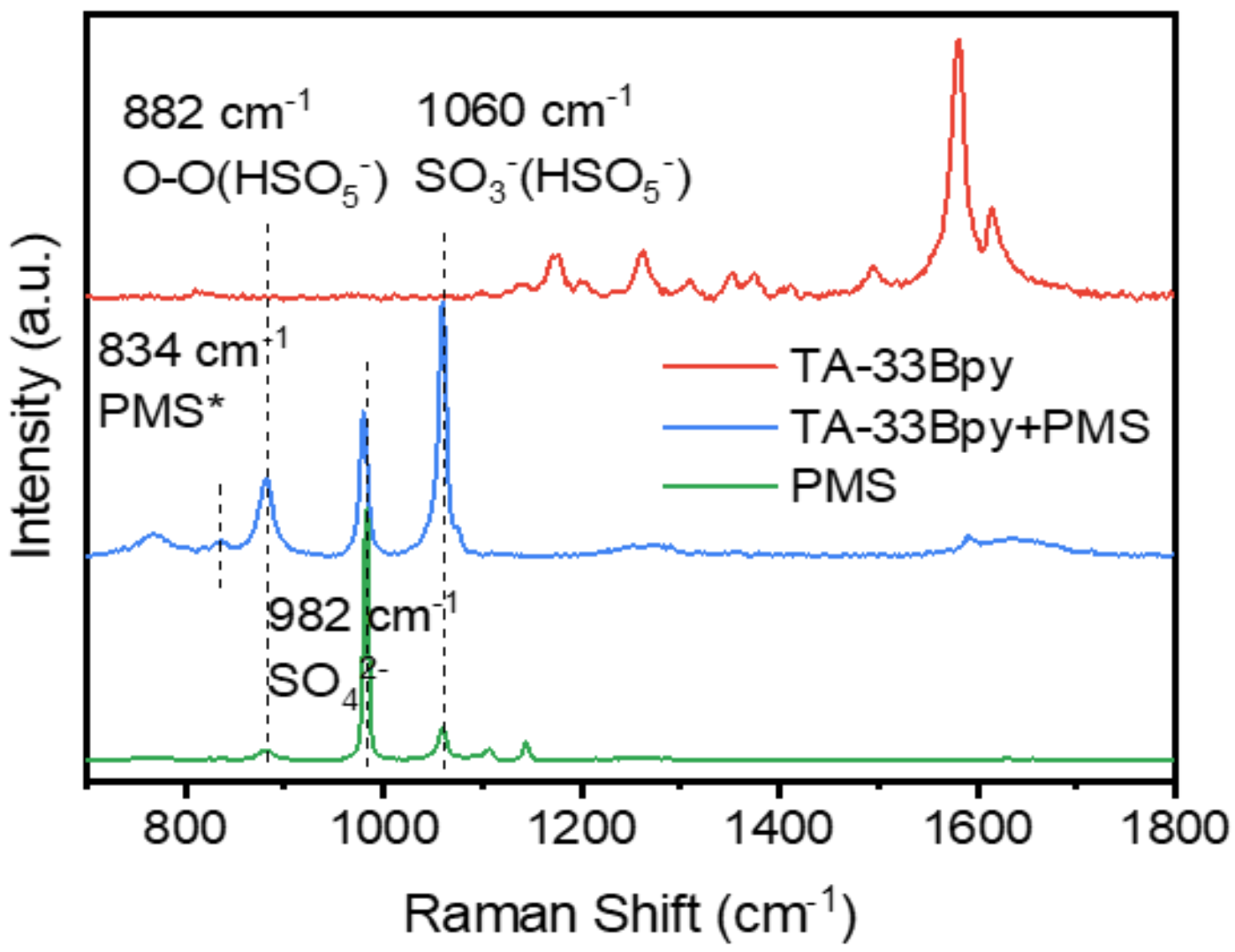

Supplementary Fig. 35 In situ Raman spectra of TA-33Bpy/PMS system.

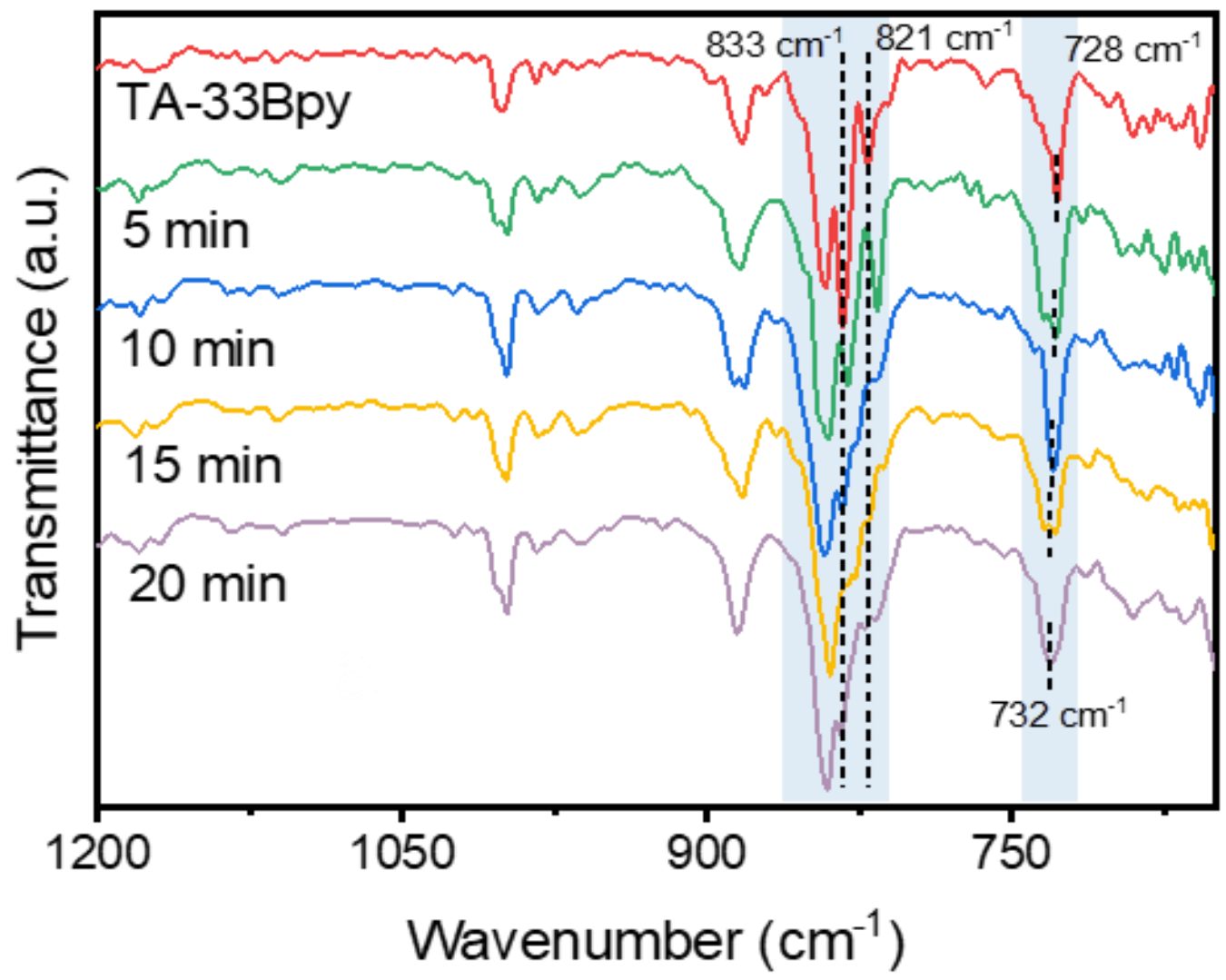

Supplementary Fig. 36 In situ FT-IR spectra of TA-33Bpy/PMS system.

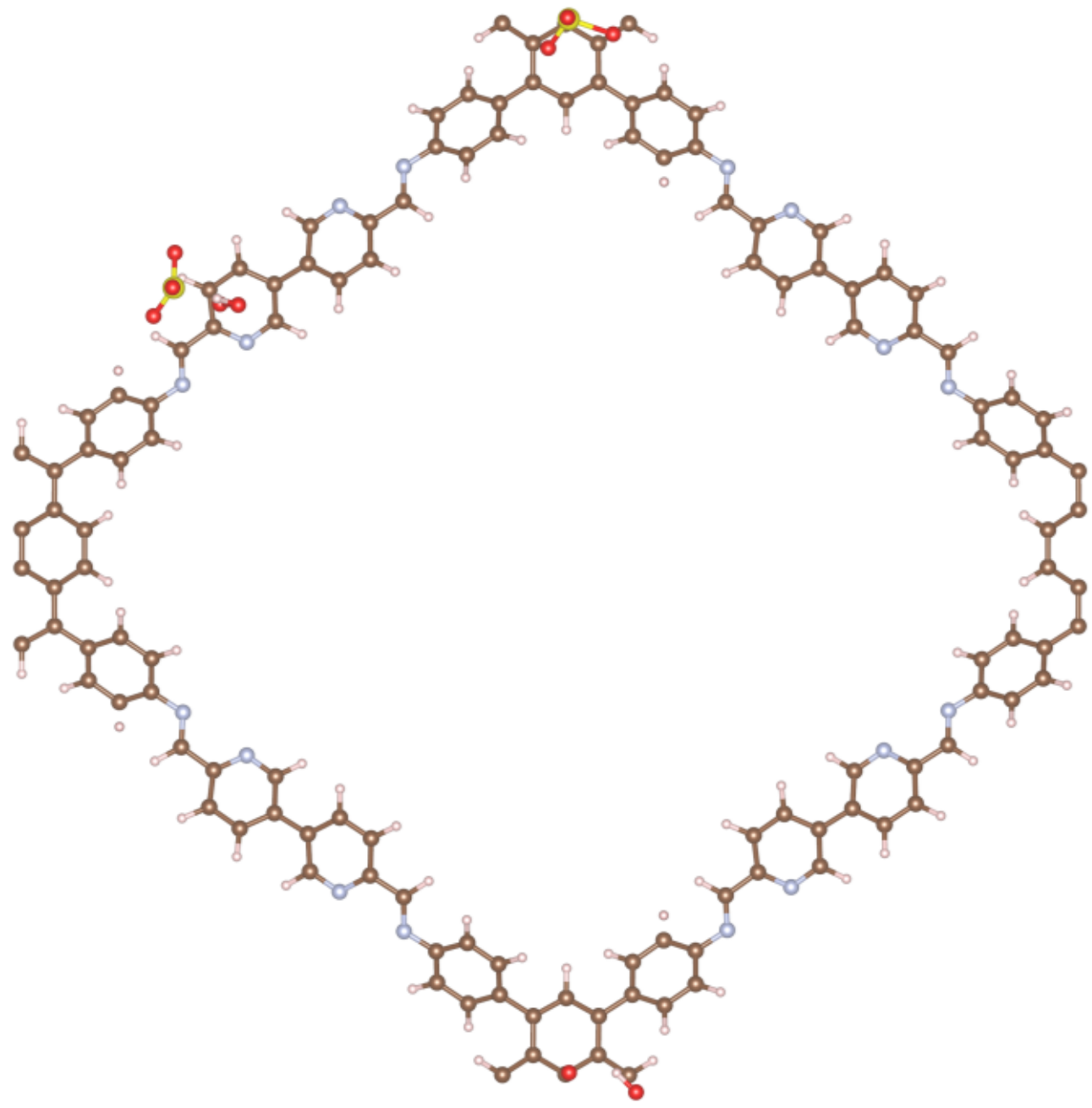

Supplementary Fig. 37 PMS^2 adsorption configurations on TA-33Bpy/PMS^1 system.

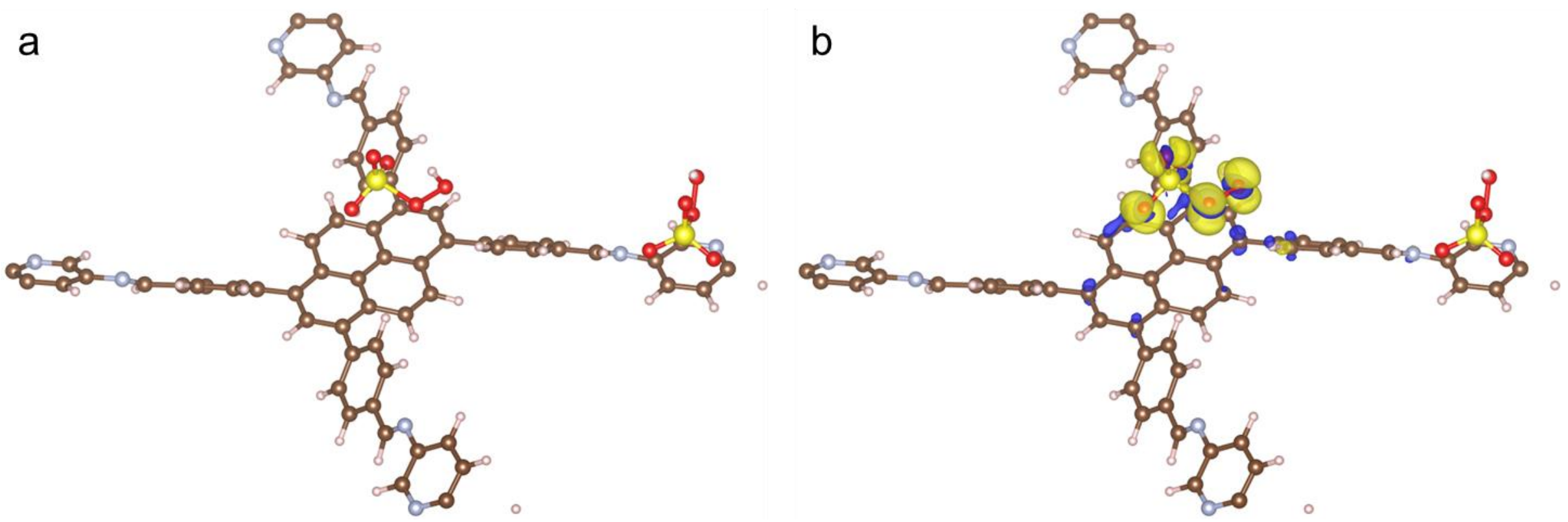

Supplementary Fig. 38 (a) The PMS^2 adsorption configurations on TF-22Bpy/PMS^1. (b) EDDs of TF-22Bpy/PMS^1 system. (Yellow and blue regions represent electron accumulation and electron depletion, respectively.)

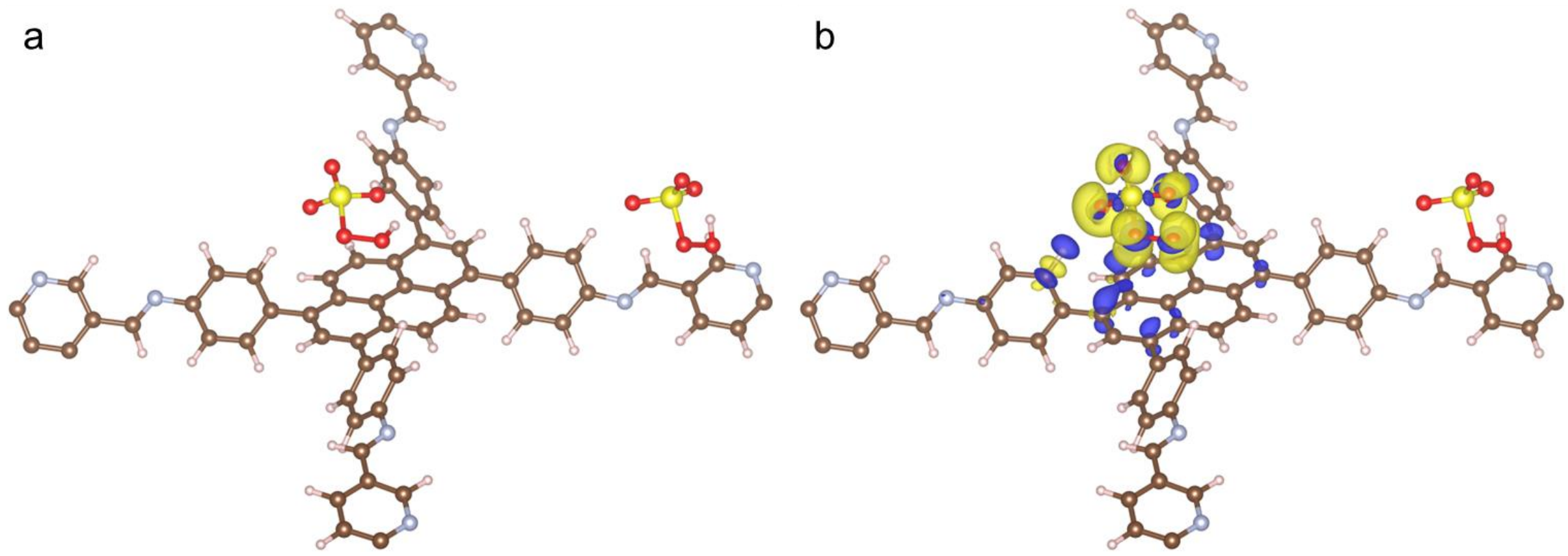

Supplementary Fig. 39 (a) PMS adsorption configurations on TA-22Bpy. (b) EDDs of TA-22Bpy/PMS[1] system. (Yellow and blue regions represent electron accumulation and electron depletion, respectively.)

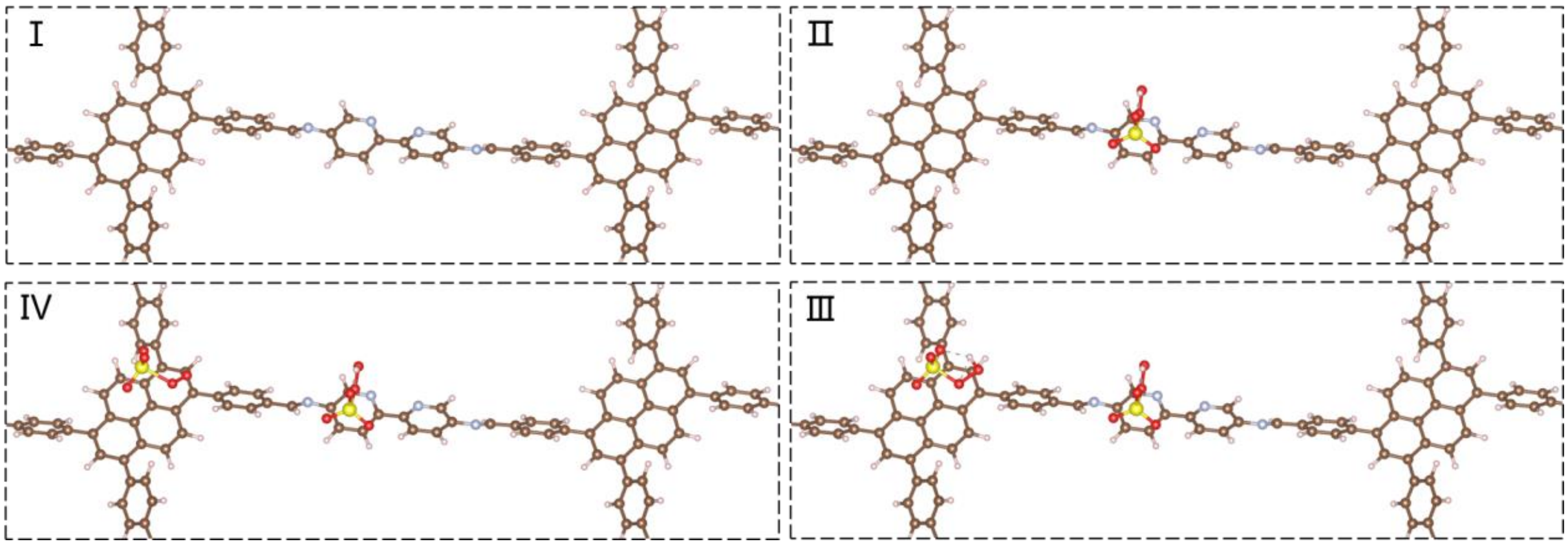

Supplementary Fig. 40 Pathway and optimized atomic structures for the main process of PMS activation on TF-22Bpy.

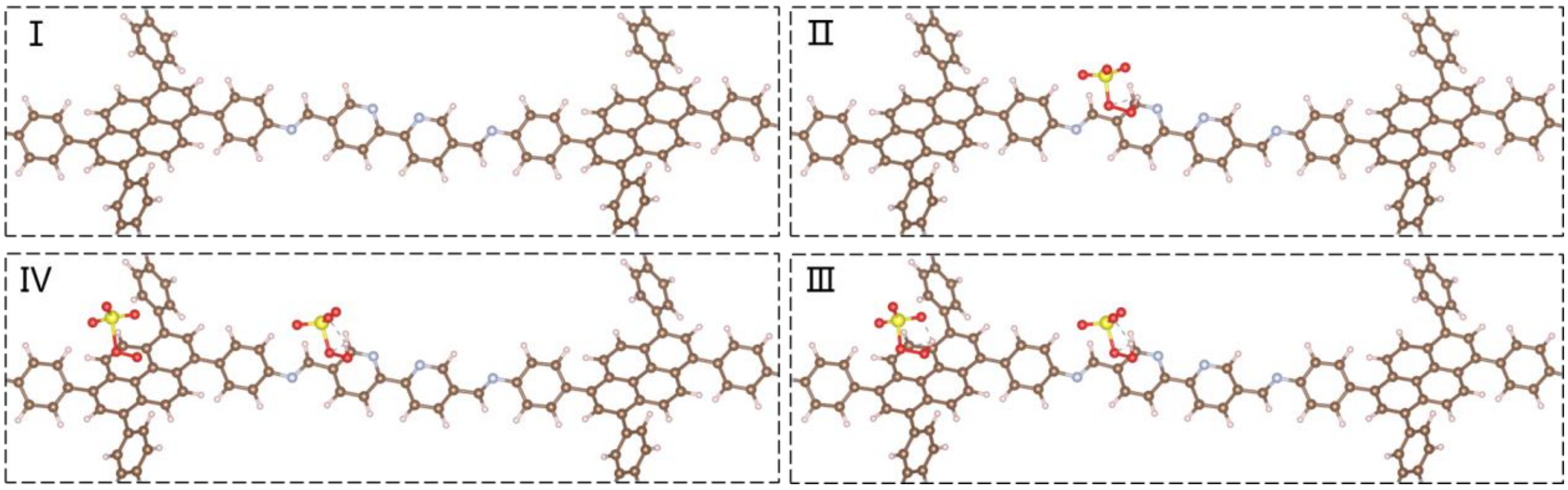

Supplementary Fig. 41 Pathway and optimized atomic structures for the main process of PMS activation on TA-22Bpy.

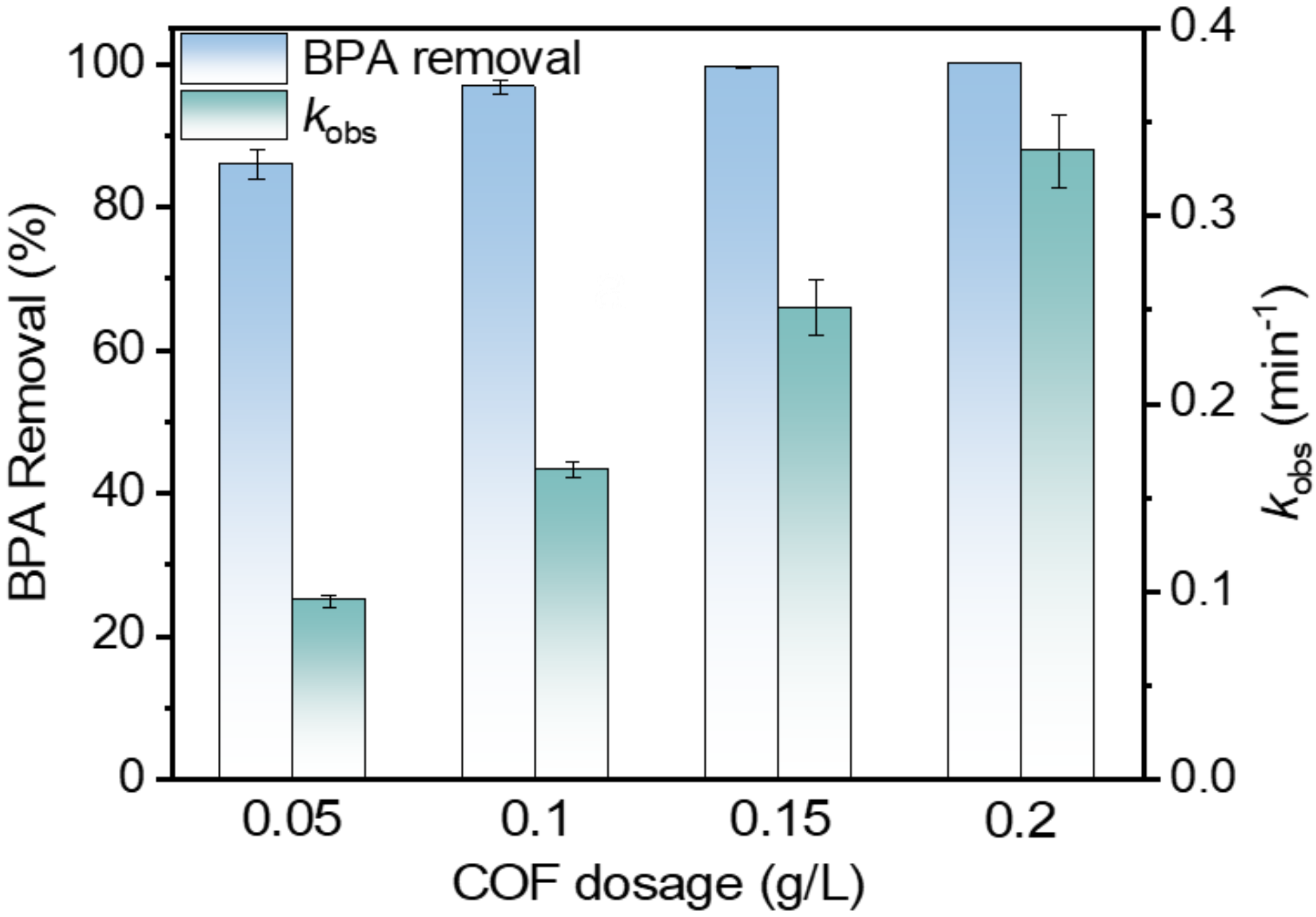

Supplementary Fig. 42 The influences of COF loading on BPA removal in TA-33Bpy/PMS system. Data for this figure is presented as mean values ± SD (n = 3).

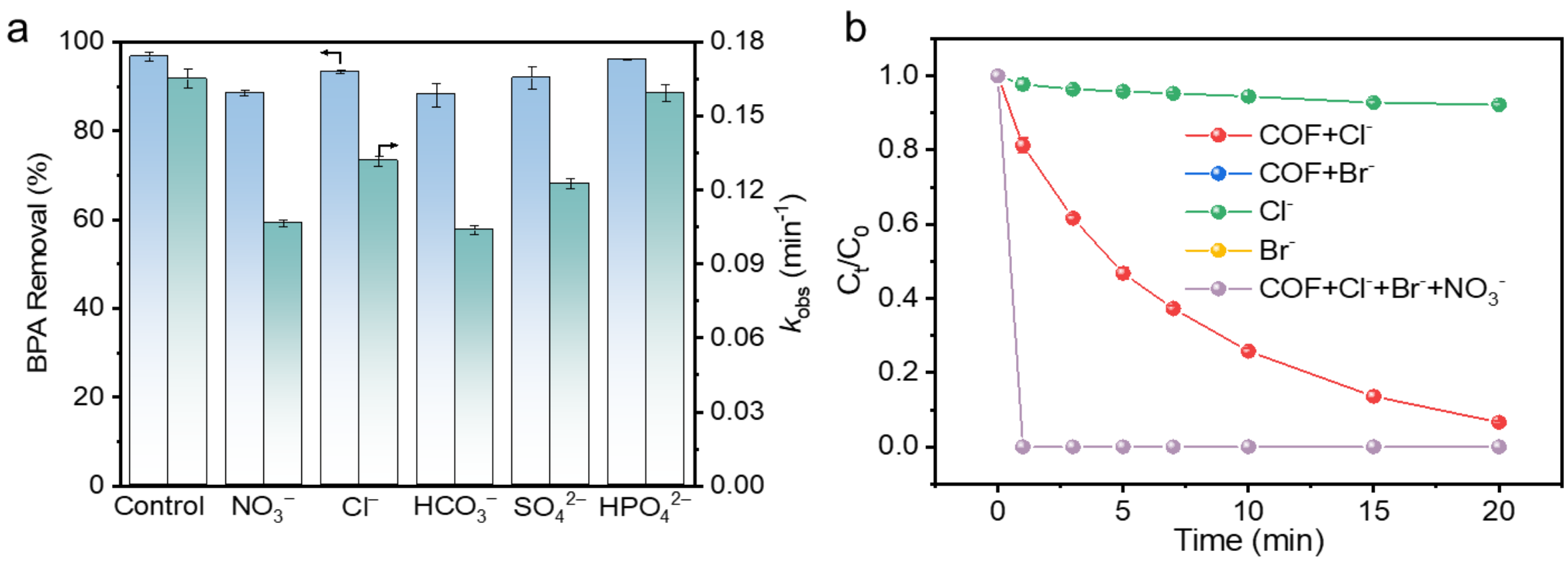

Supplementary Fig. 43 The influences of (a) coexisting ion and (b) halide ion on BPA removal in TA-33Bpy/PMS system. Data for (a-b) are presented as mean values ± SD (n = 3).

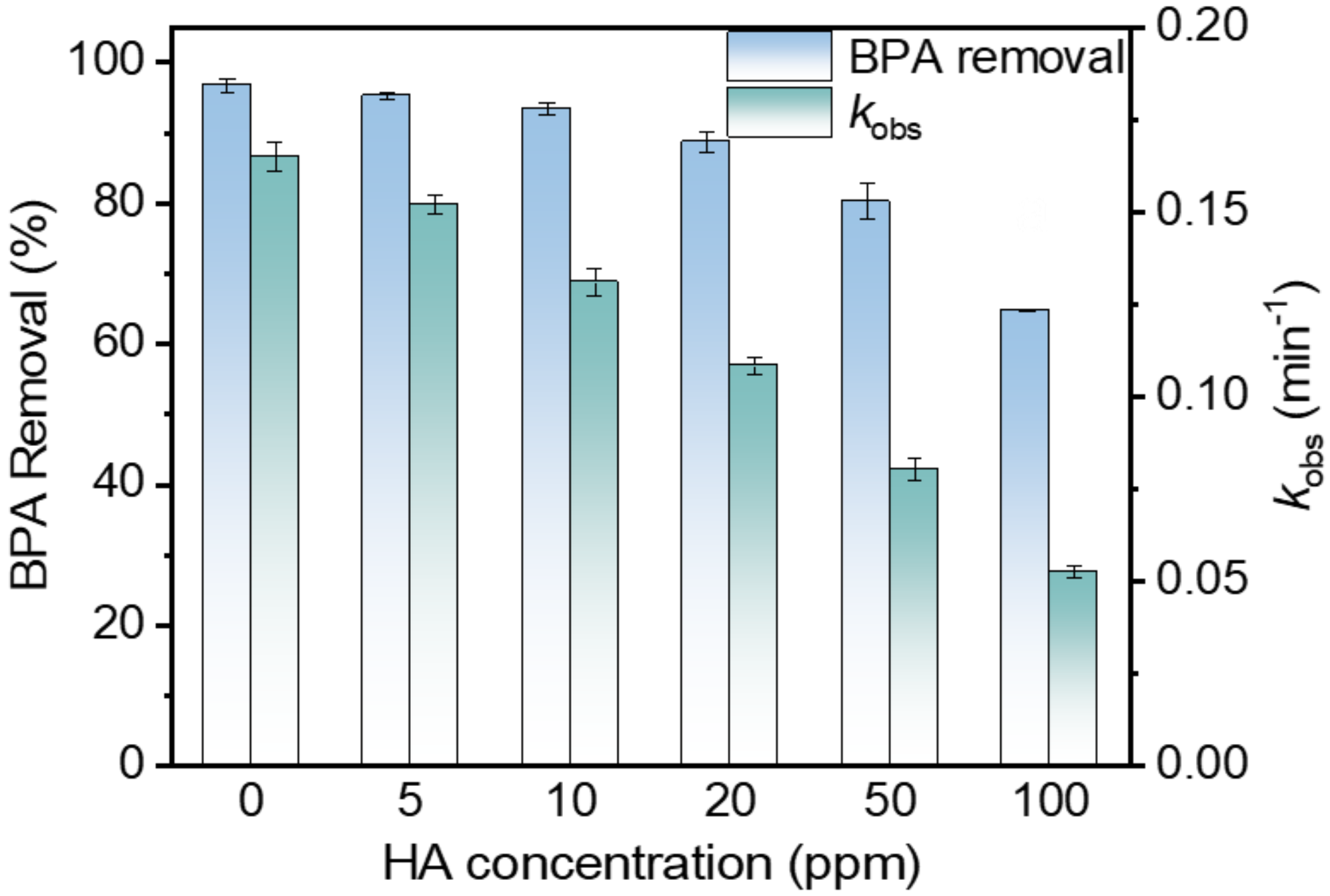

Supplementary Fig. 44 The influences of HA on BPA removal in TA-33Bpy/PMS system. Data for this figure is presented as mean values ± SD (n = 3).

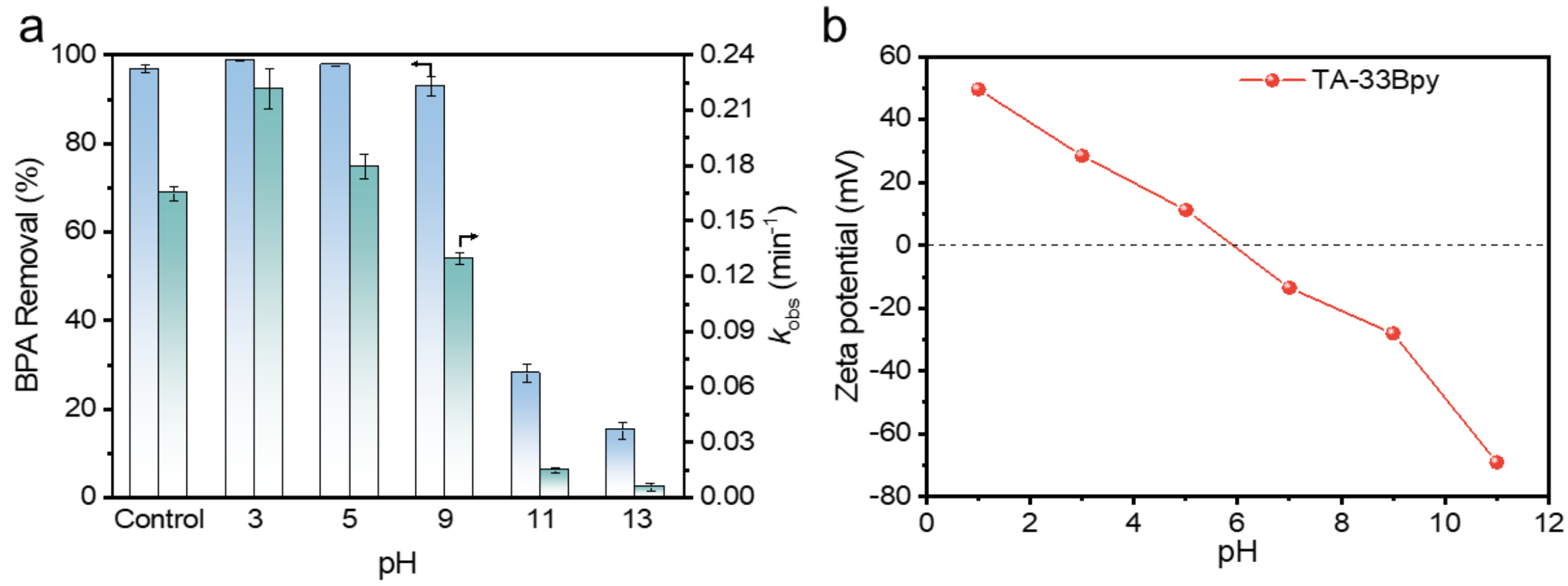

Supplementary Fig. 45 (a) The influence of pH on BPA removal in TA-33Bpy/PMS system. (b) Zeta potential of TA-33Bpy. Data for (a) is presented as mean values ± SD (n = 3).

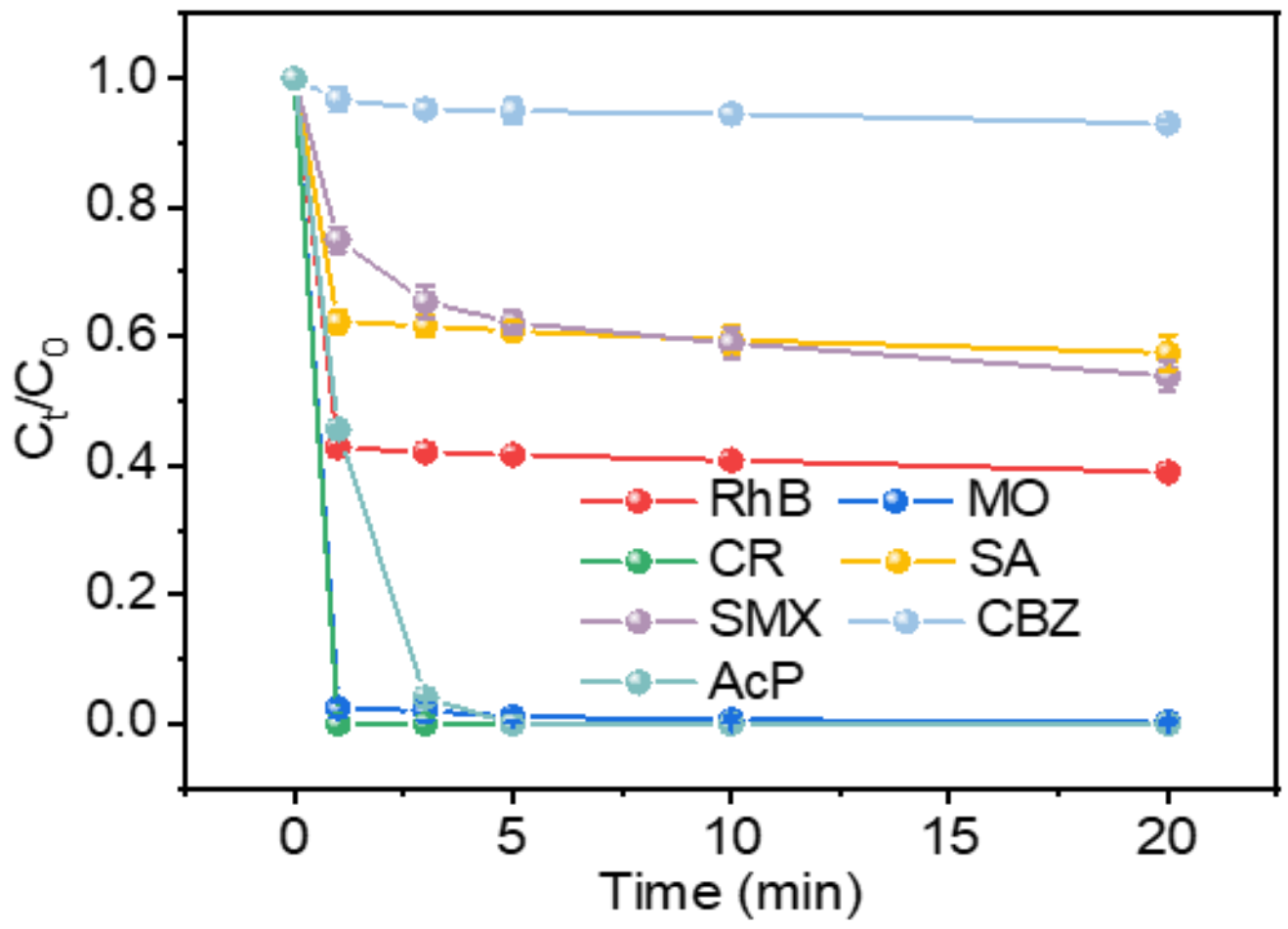

Supplementary Fig. 46 Removal of different pollutants by TA-33Bpy/PMS system. Data for this figure is presented as mean values ± SD (n = 3).

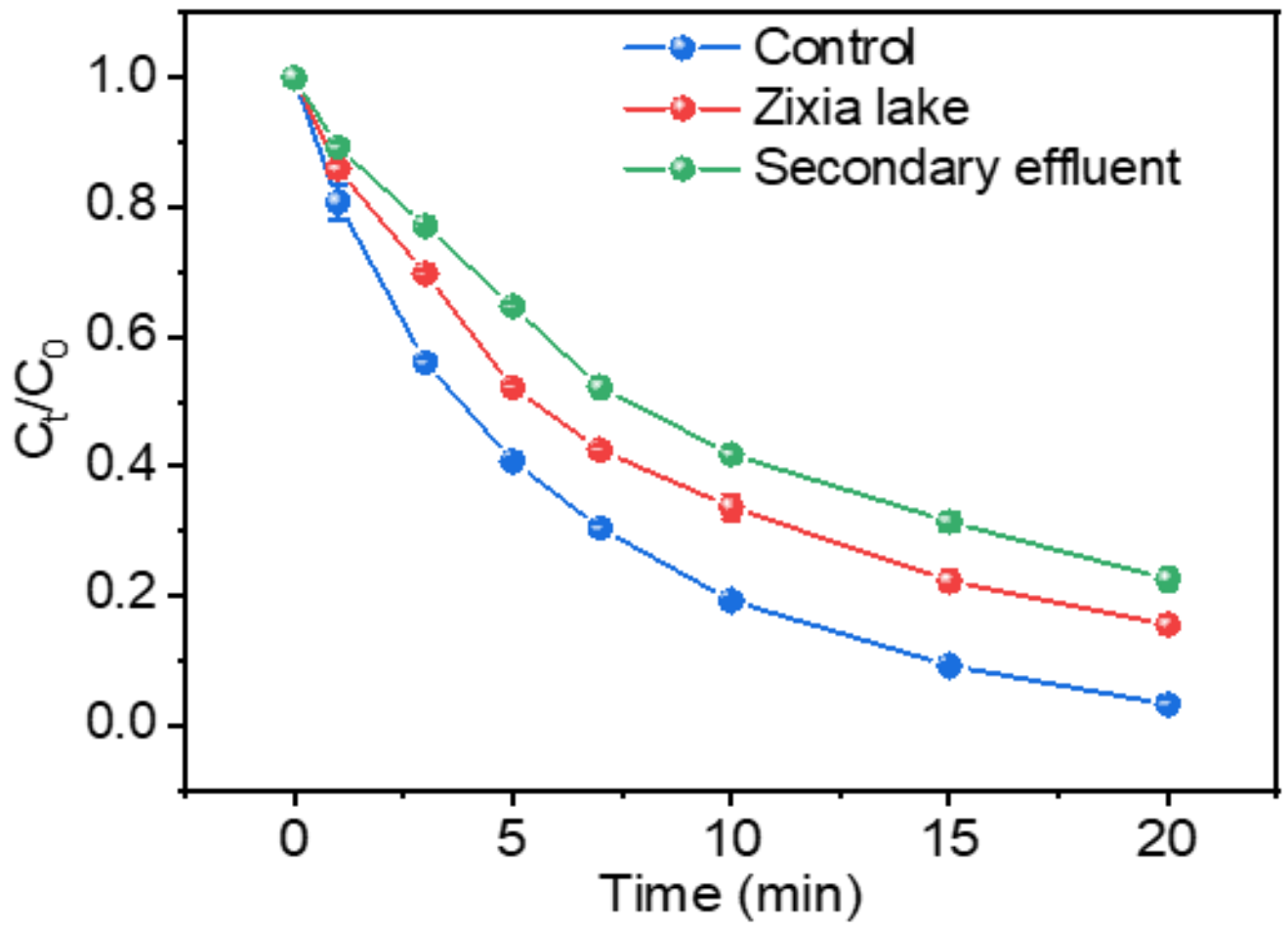

Supplementary Fig. 47 BPA Removal by TA-33Bpy/PMS system in actual water. Data for this figure is presented as mean values ± SD (n = 3).

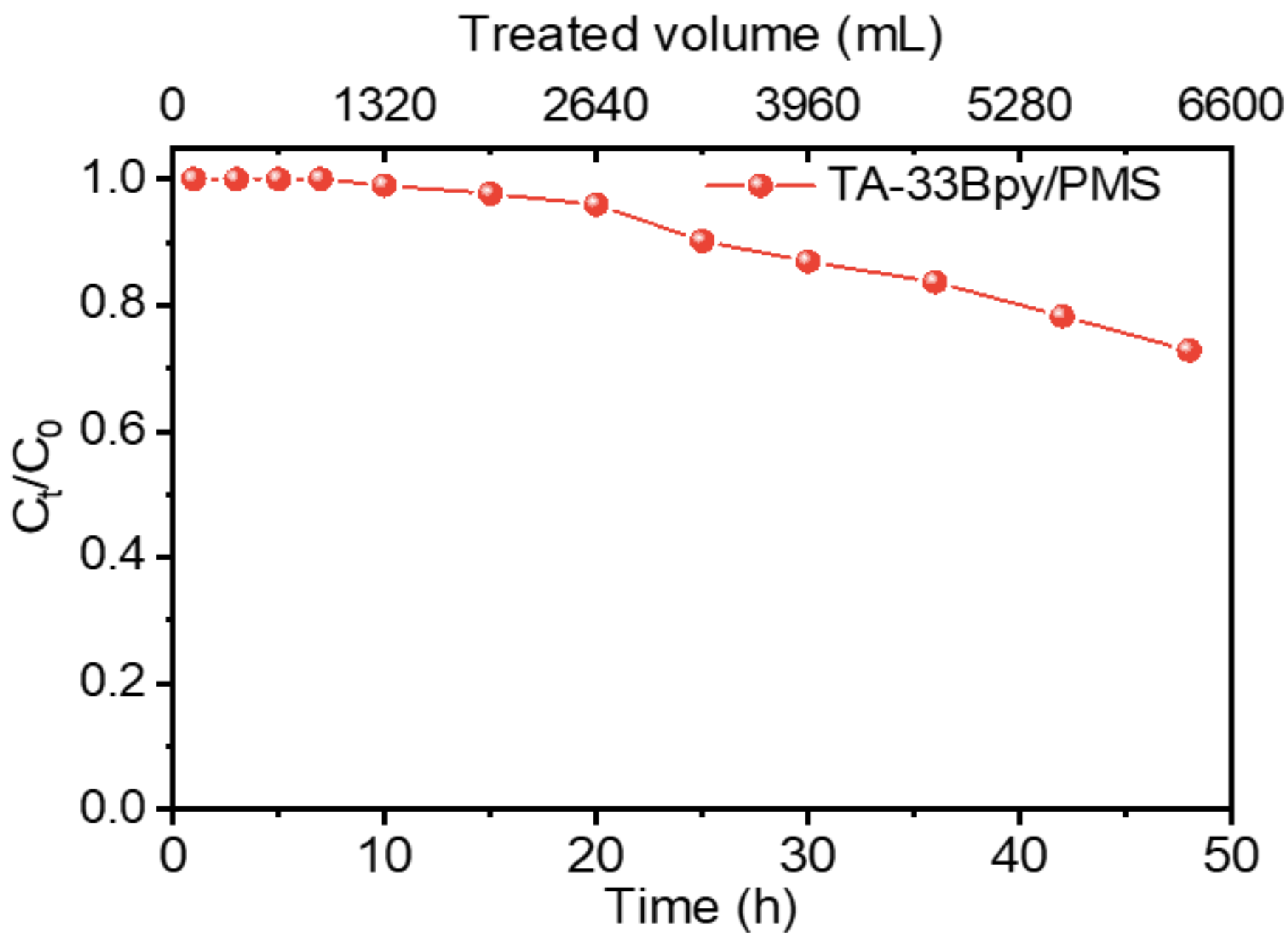

Supplementary Fig. 48 A continuous operation test of BPA degradation.

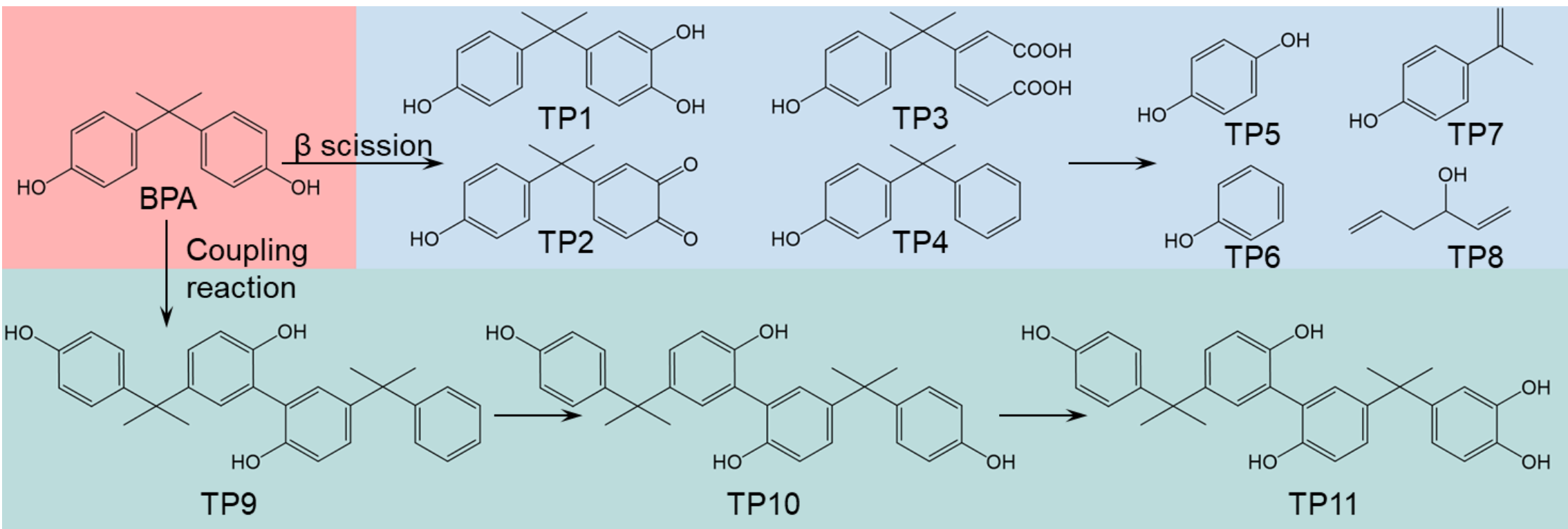

Supplementary Fig. 49 Possible degradation pathways of BPA in TA-33Bpy/PMS system.

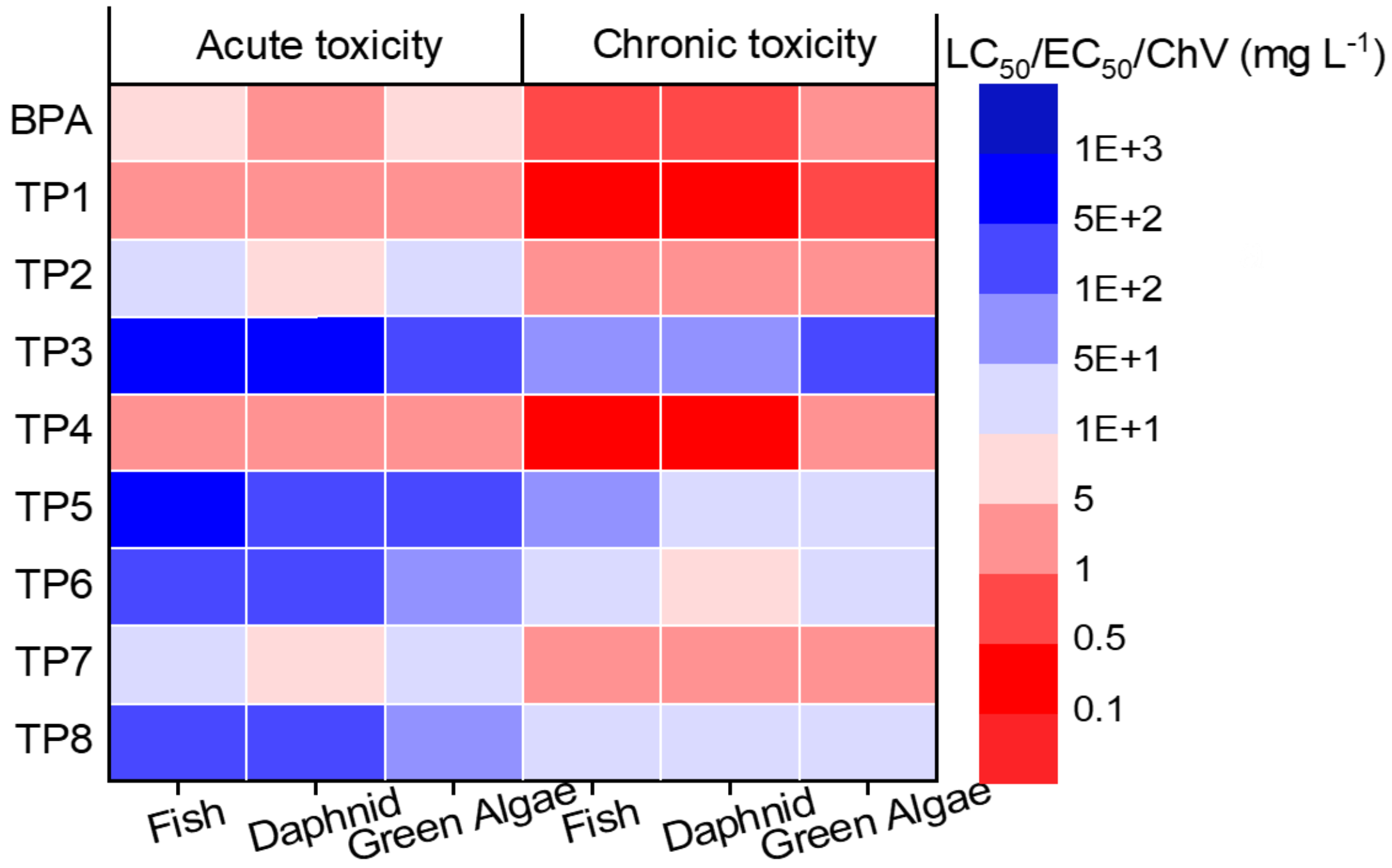

Supplementary Fig. 50 Biotoxicity assessment of BPA and its degradation intermediates.

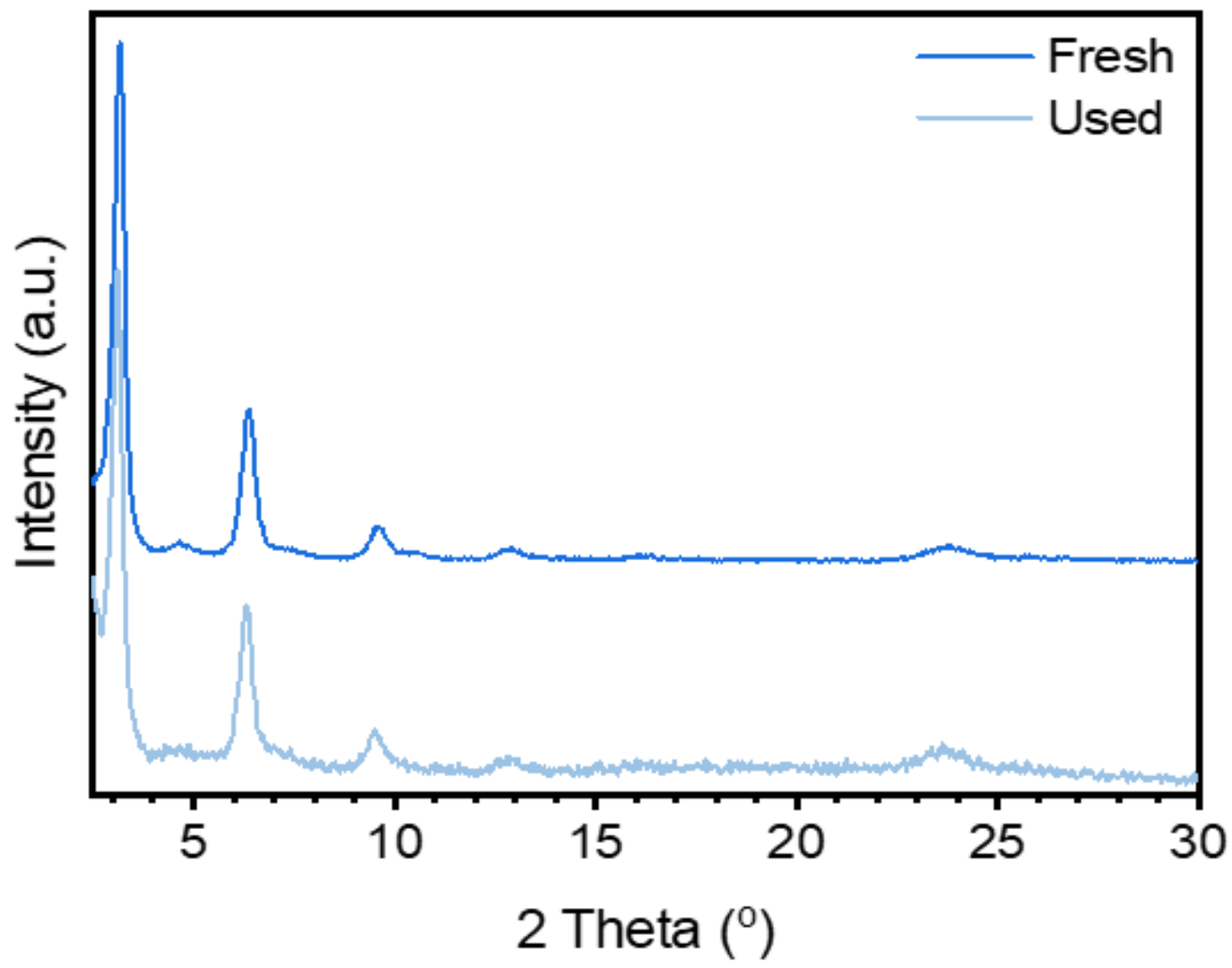

Supplementary Fig. 51 PXRD patterns of fresh and used TA-33Bpy.

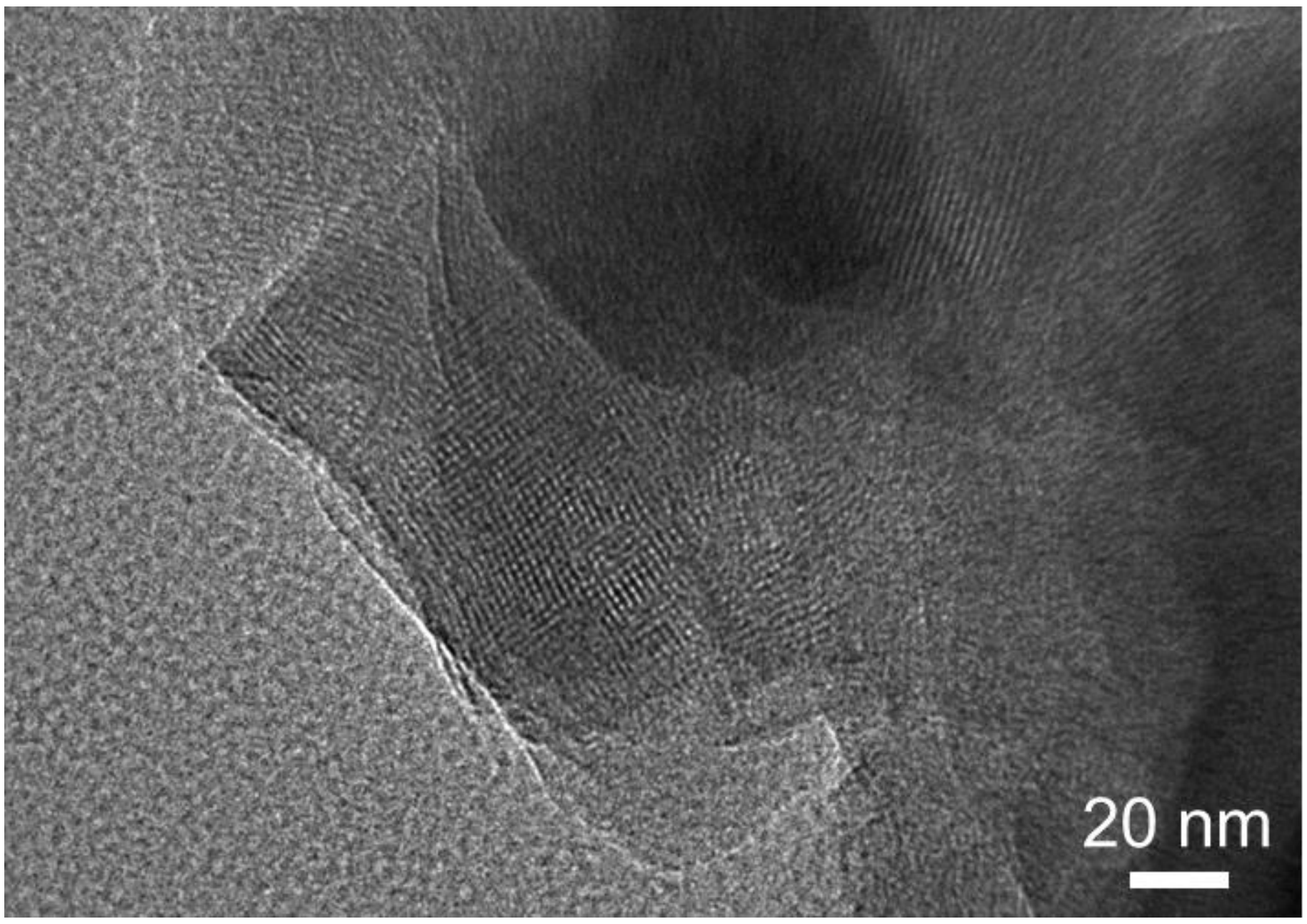

Supplementary Fig. 52 TEM image of the used TA-33Bpy.

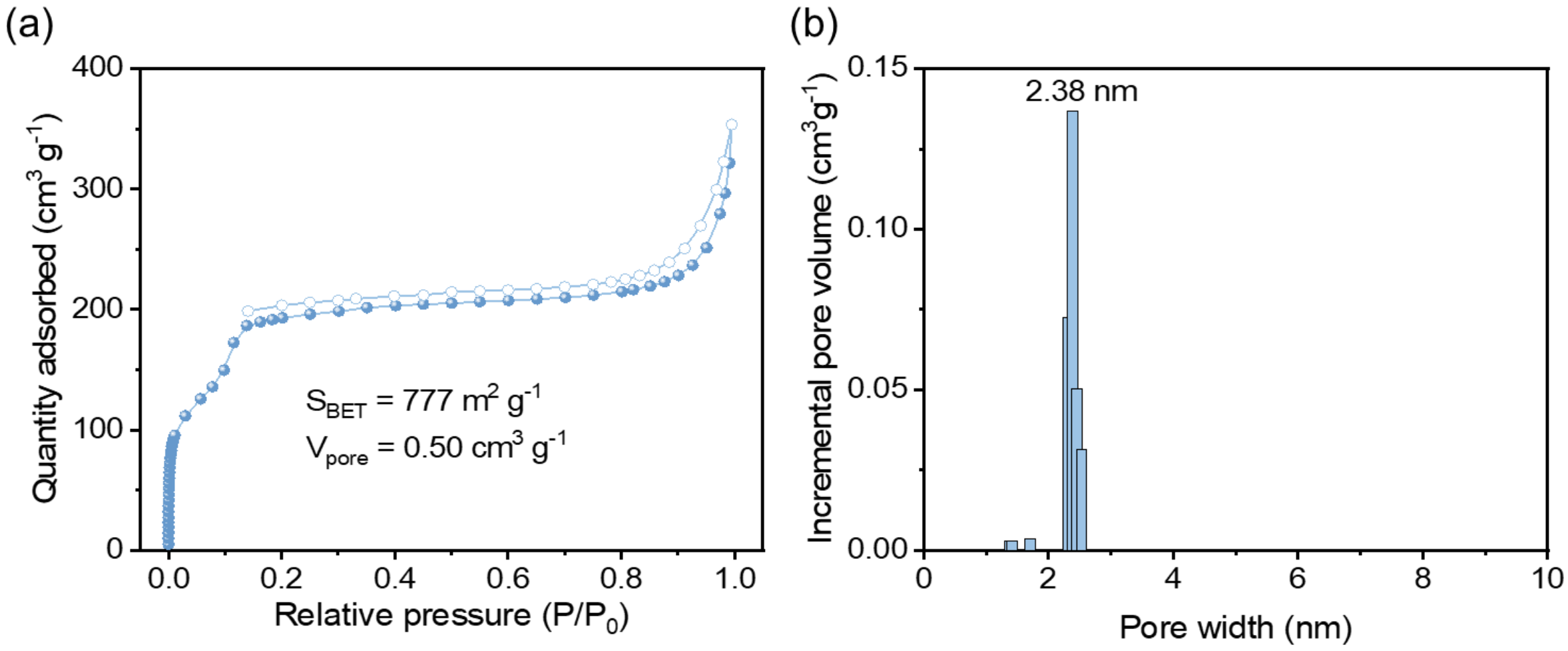

Supplementary Fig. 53 (a) N_2 adsorption/desorption isotherms and (b) pore size distribution of the used TA-33Bpy.

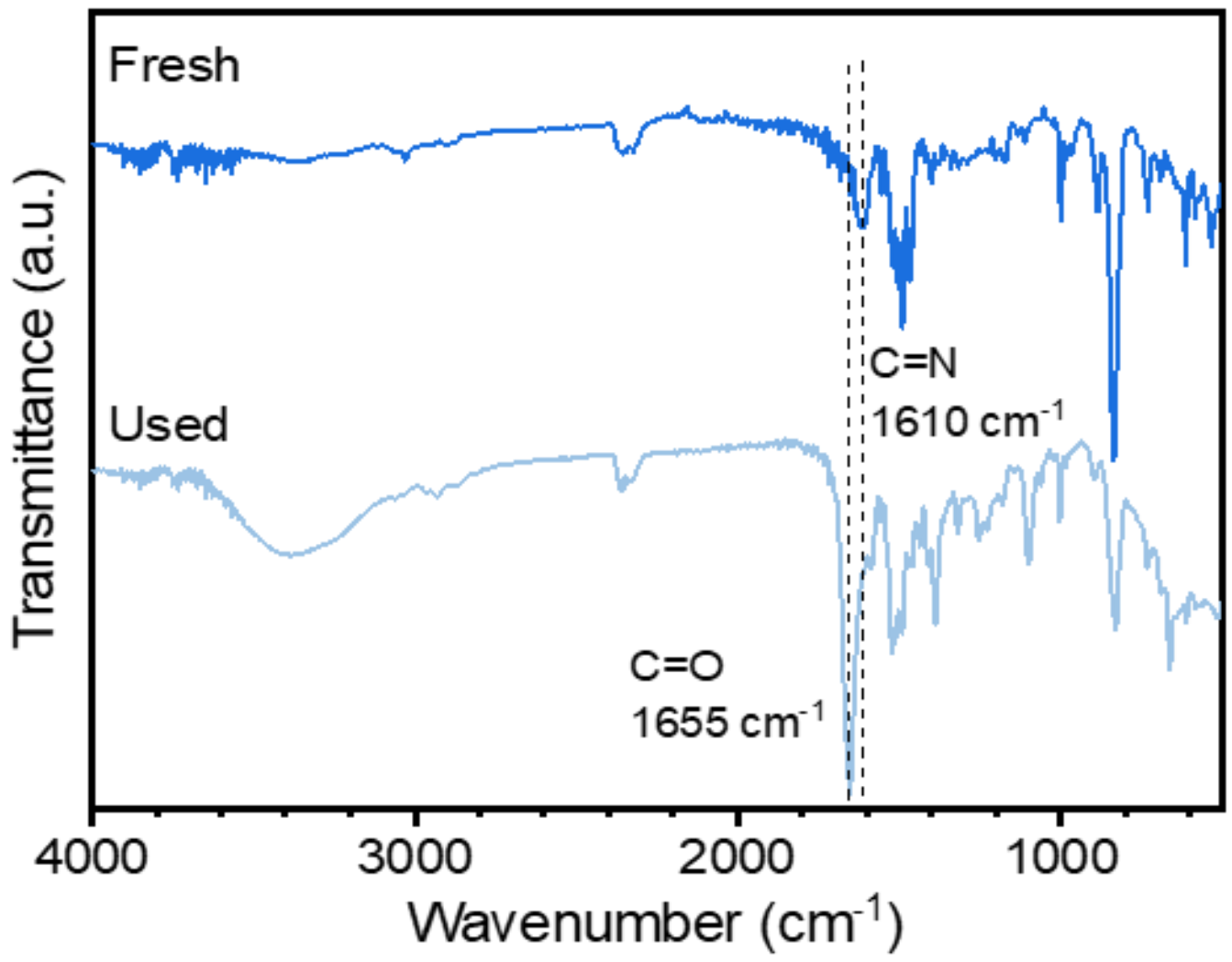

Supplementary Fig. 54 FT-IR spectra of fresh and used TA-33Bpy.

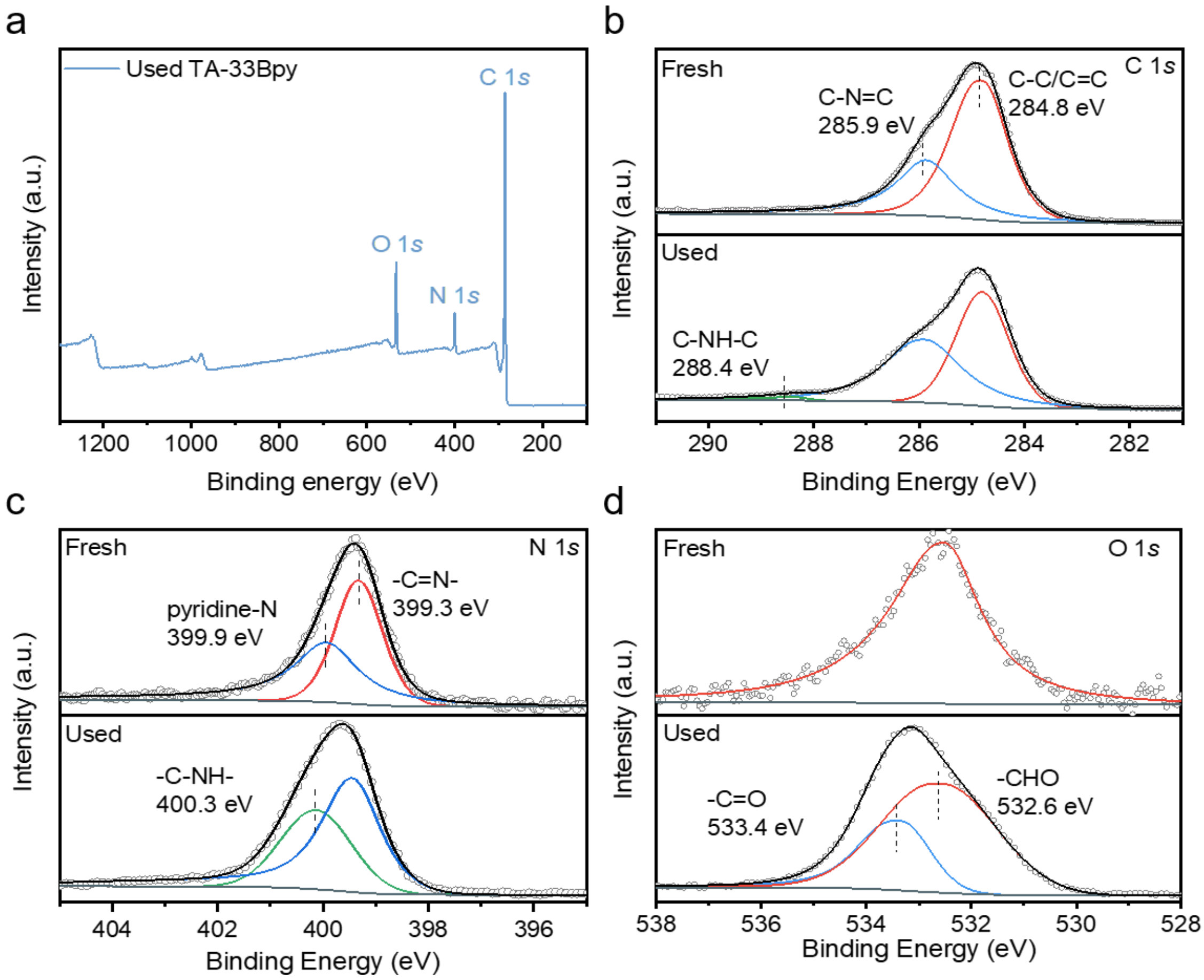

Supplementary Fig. 55 (a) XPS survey spectra, (b) high-resolution C 1*s* spectra, (c) N 1*s* spectra, (d) O 1*s* spectra of the used TA-33Bpy.

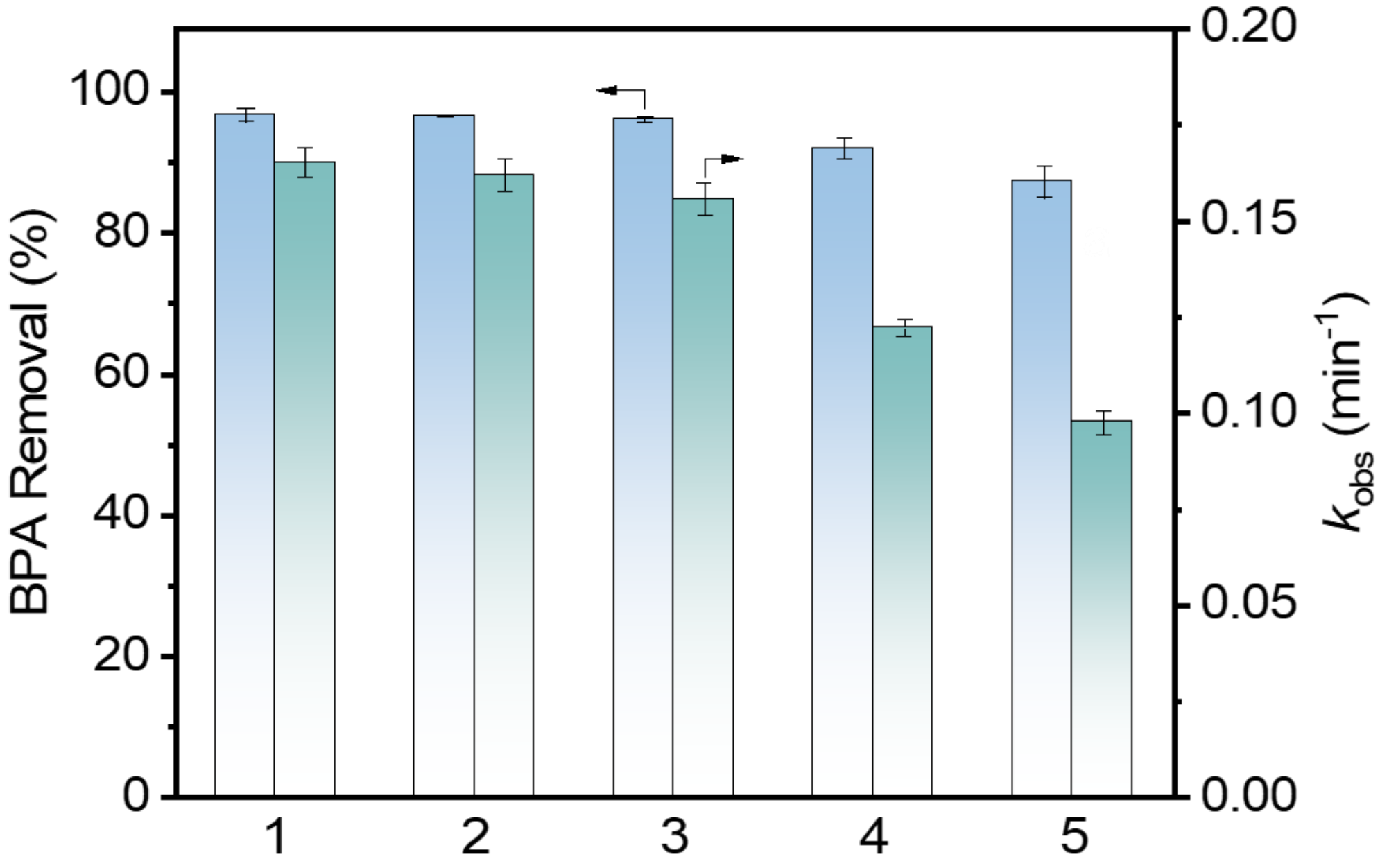

Supplementary Fig. 56 Recycling of TA-33Bpy. Data for this figure is presented as mean values ± SD (n = 3).

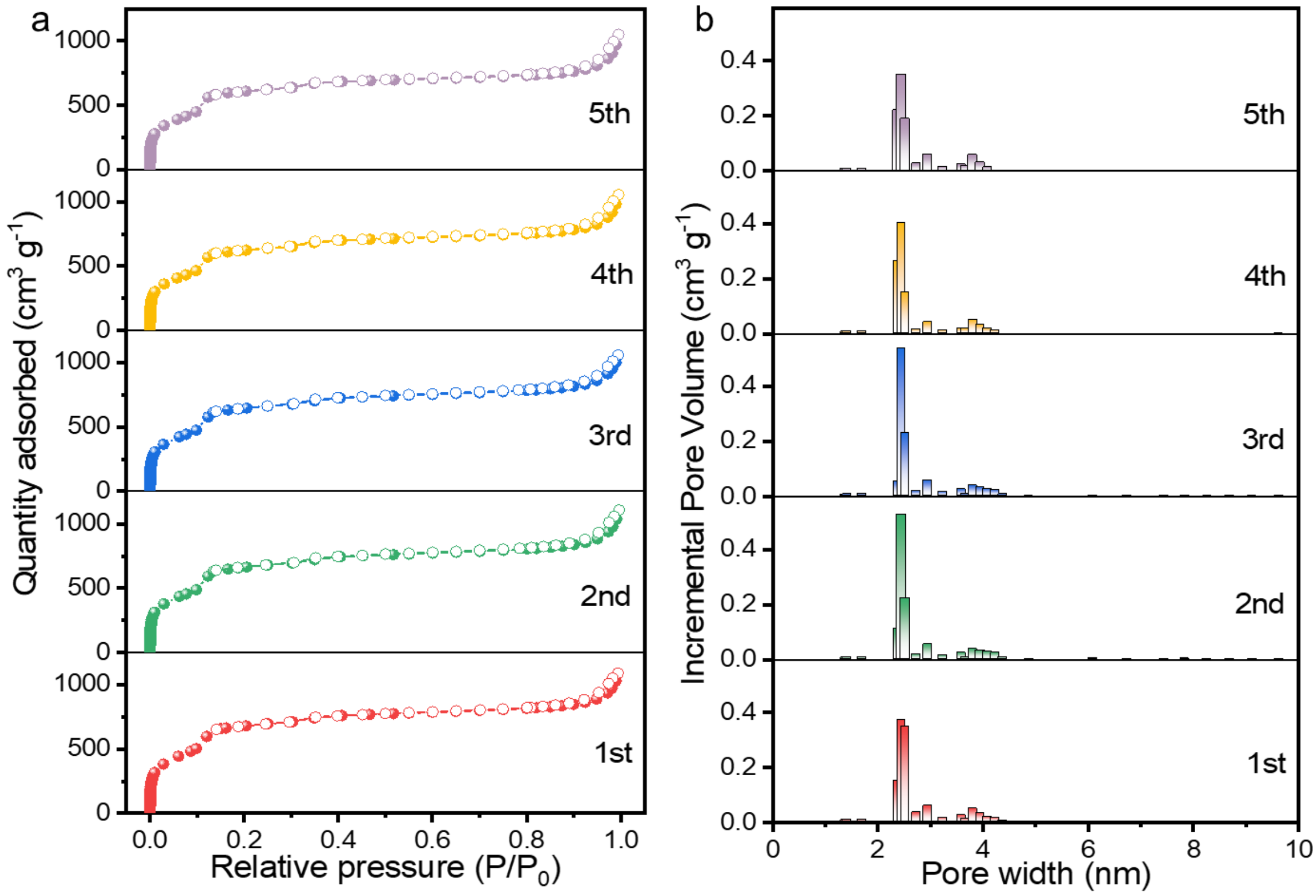

Supplementary Fig. 57 (a) N_2 adsorption-desorption isotherms, and (f) pore size distributions of the recycled TA-33Bpy.

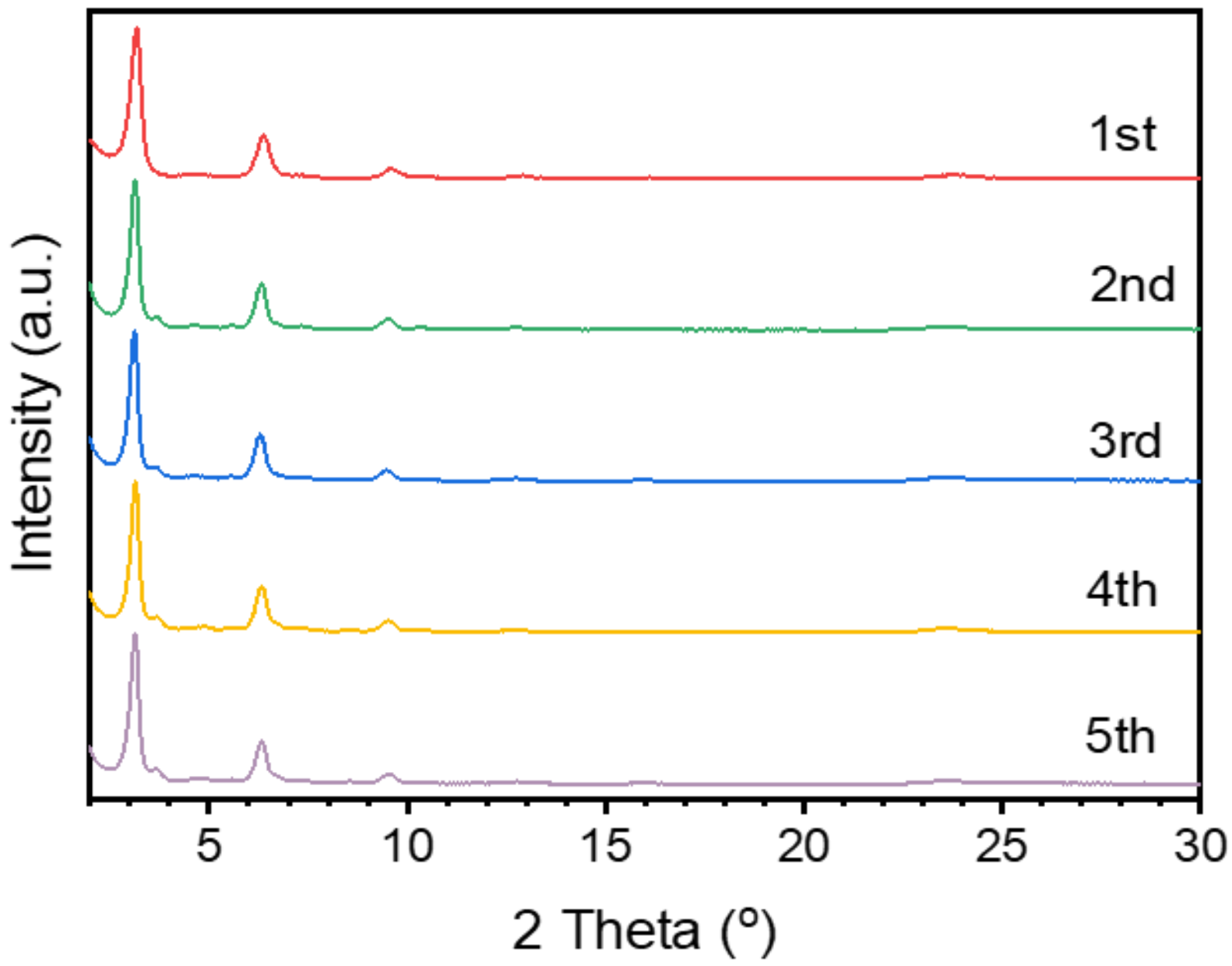

Supplementary Fig. 58 XRD patterns of the recycled TA-33Bpy.

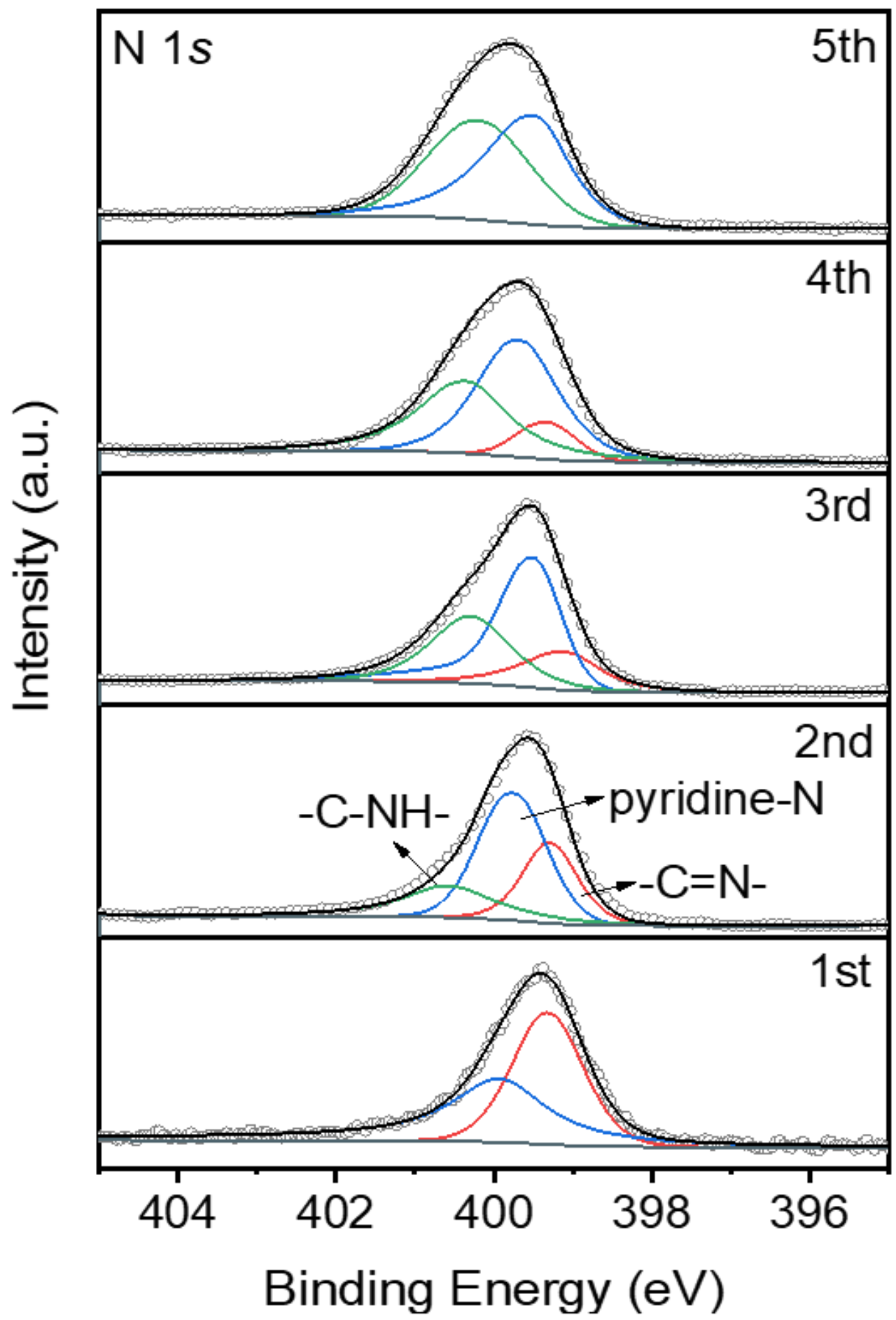

Supplementary Fig. 59 N 1*s* spectra of the recycled TA-33Bpy.

Supplementary Table 1 Rietveld refinement result of TF-22Bpy.

Name	TF-22Bpy
Chemical formula	$C_{128}H_{76}N_{16}$
Crystal system	Monoclinic
Unit cell	a = 42.620(23) Å, b =36.440(5) Å,<br>c = 3.846(8) Å<br>α = γ = 90.0º, β = 104.270(8)º
Space group	CM
Volume/Å^3	5789
X-ray source	Cu Kα
Wavelength/Å	1.5418
2θ	2-30º
Rp	4.25%
Rwp	7.54%
GOF	1.37
Z	4
Number of data points	1401

Supplementary Table 2 Fractional atomic coordinates and the unit cell of TF-22Bpy from the Rietveld refinement.

TF-22Bpy		Space group: CM<br>a = 42.620(23) Å, b =36.440(5) Å,<br>c = 3.846(8) Å<br>$\alpha = \gamma = 90.0^o$, $\beta = 104.270(8)^o$		
Name	Atom type	x	y	z
C1	C	0.06566	-0.51764	-0.08522
C2	C	0.03889	-0.46395	-0.03929
C3	C	0.00969	-0.48192	-0.03049
C4	C	-0.0194	-0.46389	-0.0198
C5	C	-0.04633	-0.48233	0.02322
C6	C	-0.0201	-0.57211	-0.04004
C7	C	0.01013	-0.58928	-0.0238
C8	C	0.04008	-0.57197	-0.01159
C9	C	0.07255	-0.59216	0.04598
C10	C	-0.05204	-0.59271	-0.08801
C11	C	0.0695	-0.62229	-0.1563
C12	C	0.09992	-0.64125	-0.0998
C13	C	0.13402	-0.63056	0.16203
C14	C	0.13707	-0.6011	0.37242
C15	C	0.10662	-0.58238	0.31887
C16	C	-0.08654	-0.58394	-0.36015
C17	C	-0.1163	-0.60364	-0.40853
C18	C	-0.11206	-0.63302	-0.19299
C19	C	-0.07753	-0.64247	0.06994
C20	C	-0.0479	-0.62261	0.12077
C21	C	-0.14373	-0.65327	-0.24095
N22	N	-0.14006	-0.67992	-0.03348
C23	C	-0.16789	-0.70182	-0.03672
C24	C	-0.20466	-0.69483	-0.24038
N25	N	-0.22938	-0.71636	-0.2199
C26	C	-0.22015	-0.74524	-0.00848
C27	C	-0.18355	-0.75283	0.20054
C28	C	-0.15774	-0.73103	0.18469
C29	C	0.16671	-0.64908	0.21179
N30	N	0.16487	-0.32392	0.01514

C31	C	0.19499	-0.69485	0.02529
C32	C	0.18967	-0.72801	-0.11458
N33	N	0.21731	-0.74611	-0.12087
C34	C	0.25101	-0.73308	0.00441
C35	C	0.25717	-0.69938	0.13753
C36	C	0.2292	-0.68042	0.14795
H37	H	0.08651	-0.53035	-0.12984
H38	H	-0.06717	-0.46961	0.06828
H39	H	0.0104	-0.61675	-0.02019
H40	H	0.04368	-0.63091	-0.36284
H41	H	0.09698	-0.66388	-0.26614
H42	H	0.16303	-0.59269	0.58061
H43	H	0.10965	-0.56026	0.49208
H44	H	-0.09038	-0.56204	-0.53884
H45	H	-0.14261	-0.59619	-0.61787
H46	H	-0.07358	-0.665	0.24026
H47	H	-0.02175	-0.63051	0.32657
H48	H	-0.17462	-0.77453	0.38517
H49	H	-0.12958	-0.73644	0.35039
H50	H	0.16339	-0.7394	-0.21785
H51	H	0.28292	-0.6873	0.22488
H52	H	-0.214	-0.67243	-0.41265
H53	H	0.23427	-0.65436	0.24198
H54	H	0.19228	-0.63957	0.41638
H55	H	-0.16973	-0.64538	-0.45111

Supplementary Table 3 Rietveld refinement result of TA-22Bpy.

Name	TA-22Bpy
Chemical formula	$C_{128}H_{76}N_{16}$
Crystal system	Monoclinic
Unit cell	a = 47.000(9) Å, b =36.470(8) Å,<br>c = 3.880(9) Å<br>α = γ = 90.0°, β = 109.600(23)°
Space group	CM
Volume/Å^3	6265
X-ray source	Cu Kα
Wavelength/Å	1.5418
2θ	2-30°
Rp	4.12%
Rwp	8.96%
GOF	1.29
Z	4
Number of data points	1401

Supplementary Table 4 Fractional atomic coordinates and the unit cell of TA-22Bpy from the Rietveld refinement.

TA-22Bpy		Space group: CM<br>a = 47.000(9) Å, b =36.470(8) Å,<br>c = 3.880(9) Å<br>α = γ = 90.0°, β = 109.600(23)°		
Name	Atom type	x	y	z
C1	C	0.06146	-0.51977	-0.10642
C2	C	0.03701	-0.45959	-0.0464
C3	C	0.01006	-0.47973	-0.02702
C4	C	-0.01673	-0.45948	-0.00493
C5	C	-0.04153	-0.48018	0.04854
C6	C	-0.01728	-0.58092	-0.02322
C7	C	0.01076	-0.60011	-0.01536
C8	C	0.03833	-0.58066	-0.01516
C9	C	0.06841	-0.60318	0.03088
C10	C	-0.04663	-0.60416	-0.05632
C11	C	0.0656	-0.63715	-0.16942
C12	C	0.09387	-0.65752	-0.13434
C13	C	0.12556	-0.64446	0.10599
C14	C	0.12834	-0.61156	0.31599
C15	C	0.10011	-0.59148	0.28515
C16	C	-0.07885	-0.59435	-0.31253
C17	C	-0.10614	-0.61634	-0.34049
C18	C	-0.10181	-0.6491	-0.1236
C19	C	-0.06954	-0.65969	0.12405
C20	C	-0.04235	-0.63764	0.1542
N21	N	-0.13072	-0.66927	-0.14367
C22	C	-0.13101	-0.70358	-0.02046
C23	C	-0.1617	-0.72049	-0.02771
C24	C	-0.19195	-0.70083	-0.174
N25	N	-0.21948	-0.71676	-0.16244
C26	C	-0.21921	-0.75194	-0.01406
C27	C	-0.1892	-0.77242	0.13498
C28	C	-0.16066	-0.75667	0.1261
N29	N	0.15542	-0.66151	0.11862
C30	C	0.15852	-0.30429	0.01083

C31	C	0.19035	-0.70821	0.0127
C32	C	0.195	-0.7463	-0.05974
N33	N	0.22406	-0.75762	-0.06673
C34	C	0.24968	-0.73312	-0.00489
C35	C	0.24565	-0.69447	0.06325
C36	C	0.2161	-0.68227	0.07344
H37	H	0.08047	-0.53401	-0.16293
H38	H	-0.06061	-0.46592	0.10392
H39	H	0.01117	-0.6309	-0.00925
H40	H	0.04164	-0.64732	-0.36274
H41	H	0.09087	-0.68216	-0.30945
H42	H	0.15265	-0.60165	0.50472
H43	H	0.10306	-0.56657	0.45768
H44	H	-0.08285	-0.56988	-0.49328
H45	H	-0.13085	-0.60826	-0.53492
H46	H	-0.06514	-0.68392	0.307
H47	H	-0.01798	-0.6464	0.34951
H48	H	-0.10864	-0.72048	0.09501
H49	H	-0.18718	-0.79972	0.26866
H50	H	-0.1376	-0.77241	0.24601
H51	H	0.13796	-0.2845	-0.08174
H52	H	0.17559	-0.76708	-0.10982
H53	H	0.26456	-0.67351	0.10079
H54	H	-0.19364	-0.67284	-0.29568
H55	H	0.21307	-0.65246	0.12335

Supplementary Table 5 Rietveld refinement result of TA-33Bpy.

Name	TA-33Bpy
Chemical formula	$C_{128}H_{76}N_{16}$
Crystal system	Monoclinic
Unit cell	a = 46.100(23) Å, b =36.570(4) Å,<br>c = 4.025(26) Å<br>α = γ = 90.0º, β = 110.700(6)º
Space group	CM
Volume/Å^3	6438
X-ray source	Cu Kα
Wavelength/Å	1.5418
2θ	2-30º
Rp	3.44%
Rwp	6.32%
GOF	1.44
Z	4
Number of data points	1401

Supplementary Table 6 Fractional atomic coordinates and the unit cell of TA-33Bpy from the Rietveld refinement.

TA-33Bpy		Space group: CM<br>a = 46.100(23) Å, b =36.570(4) Å,<br>c = 4.025(26) Å<br>$\alpha = \gamma = 90.0^{o}$, $\beta = 110.700(6)^{o}$		
Name	Atom type	x	y	z
C1	C	0.06278	-0.5192	-0.14931
C2	C	0.03848	-0.46074	-0.07062
C3	C	0.01195	-0.48032	-0.02679
C4	C	-0.01451	-0.4607	0.01805
C5	C	-0.03896	-0.48078	0.09396
C6	C	-0.01523	-0.57847	-0.00272
C7	C	0.01221	-0.59717	-0.02231
C8	C	0.03949	-0.57838	-0.04479
C9	C	0.06887	-0.60053	-0.02734
C10	C	-0.04434	-0.60084	-0.01481
C11	C	0.06585	-0.63363	-0.22437
C12	C	0.09327	-0.6546	-0.20464
C13	C	0.12431	-0.64296	0.01544
C14	C	0.12739	-0.61044	0.21609
C15	C	0.09995	-0.58979	0.20155
C16	C	-0.07556	-0.5909	-0.24564
C17	C	-0.10266	-0.61211	-0.25777
C18	C	-0.09921	-0.64411	-0.04832
C19	C	-0.06786	-0.65507	0.17158
C20	C	-0.04082	-0.63366	0.18684
N21	N	-0.12816	-0.66391	-0.06364
C22	C	-0.12996	-0.69374	0.11396
C23	C	-0.16167	-0.71038	0.07648
N24	N	-0.18974	-0.69363	-0.12831
C25	C	-0.21941	-0.70799	-0.1647
C26	C	-0.22204	-0.74117	0.01049
C27	C	-0.19326	-0.75889	0.22123
C28	C	-0.16312	-0.74359	0.25374
N29	N	0.15328	-0.66215	0.02486
C30	C	0.15516	-0.30291	-0.04267

C31	C	0.18623	-0.71334	-0.03552
C32	C	0.1898	-0.75177	-0.04306
C33	C	0.21956	-0.76674	-0.03178
C34	C	0.24569	-0.74339	-0.01665
C35	C	0.24073	-0.70497	-0.01745
N36	N	0.21165	-0.69111	-0.02454
H37	H	0.08166	-0.53302	-0.22075
H38	H	-0.05788	-0.46696	0.16478
H39	H	0.01236	-0.62706	-0.01996
H40	H	0.04236	-0.64293	-0.40015
H41	H	0.09026	-0.67894	-0.37254
H42	H	0.15124	-0.60163	0.38906
H43	H	0.103	-0.56546	0.37033
H44	H	-0.07894	-0.56692	-0.41947
H45	H	-0.1266	-0.604	-0.43422
H46	H	-0.06387	-0.67952	0.33793
H47	H	-0.01715	-0.64256	0.36131
H48	H	-0.10848	-0.70688	0.29572
H49	H	-0.24085	-0.69368	-0.33414
H50	H	-0.194	-0.78384	0.37078
H51	H	-0.14111	-0.75719	0.42087
H52	H	0.13424	-0.28503	-0.09433
H53	H	0.16991	-0.76995	-0.05102
H54	H	0.22231	-0.7965	-0.026
H55	H	0.25986	-0.68591	-0.0149

Supplementary Table 7 Textural properties of the COFs samples.

Samples	Surface Area (m^2 g^{-1})	Pore Volume (cm^3 g^{-1})	Pore Size (nm)
TF-22Bpy	2265	0.95	2.50
TA-22Bpy	2565	1.23	2.52
TA-33Bpy	2223	1.29	2.48

Supplementary Table 8 Catalytic performance comparison of recently reported catalysts for BPA removal.

Type	Catalyst (g L^{-1})	PMS (mM)	BPA (mg L^{-1})	k_{obs} (min^{-1})	*k-value* (min^{-1} M^{-1})	Ref.
COFs	TA-33Bpy (0.1)	0.65	20	0.165	50.77	This work
	BPY-COFs (0.3)	5	10	1.250	8.33	1
	BPD-COFs (0.3)	5	10	0.130	0.87	
Carbon Material	NOPC-1 (0.2)	0.15	10	0.152	50.49	2
	NOPC-2 (0.2)	0.15	10	0.187	62.12	
	NOPC (0.2)	0.15	10	0.300	99.65	
	NOPC-3 (0.2)	0.15	10	0.064	21.26	
	NOPC-4 (0.2)	0.15	10	0.026	8.64	
	g-C_3N_4(0.2)	0.15	10	0.004	1.23	
	NC-1 (0.15)	0.4	10	0.066	10.97	3
	NC-2 (0.15)	0.4	10	0.016	2.72	
	NC-3 (0.15)	0.4	10	0.003	0.57	
Single-Atom Catalyst	Fe-SAC (0.1)	1.30	25	0.100	19.20	4
	Co-SAC (0.1)	1.30	25	0.080	15.36	
	Mn-SAC (0.1)	1.30	25	0.060	11.52	
	Ni-SAC (0.1)	1.30	25	0.050	9.60	
	Cu-SAC (0.1)	1.30	25	0.050	9.60	
Metal/Carbon Material	FM@NBC-8 (0.1)	0.5	20	0.685	273.90	5
	F@NBC-8 (0.1)	0.5	20	0.035	13.87	
	M@NBC-8 (0.1)	0.5	20	0.007	3.00	
	NBC-8 (0.1)	0.5	20	0.007	2.94	
	ZIF(FeC)-Cl (0.1)	0.5	20	0.276	110.56	6
	ZIF(FeC) (0.1)	0.5	20	0.036	14.44	

k-value was calculated by the following Eq. 5:

$$k\text{-}value = (k_{obs} \times C_{[Pollutant]}) / (C_{[Catalyst]} \times C_{[PMS]}) \quad (5)$$

Supplementary Table 9 Textural properties, k_{obs} and area-normalized k of TA-33Bpy with different recycle.

Number of use	Surface Area (m^2 g^{-1})	Pore Volume (cm^3 g^{-1})	Pore Size (nm)	k_{obs} (min^{-1})	area-normalized k ($\times 10^{-5}$)
1	2223	1.29	2.48	0.165	7.42
2	2150	1.30	2.48	0.162	7.53
3	2095	1.26	2.48	0.156	7.45
4	1999	1.24	2.48	0.122	6.10
5	1982	1.22	2.48	0.098	4.94

Supplementary Table 10 Nitrogen spices of TA-33Bpy with different recycle.

Number of use	-C=N- (at.%)	Pyridine-N (at.%)	-C-NH- (at.%)
1	48.78	51.22	0
2	26.95	50.93	22.12
3	18.31	50.47	31.22
4	10.84	49.88	39.28
5	0	50.59	49.41

Supplementary References

1 Weng, Z. *et al.* Site Engineering of Covalent Organic Frameworks for Regulating Peroxymonosulfate Activation to Generate Singlet Oxygen with 100 % Selectivity. *Angew. Chem. Int. Ed.* **62**, e202310934 (2023).

2 He, Y.-L. *et al.* Activating peroxymonosulfate by N and O co-doped porous carbon for efficient BPA degradation: A re-visit to the removal mechanism and the effects of surface unpaired electrons. *Applied Catalysis B: Environmental* **314**, 121390 (2022).

3 Qu, G. *et al.* Enhanced peroxymonosulfate activation via heteroatomic doping defects of pyridinic and pyrrolic N in 2D N-doped carbon nanosheets for BPA degradation. *Journal of Hazardous Materials* **461**, 132626 (2024).

4 Gao, Y. *et al.* Activity Trends and Mechanisms in Peroxymonosulfate-Assisted Catalytic Production of Singlet Oxygen over Atomic Metal-N-C Catalysts. *Angew. Chem. Int. Ed.* **60**, 22513-22521 (2021).

5 Xiao, H. *et al.* N-doped biochar-Fe/Mn as a superior peroxymonosulfate activator for enhanced bisphenol a degradation. *Water Res.* **278**, 123399 (2025).

6 Huang, J. *et al.* Chlorine atoms modulated Fe d-band centers drive non-radical pathways to degrade bisphenol A with attenuated intermediates toxicity. *Chem. Eng. J.* **524**, 169699 (2025).
