# Supplementary material for: Engineering N-site isomerism in donor-acceptor covalent organic frameworks for efficient Fenton-like water purification
Source: Nat Commun. 2026 Aug 7;17:9492. doi: 10.1038/s41467-026-76345-2 (PMC13542144; doi:10.1038/s41467-026-76345-2)
Supplement: Supplementary file 2 [file 41467_2026_76345_MOESM2_ESM.pdf]

nature portfolio

Peer Review File

Engineering N-site isomerism in donor-acceptor covalent organic frameworks for efficient Fenton-like water purification

Corresponding Author: Professor Jiansheng Li

This file contains all reviewer reports in order by version, followed by all author rebuttals in order by version.

Version 0:

Reviewer comments:

Reviewer #1

(Remarks to the Author)
This manuscript reports three constitutionally isomeric donor–acceptor COFs (TF-22Bpy, TA-22Bpy, TA-33Bpy) and claims that "N-site isomeric engineering" enables PMS-triggered charge separation that drives 100% selective singlet oxygen ($^1O_2$) generation and improved pollutant (BPA) degradation kinetics (kobs up to 0.165 min^{-1}). While the concept of linkage/regioisomer control in COFs is interesting and potentially valuable for advanced oxidation processes, the current study does not meet the mechanistic rigor, structural certainty, and breadth of validation expected for a high-impact journal such as Nature Communications. In its present form, the work reads as an incremental performance comparison across three related COFs with an incomplete/over-asserted mechanistic narrative and insufficient validation under realistic operating conditions.

My major concerns are:

1. The manuscript makes strong structure–property claims based on precise positional engineering, yet the crystallographic description contains internal inconsistencies that raise concern about the reliability of the structural models (e.g., declaring "tetragonal lattices" while reporting space groups such as P1, and providing lattice parameters that appear swapped/physically inconsistent with tetragonal symmetry; assigning reflections/planes differently across samples while also claiming identical topology/AA stacking). For a paper whose core claim is regioisomerism-driven electronic regulation, the framework structure must be established with much higher confidence.I highly recommend full crystallographic validation: Rietveld refinement (not only Pawley), goodness-of-fit reporting, preferred orientation considerations, and clear stacking model justification (AA vs alternatives). I also recommend performing PDF analysis (total scattering). Please also provide the CIFs of the COFs.

2. The manuscript relies heavily on scavenger tests (NaN_3), D_2O enhancement, TEMP EPR, DMA trapping, and negative results for radical probes. These are suggestive, but not sufficient to claim exclusive $^1O_2$ production with 100% selectivity, especially in heterogeneous PMS systems where probe artifacts, adsorption, and surface-confined radicals can mislead. For example, DMPO oxidation to DMPOX can occur under multiple oxidizing conditions; NaN_3 and D_2O effects are not uniquely diagnostic; DMA can be oxidized via non-$^1O_2$ pathways under some circumstances; and probe accessibility in porous solids is a major confounder. I recommend quantitative $^1O_2$ determination with an established kinetic probe (e.g., furfuryl alcohol (FFA) kinetics) and proper controls for adsorption, light, and PMS background. Besides, I recommend conducting time-resolved $^1O_2$ phosphorescence at 1270 nm (gold-standard) or, at minimum, SOSG with careful controls and calibration. Please demonstrate ROS selectivity across multiple probes and provide a mass balance / oxidant utilization (PMS-to-oxidation efficiency).

3. The paper proposes that PMS induces spatially separated holes/electrons along donor/acceptor π-columns that simultaneously drive PMS oxidation and $SO_5^{\bullet -}$ reduction. However, the experimental evidence is largely indirect (EPR g=2.003 signal assignment; hole/electron scavenger effects) and could also be consistent with alternative interpretations such as surface oxidation/doping, formation of persistent radicals, or changes in conductivity without true spatial charge separation. The manuscript also borrows concepts from photoinduced D–A systems, but PMS activation here is not clearly distinguished from chemical oxidation/doping phenomena.Direct evidence of charge separation/transport: transient

absorption spectroscopy, time-resolved PL, photocurrent/IMPS/IMVS (even if dark, provide clear electrochemical evidence of charge carrier generation/consumption) is necessary. I recommend providing operando/ in situ spectroscopy under PMS exposure (Raman/FTIR/XPS) with time evolution to show reversible vs irreversible changes and identify the actual catalytic state. Besides, explicit quantification of PMS adsorption/activation: isothermal PMS uptake, adsorption constants, and correlation with activity is necessary.

4. A major issue for translation and high-impact claims: the manuscript reports that after reaction the imine C=N band disappears and a new amide C=O band appears (suggesting oxidation/hydrolysis/amide formation), alongside a dramatic BET surface-area loss and pore blocking/distortion. This indicates the catalyst is not stable in the reaction environment and may be chemically converted into a different material during operation. If the active state is a transformed framework, that must be acknowledged and mechanistically addressed. Multi-cycle reusability with activity retention (≥5–10 cycles), with full post-reaction characterization after each cycle is needed. I also recommend providing carbon/nitrogen mass balance and identification of framework decomposition products. It is important to demonstrate whether the transformed material is the true active species (compare fresh vs spent catalyst activity normalized to surface area / active sites).

5. The application is primarily BPA degradation under selected conditions, with limited demonstration across contaminant classes and realistic matrices. In advanced oxidation for water, a high-impact paper should show robustness against real-world confounders and broader pollutant scope, plus meaningful comparisons to state-of-the-art benchmarks. It is important to discuss the substrate scope: multiple pollutants (electron-rich vs electron-poor), pharmaceuticals, dyes, PFAS surrogate (if feasible), etc., and relate selectivity to $^1O_2$ chemistry. I recommend realistic water tests: actual secondary effluent, surface water, high NOM, high alkalinity, chloride/bromide/nitrate mixtures—plus byproduct analysis. Bromate/chlorate or halogenated byproduct assessment (especially if oxidants are used) is needed. It is also imperative to benchmark against leading metal-free catalysts/COFs and report energy/oxidant efficiency metrics.

6. kobs comparisons across materials with different adsorption capacity, morphology (sea-urchin-like vs particles), and potentially different accessible active sites can be misleading without normalization. TOC removal is modest relative to BPA disappearance, suggesting partial oxidation and/or adsorption/polymerization, which needs deeper discussion. Normalize activity to accessible surface area, active site density (N content, pyridine/imine quantification), or catalyst external surface area if internal pores are not fully accessible. Provide error bars (n≥3), statistical tests, and report catalyst loading dependence with kinetic regimes identified (mass-transfer vs kinetic control). Provide oxidant consumption kinetics and PMS utilization efficiency.

In summary, while the idea of N-site isomerism as a handle to tune COF electronic structure is interesting, the central claims (exclusive $^1O_2$, PMS-triggered spatial charge separation, and practical robustness) are not supported at the level required for Nature Communications. The manuscript would require substantial additional mechanistic and stability work, clearer and more defensible structural validation, and broader environmental relevance. I therefore recommend rejection at this time for Nature Communications.

Reviewer #2

(Remarks to the Author)
The manuscript titled “Engineering N-site isomerism in donor-acceptor covalent organic frameworks for efficient Fenton-like water purification” describes the development of a engineering strategy to modulate the electronic structures of donor-acceptor (D-A) covalent organic frameworks (COFs). The authors systematically synthesized three D-A COFs (TF-22Bpy, TA-22Bpy, and TA-33Bpy) with varying positions of imine and pyridine nitrogen atoms. The performance of these COFs in activating peroxymonosulfate (PMS) for singlet oxygen (1O2) generation and BPA degradation was evaluated. The results showed that TA-33Bpy exhibited the highest catalytic activity, with a significant enhancement in BPA removal efficiency compared to the other two COFs. The study also included theoretical calculations and experimental analyses to elucidate the mechanism of PMS activation and 1O2 generation. I think this work will be of interest to others in the field, and I recommend it for publication after the authors address the following:
1. The article employs various methods, including DFT calculations, experimental probes, and electrochemical techniques. However, some details in the methodology section could be further clarified. For instance, were all COF structures optimized in the DFT calculations? Was the solvent effect on the COF structure considered?
2. The article mentions that BPA oligomers may undergo polymerization on the catalyst surface, but the specific impact of this phenomenon on catalytic performance is not further discussed. I suggest adding a more detailed analysis of this polymerization process in the discussion section, particularly how it affects the catalyst's stability, active sites, and long-term catalytic efficiency.
3. Although the article discusses the stability of TA-33Bpy and possible issues such as pore blocking, could other countermeasures or suggestions for improvement be provided to maintain the catalyst's efficiency over extended use?
4. The N-site isomeric engineering strategy proposed in this study shows significant effects in D-A COFs. Could this strategy be extended to other types of COFs or catalytic reactions? Exploring this could further enhance the broader applicability of the research.
5. Please ensure proper chemical formula formatting, maintaining consistency and accuracy throughout.

Reviewer #3

(Remarks to the Author)

In this manuscript, the authors presented an investigation on the rational design of donor-acceptor covalent organic frameworks (D-A COFs) via N-site isomeric engineering to achieve highly efficient and selective generation of singlet oxygen (1O2) for peroxymonosulfate (PMS)-based water purification. A novel mechanism of 1O2 generation via PMS activation was proposed for the first time. PMS-induced spatially separated charges (holes and electrons) in D-A COFs can sequentially achieve the oxidation of HSO52– and the reduction of SO5•– for 1O2 generation. The central findings were well-supported by a convincing combination of detailed experiments and theoretical calculations. In general, the investigation establishes a clear structure-property-activity relationship and offers a valuable design principle for COFs-based catalysts. However, some conclusions are needed for more evidence and some details still need to be improved. Therefore, I suggest its publication in Nature Communications after all these problems are well addressed. The comments are listed below:

1.Line 91, the authors mentioned that “a PMS-triggered self-catalytic mechanism…” is confusing. While the concept of spatially separated redox sites driving the cycle is clear, the term "self-catalytic" could be misinterpreted. It might imply autocatalysis or regeneration without external reagents.
2.How are the structures of adjacent pyridine-N in TF-22Bpy and TA-22Bpy as well as pyridine-like structure in TA-33Bpy formed during the preparation of COFs?
3.Due to the presence of holes and electrons, the effect of dissolved oxygen in the system needs to be taken into account.
4.What about the consumption of PMS during the process?
5.The details of EPR and GOP tests should be provided.
6.Some of the figures in the manuscript are blurry, such as Fig. 2a-c and insert of Fig.3a.
7.There are some spelling and grammar mistakes in the text. For example, page 6, line 128, “separation and directional electron transfer between42,48” should be revised as “separation and directional electron transfer42,48”. The authors should check this manuscript carefully.

Version 1:

Reviewer comments:

Reviewer #1

(Remarks to the Author)
The authors have answered to all my comments satisfactorily. I recommend the paper for publication in Nature Communications.

Reviewer #2

(Remarks to the Author)
I am satisfied with the revision and recommend the publication.

Reviewer #3

(Remarks to the Author)
The manuscript has been revised well.

Open Access This Peer Review File is licensed under a Creative Commons Attribution 4.0 International License, which permits use, sharing, adaptation, distribution and reproduction in any medium or format, as long as you give appropriate credit to the original author(s) and the source, provide a link to the Creative Commons license, and indicate if changes were made.
In cases where reviewers are anonymous, credit should be given to 'Anonymous Referee' and the source.
The images or other third party material in this Peer Review File are included in the article's Creative Commons license, unless indicated otherwise in a credit line to the material. If material is not included in the article's Creative Commons license and your intended use is not permitted by statutory regulation or exceeds the permitted use, you will need to obtain permission directly from the copyright holder.
To view a copy of this license, visit https://creativecommons.org/licenses/by/4.0/

List of Changes and Response to Referees' Comments

(NCOMMS-26-005199)

Reviewer #1

General Comments: This manuscript reports three constitutionally isomeric donor–acceptor COFs (TF-22Bpy, TA-22Bpy, TA-33Bpy) and claims that "N-site isomeric engineering" enables PMS-triggered charge separation that drives 100% selective singlet oxygen ($^1O_2$) generation and improved pollutant (BPA) degradation kinetics (k_{obs} up to 0.165 min^{-1}). While the concept of linkage/regioisomer control in COFs is interesting and potentially valuable for advanced oxidation processes, the current study does not meet the mechanistic rigor, structural certainty, and breadth of validation expected for a high-impact journal such as Nature Communications. In its present form, the work reads as an incremental performance comparison across three related COFs with an incomplete/over-asserted mechanistic narrative and insufficient validation under realistic operating conditions.

Response: We sincerely thank Reviewer #1 for this thoughtful and constructive comments on our manuscript. The concerns regarding mechanistic rigor, structural certainty, and the breadth of validation have been carefully considered, and a series of additional experiments and analyses have been performed to substantially strengthen the manuscript. Specifically, to improve the reliability of the structural assignment, the structural models of all three COFs were reconstructed using the same space group (*Cm*), followed by Rietveld refinements to enable a more consistent and rigorous structural comparison. With respect to the proposed mechanism, additional probe experiments, in situ characterizations, and photoelectrochemical measurements were incorporated, providing stronger evidence for PMS-induced charge polarization and $^1O_2$-dominant process in COF/PMS system. To broaden the validation of catalytic applicability, the effects of water-quality parameters, different pollutant substrates, catalyst dosage, and recycling performance were further evaluated. Based on these additional results, the manuscript has been substantially revised, with the mechanistic claims moderated, the structure–activity relationship clarified, and the overall evidence chain strengthened.

Comment 1. The manuscript makes strong structure–property claims based on precise positional engineering, yet the crystallographic description contains internal inconsistencies that raise concern about the reliability of the structural models (e.g., declaring "tetragonal lattices" while reporting space groups such as P1, and providing lattice parameters that appear swapped/physically inconsistent with tetragonal symmetry; assigning reflections/planes differently across samples while also claiming identical topology/AA stacking). For a paper whose core claim is regioisomerism-driven electronic regulation, the framework structure must be established with much higher confidence. I highly recommend full crystallographic validation: Rietveld refinement (not only Pawley), goodness-of-fit reporting, preferred orientation considerations, and clear stacking model justification (AA vs alternatives). I also recommend performing PDF analysis (total scattering). Please also provide the CIFs of the COFs.

Response: We thank Reviewer #1 for the helpful comments. Following the suggestion, we have rebuilt the structural models of all three COFs under same space groups (*Cm*) and strengthened the crystallographic analysis. Specifically, the stacking model assignment has been clarified by comparing the experimental PXRD patterns with simulated patterns based on different stacking modes. Rietveld refinements were then performed for TF-22Bpy, TA-22Bpy, and TA-33Bpy, the corresponding refinement profiles, goodness-of-fit parameters, and crystallographic information have been added to the revised manuscript and Supporting Information. Previous inconsistencies in the lattice description, space-group notation, lattice parameters, and peak indexing have also been corrected. In addition, the CIF files of the three COFs have been provided with the revised submission. Relevant revisions are added as follows:

Page 8, line 158,

"Powder X-ray diffraction (PXRD) patterns verified the high crystallinity of the three isomeric COFs (Fig. 2a-c). To determine the stacking mode with higher confidence, we compared the experimental PXRD data with simulated patterns based on inclined AA-, AB-, and ABC-stacking models. In all three cases, the inclined AA-stacking model reproduced the experimental diffraction features most satisfactorily, whereas the AB- and ABC-stacking models showed obvious mismatches in peak positions and relative intensities (Fig. S2-4). These results indicate that TF-22Bpy, TA-22Bpy, and TA-33Bpy adopt an inclined AA-stacking mode despite their distinct regioisomeric configurations.

Based on these validated stacking models, Rietveld refinements were performed using monoclinic structural models to yield good agreement between the observed and calculated PXRD profiles. All three COFs were satisfactorily refined in the monoclinic CM space group, with low residual factors and reasonable GOF values, confirming the reliability of the structural models. The refined lattice parameters further reveal subtle isomer-dependent variations in framework geometry, while preserving the overall topology and inclined AA stacking."

Page 9, line 203,

"Despite these morphological differences, high-resolution TEM images of all isomeric COFs display well-defined lattice fringes with interplanar spacing of about 1.83 nm (Fig. 2g-h), which corresponds to the (020) plane in COFs, consistent with the PXRD results."

Supporting Information, Page 8-10,

"

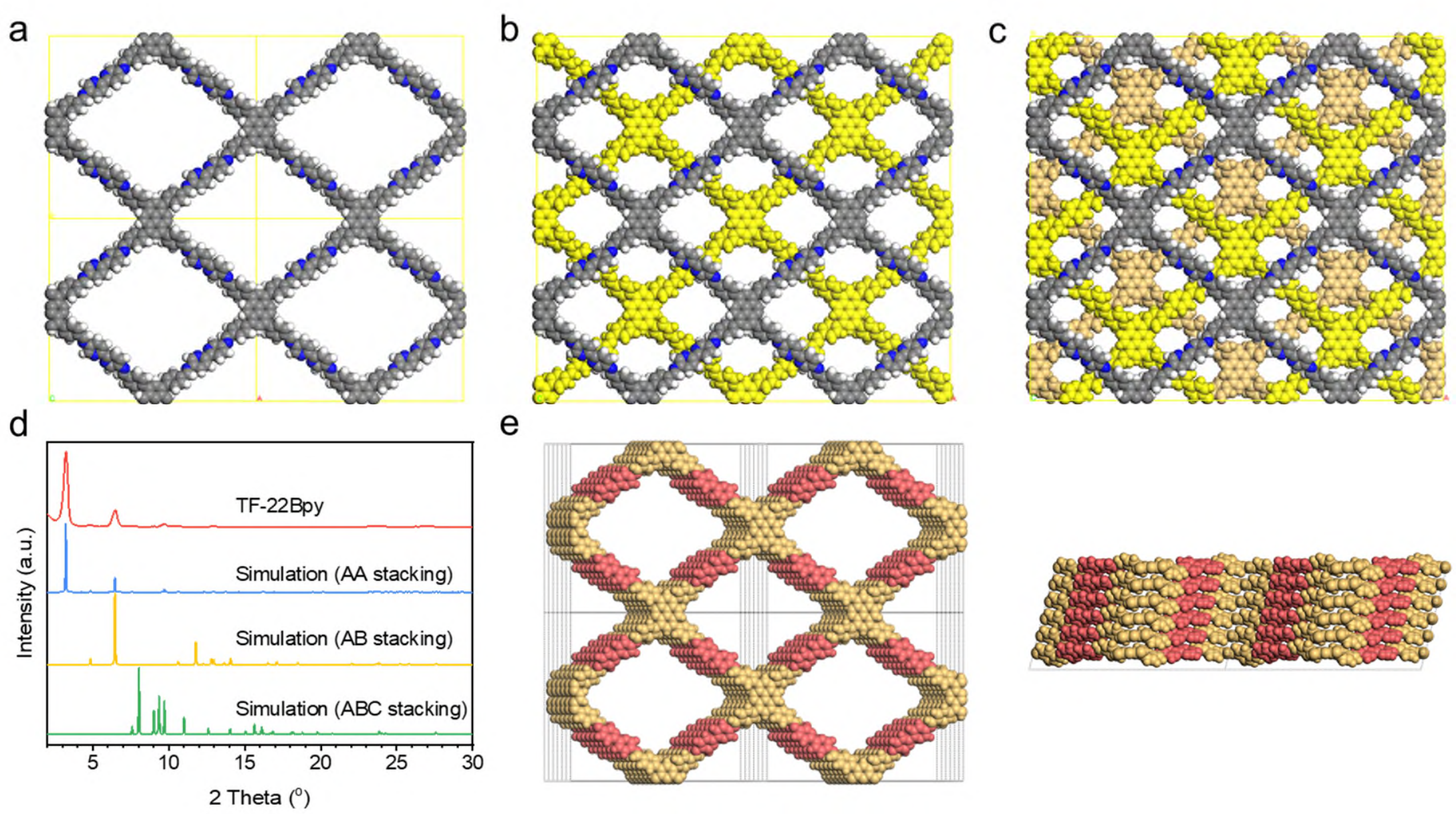

Fig. S2 Theoretically modelled inclined (a) AA stacking, (b) AB stacking, (b) ABC stacking models, and (d) corresponding simulated XRD patterns of TF-22Bpy. (e) Top view (left) and side view (right) of the inclined AA stacking model.

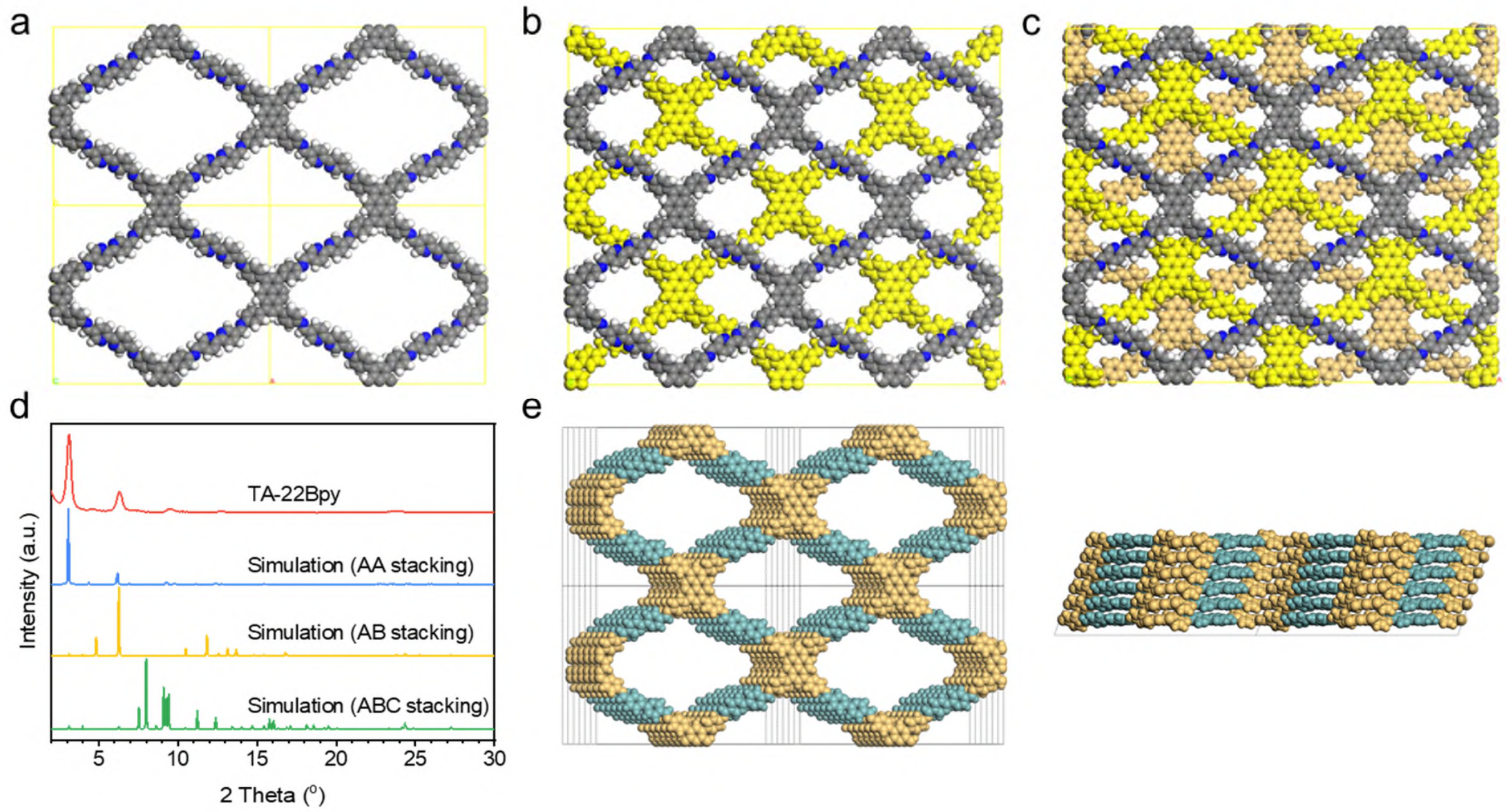

Fig. S3 Theoretically modelled inclined (a) AA stacking, (b) AB stacking, (b) ABC stacking models, and (d) corresponding simulated XRD patterns of TA-22Bpy. (e) Top view (left) and side view (right) of the inclined AA stacking model.

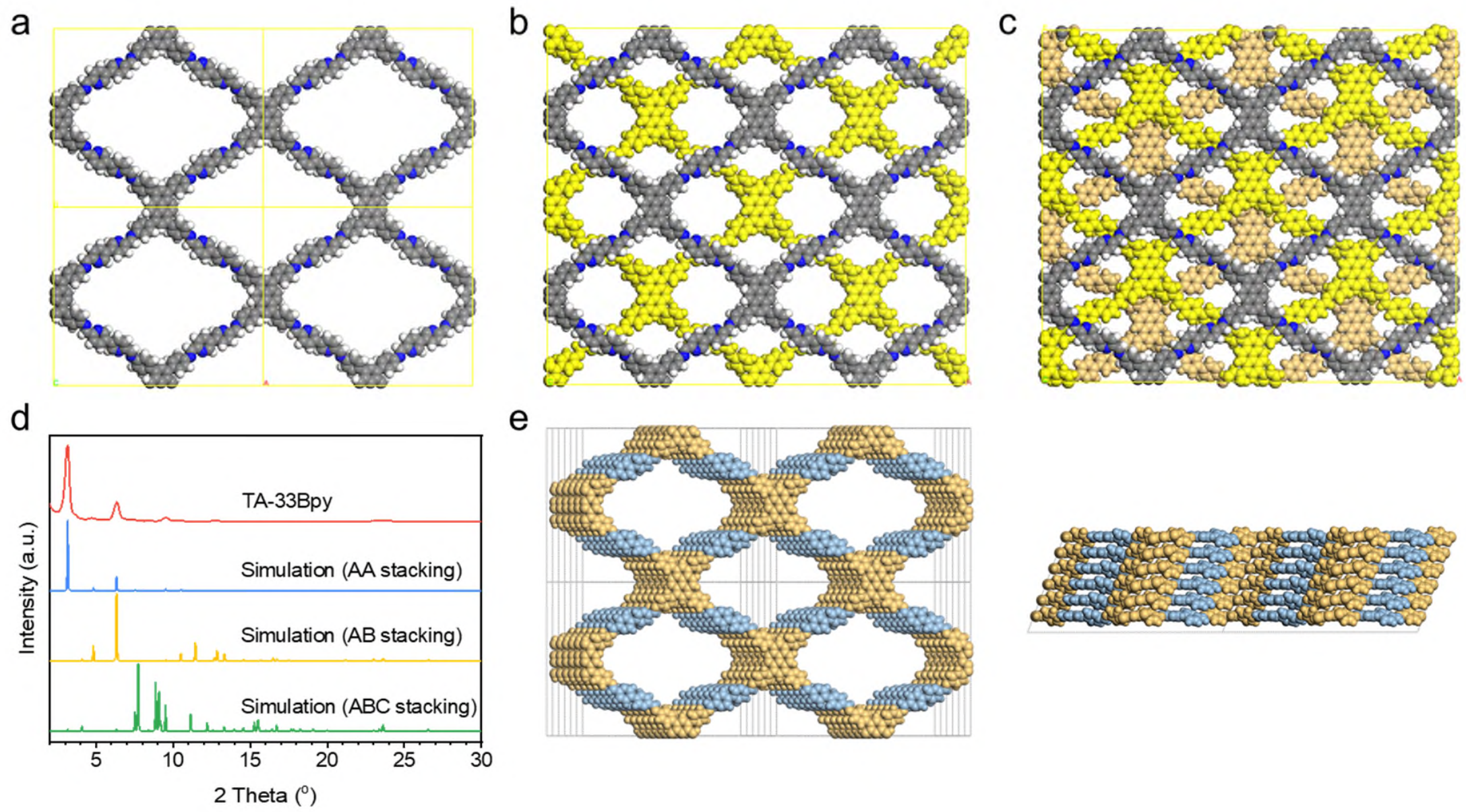

Fig. S4 Theoretically modelled inclined (a) AA stacking, (b) AB stacking, (b) ABC stacking models, and (d) corresponding simulated XRD patterns of TA-33Bpy. (e) Top view (left) and side view (right) of the inclined AA stacking model."

Supporting Information, Page 66-74,

Table S1 Rietveld refinement result of TF-22Bpy.

Name	TF-22Bpy
Chemical formula	$C_{128}H_{76}N_{16}$
Crystal system	Monoclinic
Unit cell	a = 42.620(23) Å, b =36.440(5) Å,<br>c = 3.846(8) Å<br>α = γ = 90.0°, β = 104.270(8)°
Space group	CM
Volume/Å^3	5789
X-ray source	Cu Kα
Wavelength/Å	1.5418
2θ	2-30°
Rp	4.25%
Rwp	7.54%
GOF	1.37
Z	4
Number of data points	1401

Table S2 Fractional atomic coordinates and the unit cell of TF-22Bpy from the Rietveld refinement.

TF-22Bpy		Space group: CM<br>a = 42.620(23) Å, b =36.440(5) Å,<br>c = 3.846(8) Å<br>$\alpha = \gamma = 90.0^o$, $\beta = 104.270(8)^o$		
Name	Atom type	x	y	z
C1	C	0.06566	-0.51764	-0.08522
C2	C	0.03889	-0.46395	-0.03929
C3	C	0.00969	-0.48192	-0.03049
C4	C	-0.0194	-0.46389	-0.0198
C5	C	-0.04633	-0.48233	0.02322
C6	C	-0.0201	-0.57211	-0.04004
C7	C	0.01013	-0.58928	-0.0238
C8	C	0.04008	-0.57197	-0.01159
C9	C	0.07255	-0.59216	0.04598
C10	C	-0.05204	-0.59271	-0.08801
C11	C	0.0695	-0.62229	-0.1563
C12	C	0.09992	-0.64125	-0.0998
C13	C	0.13402	-0.63056	0.16203
C14	C	0.13707	-0.6011	0.37242
C15	C	0.10662	-0.58238	0.31887
C16	C	-0.08654	-0.58394	-0.36015
C17	C	-0.1163	-0.60364	-0.40853
C18	C	-0.11206	-0.63302	-0.19299
C19	C	-0.07753	-0.64247	0.06994
C20	C	-0.0479	-0.62261	0.12077
C21	C	-0.14373	-0.65327	-0.24095

N22	N	-0.14006	-0.67992	-0.03348
C23	C	-0.16789	-0.70182	-0.03672
C24	C	-0.20466	-0.69483	-0.24038
N25	N	-0.22938	-0.71636	-0.2199
C26	C	-0.22015	-0.74524	-0.00848
C27	C	-0.18355	-0.75283	0.20054
C28	C	-0.15774	-0.73103	0.18469
C29	C	0.16671	-0.64908	0.21179
N30	N	0.16487	-0.32392	0.01514
C31	C	0.19499	-0.69485	0.02529
C32	C	0.18967	-0.72801	-0.11458
N33	N	0.21731	-0.74611	-0.12087
C34	C	0.25101	-0.73308	0.00441
C35	C	0.25717	-0.69938	0.13753
C36	C	0.2292	-0.68042	0.14795
H37	H	0.08651	-0.53035	-0.12984
H38	H	-0.06717	-0.46961	0.06828
H39	H	0.0104	-0.61675	-0.02019
H40	H	0.04368	-0.63091	-0.36284
H41	H	0.09698	-0.66388	-0.26614
H42	H	0.16303	-0.59269	0.58061
H43	H	0.10965	-0.56026	0.49208
H44	H	-0.09038	-0.56204	-0.53884
H45	H	-0.14261	-0.59619	-0.61787
H46	H	-0.07358	-0.665	0.24026
H47	H	-0.02175	-0.63051	0.32657
H48	H	-0.17462	-0.77453	0.38517

H49	H	-0.12958	-0.73644	0.35039
H50	H	0.16339	-0.7394	-0.21785
H51	H	0.28292	-0.6873	0.22488
H52	H	-0.214	-0.67243	-0.41265
H53	H	0.23427	-0.65436	0.24198
H54	H	0.19228	-0.63957	0.41638
H55	H	-0.16973	-0.64538	-0.45111

Table S3 Rietveld refinement result of TA-22Bpy.

Name	TA-22Bpy
Chemical formula	$C_{128}H_{76}N_{16}$
Crystal system	Monoclinic
Unit cell	a = 47.000(9) Å, b =36.470(8) Å,<br>c = 3.880(9) Å<br>α = γ = 90.0°, β = 109.600(23)°
Space group	CM
Volume/$Å^3$	6265
X-ray source	Cu Kα
Wavelength/Å	1.5418
2θ	2-30°
Rp	4.12%
Rwp	8.96%
GOF	1.29
Z	4
Number of data points	1401

Table S4 Fractional atomic coordinates and the unit cell of TA-22Bpy from the Rietveld refinement.

TA-22Bpy		Space group: CM<br>a = 47.000(9) Å, b =36.470(8) Å,<br>c = 3.880(9) Å<br>α = γ = 90.0º, β = 109.600(23)º		
Name	Atom type	x	y	z
C1	C	0.06146	-0.51977	-0.10642
C2	C	0.03701	-0.45959	-0.0464
C3	C	0.01006	-0.47973	-0.02702
C4	C	-0.01673	-0.45948	-0.00493
C5	C	-0.04153	-0.48018	0.04854
C6	C	-0.01728	-0.58092	-0.02322
C7	C	0.01076	-0.60011	-0.01536
C8	C	0.03833	-0.58066	-0.01516
C9	C	0.06841	-0.60318	0.03088
C10	C	-0.04663	-0.60416	-0.05632
C11	C	0.0656	-0.63715	-0.16942
C12	C	0.09387	-0.65752	-0.13434
C13	C	0.12556	-0.64446	0.10599
C14	C	0.12834	-0.61156	0.31599
C15	C	0.10011	-0.59148	0.28515
C16	C	-0.07885	-0.59435	-0.31253
C17	C	-0.10614	-0.61634	-0.34049
C18	C	-0.10181	-0.6491	-0.1236
C19	C	-0.06954	-0.65969	0.12405
C20	C	-0.04235	-0.63764	0.1542
N21	N	-0.13072	-0.66927	-0.14367

C22	C	-0.13101	-0.70358	-0.02046
C23	C	-0.1617	-0.72049	-0.02771
C24	C	-0.19195	-0.70083	-0.174
N25	N	-0.21948	-0.71676	-0.16244
C26	C	-0.21921	-0.75194	-0.01406
C27	C	-0.1892	-0.77242	0.13498
C28	C	-0.16066	-0.75667	0.1261
N29	N	0.15542	-0.66151	0.11862
C30	C	0.15852	-0.30429	0.01083
C31	C	0.19035	-0.70821	0.0127
C32	C	0.195	-0.7463	-0.05974
N33	N	0.22406	-0.75762	-0.06673
C34	C	0.24968	-0.73312	-0.00489
C35	C	0.24565	-0.69447	0.06325
C36	C	0.2161	-0.68227	0.07344
H37	H	0.08047	-0.53401	-0.16293
H38	H	-0.06061	-0.46592	0.10392
H39	H	0.01117	-0.6309	-0.00925
H40	H	0.04164	-0.64732	-0.36274
H41	H	0.09087	-0.68216	-0.30945
H42	H	0.15265	-0.60165	0.50472
H43	H	0.10306	-0.56657	0.45768
H44	H	-0.08285	-0.56988	-0.49328
H45	H	-0.13085	-0.60826	-0.53492
H46	H	-0.06514	-0.68392	0.307
H47	H	-0.01798	-0.6464	0.34951
H48	H	-0.10864	-0.72048	0.09501

H49	H	-0.18718	-0.79972	0.26866
H50	H	-0.1376	-0.77241	0.24601
H51	H	0.13796	-0.2845	-0.08174
H52	H	0.17559	-0.76708	-0.10982
H53	H	0.26456	-0.67351	0.10079
H54	H	-0.19364	-0.67284	-0.29568
H55	H	0.21307	-0.65246	0.12335

Table S5 Rietveld refinement result of TA-33Bpy.

Name	TA-33Bpy
Chemical formula	$C_{128}H_{76}N_{16}$
Crystal system	Monoclinic
Unit cell	a = 46.100(23) Å, b =36.570(4) Å,<br>c = 4.025(26) Å<br>α = γ = 90.0º, β = 110.700(6)º
Space group	CM
Volume/$Å^3$	6438
X-ray source	Cu Kα
Wavelength/Å	1.5418
2θ	2-30º
Rp	3.44%
Rwp	6.32%
GOF	1.44
Z	4
Number of data points	1401

Table S6 Fractional atomic coordinates and the unit cell of TA-33Bpy from the Rietveld refinement.

TA-33Bpy		Space group: CM<br>a = 46.100(23) Å, b =36.570(4) Å,<br>c = 4.025(26) Å<br>α = γ = 90.0º, β = 110.700(6)º		
Name	Atom type	x	y	z
C1	C	0.06278	-0.5192	-0.14931
C2	C	0.03848	-0.46074	-0.07062
C3	C	0.01195	-0.48032	-0.02679
C4	C	-0.01451	-0.4607	0.01805
C5	C	-0.03896	-0.48078	0.09396
C6	C	-0.01523	-0.57847	-0.00272
C7	C	0.01221	-0.59717	-0.02231
C8	C	0.03949	-0.57838	-0.04479
C9	C	0.06887	-0.60053	-0.02734
C10	C	-0.04434	-0.60084	-0.01481
C11	C	0.06585	-0.63363	-0.22437
C12	C	0.09327	-0.6546	-0.20464
C13	C	0.12431	-0.64296	0.01544
C14	C	0.12739	-0.61044	0.21609
C15	C	0.09995	-0.58979	0.20155
C16	C	-0.07556	-0.5909	-0.24564
C17	C	-0.10266	-0.61211	-0.25777
C18	C	-0.09921	-0.64411	-0.04832
C19	C	-0.06786	-0.65507	0.17158
C20	C	-0.04082	-0.63366	0.18684
N21	N	-0.12816	-0.66391	-0.06364

C22	C	-0.12996	-0.69374	0.11396
C23	C	-0.16167	-0.71038	0.07648
N24	N	-0.18974	-0.69363	-0.12831
C25	C	-0.21941	-0.70799	-0.1647
C26	C	-0.22204	-0.74117	0.01049
C27	C	-0.19326	-0.75889	0.22123
C28	C	-0.16312	-0.74359	0.25374
N29	N	0.15328	-0.66215	0.02486
C30	C	0.15516	-0.30291	-0.04267
C31	C	0.18623	-0.71334	-0.03552
C32	C	0.1898	-0.75177	-0.04306
C33	C	0.21956	-0.76674	-0.03178
C34	C	0.24569	-0.74339	-0.01665
C35	C	0.24073	-0.70497	-0.01745
N36	N	0.21165	-0.69111	-0.02454
H37	H	0.08166	-0.53302	-0.22075
H38	H	-0.05788	-0.46696	0.16478
H39	H	0.01236	-0.62706	-0.01996
H40	H	0.04236	-0.64293	-0.40015
H41	H	0.09026	-0.67894	-0.37254
H42	H	0.15124	-0.60163	0.38906
H43	H	0.103	-0.56546	0.37033
H44	H	-0.07894	-0.56692	-0.41947
H45	H	-0.1266	-0.604	-0.43422
H46	H	-0.06387	-0.67952	0.33793
H47	H	-0.01715	-0.64256	0.36131
H48	H	-0.10848	-0.70688	0.29572

H49	H	-0.24085	-0.69368	-0.33414
H50	H	-0.194	-0.78384	0.37078
H51	H	-0.14111	-0.75719	0.42087
H52	H	0.13424	-0.28503	-0.09433
H53	H	0.16991	-0.76995	-0.05102
H54	H	0.22231	-0.7965	-0.026
H55	H	0.25986	-0.68591	-0.0149

Comment 2. The manuscript relies heavily on scavenger tests (NaN_3), D_2O enhancement, TEMP EPR, DMA trapping, and negative results for radical probes. These are suggestive, but not sufficient to claim exclusive $^1O_2$ production with 100% selectivity, especially in heterogeneous PMS systems where probe artifacts, adsorption, and surface-confined radicals can mislead. For example, DMPO oxidation to DMPOX can occur under multiple oxidizing conditions; NaN_3 and D_2O effects are not uniquely diagnostic; DMA can be oxidized via non-$^1O_2$ pathways under some circumstances; and probe accessibility in porous solids is a major confounder. I recommend quantitative $^1O_2$ determination with an established kinetic probe (e.g., furfuryl alcohol (FFA) kinetics) and proper controls for adsorption, light, and PMS background. Besides, I recommend conducting time-resolved $^1O_2$ phosphorescence at 1270 nm (gold-standard) or, at minimum, SOSG with careful controls and calibration. Please demonstrate ROS selectivity across multiple probes and provide a mass balance / oxidant utilization (PMS-to-oxidation efficiency).

Response: We appreciate the valuable comments from Reviewer #1. Following the suggestion, we revised the manuscript to avoid overstating "100% selectivity", and described the ROS pathway more cautiously as a $^1O_2$-dominated process. To strengthen the ROS identification, we performed additional FFA kinetic probe tests and SOSG measurements with appropriate controls. In addition, PMS consumption, $^1O_2$ yield, and oxidant-utilization/activation-efficiency analyses have been added to provide a more quantitative basis for evaluating ROS selectivity and oxidant use. Relevant revisions are added as follows:

Page 13, line 297,

"To quantitatively verify $^1O_2$ generation in the heterogeneous PMS system, we further conducted PMS consumption, ABDA-based $^1O_2$ quantification, and FFA kinetic measurements. Within 20 min, the PMS consumption reached 50.2%, 23.8%, and 16.4% for TA-33Bpy, TA-22Bpy, and TF-22Bpy, respectively (Fig. S20). Consistently, ABDA quantification confirmed $^1O_2$ production of 0.122, 0.104, and 0.082 mM, respectively (Fig. S21a). The corresponding PMS utilization efficiencies were calculated to be 37.25%, 67.35%, and 76.86%, indicating that the slower-reacting system used PMS more efficiently (Fig. S21b). FFA kinetics further showed that the steady-state concentration (C_{ss}) of $^1O_2$ followed an order of TA-33Bpy (1.60×10^{-12}M) > TA-22Bpy (1.00×10^{-12}M) > TF-22Bpy (0.82×10^{-12}M), in good agreement with the ABDA results (Fig. S21c)[54,55]. Notably, the opposite

trends in $^1O_2$ productivity and PMS utilization efficiency suggest that more efficient oxidant use does not necessarily lead to higher apparent activity."

Page 14, line 332,

"These EPR findings were further supported by chemical trapping using 9,10-dimethylanthracene (DMA) and Singlet Oxygen Sensor Green (SOSG), two selective probes for $^1O_2$. UV-vis spectra showed a clear decrease in the DMA adsorption peak, and high-resolution mass spectrometry (HR-MS) identified the corresponding oxidation product, DMA-O_2 (Fig. 3h and S24)[20]. Moreover, a clear fluorescence signal of SOSG-EP was observed in Fig. 3i, showing an increased intensity with reaction time. Together, these results provide direct and complementary evidence that $^1O_2$ is the predominant ROSs in the TA-33Bpy/PMS system."

Page 15,

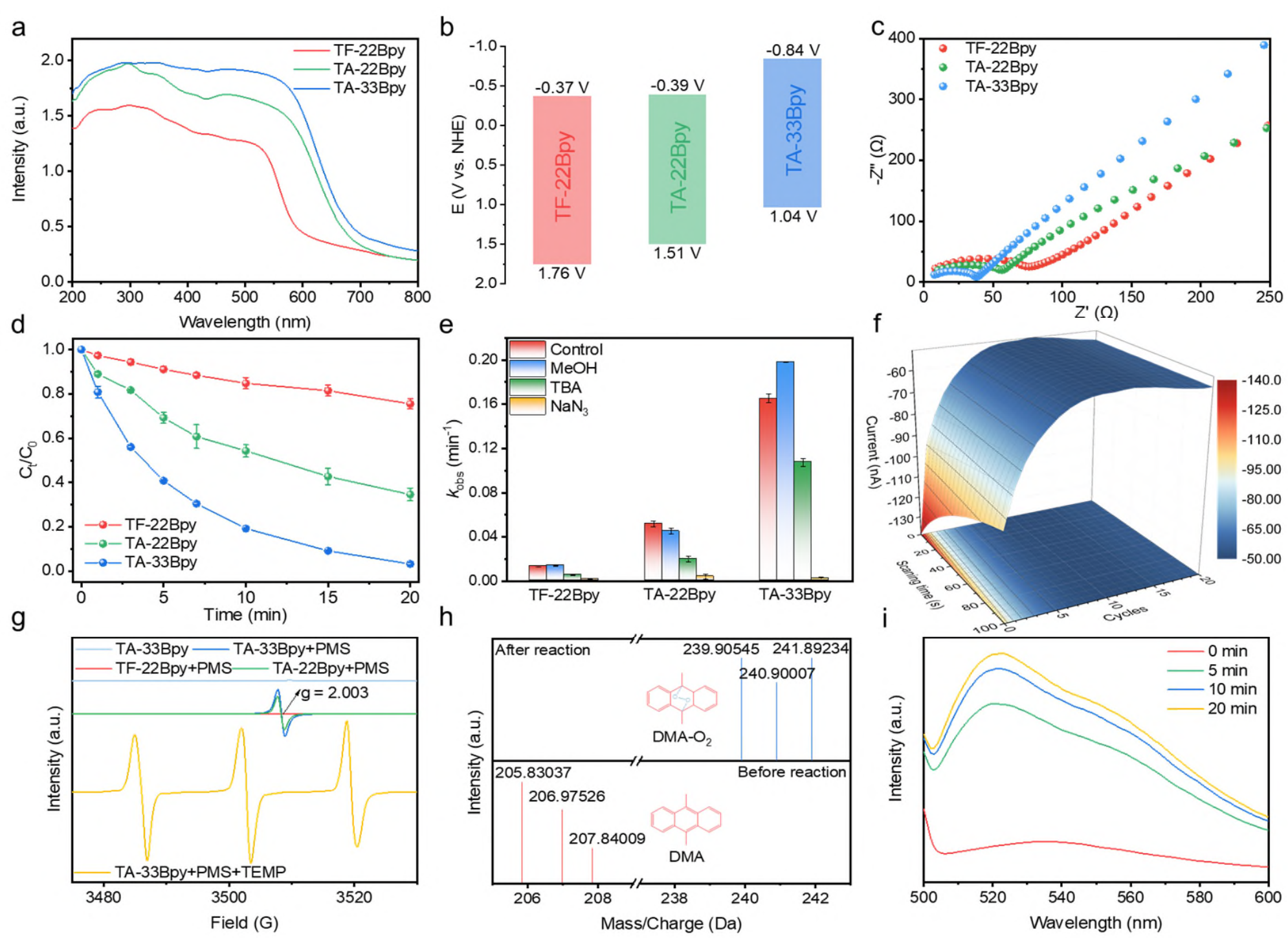

Fig. 3 Physicochemical properties and Fenton-like performance. (a) UV–vis–NIR diffuse reflectance spectra, (b) electronic band structures, and (c) electrochemical impedance spectra of the isomeric COFs. (d) BPA removal and (e) quenching experiments of all COFs/PMS system. (f) Scanning electrochemical microscopic analysis of TA-33Bpy/PMS systems. (g) EPR spectra of TA-33Bpy/PMS system using TEMP as trapping agents. (h) HR-MS chromatogram of typical DMA-O_2 from DMA oxidation in TA-33Bpy/PMS system. (i) Fluorescence spectra of SOSG-EP in TA-33Bpy/PMS system.

Page 29, line 764,

“54 Yun, E.-T., Lee, J. H., Kim, J., Park, H.-D. & Lee, J. Identifying the Nonradical Mechanism in the Peroxymonosulfate Activation Process: Singlet Oxygenation Versus Mediated Electron Transfer. *Environ. Sci. Technol.* **52**, 7032-7042 (2018).

55 Song, S.-Y. *et al.* Unveiling the unforeseen role of manganese constituent in creating a carbon-based composite as a high-efficiency persulfate activator: Catalyzing carbon phase graphitization and promoting persulfate binding affinity. *Appl. Catal. B Environ. Energy* **378**, 125598 (2025).”

Supporting Information, Page 26-27,

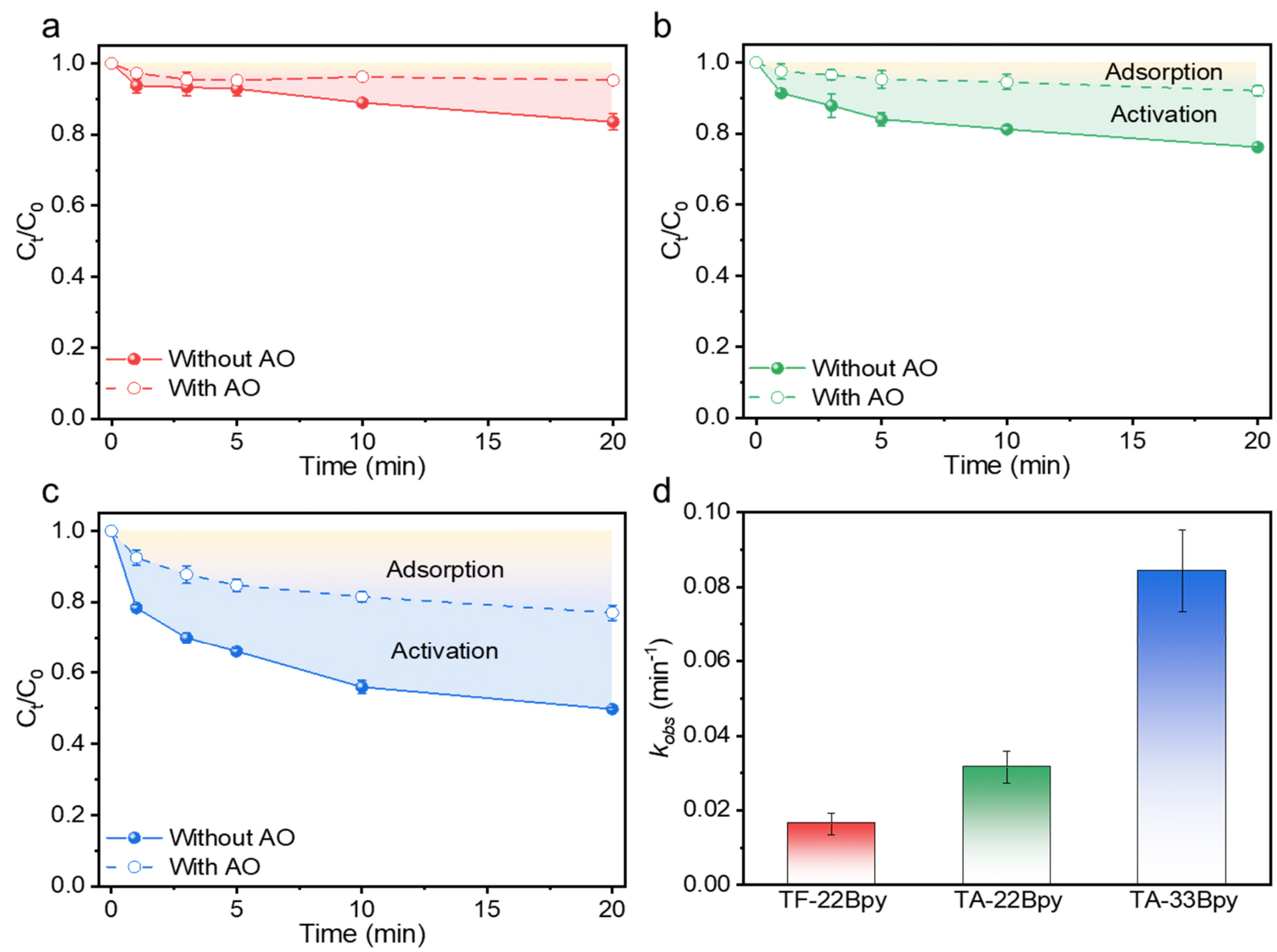

Fig. S20 The consumption and adsorption of PMS by (a) TF-22Bpy, (b) TA-22Bpy and (c) TA-33Bpy. (d) PMS consumption kinetics

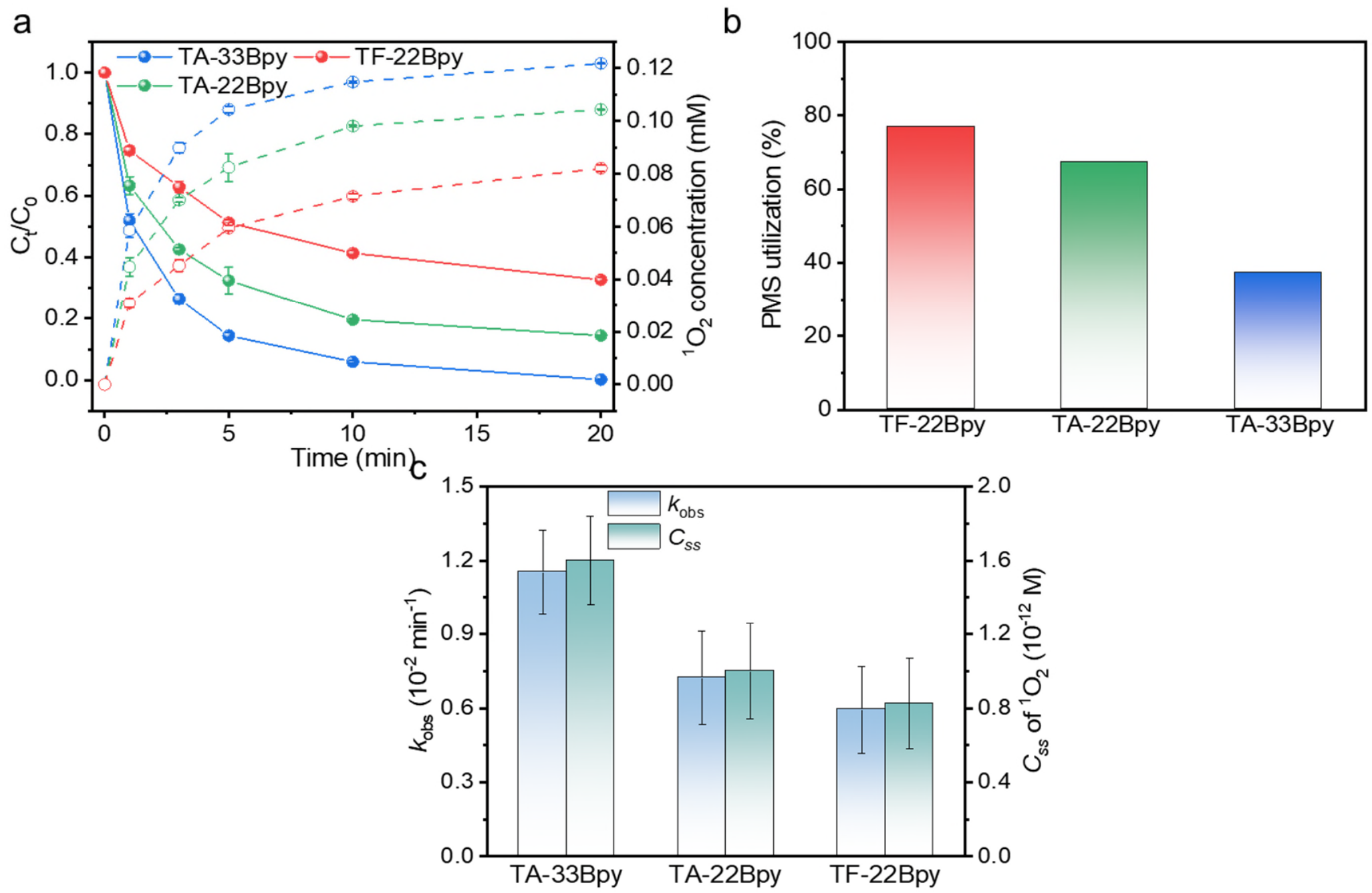

Fig. S21 (a) The consumption of ABDA and generation of $^1O_2$ in COFs/PMS system. (b) Efficiency of PMS to $^1O_2$. (c) The k_{obs} of FFA probes and steady-state concentration of $^1O_2$.

Comment 3. The paper proposes that PMS induces spatially separated holes/electrons along donor/acceptor π-columns that simultaneously drive PMS oxidation and $SO_5^{\bullet-}$ reduction. However, the experimental evidence is largely indirect (EPR g=2.003 signal assignment; hole/electron scavenger effects) and could also be consistent with alternative interpretations such as surface oxidation/doping, formation of persistent radicals, or changes in conductivity without true spatial charge separation. The manuscript also borrows concepts from photoinduced D–A systems, but PMS activation here is not clearly distinguished from chemical oxidation/doping phenomena. Direct evidence of charge separation/transport: transient absorption spectroscopy, time-resolved PL, photocurrent/IMPS/IMVS (even if dark, provide clear electrochemical evidence of charge carrier generation/consumption) is necessary. I recommend providing operando/ in situ spectroscopy under PMS exposure (Raman/FTIR/XPS) with time evolution to show reversible vs irreversible changes and identify the actual catalytic state. Besides, explicit quantification of PMS adsorption/activation: isothermal PMS uptake, adsorption constants, and correlation with activity is necessary.

Response: We appreciate the insightful comments from Reviewer #1. Following the comments, we revised the mechanistic description to a PMS-triggered charge polarization process, so as to distinguish it from the charge separation described in conventional photocatalytic systems. Moreover, a series of experiments on charge redistribution, catalytic-state evolution, and PMS adsorption/activation were conducted. Specifically, transient absorption spectroscopy, femtosecond fluorescence lifetime measurements, and photocurrent tests were provided to investigate the ground-state charge polarization and interfacial electron transfer; in situ FTIR and Raman measurements under PMS exposure were performed to monitor the evolution of the PMS–COF interfacial state during reaction; post-reaction XPS spectra of samples collected after different catalytic cycles were analyzed to evaluate possible irreversible surface oxidation or doping-like effects; PMS adsorption capacity and adsorption isotherm measurements were included to quantify PMS uptake and correlate PMS affinity with catalytic activity. Relevant revisions are added as follows:

Page 2, line 24,

"Mechanistic investigations indicate that direct PMS-COF interaction induces charge polarization within the donor-acceptor framework, generating localized electron-deficient and electron-enriched domains that promote the coupled redox steps required for selective $^1O_2$ generation.

This work identifies PMS-triggered charge polarization as a key determinant of PMS activation and offers a design principle for high-performance COF catalysts for water purification."

Page 5, line 92,

"More importantly, we elucidate for the first time a PMS-triggered charge polarization mechanism, in which direct PMS–COF interaction induces electronic redistribution across the donor (D^+) and acceptor (A^-) units, thereby cooperatively promoting PMS oxidation and $SO_5^{\bullet-}$ reduction toward predominant $^1O_2$ generation. This work not only demonstrates the critical role of PMS-triggered charge polarization on PMS activation for $^1O_2$ generation but also establishes a new design paradigm for developing high-performance COFs, advancing the development of catalysts toward efficient Fenton-like water purification."

Page 6, line 132,

"This clear spatial separation of frontier orbitals is the characteristic of a donor-acceptor architecture and is intrinsically favorable for intermolecular charge polarization and electron transfer[42,49]."

Page 11, line 231,

"A smaller band gap generally favors charge polarization and transfer."

Page 15-16,

"The interaction between PMS and COF catalysts was first examined by EPR method. Notably, a strong EPR signal at $g = 2.003$ was observed only when TA-33Bpy was brought into contact with PMS, whereas no signal was detected for pure PMS or TA-33Bpy suspension alone (Fig. 3g), indicating the generation of abundant unpaired electrons in the TA-33Bpy/PMS system. Similar EPR responses have been reported for chemically oxidized COFs treated with electrophilic oxidants such as iodine, where the oxidant acts as an electron acceptor and induces electron delocalization within the conjugated framework[56,57]. Considering the electrophilic nature of PMS, we therefore attribute the EPR signal to a PMS-triggered ground-state charge polarization within the donor-acceptor COF. Specifically, PMS is proposed to interact with the COF as an electron acceptor, withdrawing electron density from the conjugated skeleton and generating localized electron-deficient donor domains (D^+) together with electron-enriched PMS-associated acceptor domains (A^-). Consistent with this interpretation, the EPR intensity followed the order TA-33Bpy/PMS > TA-22Bpy/PMS > TF-

22Bpy/PMS under identical conditions, indicating progressively stronger PMS-COF electronic interaction from TF-22Bpy to TA-33Bpy. Moreover, the signal intensity increased significantly with increasing COF dosage but remained nearly unchanged with increasing PMS concentration (Fig. S25 and S26), confirming that the unpaired electrons mainly originated from the COF framework rather than from PMS itself.

To further probe the electronic consequence of PMS binding, we performed time-resolved photoluminescence (TR-PL) decay plots, transient absorption spectroscopy (TAS) and photocurrent tests in the absence and presence of PMS. Although these techniques are frequently used in photocatalytic systems, here they serve as probes of interfacial electron transfer and carrier extraction induced by PMS. Upon PMS addition, both the amplitude-averaged and intensity-averaged fluorescence lifetimes were prolonged, and the contribution of the long-lived decay component increased markedly, indicating substantial perturbation of the excited-state relaxation pathway (Fig. S27). Consistently, femtosecond TAS revealed pronounced changes in the decay kinetics after PMS introduction, whereas the photocurrent was markedly suppressed with little change in the electrochemical impedance (Fig. S28 and S29). This combination indicates that PMS efficiently extracts electrons from the COF framework, rather than simply altering its conductivity. These results therefore support the role of PMS as an electron acceptor upon association with the COF."

Page 17, line 382,

"To further explore the role of PMS-induced charge polarization in the process, we conducted quenching experiments using ammonium oxalate (AO) and EDTA-2Na as scavengers of electron-deficient sites, and $K_2Cr_2O_7$ as a scavenger of electron-enriched sites."

Page 17, line 387,

"These results confirm that both electron-deficient donor (D^+) and electron-rich acceptor (A^-) regions actively participate in PMS activation for BPA degradation. Based on the above results, to separate PMS adsorption from PMS activation, we used EDTA-2Na to suppress PMS conversion, allowing the intrinsic PMS uptake by the COF frameworks to be evaluated. The PMS adsorption capacity was determined to be 23%, 8%, and 4.75% for TA-33Bpy, TA-22Bpy, and TF-22Bpy, respectively (Fig. S20a-c). Moreover, the adsorption isotherms were well described by the Langmuir model, affording q_m values of 7868, 7188, and 7369 mg g^{-1}, and K_L values of 0.466, 0.366, and 0.247

L g^{-1}, respectively (Fig. S30). Importantly, the K_L values show the same trend as the catalytic rates, with k_{obs} decreasing from 0.165 to 0.052 and 0.013 min^{-1} for TA-33Bpy, TA-22Bpy, and TF-22Bpy, respectively. This positive correlation indicates that stronger PMS affinity favors interfacial oxidant enrichment and contributes to the enhanced catalytic activity. Control experiments under N_2 bubbling showed nearly unchanged catalytic activity, suggesting that dissolved oxygen (DO) makes a negligible contribution to $^1O_2$ generation. The generation of these charge carriers was directly probed using 2,2,6,6-tetramethylpiperidine-1-oxyl (TEMPO) as a spin-trapping agent. TEMPO exhibits a characteristic triplet EPR signal, which diminishes upon reaction with polarized charges to form the spin-silent adduct[59,60]"

Page 17, line 406,

"Together, these results support a mechanism in which PMS first associates with the COF framework, then induces interfacial charge polarization, and finally undergoes selective activation toward $^1O_2$ generation."

Page 18, line 413,

"Electron density differences (EDDs) analysis revealed net charge transfer from COF framework to PMS^1 (Fig. 4b, S32-S34). Notably, for TA-22Bpy and TA-33Bpy, the pyrene (donor) units also exhibited significant electron depletion, indicating that PMS induces framework-wide electronic polarization across the entire D-A framework. Further analysis of the frontier molecular orbitals in the COFs/PMS^1 systems provided deeper insights into this polarized electronic state. In TA-33Bpy/PMS^1, the HOMO remained localized on the pyrene unit and PMS-associated region, while the LUMO resided on the bipyridine linker, preserving a well-defined donor-acceptor orbital arrangement that is favorable for PMS-induced charge polarization (Fig. 4c)."

Page 18, line 426,

"This stronger orbital overlap is less favorable for maintaining a polarized interfacial electronic state, consistent with the weaker EPR signal experimentally observed for TF-22Bpy/PMS[61].

Bader charge analysis further quantified the PMS-induced charge polarization."

Page 18, line 431,

"The larger extent of charge polarization in TA-33Bpy suggests a stronger electronic interaction with PMS and a greater driving force for interfacial electron redistribution."

Page 19, line 437,

"As shown in Fig. S36, with the addition of PMS, the characteristic peaks of the pyridine ring (728 cm^{-1}) and the aromatic ring (821 and 833 cm^{-1}) shifted and their intensities changed, indicating that PMS was adsorbed around the linkage.

Next, the adsorption of the second PMS molecule (PMS^2) within the electron-deficient region of the COF/PMS^1 system and its subsequent oxidation to $SO_5^{\bullet-}$ were simulated."

Page 19, line 452

"Importantly, the PMS-induced polarized electronic state in TA-33Bpy/PMS^1 enables the coupled redox steps required for both PMS oxidation and $SO_5^{\bullet-}$ conversion, thereby promoting selective $^1O_2$ generation."

Page 19, line 456

"(i) Electrophilic PMS interacts with the donor-acceptor framework and induces charge polarization, generating localized electron-deficient (D^+) and electron-enriched (A^-) domains; (ii) Additional HSO_5^- molecules were oxidized at the electron-deficient region to form $SO_5^{\bullet-}$; (iii) $SO_5^{\bullet-}$ was reduced at the electron-enriched region to produce $^1O_2$. Structurally, the donor (pyrene) and acceptor (bipyridine) units in the TA-33Bpy are orderly arranged within the x-y plane, which is favorable for establishing PMS-induced charge polarization, while their columnar stacking along the z direction supports efficient interfacial electronic communication (Fig. 4g)[43,61,62]."

Page 20, line 465

"Within this architecture, the D^+ domains preferentially participate in HSO_5^- oxidation (Fig. 4h, green arrow), while A^- domains promote $^1O_2$ formation via $SO_5^{\bullet-}$ reduction (Fig. 4h, blue arrow).

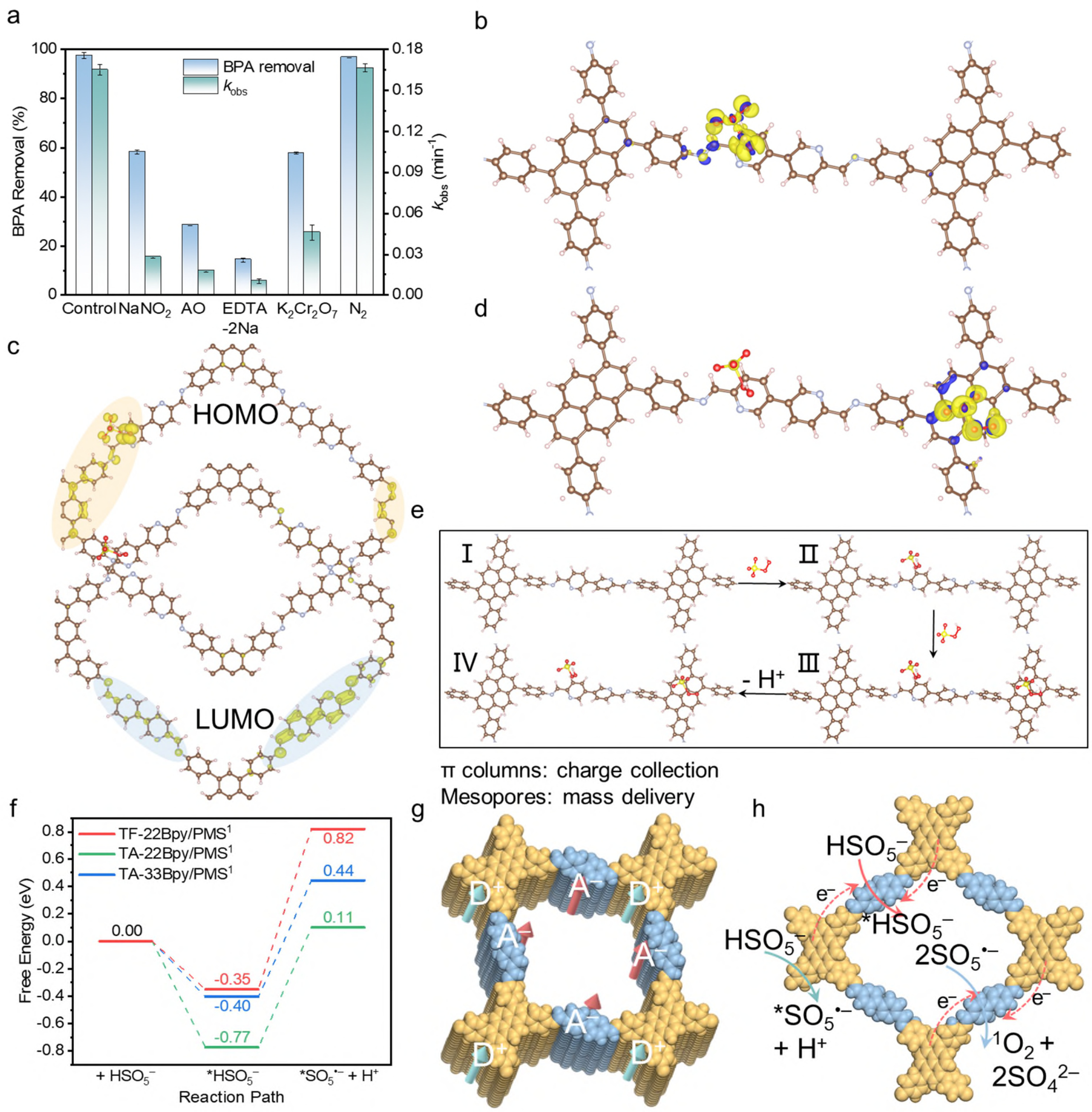

Fig. 4 Origin of $^1O_2$ generation. (a) Quenching experiments of electron-deficient sites, electron-enriched sites, $SO_5^{\bullet-}$ and DO in TA-33Bpy/PMS system. (b) EDDs of PMS[1] adsorption model on TA-33Bpy. (c) HOMO and LUMO of TA-33Bpy/PMS[1] system. (d) EDDs of PMS[2] adsorption model on TA-33Bpy/PMS[1] system. (e) The pathway and optimized atomic structures for the main process of the PMS activation on TA-33Bpy. (f) Free energy diagram for PMS[2] activation in all COFs/PMS[1] systems. (g) Ordered donor–acceptor π-columns (shown in one quadrangle of fourteen layers), illustrating the PMS-induced charge polarization within the framework, with acceptor columns (blue) associated with electron-enriched (A^-) regions and donor columns (orange) associated with electron-deficient (D^+) regions. (h) Overall reaction scheme and one-layer square TA-33Bpy COF to depict the PMS-induced charge polarization (red arrow) from the D knot to A linker; HSO_5^- oxidation

at D^+ domains (green arrow); and $SO_5^{\bullet-}$ reduction to produce $^1O_2$ at A^- regions (blue arrow)."

Page 24-25, line 586,

"By combining experimental investigations with density functional theory calculations, we reveal that direct PMS–COF interaction induces charge polarization along the donor–acceptor π-columns for the first time. This polarized electronic configuration enables coupled PMS oxidation and $SO_5^{\bullet-}$ reduction, resulting in predominant generation of singlet oxygen ($^1O_2$). This work highlights the decisive role of PMS-triggered charge polarization in mediating PMS activation and establishes, for the first time, N-site isomeric engineering as an isomerism-guided design strategy for COF-based PMS activation, with potential applicability to broader nitrogen-containing COFs and catalytic reactions."

Supporting Information, Page 3,

"**Transient photocurrent response**

Indium tin oxide (ITO) glasses, platinum plate electrode and saturated calomel electrode (SCE) were used as working electrode, assistant electrode and reference electrode, respectively. For the preparation of working electrodes, 5 mg of catalyst was dispersed into a mixture of 0.45 mL of ethanol and 0.05 mL of Nafion® solution, followed by sonication for 3 h. The suspension (50 μL) was loaded onto a glassy carbon electrode and repeated dropwise two times for preparing a COFs-coated platinum plate electrode (COFs PPE). A 300 W xenon lamp was used as the light source, and the transient photocurrent with a bias of 0 V was tested over time during the on/off light cycle."

Supporting Information, Page 26,

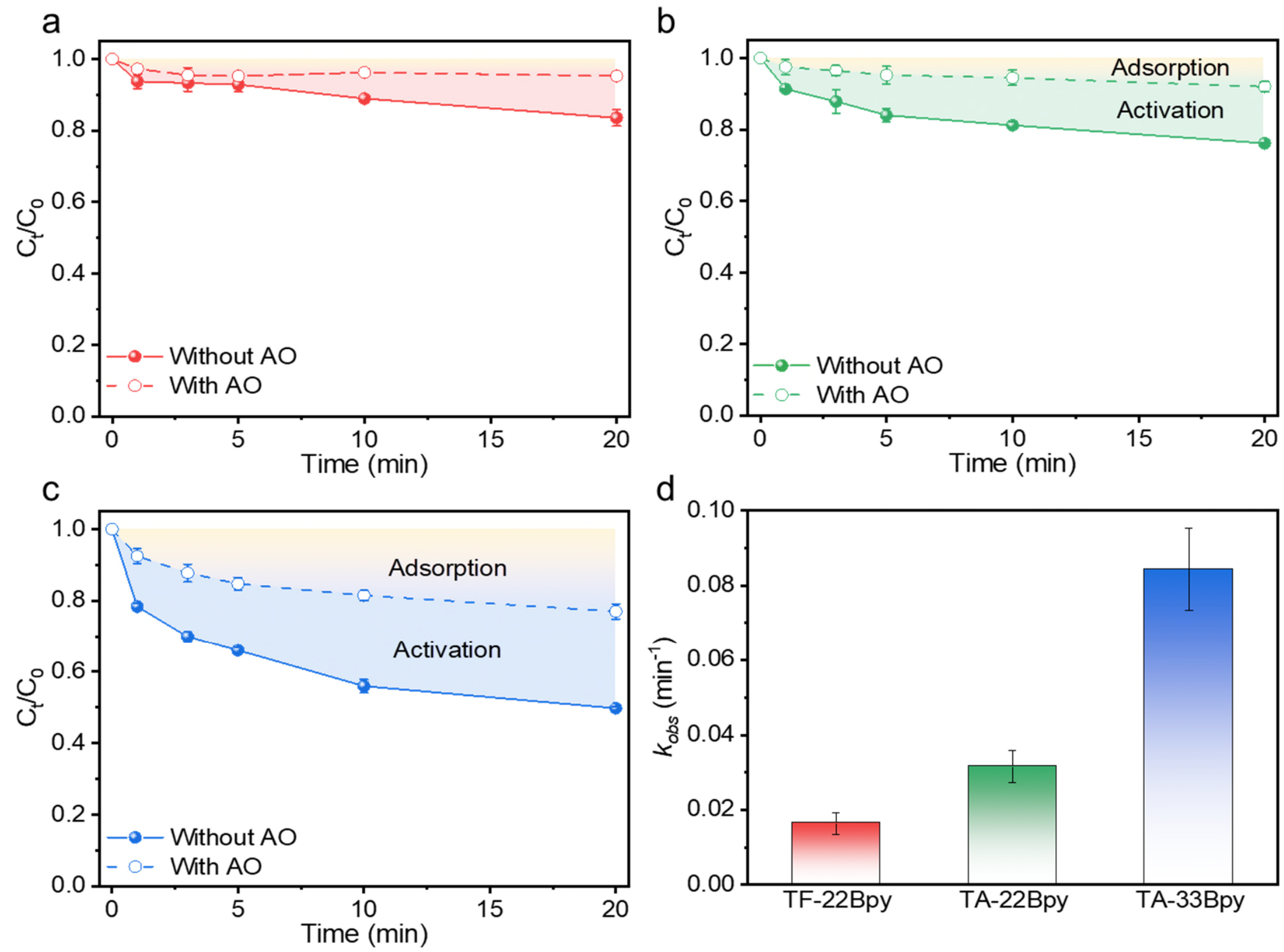

Fig. S20 The consumption and adsorption of PMS by (a) TF-22Bpy, (b) TA-22Bpy and (c) TA-33Bpy. (d) PMS consumption kinetics.

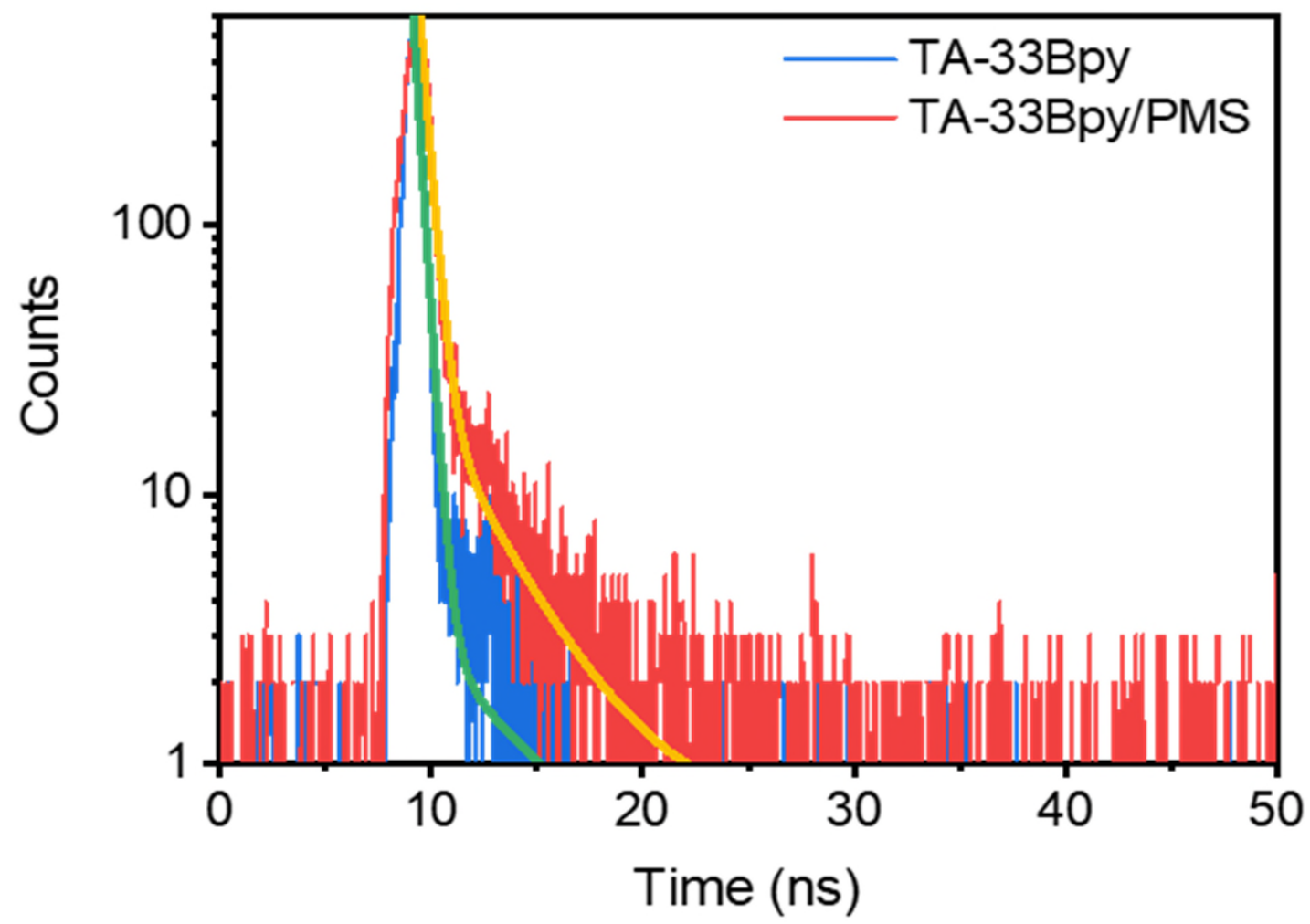

Fig. S27 Time-resolved photoluminescence decay plots of TA-33Bpy and TA-33Bpy/PMS.

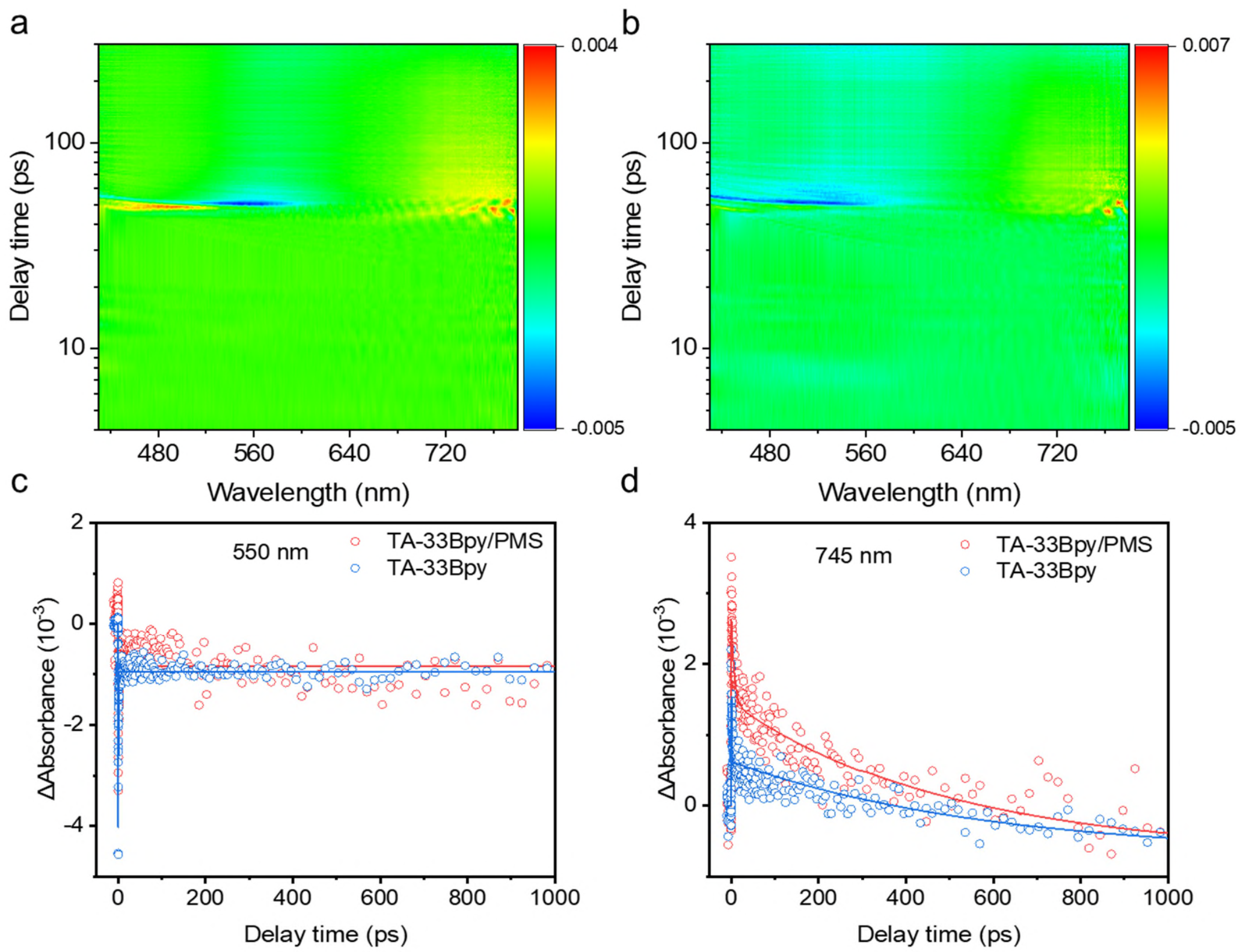

Fig. S28 2D mapping TA spectra of (a) TA-33Bpy and (b) TA-33Bpy/PMS. Decay kinetic curves on (c) 550 nm and (d) 745 nm.

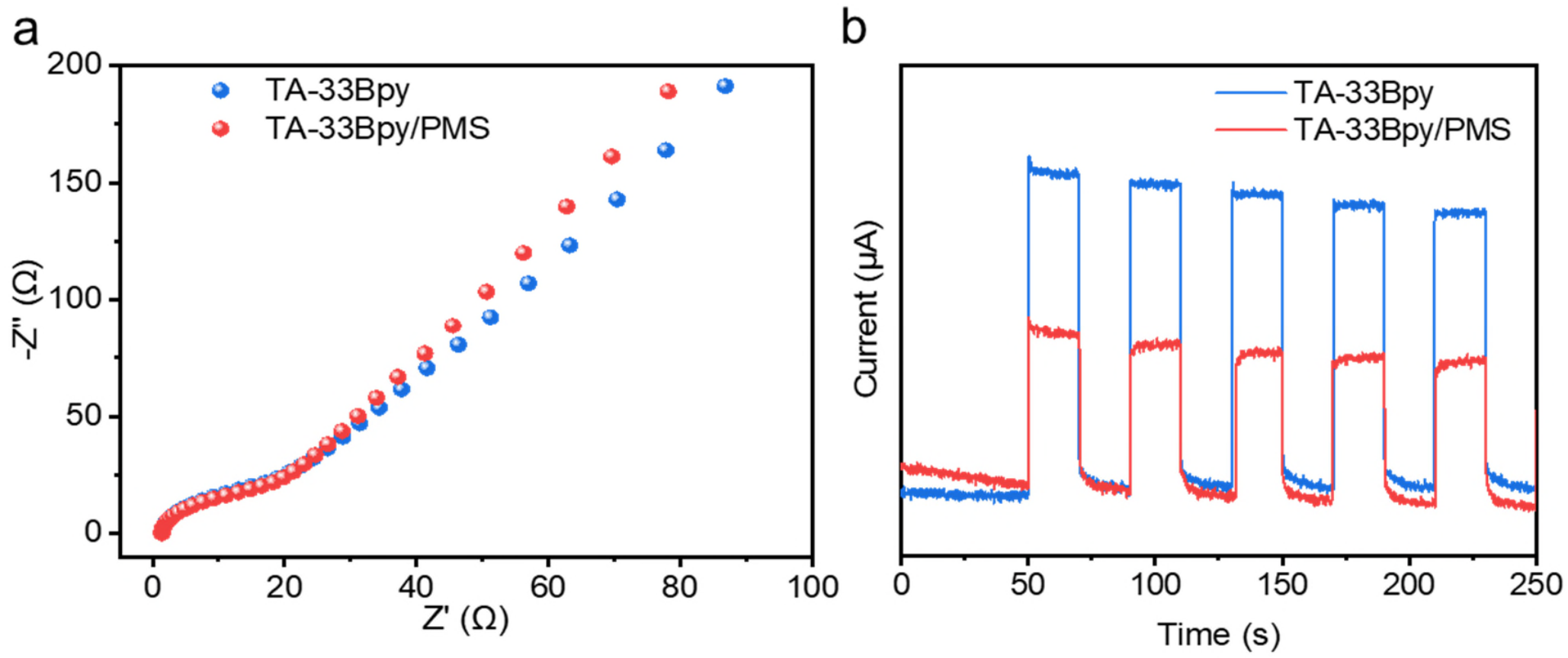

Fig. S29 (a) Electrochemical impedance spectra and (b) transient photocurrent responses of TA-33Bpy and TA-33Bpy/PMS.

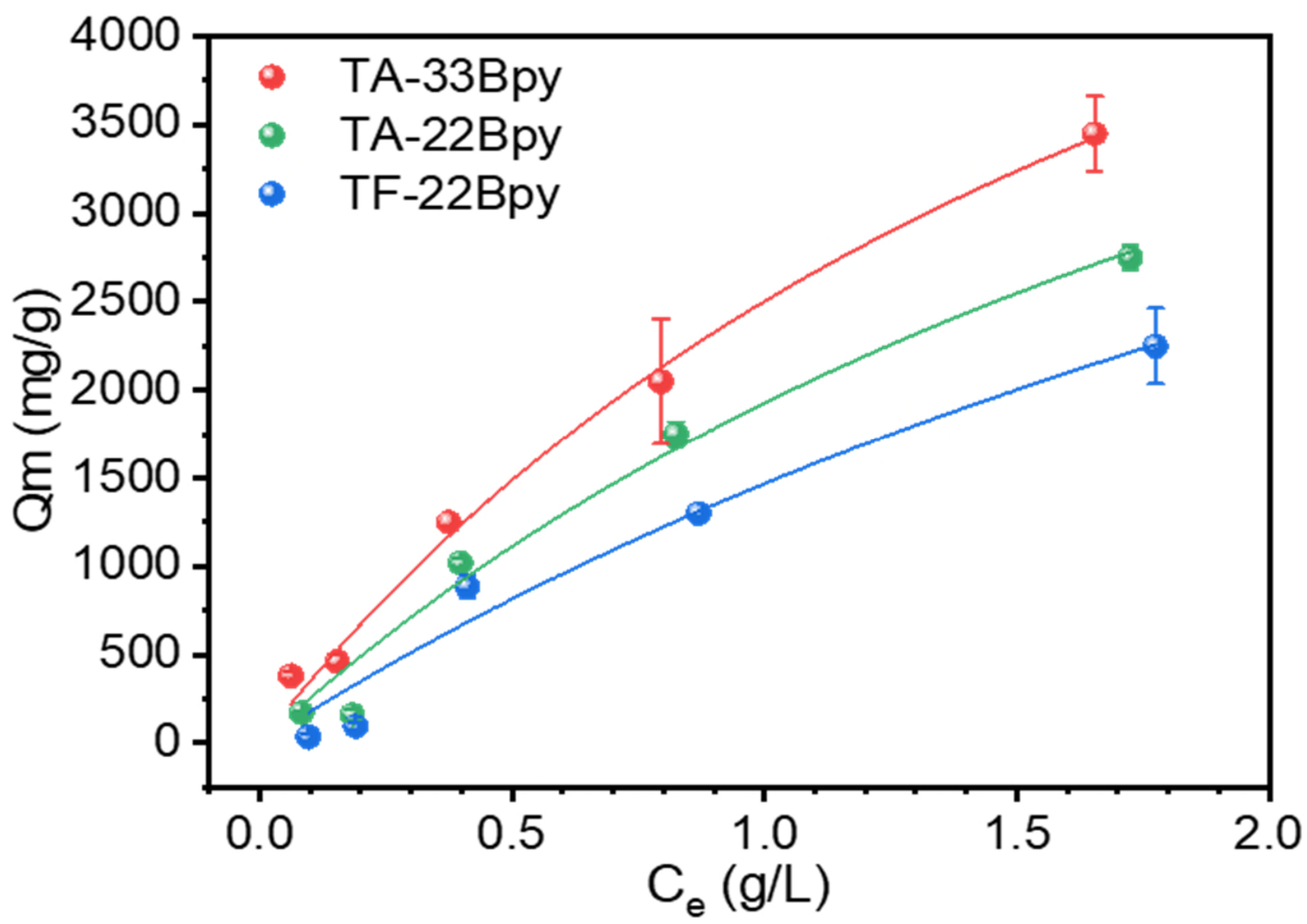

Fig. S30 Isothermal adsorption curves of PMS by COFs.

Supporting Information, Page 42,

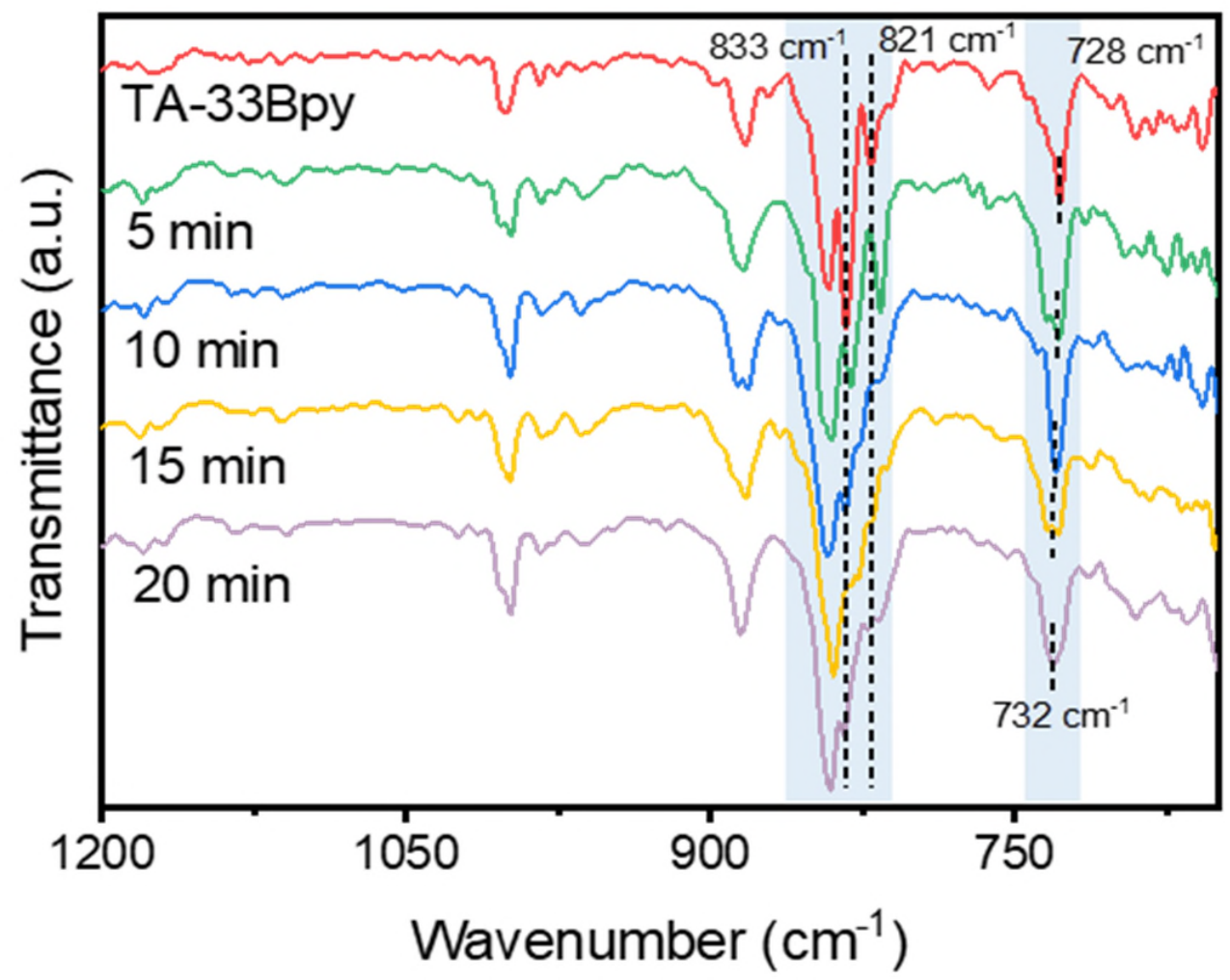

Fig. S36 In situ FT-IR spectra of TA-33Bpy/PMS system.

Supporting Information, Page 65,

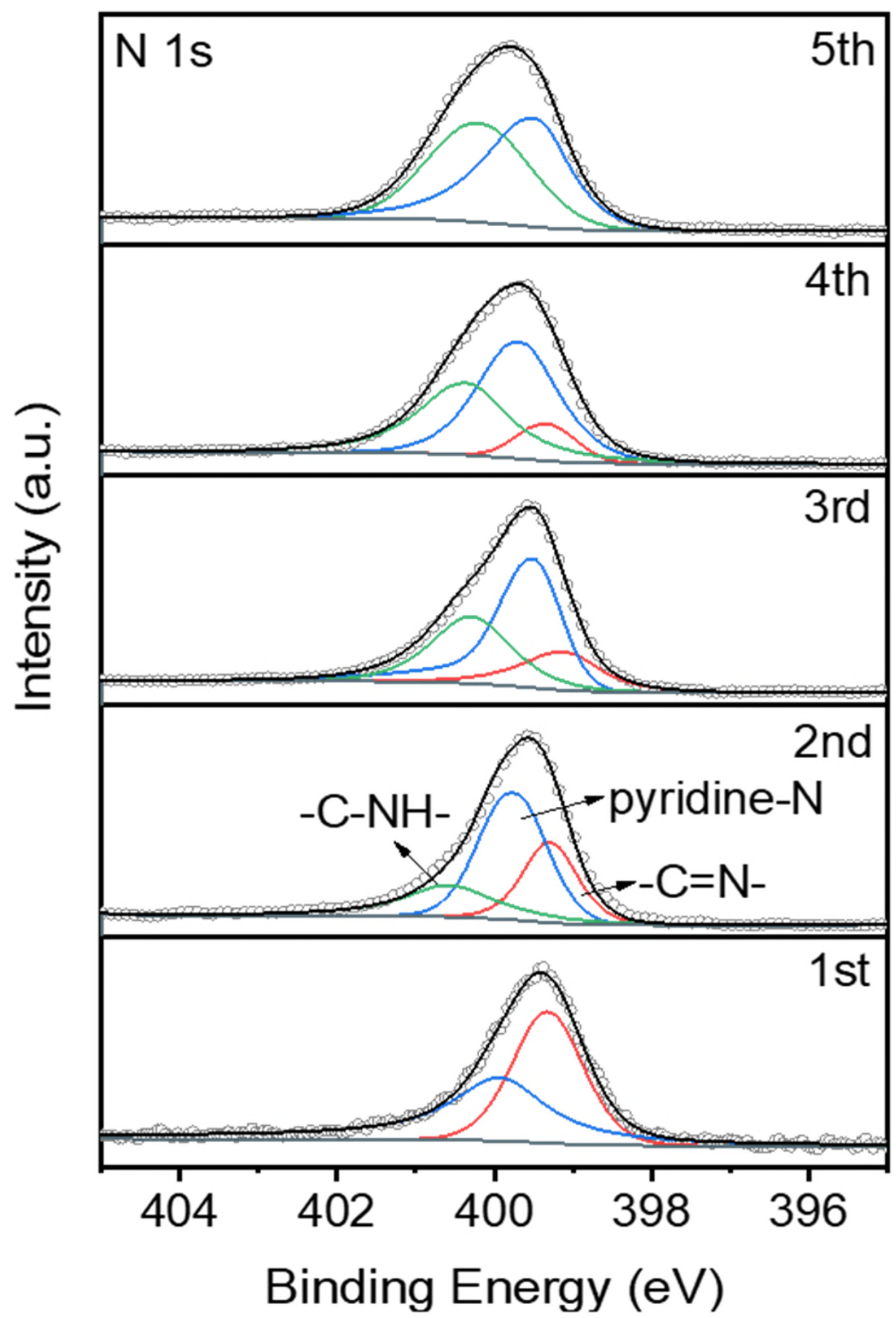

Fig. S59 N 1s spectra of the recycled TA-33Bpy.

Comment 4. A major issue for translation and high-impact claims: the manuscript reports that after reaction the imine C=N band disappears and a new amide C=O band appears (suggesting oxidation/hydrolysis/amide formation), alongside a dramatic BET surface-area loss and pore blocking/distortion. This indicates the catalyst is not stable in the reaction environment and may be chemically converted into a different material during operation. If the active state is a transformed framework, that must be acknowledged and mechanistically addressed. Multi-cycle reusability with activity retention (≥5–10 cycles), with full post-reaction characterization after each cycle is needed. I also recommend providing carbon/nitrogen mass balance and identification of framework decomposition products. It is important to demonstrate whether the transformed material is the true active species (compare fresh vs spent catalyst activity normalized to surface area / active sites).

Response: We appreciate Reviewer #1 for this valuable comment. As suggested, we performed five consecutive catalytic cycles and recovered the catalyst after each cycle. The recovered catalyst from each cycle was systematically characterized by PXRD, N_2 adsorption-desorption measurements, and XPS. In addition, the catalytic activity of the fresh and spent catalysts was compared after normalization by specific surface area, in order to assess whether the evolved material contributes differently to the observed activity. The corresponding revisions have been added to the manuscript as follows:

Page 23, line 556,

"Furthermore, recycling experiments were performed, and the catalysts recovered after each cycle were systematically characterized to track their structural evolution during operation. As shown in Fig. S56, the reaction rate gradually decreased with increasing cycle number, accompanied by slight reductions in specific surface area and pore volume, whereas the pore size remained essentially unchanged (Fig. S57). In addition, the PXRD patterns showed negligible changes in crystallinity throughout the cycling process (Fig. S58). These results suggest that the decrease in surface area and partial pore blockage mainly originated from the accumulation of BPA-derived polymeric products during continuous-flow operation, which could be largely removed by methanol washing. To minimize the influence of surface-area variation, the observed rate constants (k_{obs}) were further normalized by the specific surface area for each cycle (Table S9). The area-normalized kinetic constant began to decline noticeably after the fourth cycle, indicating a gradual decrease in the

intrinsic catalytic activity. To further probe the chemical evolution of the framework, we conducted XPS analyses on the catalysts collected after different cycles. As shown in Fig. S59, the amide-related signal progressively increased, while the imine-related signal gradually weakened with increasing cycle number. Notably, the imine species nearly disappeared after the fourth cycle, consistent with the decline in the area-normalized activity, suggesting that the imine linkage plays a critical role in PMS activation (Table S10). The gradual loss of catalytic activity was mainly attributed to the limited stability of imine linkages under prolonged oxidative conditions. Accordingly, periodic regeneration through organic-solvent washing followed by mild drying, together with post-synthetic conversion of imine linkages into more robust heterocyclic linkages while preserving their protonatable character for PMS interaction, may provide effective strategies for improving the long-term durability of TA-33Bpy."

Supporting Information, Page 62-65,

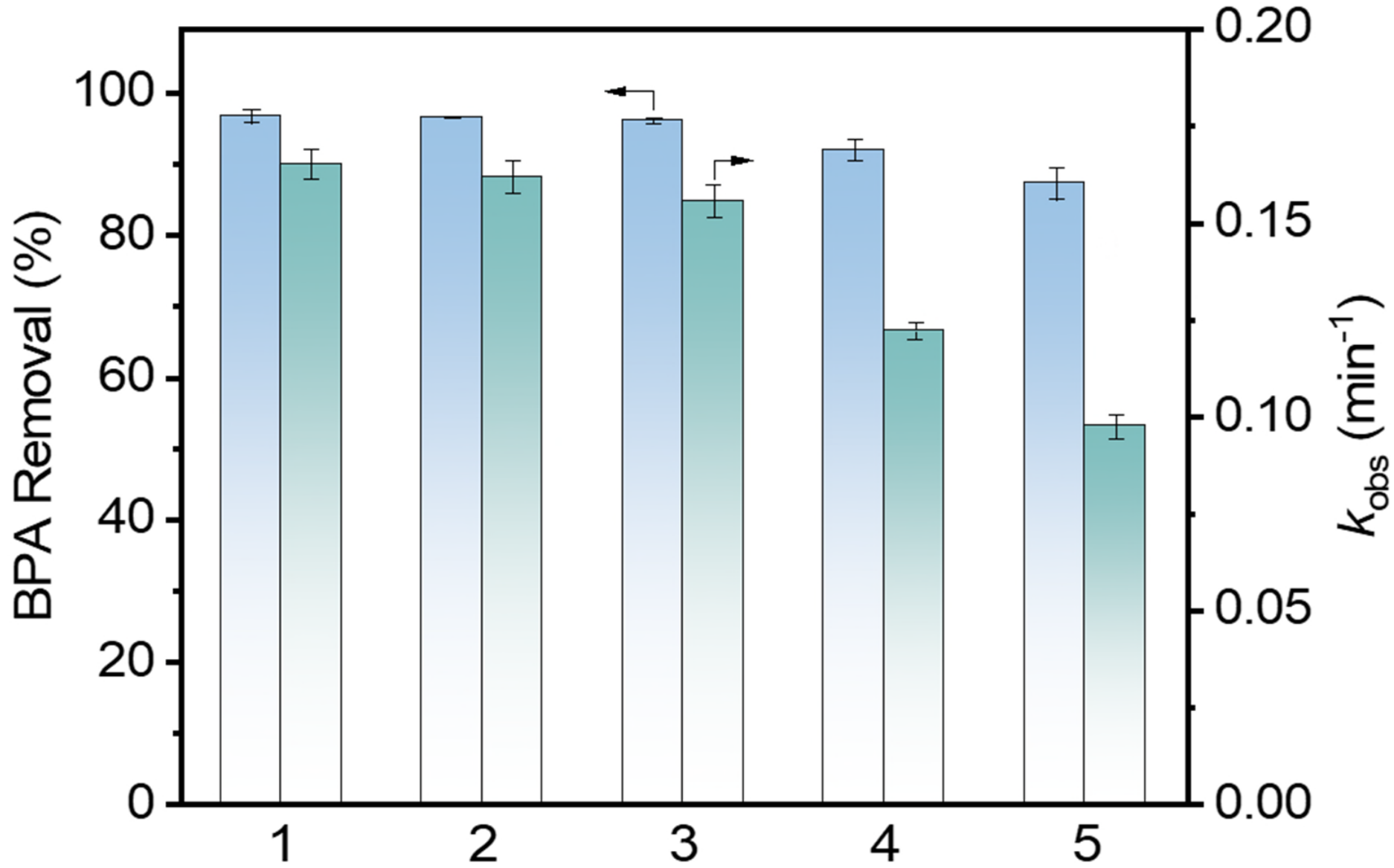

Fig. S56 Recycling of TA-33Bpy.

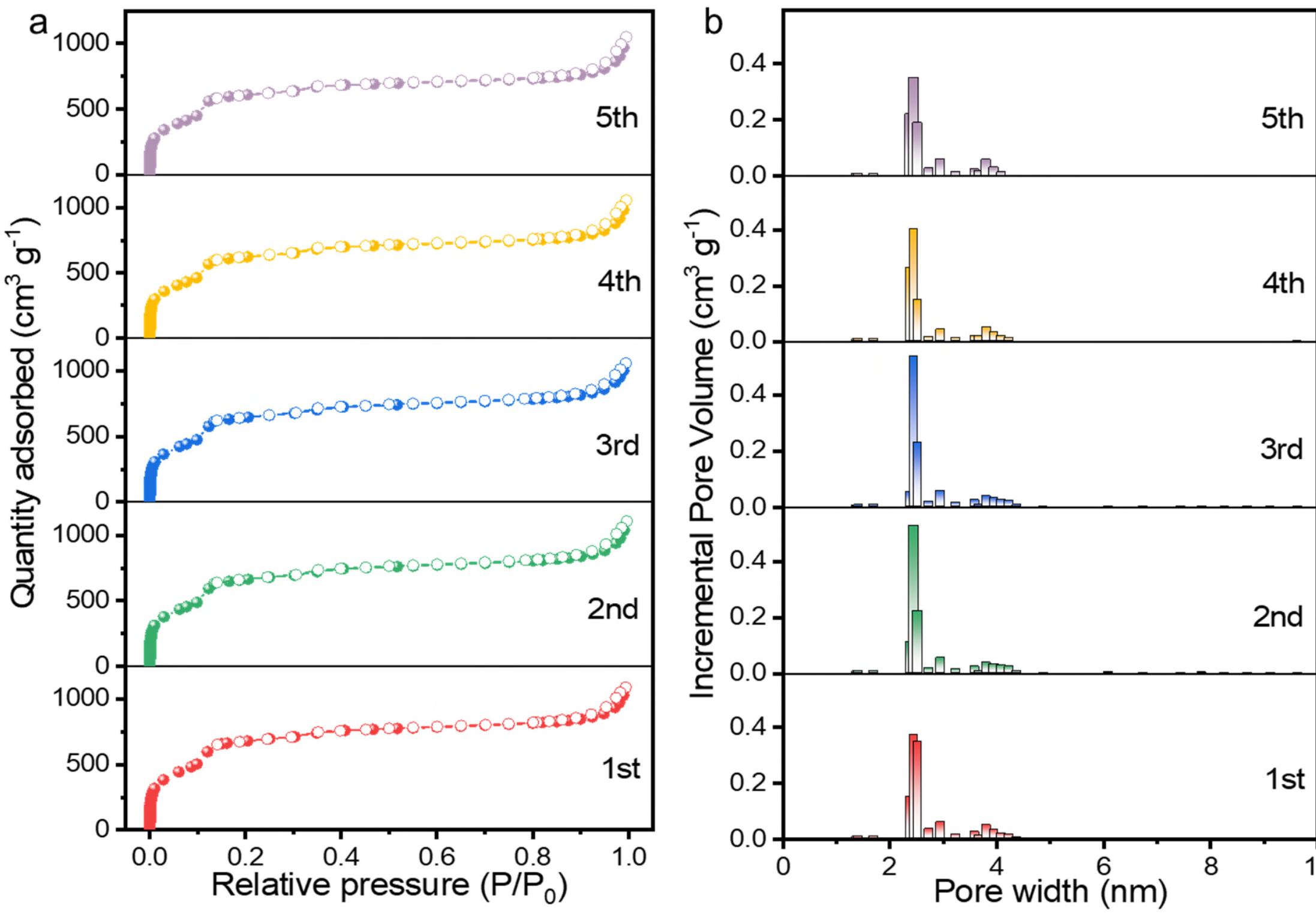

Fig. S57 (a) N_2 adsorption-desorption isotherms, and (f) pore size distributions of the recycled TA-33Bpy.

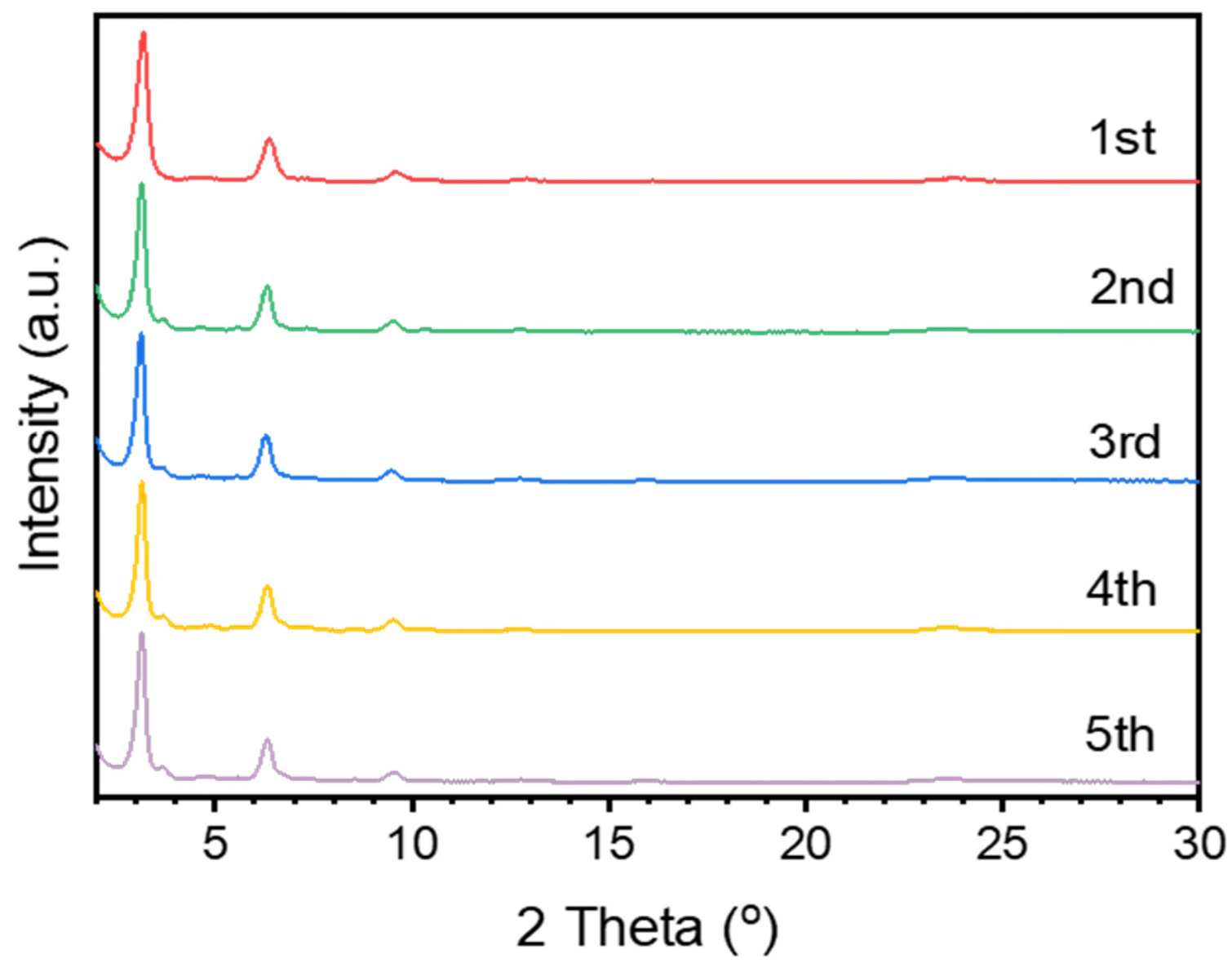

Fig. S58 XRD patterns of the recycled TA-33Bpy.

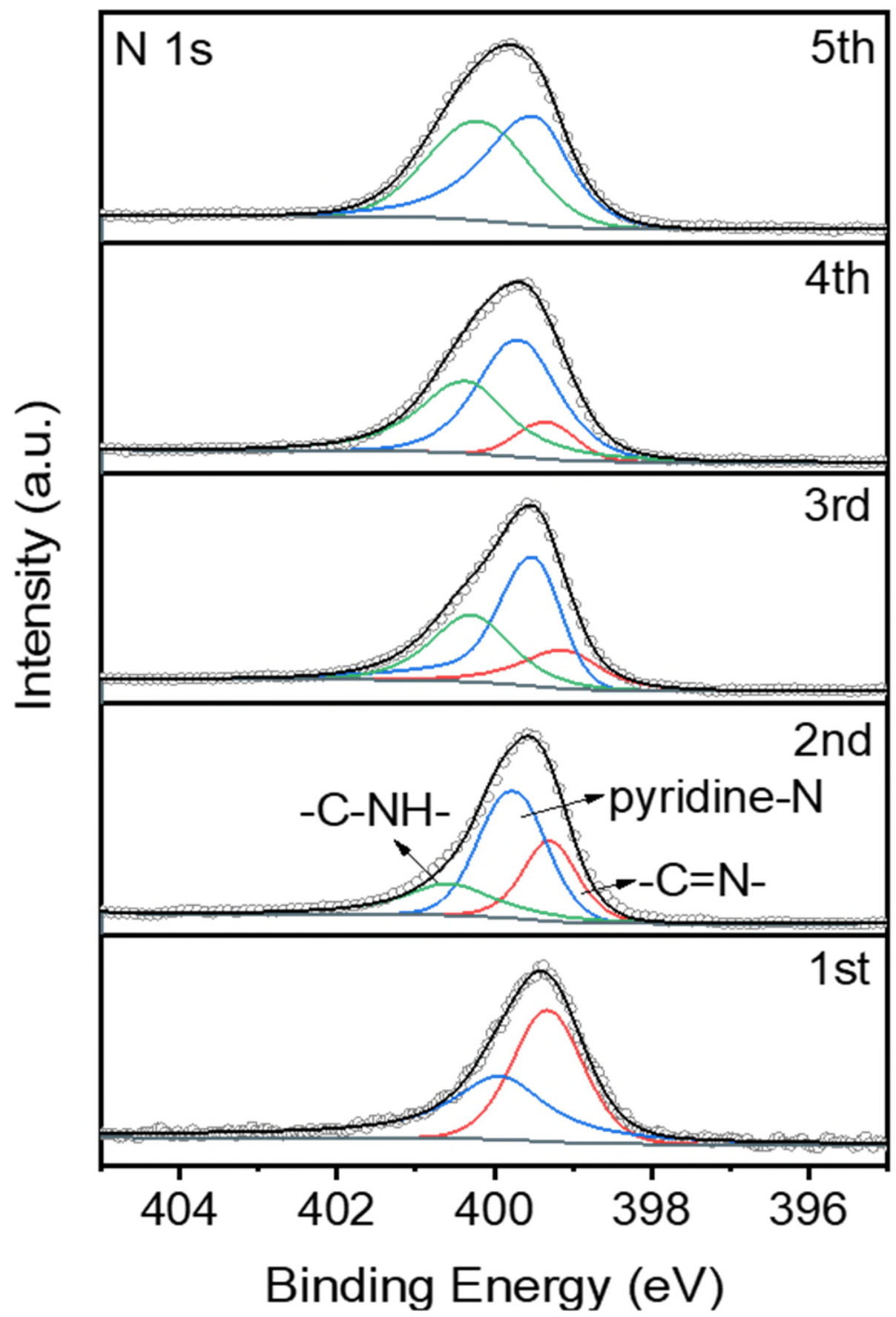

Fig. S59 N 1s spectra of the recycled TA-33Bpy.

Supporting Information, Page 77-78,

Table S9. Textural properties, k_{obs} and area-normalized k of TA-33Bpy with different recycle.

Number of use	Surface Area (m^2 g^{-1})	Pore Volume (cm^3 g^{-1})	Pore Size (nm)	k_{obs} (min^{-1})	area-normalized k ($\times10^{-5}$)
1	2223	1.29	2.48	0.165	7.42
2	2150	1.30	2.48	0.162	7.53
3	2095	1.26	2.48	0.156	7.45
4	1999	1.24	2.48	0.122	6.10
5	1982	1.22	2.48	0.098	4.94

Table S10. Nitrogen spices of TA-33Bpy with different recycle.

Number of use	-C=N- (at.%)	Pyridine-N (at.%)	-C-NH- (at.%)
1	48.78	51.22	0
2	26.95	50.93	22.12
3	18.31	50.47	31.22
4	10.84	49.88	39.28
5	0	50.59	49.41

Comment 5. The application is primarily BPA degradation under selected conditions, with limited demonstration across contaminant classes and realistic matrices. In advanced oxidation for water, a high-impact paper should show robustness against real-world confounders and broader pollutant scope, plus meaningful comparisons to state-of-the-art benchmarks. It is important to discuss the substrate scope: multiple pollutants (electron-rich vs electron-poor), pharmaceuticals, dyes, PFAS surrogate (if feasible), etc., and relate selectivity to $^1O_2$ chemistry. I recommend realistic water tests: actual secondary effluent, surface water, high NOM, high alkalinity, chloride/bromide/nitrate mixtures—plus byproduct analysis. Bromate/chlorate or halogenated byproduct assessment (especially if oxidants are used) is needed. It is also imperative to benchmark against leading metal-free catalysts/COFs and report energy/oxidant efficiency metrics.

Response: We appreciate the constructive comments from Reviewer #1. Following the suggestion, the application-oriented evaluation of the COF/PMS system has been expanded beyond BPA degradation. Specifically, additional pollutant degradation tests were conducted using representative substrates, including rhodamine B (RhB), methyl orange (MO), Congo red (CR), sulfanilamide (SA), sulfamethoxazole (SMX), carbamazepine (CBZ), and acetaminophen (AcP). To evaluate the robustness of the system under more practical water-treatment conditions, we performed additional experiments in the presence of humic acid, high alkalinity, and mixed coexisting anions, including Cl^-, Br^-, and NO_3^-. Degradation tests were also carried out in real water matrices, including surface water and a secondary effluent. In addition, potential halogenated byproducts and oxyhalide byproducts, including chlorate and bromate, were examined under the tested conditions. Moreover, the benchmarking section has been strengthened by comparing the present COF/PMS system with representative metal-free catalysts and COF-based PMS activation systems reported in the literature. A normalized activity parameter (k-value = $(k_{obs} \times C_{BPA})/(C_{PMS} \times C_{cat.})$), was introduced to enable a more meaningful comparison of catalytic performance under different reaction conditions. Relevant revisions are added as follows:

Page 12, line 262,

"To further isolate the contribution of surface area, the k_{obs} values were standardized based on the specific surface areas of three COFs. The resulting area-normalized k_{obs} values were 7.42×10^{-5}, 2.03×10^{-5} and 0.57×10^{-5} for TA-33Bpy, TA-22Bpy and TF-22Bpy, respectively. This result indicated

that the improvement in performance mainly originated from the intrinsic effect of the N-site isomerism in regulating the electronic structure. In order to conduct a more comprehensive comparison with various systems, a standardized kinetic model (*k*-value) was adopted, taking into account of the amount of catalyst, oxidant and pollutant concentrations. According to Table S8, the k-value of TA-33Bpy exceeds that of the majority of metal-based and non-metal-based catalysts, highlighting the superior efficiency of TA-33Bpy/PMS system."

Page 21, line 482,

"The influence of COF loading on BPA removal was systematically investigated (Fig. S42). Increasing the catalyst dosage from 0.05 to 0.2 g L^{-1} led to a corresponding increase in the reaction rate constant from 0.09 to 0.33 min^{-1}. To further evaluate the environmental adaptability of the TA-33Bpy/PMS system, we examined the effects of common water constituents and solution pH on BPA degradation. As shown in Fig. S43, the presence of NO_3^-, Cl^-, HCO_3^-, SO_4^{2-} and HPO_4^{2-} exerted a negligible influence on both BPA removal efficiency and k_{obs}. Interestingly, in the presence of Br^-, BPA was rapidly removed within 1 min even without catalyst addition, which is likely related to the relatively high nucleophilicity of bromide species. Moreover, complete BPA removal could still be achieved within 1 min in mixed systems containing Br^-, Cl^- and NO_3^-, while HPLC-MS analysis confirmed the absence of typical halogenated byproducts. To investigate the influence of natural organic matter, we further evaluated the catalytic performance in the presence of different concentrations of humic acid (HA). As depicted in Fig. S44, BPA removal efficiency and reaction kinetics gradually decreased with an increasing HA concentration, likely due to competitive ROS consumption and partial blockage of catalytic active sites by HA adsorption. The effect of solution pH was also examined. Acidic conditions favored BPA degradation, whereas alkaline conditions significantly suppressed the reaction (Fig. S45a). Zeta-potential measurements showed that the surface of TA-33Bpy became progressively more negatively charged with increasing pH (Fig. S45b). Such deprotonation-induced surface charge variation likely enhanced the electrostatic repulsion between TA-33Bpy and anionic HSO_5^- species, thereby reducing the interfacial PMS activation efficiency."

Page 22, line 504,

"The universality of the TA-33Bpy/PMS system was further evaluated using a variety of

representative pollutants, including rhodamine B (RhB), methyl orange (MO), Congo red (CR), sulfanilamide (SA), sulfamethoxazole (SMX), carbamazepine (CBZ), and acetaminophen (AcP). As shown in Fig. S46, MO, CR, and AcP were rapidly degraded, while sulfonamides and RhB exhibited relatively lower removal efficiencies. In contrast, only 7.0% of CBZ was removed under the same conditions. These results indicate that the TA-33Bpy/PMS system possesses broad applicability and shows preferential reactivity toward electron-rich contaminants that are neutral or negatively charged under acidic conditions. In addition, BPA degradation was further examined in real water matrices, including a secondary effluent and surface water. As shown in Fig. S47, a slight inhibition of BPA removal was observed in both cases, which is likely attributable to the presence of coexisting constituents in the water matrix that compete for reactive species or interfere with PMS activation."

Supporting Information, Page 48-53,

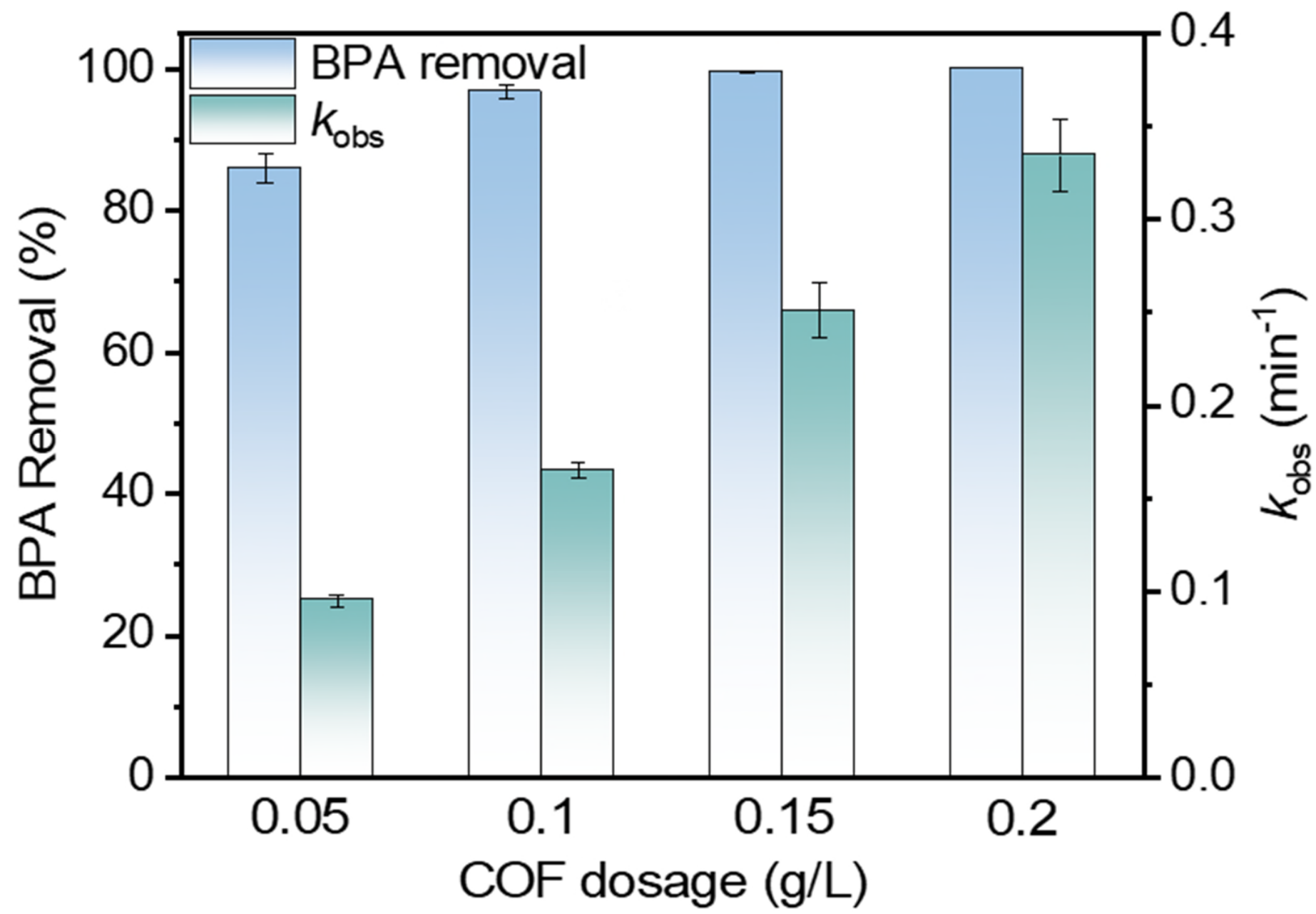

Fig. S42 The influences of COF loading on BPA removal in TA-33Bpy/PMS system.

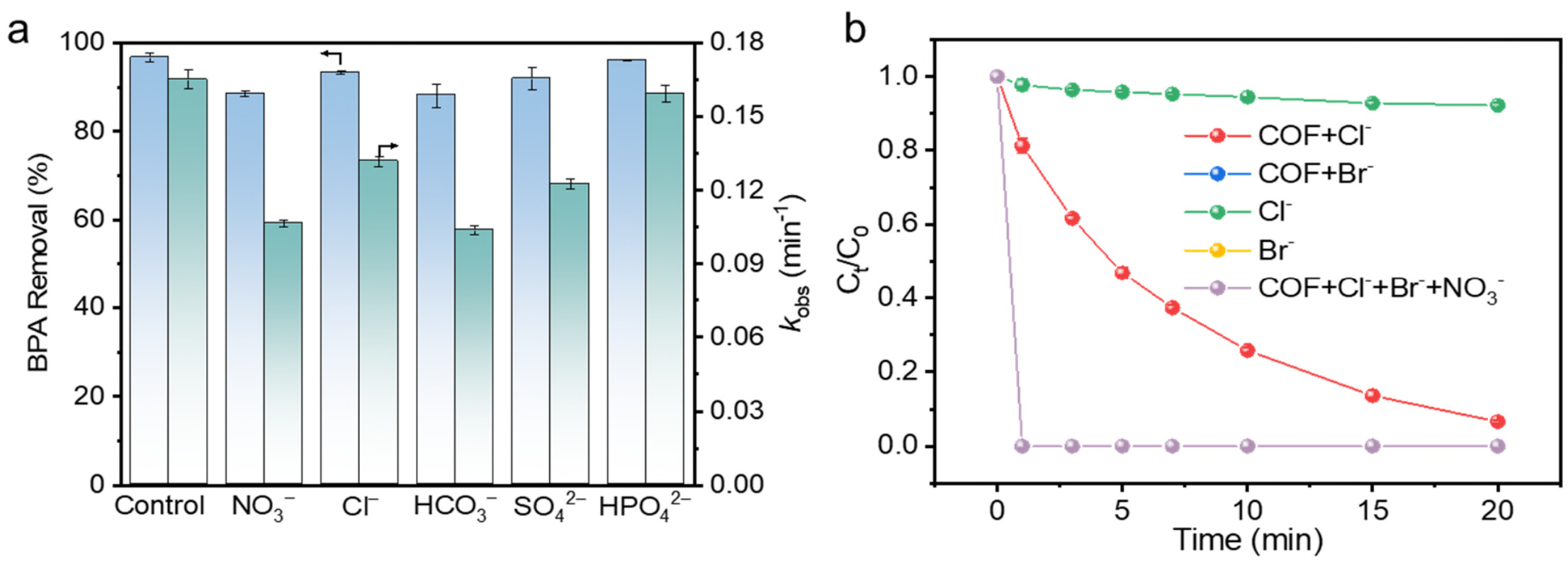

Fig. S43 The influences of (a) coexisting ion and (b) halide ion on BPA removal in TA-33Bpy/PMS system.

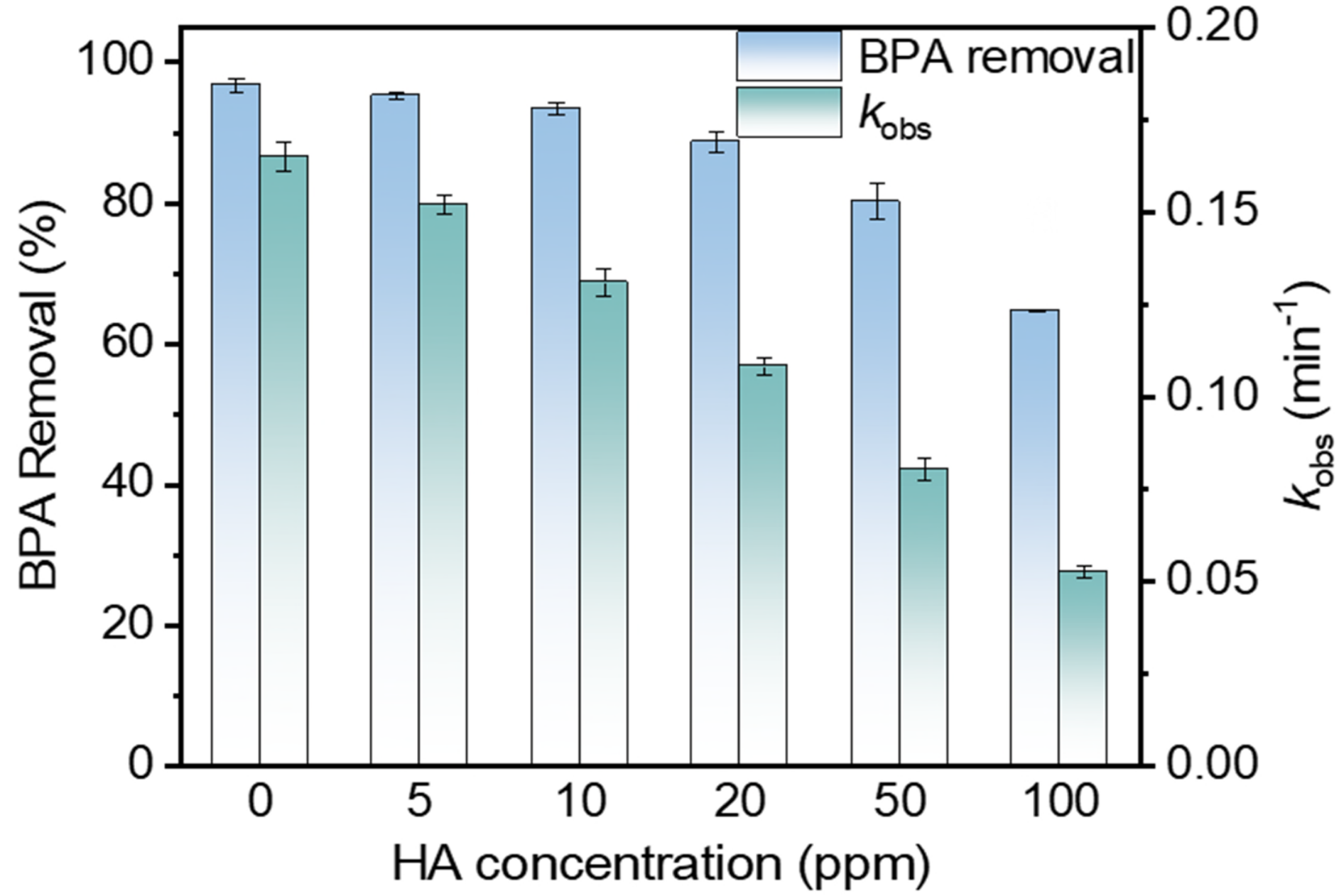

Fig. S44 The influences of HA on BPA removal in TA-33Bpy/PMS system.

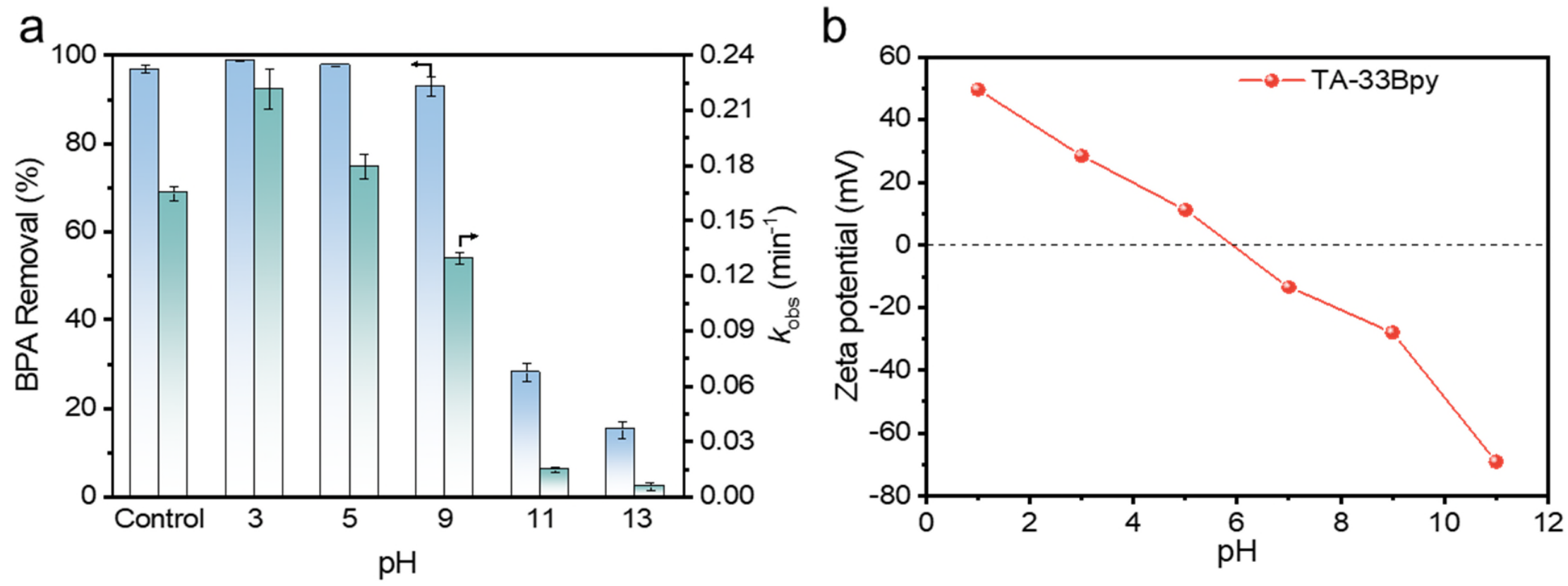

Fig. S45 (a) The influence of pH on BPA removal in TA-33Bpy/PMS system. (b) Zeta potential of TA-33Bpy.

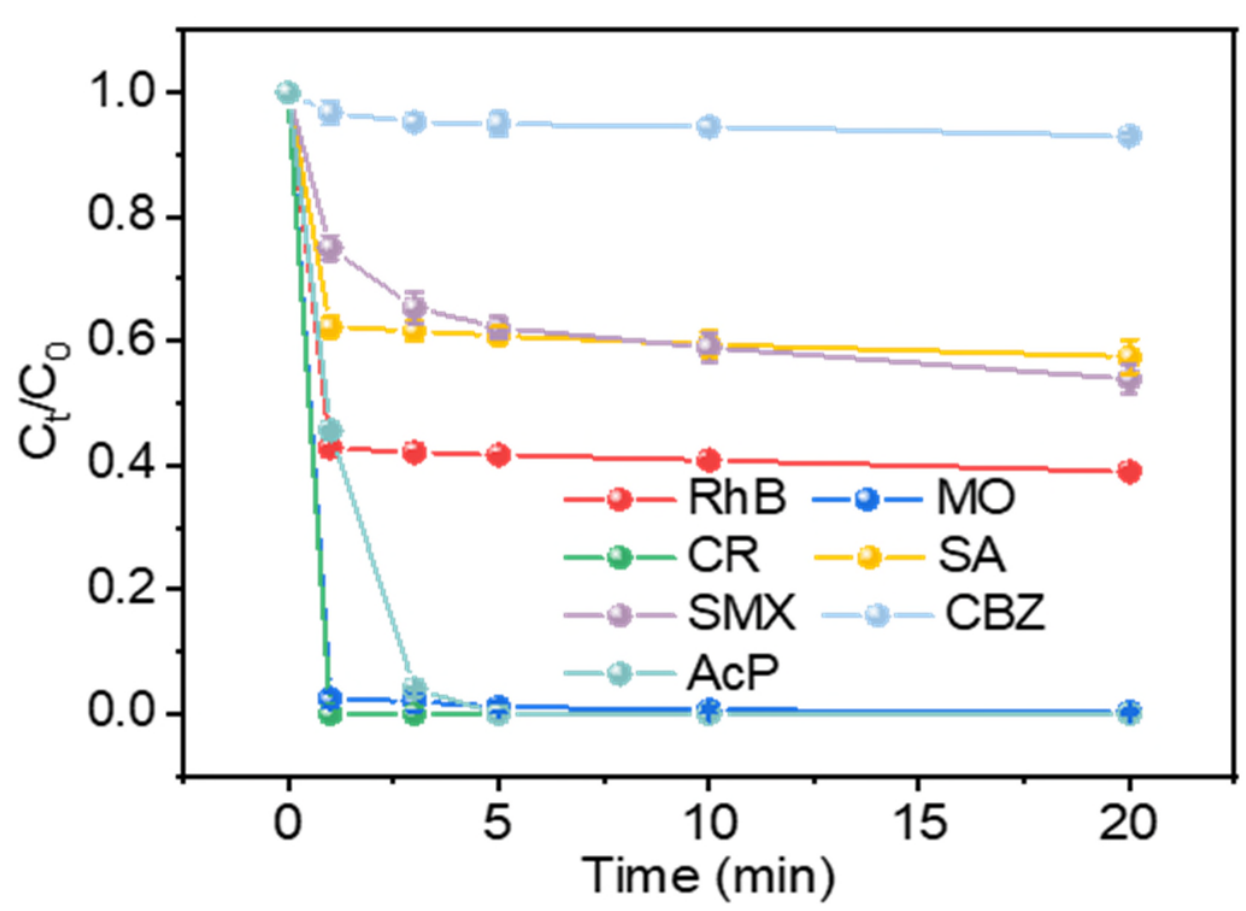

Fig. S46 Removal of different pollutants by TA-33Bpy/PMS system.

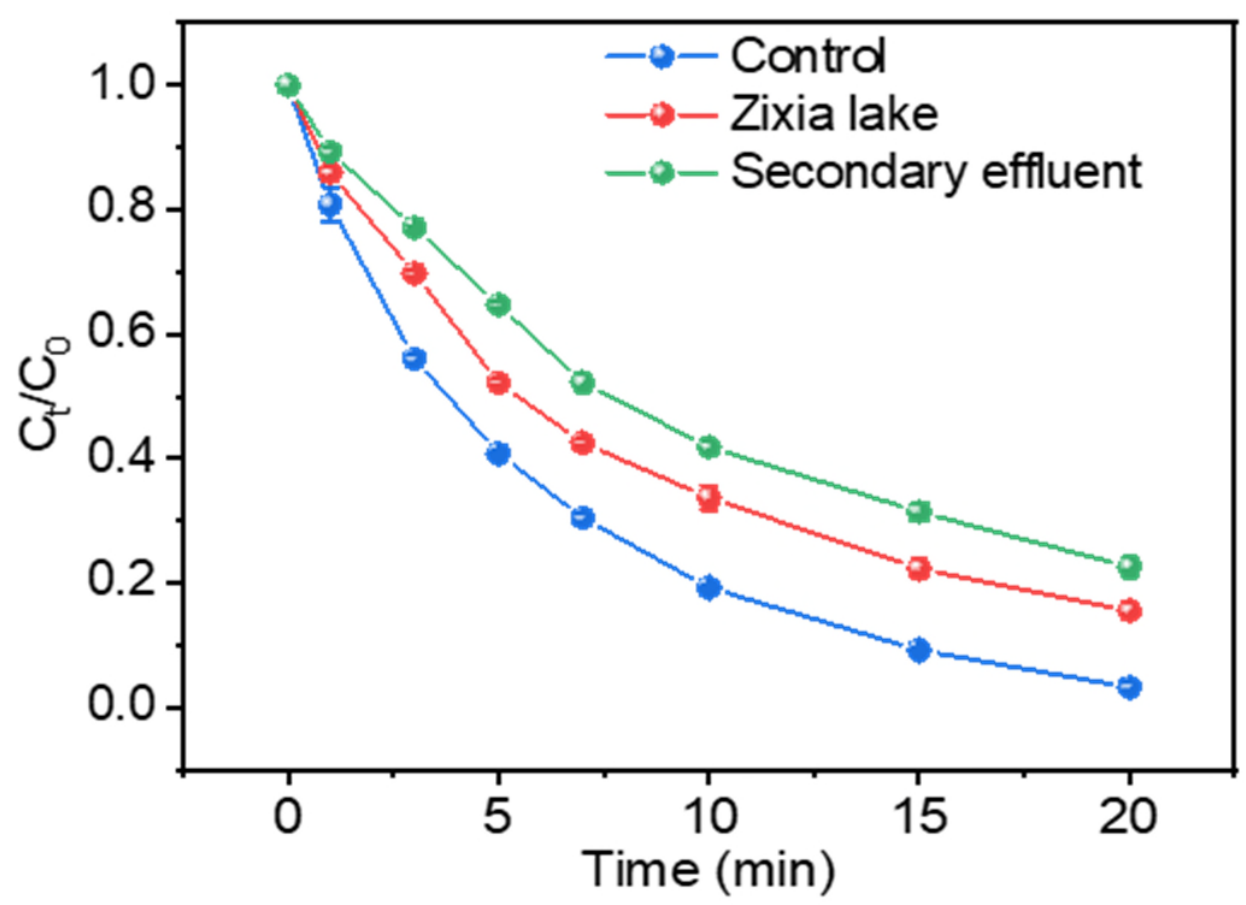

Fig. S47 BPA Removal by TA-33Bpy/PMS system in actual water.

Supporting Information, Page 76,

Table S8. Catalytic performance comparison of recently reported catalysts for BPA removal.

Type	Catalyst (g L^{-1})	PMS (mM)	BPA (mg L^{-1})	k_{obs} (min^{-1})	*k-value* (min^{-1} M^{-1})	Ref.
COFs	TA-33Bpy (0.1)	0.65	20	0.165	50.77	This work
	BPY-COFs (0.3)	5	10	1.250	8.33	1
	BPD-COFs (0.3)	5	10	0.130	0.87	
Carbon Material	NOPC-1 (0.2)	0.15	10	0.152	50.49	2
	NOPC-2 (0.2)	0.15	10	0.187	62.12	
	NOPC (0.2)	0.15	10	0.300	99.65	
	NOPC-3 (0.2)	0.15	10	0.064	21.26	
	NOPC-4 (0.2)	0.15	10	0.026	8.64	
	g-C_3N_4(0.2)	0.15	10	0.004	1.23	
	NC-1 (0.15)	0.4	10	0.066	10.97	3
	NC-2 (0.15)	0.4	10	0.016	2.72	
	NC-3 (0.15)	0.4	10	0.003	0.57	
Single-Atom Catalyst	Fe-SAC (0.1)	1.30	25	0.100	19.20	4
	Co-SAC (0.1)	1.30	25	0.080	15.36	
	Mn-SAC (0.1)	1.30	25	0.060	11.52	
	Ni-SAC (0.1)	1.30	25	0.050	9.60	
	Cu-SAC (0.1)	1.30	25	0.050	9.60	
Metal/Carbon Material	FM@NBC-8 (0.1)	0.5	20	0.685	273.90	5
	F@NBC-8 (0.1)	0.5	20	0.035	13.87	
	M@NBC-8 (0.1)	0.5	20	0.007	3.00	
	NBC-8 (0.1)	0.5	20	0.007	2.94	
	ZIF(FeC)-Cl (0.1)	0.5	20	0.276	110.56	6
	ZIF(FeC) (0.1)	0.5	20	0.036	14.44	

k-value was calculated by the following equation:

$$k\text{-}value = (k_{obs} \times C_{[Pollutant]}) / (C_{[Catalyst]} \times C_{[PMS]})$$

Supporting Information, Page 79,

1 Weng, Z. *et al.* Site Engineering of Covalent Organic Frameworks for Regulating Peroxymonosulfate Activation to Generate Singlet Oxygen with 100 % Selectivity. *Angew. Chem. Int. Ed.* 62, e202310934 (2023).

2 He, Y.-L. *et al.* Activating peroxymonosulfate by N and O co-doped porous carbon for efficient BPA degradation: A re-visit to the removal mechanism and the effects of surface unpaired electrons. *Applied Catalysis B: Environmental* 314, 121390 (2022).

3 Qu, G. *et al.* Enhanced peroxymonosulfate activation via heteroatomic doping defects of pyridinic and pyrrolic N in 2D N-doped carbon nanosheets for BPA degradation. *Journal of Hazardous Materials* 461, 132626 (2024).

4 Gao, Y. *et al.* Activity Trends and Mechanisms in Peroxymonosulfate-Assisted Catalytic Production of Singlet Oxygen over Atomic Metal-N-C Catalysts. *Angew. Chem. Int. Ed.* 60, 22513-22521 (2021).

5 Xiao, H. *et al.* N-doped biochar-Fe/Mn as a superior peroxymonosulfate activator for enhanced bisphenol a degradation. *Water Res.* 278, 123399 (2025).

6 Huang, J. *et al.* Chlorine atoms modulated Fe d-band centers drive non-radical pathways to degrade bisphenol A with attenuated intermediates toxicity. *Chem. Eng. J.* 524, 169699 (2025).

Comment 6. k_{obs} comparisons across materials with different adsorption capacity, morphology (sea-urchin-like vs particles), and potentially different accessible active sites can be misleading without normalization. TOC removal is modest relative to BPA disappearance, suggesting partial oxidation and/or adsorption/polymerization, which needs deeper discussion. Normalize activity to accessible surface area, active site density (N content, pyridine/imine quantification), or catalyst external surface area if internal pores are not fully accessible. Provide error bars (n≥3), statistical tests, and report catalyst loading dependence with kinetic regimes identified (mass-transfer vs kinetic control). Provide oxidant consumption kinetics and PMS utilization efficiency.

Response: We appreciate the valuable comments from Reviewer #1. Following the suggestion, k_{obs} was normalized by the specific surface area to minimize the influence of differences in morphology and structural accessibility among the COFs. The discussion of BPA adsorption/aggregation behavior has also been expanded. In addition, catalyst-loading-dependent experiments were added to clarify the kinetic regime, including the possible contributions of mass-transfer and kinetic control. Oxidant consumption kinetics and PMS utilization efficiency were further analyzed to provide a more comprehensive evaluation of catalytic performance. The revised parts are presented as follows:

Page 12, line 262,

"To further isolate the contribution of surface area, the k_{obs} values were standardized based on the specific surface areas of three COFs. The resulting area-normalized k_{obs} values were 7.42×10^{-5}, 2.03×10^{-5} and 0.57×10^{-5} for TA-33Bpy, TA-22Bpy and TF-22Bpy, respectively. This result indicated that the improvement in performance mainly originated from the intrinsic effect of the N-site isomerism in regulating the electronic structure."

Page 13, line 297,

"To quantitatively verify $^1O_2$ generation in the heterogeneous PMS system, we further conducted PMS consumption, ABDA-based $^1O_2$ quantification, and FFA kinetic measurements. Within 20 min, the PMS consumption reached 50.2%, 23.8%, and 16.4% for TA-33Bpy, TA-22Bpy, and TF-22Bpy, respectively (Fig. S20). Consistently, ABDA quantification confirmed $^1O_2$ production of 0.122, 0.104, and 0.082 mM, respectively (Fig. S21a). The corresponding PMS utilization efficiencies were calculated to be 37.25%, 67.35%, and 76.86%, indicating that the slower-reacting system used PMS more efficiently (Fig. S21b)."

Page 21, line 482,

"The influence of COF loading on BPA removal was systematically investigated (Fig. S42). Increasing the catalyst dosage from 0.05 to 0.2 g L^{-1} led to a corresponding increase in the reaction rate constant from 0.09 to 0.33 min^{-1}."

Page 22, line 525,

"The identified intermediates suggest that BPA undergoes sequential $^1O_2$-driven oxidation and ring-cleavage reactions, ultimately yielding small organic acids and, eventually, CO_2 and H_2O. Meanwhile, analysis of the methanol-eluted fraction from the spent catalyst surface revealed BPA oligomers, indicating that part of BPA follows a parallel surface-confined oligomerization/polymerization pathway. This interpretation is consistent with recent studies showing that selective nonradical oxidation in persulfate-based AOPs, including $^1O_2$-mediated pathways, can convert phenolic substrates into phenoxy-radical-type intermediates that further couple on catalyst surfaces[63]. The accumulation of these surface-bound products may facilitate initial pollutant removal, but persistent deposition could progressively block active sites and impede interfacial mass/electron transfer, thereby lowering catalyst stability and long-term catalytic efficiency. Furthermore, the ecological toxicity of the detected aqueous intermediates was evaluated using ECOSAR software (version 2.2) based on quantitative structure-activity relationship (QSAR) models."

Page 30, line 791,

"63 Zhang, P., Yang, Y., Duan, X. & Wang, S. Oxidative polymerization versus degradation of organic pollutants in heterogeneous catalytic persulfate chemistry. *Water Res.* **255**, 121485 (2024)."

Supporting Information, Page 26-27,

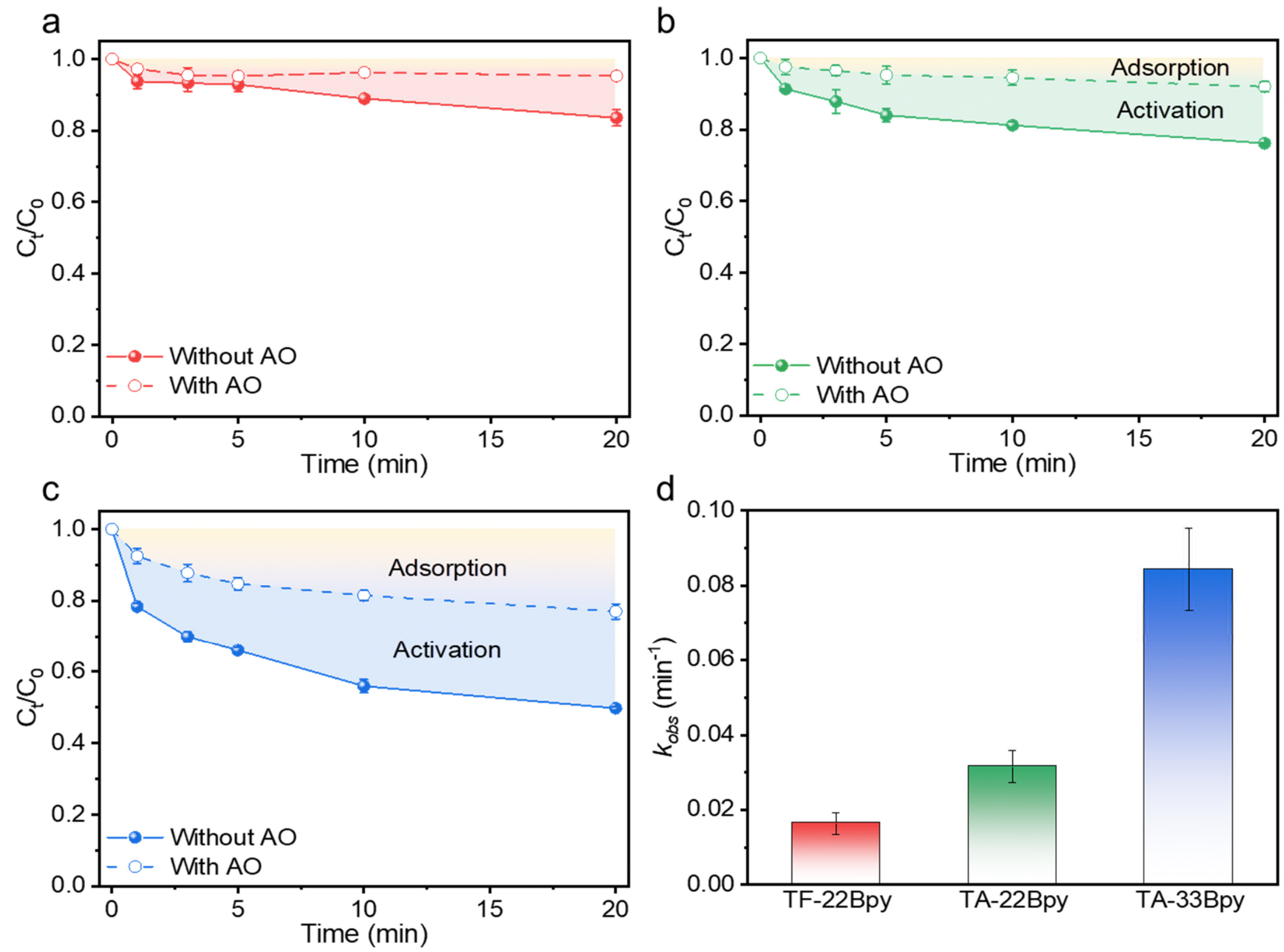

Fig. S20 The consumption and adsorption of PMS by (a) TF-22Bpy, (b) TA-22Bpy and (c) TA-33Bpy. (d) PMS consumption kinetics.

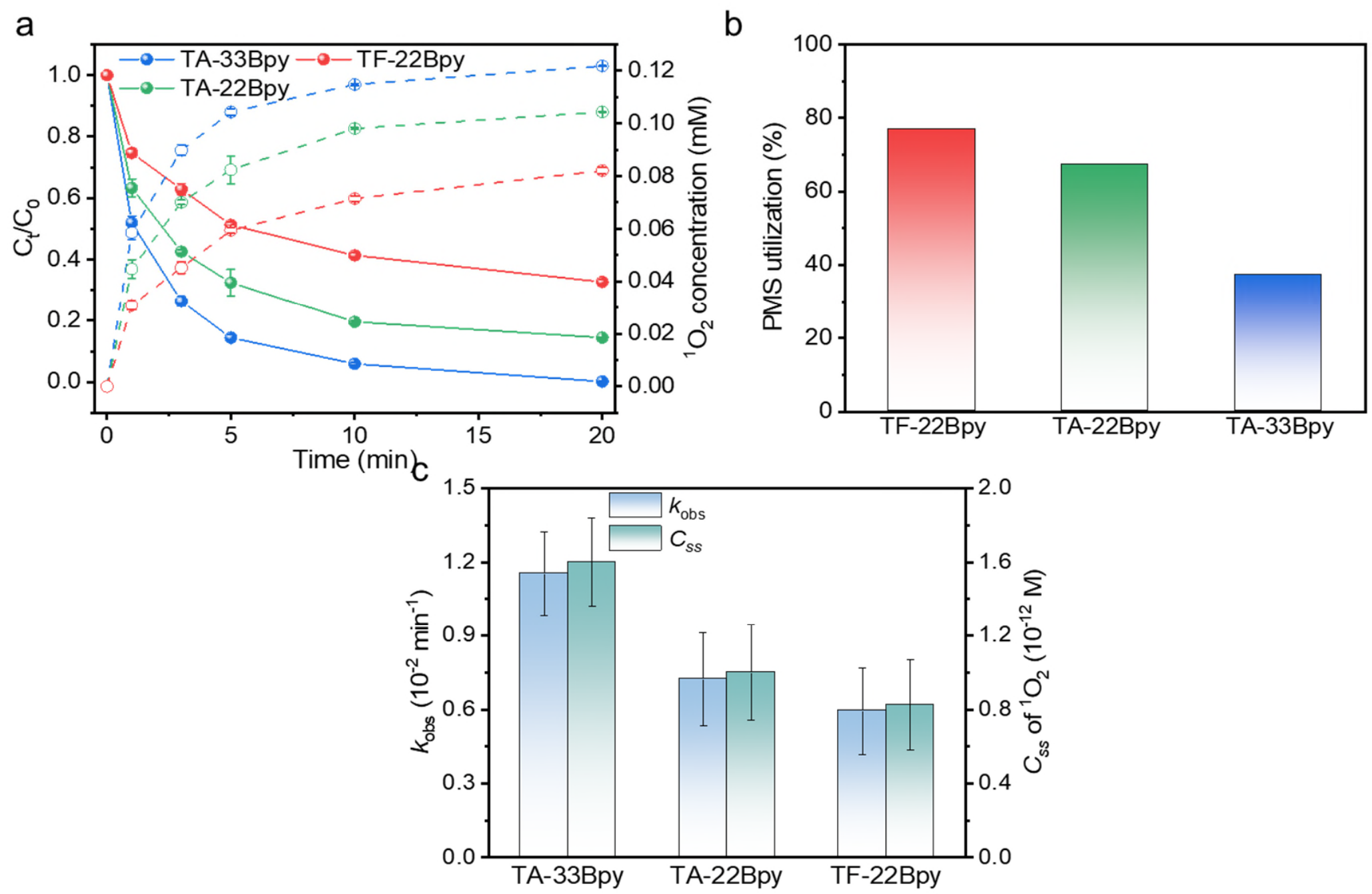

Fig. S21 (a) The consumption of ABDA and generation of $^{1}O_2$ in COFs/PMS system. (b) Efficiency of PMS to $^{1}O_2$. (c) The k_{obs} of FFA probes and steady-state concentration of $^{1}O_2$.

Supporting Information, Page 48,

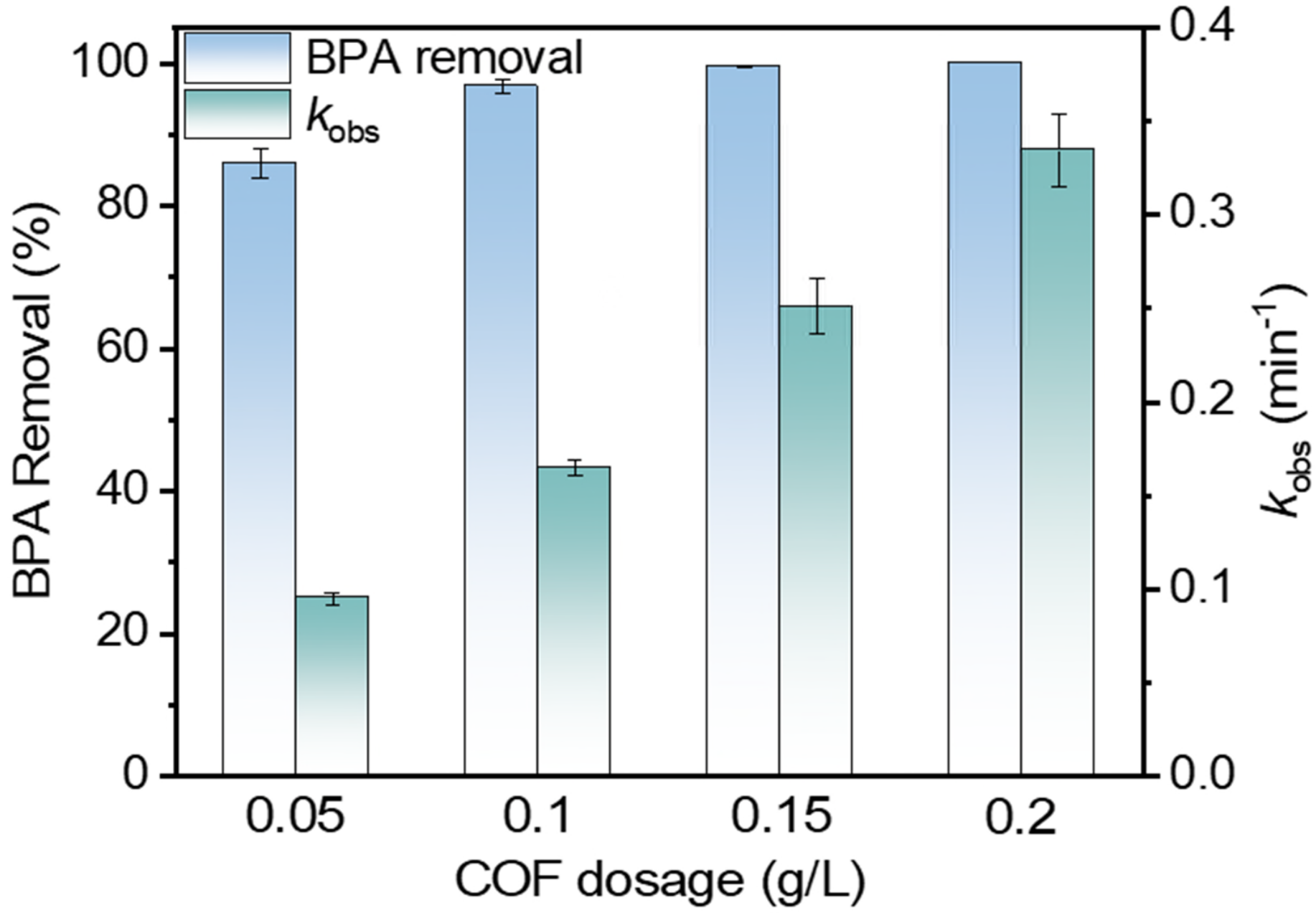

Fig. S42 The influences of COF loading on BPA removal in TA-33Bpy/PMS system.

Comment 7. In summary, while the idea of N-site isomerism as a handle to tune COF electronic structure is interesting, the central claims (exclusive $^1O_2$, PMS-triggered spatial charge separation, and practical robustness) are not supported at the level required for Nature Communications. The manuscript would require substantial additional mechanistic and stability work, clearer and more defensible structural validation, and broader environmental relevance. I therefore recommend rejection at this time for Nature Communications.

Response:

We sincerely thank Reviewer #1 for the thorough and critical assessment of our manuscript. We greatly appreciate the reviewer's recognition that "the idea of N-site isomerism as a handle to tune COF electronic structure is interesting." We have taken very seriously the concerns regarding the mechanistic claims, structural validation, and environmental relevance. In response, substantial revisions and additional analyses have been incorporated to strengthen the crystallographic validation of the COF structures, refine the mechanistic interpretation of PMS activation and $^1O_2$ generation, expand the evaluation of catalyst stability and structural evolution during operation, and broaden the assessment of application-relevant performance.

We believe that, with these substantial additions, the central claims are now supported by multiple lines of evidence. The revised manuscript has been carefully re-organized, with all new data incorporated and the claims tempered where appropriate. We respectfully hope that the improved version may be reconsidered for publication in *Nature Communications*

Reviewer #2

General Comments: The manuscript titled "Engineering N-site isomerism in donor-acceptor covalent organic frameworks for efficient Fenton-like water purification" describes the development of a engineering strategy to modulate the electronic structures of donor-acceptor (D-A) covalent organic frameworks (COFs). The authors systematically synthesized three D-A COFs (TF-22Bpy, TA-22Bpy, and TA-33Bpy) with varying positions of imine and pyridine nitrogen atoms. The performance of these COFs in activating peroxymonosulfate (PMS) for singlet oxygen ($^1O_2$) generation and BPA degradation was evaluated. The results showed that TA-33Bpy exhibited the highest catalytic activity, with a significant enhancement in BPA removal efficiency compared to the other two COFs. The study also included theoretical calculations and experimental analyses to elucidate the mechanism of PMS activation and $^1O_2$ generation. I think this work will be of interest to others in the field, and I recommend it for publication after the authors address the following:

Response: We thank Reviewer #2 for the positive evaluation and encouraging comments on our work. We have carefully addressed all the specific comments raised below and revised the manuscript accordingly. We believe that these revisions have further improved the quality and clarity of the manuscript.

Comment 1. The article employs various methods, including DFT calculations, experimental probes, and electrochemical techniques. However, some details in the methodology section could be further clarified. For instance, were all COF structures optimized in the DFT calculations? Was the solvent effect on the COF structure considered?

Response: We appreciate the valuable comments from Reviewer #2. In the DFT calculations, all COF structures were fully optimized in vacuum without considering solvent effects. Following the reviewer's suggestion, relevant details of DFT calculations, experimental probes, and electrochemical techniques have been added. Relevant revisions are as follows:

Supporting Information, Page 1,

"Singlet Oxygen Sensor Green (SOSG) were purchased from Shanghai Adamas Reagent Co., Ltd."

Supporting Information, Page 3,

"**Transient photocurrent response**

Indium tin oxide (ITO) glasses, platinum plate electrode and saturated calomel electrode (SCE) were used as working electrode, assistant electrode and reference electrode, respectively. For the preparation of working electrodes, 5 mg of catalyst was dispersed into a mixture of 0.45 mL of ethanol and 0.05 mL of Nafion® solution, followed by sonication for 3 h. The suspension (50 μL) was loaded onto a glassy carbon electrode and repeated dropwise two times for preparing a COFs-coated platinum plate electrode (COFs PPE). A 300 W xenon lamp was used as the light source, and the transient photocurrent with a bias of 0 V was tested over time during the on/off light cycle.

Galvanic oxidation process

For the preparation of working electrodes, 5 mg of catalyst was dispersed into a mixture of 0.45 mL of ethanol and 0.05 mL of Nafion® solution, followed by sonication for 3 h. The suspension (200 μL) was uniformly coated onto a graphite plate with dimensions of 20 mm × 20 mm × 3 mm. The plate was then dried in a 60 °C oven. The two graphite plates were separated and placed in two electrolyte chambers with 0.5 M Na_2SO_4 containing 20 ppm BPA and 0.1 g L^{-1} PMS respectively, and connected by agar salt bridges to maintain charge balance during the reaction process. The current changes during the reaction were monitored in real time using the CHI 760E electrochemical workstation."

Supporting Information, Page 4,

"As for recycling experiment, the used catalyst was washed with ethanol to remove the oligomeric products and then dried in a vacuum oven at 60 °C.

Continuous-flow performance

The catalyst (100 mg) was dispersed in a 20 mL flow column. A 10 mg/L BPA solution and a 1.3 M PMS solution were introduced into the reaction column from two different pipelines at a rate of 1.1 mL per second from bottom to top, and the hydraulic retention time was 9.1 min."

Supporting Information, Page 5,

"**SOSG probe measurement**

The generation of $^1O_2$ was detected by an SOSG probe measurement. Initially, SOSG (100 μg) was dissolved in 300 μL of MeOH to obtain a SOSG solution with a concentration of 500 μM, which was freshly prepared prior to each use. Catalysts (1 mg) and H_2O (10 mL) were added into a 25 mL glass beaker, followed by sonication for 10 min to ensure that the material was completely dispersed. Afterward, 150 μL of SOSG solution was added into the beaker; then, PMS (100 mM, 100 μL) was added to initiate the reaction. After 5, 10, 15, and 20 min, 1 mL of the sample was withdrawn and added to 1 mL MeOH. The obtained sample was immediately filtered with a PTFE syringe filter (0.22 μm). Finally, the sample was assessed by using a fluorescence spectrometer (Lengguang, F97). The excitation wavelength was set at 488 nm, and the emission wavelength range was 500−600 nm.

Electronic paramagnetic resonance (EPR) measurement

To characterize the charge polarization of COFs induced by PMS, we added 250 μL of PMS aqueous solution (0.4 g L^{-1}) to 250 μL of the COFs dispersion solution (0.2 g L^{-1}), followed by vigorously shaken for 5 seconds to ensure complete mixing. After adsorbing the reaction solution with a capillary, the EPR signal was detected by Bruker A300. Similarly, in order to detect TEMP-$^1O_2$ and DMPO-$SO_4^{\bullet-}$/$^{\bullet}OH$, the COF dispersion solutions were prepared containing 50 mM TEMP and 200 mM DMPO, respectively. As for DMPO-$O_2^{\bullet-}$, the COFs (0.2 g L^{-1}) were dispersed into a methanol solution containing 200 mM DMPO."

Supporting Information, Page 6,

"**DFT Calculations**

All COFs structures were fully optimized in vacuum without considering solvent effects. The calculations were performed within the framework of density functional theory (DFT) using the projector augmented-wave (PAW) method, as implemented in the Vienna *ab initio* simulation package (VASP). The Perdew-Burke-Ernzerhof (PBE) functional within the generalized gradient

approximation (GGA) was employed to describe the exchange-correlation potential, and long-range van der Waals interactions were included using the DFT-D3 method."

Comment 2. The article mentions that BPA oligomers may undergo polymerization on the catalyst surface, but the specific impact of this phenomenon on catalytic performance is not further discussed. I suggest adding a more detailed analysis of this polymerization process in the discussion section, particularly how it affects the catalyst's stability, active sites, and long-term catalytic efficiency.

Response: We appreciate the insightful comments from Reviewer #2. Following the reviewer's suggestion, we have now added the relevant discussion to the revised manuscript. Relevant revisions are as follows:

Page 22, line 525

"The identified intermediates suggest that BPA undergoes sequential $^1O_2$-driven oxidation and ring-cleavage reactions, ultimately yielding small organic acids and, eventually, CO_2 and H_2O. Meanwhile, analysis of the methanol-eluted fraction from the spent catalyst surface revealed BPA oligomers, indicating that part of BPA follows a parallel surface-confined oligomerization/polymerization pathway. This interpretation is consistent with recent studies showing that selective nonradical oxidation in persulfate-based AOPs, including $^1O_2$-mediated pathways, can convert phenolic substrates into phenoxy-radical-type intermediates that further couple on catalyst surfaces[63]. The accumulation of these surface-bound products may facilitate initial pollutant removal, but persistent deposition could progressively block active sites and impede interfacial mass/electron transfer, thereby lowering catalyst stability and long-term catalytic efficiency. Furthermore, the ecological toxicity of the detected aqueous intermediates was evaluated using ECOSAR software (version 2.2) based on quantitative structure-activity relationship (QSAR) models."

Page 30, line 791,

"63 Zhang, P., Yang, Y., Duan, X. & Wang, S. Oxidative polymerization versus degradation of organic pollutants in heterogeneous catalytic persulfate chemistry. *Water Res.* **255**, 121485 (2024)."

Comment 3. Although the article discusses the stability of TA-33Bpy and possible issues such as pore blocking, could other countermeasures or suggestions for improvement be provided to maintain the catalyst's efficiency over extended use?

Response: We appreciate Reviewer #2's constructive comments. In the revised manuscript, we have added a discussion on potential strategies to sustain the catalytic efficiency of TA-33Bpy during prolonged operation. The corresponding revisions are presented as follows:

Page 23, line 545,

"N_2 adsorption-desorption results showed that the specific surface area, pore volume and pore size decreased from 2223, 1.29 and 2.48 to 777 m^2/g, 0.50 cm^3/g and 2.38 nm, respectively (Fig. S53)."

Page 23, line 556,

"Furthermore, recycling experiments were performed, and the catalysts recovered after each cycle were systematically characterized to track their structural evolution during operation. As shown in Fig. S56, the reaction rate gradually decreased with increasing cycle number, accompanied by slight reductions in specific surface area and pore volume, whereas the pore size remained essentially unchanged (Fig. S57). In addition, the PXRD patterns showed negligible changes in crystallinity throughout the cycling process (Fig. S58). These results suggest that the decrease in surface area and partial pore blockage mainly originated from the accumulation of BPA-derived polymeric products during continuous-flow operation, which could be largely removed by methanol washing. To minimize the influence of surface-area variation, the observed rate constants (k_{obs}) were further normalized by the specific surface area for each cycle (Table S9). The area-normalized kinetic constant began to decline noticeably after the fourth cycle, indicating a gradual decrease in the intrinsic catalytic activity. To further probe the chemical evolution of the framework, we conducted XPS analyses on the catalysts collected after different cycles. As shown in Fig. S59, the amide-related signal progressively increased, while the imine-related signal gradually weakened with increasing cycle number. Notably, the imine species nearly disappeared after the fourth cycle, consistent with the decline in the area-normalized activity, suggesting that the imine linkage plays a critical role in PMS activation (Table S10). The gradual loss of catalytic activity was mainly attributed to the limited stability of imine linkages under prolonged oxidative conditions. Accordingly, periodic regeneration through organic-solvent washing followed by mild drying, together with post-synthetic conversion of imine linkages into more robust heterocyclic linkages while preserving their protonatable character

for PMS interaction, may provide effective strategies for improving the long-term durability of TA-33Bpy."

Supporting Information, Page 62-65,

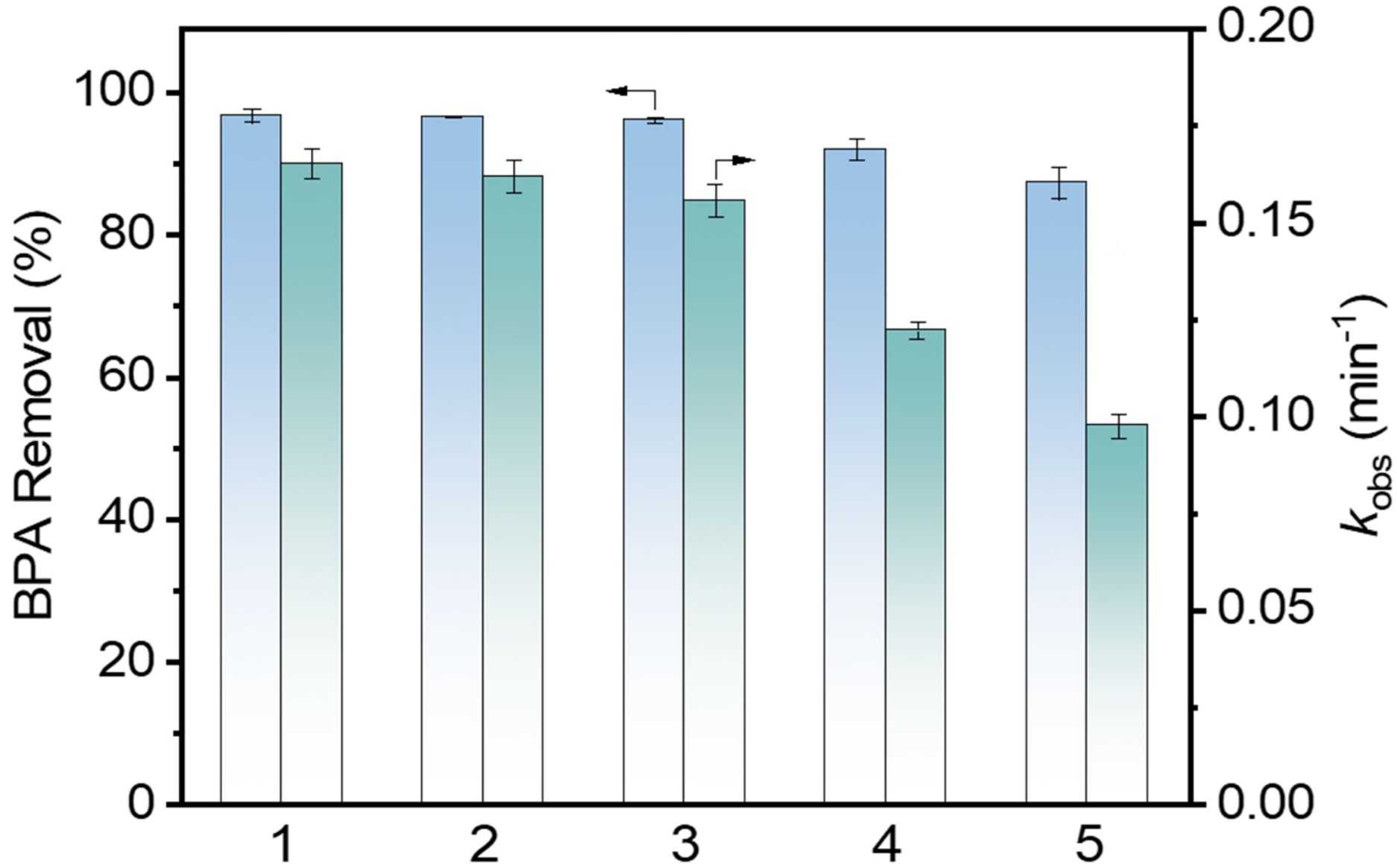

Fig. S56 Recycling of TA-33Bpy.

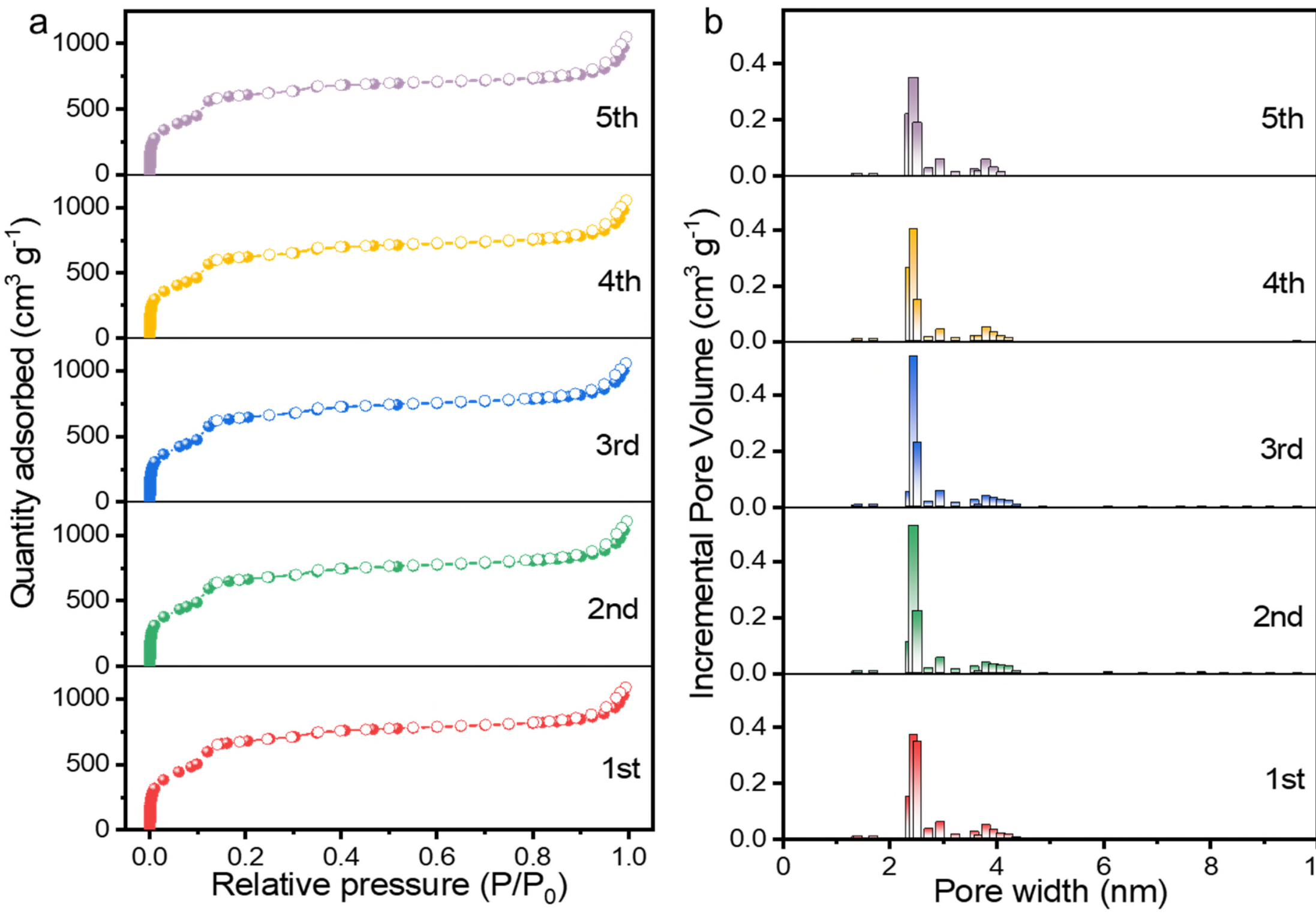

Fig. S57 (a) N_2 adsorption-desorption isotherms, and (f) pore size distributions of the recycled TA-33Bpy.

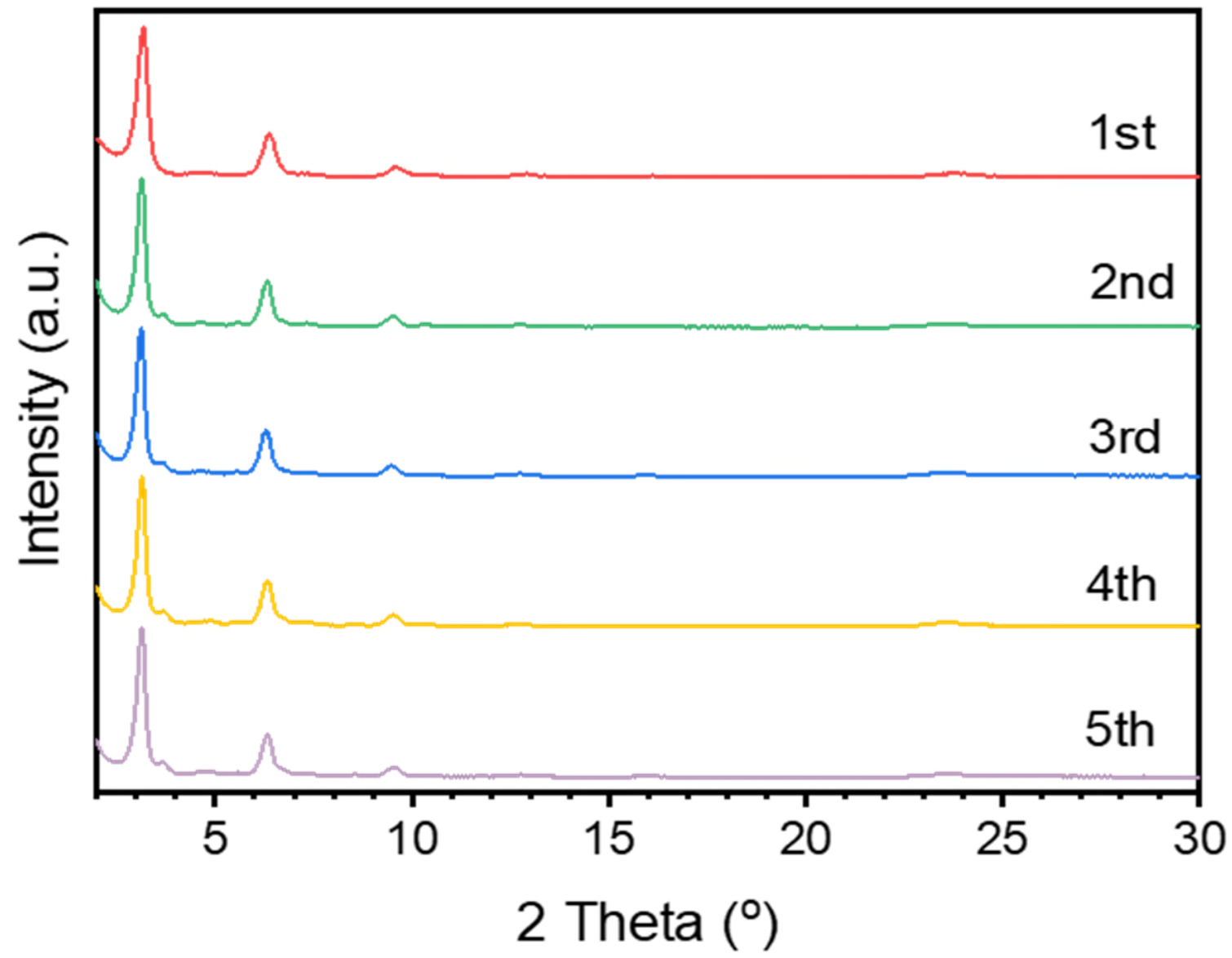

Fig. S58 XRD patterns of the recycled TA-33Bpy.

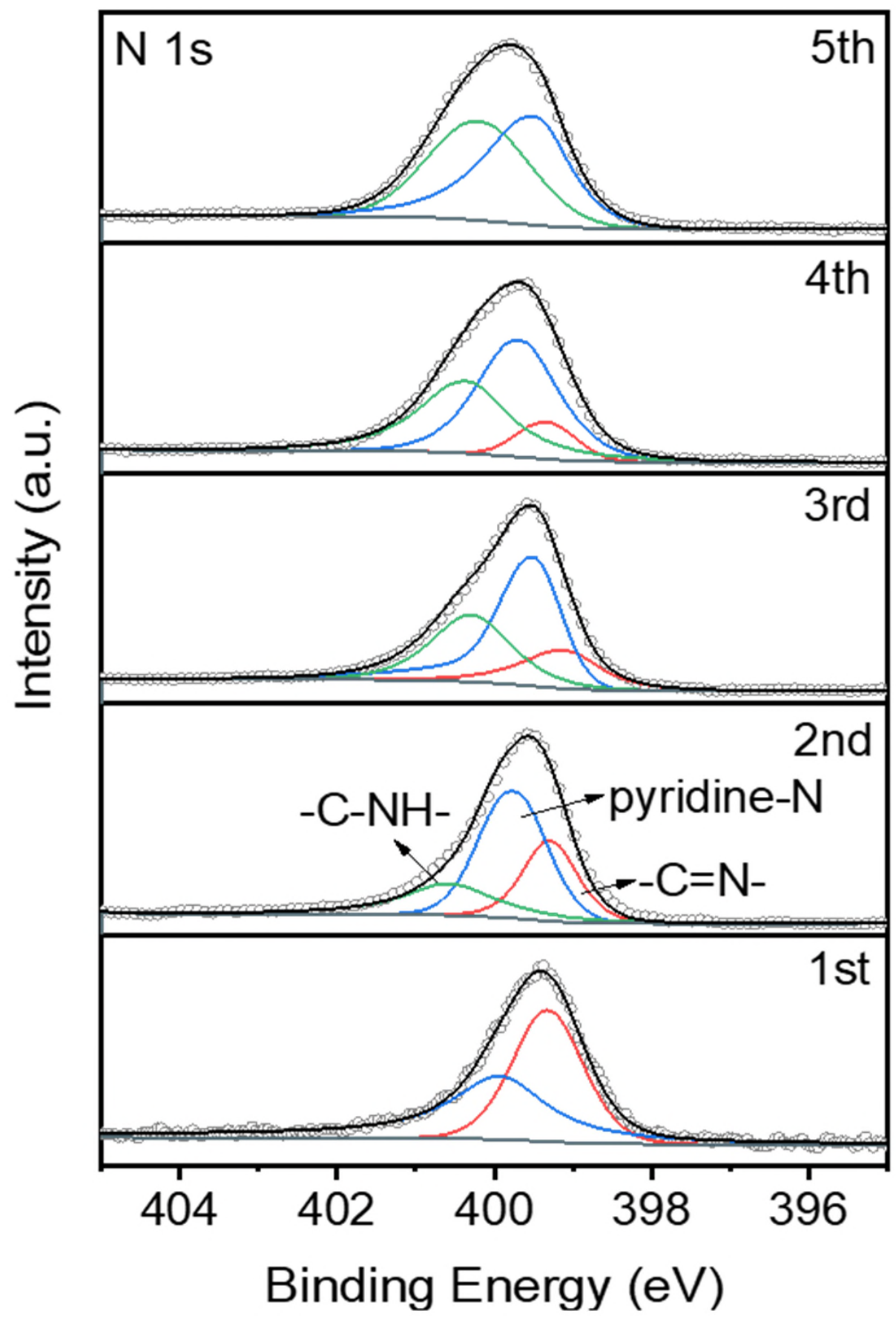

Fig. S59 N 1s spectra of the recycled TA-33Bpy.

Comment 4. The N-site isomeric engineering strategy proposed in this study shows significant effects in D-A COFs. Could this strategy be extended to other types of COFs or catalytic reactions? Exploring this could further enhance the broader applicability of the research.

Response: We appreciate the valuable comments from Reviewer #2. As the reviewers noted, isomeric engineering has indeed emerged as an effective strategy for regulating the structure and properties of COFs, including topological isomerism, constitutional isomerism, and stereoisomerism. However, its application in PMS-AOPs remains largely unexplored. To the best of our knowledge, the present work represents the first attempt to introduce N-site isomeric engineering into COF-based PMS-AOPs, thereby establishing a direct connection between N-site arrangement, COF-PMS interfacial electronic interaction, and selective ROS generation. In the revised manuscript, we have further discussed the broader significance and potential extensibility of this strategy. The corresponding revisions are presented as follows:

Page 25, line 590,

"This work highlights the decisive role of PMS-triggered charge polarization in mediating PMS activation and establishes, for the first time, N-site isomeric engineering as an isomerism-guided design strategy for COF-based PMS activation, with potential applicability to broader nitrogen-containing COFs and catalytic reactions."

Comment 5. Please ensure proper chemical formula formatting, maintaining consistency and accuracy throughout.

Response: We appreciate the helpful comment from Reviewer #2. Following the Reviewer's suggestion, the manuscript has been double checked and revised to ensure consistent and accurate formatting of chemical formulas throughout the text, figures, captions, and Supporting Information. Relative revisions are added as follows:

Page 5, line 114,

"Similarly, TA-33Bpy shows a pyridine-like N environment stabilized by an intramolecular [$N{-}H^{+}{\cdots}N$] hydrogen bond between the protonated pyridine-N and the neighboring imine-N"

Page 9, line 186,

"High-resolution N 1s spectra could be deconvoluted into two components corresponding to imine-N and pyridine-N."

Page 9, line 192,

"N-site isomerism"

Page 10, line 213,

"(e) N_2 adsorption-desorption isotherms"

Page 10, line 217,

"To elucidate the influences of N-site isomerism on the electronic and band structures of the COFs"

Page 21, line 500,

"Such deprotonation-induced surface charge variation likely enhanced the electrostatic repulsion between TA-33Bpy and anionic HSO_5^- species, thereby reducing the interfacial PMS activation efficiency."

Page 23, line 545,

"N_2 adsorption-desorption results showed that the specific surface area, pore volume and pore size decreased from 2223, 1.29 and 2.48 to 777 m^2/g, 0.50 cm^3/g and 2.38 nm, respectively"

Page 24, line 581,

"In summary, we have developed a N-site isomeric strategy to fine-tune the electronic structures of D-A COFs"

Reviewer #3

General Comments: In this manuscript, the authors presented an investigation on the rational design of donor-acceptor covalent organic frameworks (D-A COFs) via N-site isomeric engineering to achieve highly efficient and selective generation of singlet oxygen ($^1O_2$) for peroxymonosulfate (PMS)-based water purification. A novel mechanism of $^1O_2$ generation via PMS activation was proposed for the first time. PMS-induced spatially separated charges (holes and electrons) in D-A COFs can sequentially achieve the oxidation of HSO_5^- and the reduction of $SO_5^{\bullet-}$ for $^1O_2$ generation. The central findings were well-supported by a convincing combination of detailed experiments and theoretical calculations. In general, the investigation establishes a clear structure-property-activity relationship and offers a valuable design principle for COFs-based catalysts. However, some conclusions are needed for more evidence and some details still need to be improved. Therefore, I suggest its publication in Nature Communications after all these problems are well addressed. The comments are listed below:

Response: We thank Reviewer #3 very much for encouragement and kind comments. Following the suggestions, we have carefully revised the manuscript and addressed all the specific comments point by point. Additional evidence, clarifications, and textual revisions have been incorporated where appropriate to further strengthen the conclusions and improve the accuracy and clarity of the manuscript. Below we respond to each comment in detail.

Comment 1. Line 91, the authors mentioned that "a PMS-triggered self-catalytic mechanism…" is confusing. While the concept of spatially separated redox sites driving the cycle is clear, the term "self-catalytic" could be misinterpreted. It might imply autocatalysis or regeneration without external reagents.

Response: We appreciate the Reviewer for helpful comments. Following the suggestion, the term "self-catalytic" has been revised. Relevant revision is added as follows:

Page 5, line 92,

"More importantly, we elucidate for the first time a PMS-triggered charge polarization mechanism, in which direct PMS–COF interaction induces electronic redistribution across the donor (D^+) and acceptor (A^-) units, thereby cooperatively promoting PMS oxidation and $SO_5^{\bullet-}$ reduction toward predominant $^1O_2$ generation."

Comment 2. How are the structures of adjacent pyridine-N in TF-22Bpy and TA-22Bpy as well as pyridine-like structure in TA-33Bpy formed during the preparation of COFs?

Response: We appreciate the valuable comments from Reviewer #3. Following the reviewer's suggestion, the formation mechanism of adjacent pyridine-N in TF-22Bpy and TA-22Bpy as well as pyridine-like structure in TA-33Bpy has been discussed. Relevant revisions are added as follows:

Page 5, line 111,

"The adjacent pyridine-N motif in TF-22Bpy and TA-22Bpy is derived from the 2,2'-bipyridyl unit, whose protonated form preferentially adopts a cis conformation under acidic solvothermal conditions because of the intramolecular $[N{-}H^{+}\cdots N]$ hydrogen bond between the two pyridine N atoms. Similarly, TA-33Bpy shows a pyridine-like N environment stabilized by an intramolecular $[N{-}H^{+}\cdots N]$ hydrogen bond between the protonated pyridine N and the neighboring imine N[48]."

Page 29, line 746,

"48 Mi, Z. et al. Stable Radical Cation-Containing Covalent Organic Frameworks Exhibiting Remarkable Structure-Enhanced Photothermal Conversion. J. Am. Chem. Soc. 141, 14433-14442 (2019)."

Comment 3. Due to the presence of holes and electrons, the effect of dissolved oxygen in the system needs to be taken into account.

Response: We appreciate the insightful comments from Reviewer #3. Following the reviewer's suggestion, the effect of dissolved oxygen has been investigated. Relevant revisions are added as follows:

Page 17, line 399,

"Control experiments under N_2 bubbling showed nearly unchanged catalytic activity, suggesting that dissolved oxygen (DO) makes a negligible contribution to $^1O_2$ generation."

Page 20,

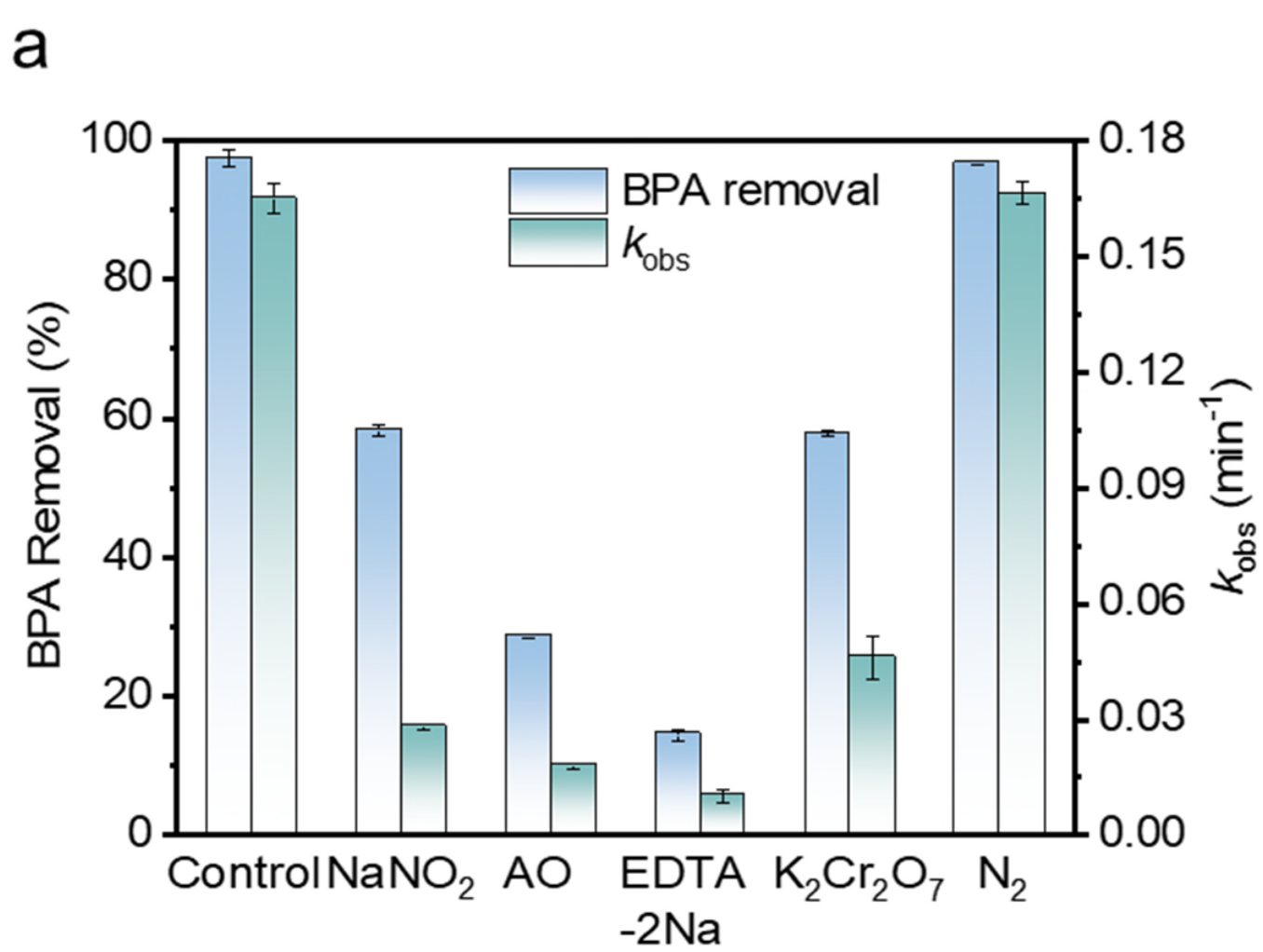

"

Fig. 4 Origin of $^1O_2$ generation. (a) Quenching experiments of electron-deficient sites, electron-enriched sites, $SO_5^{\bullet-}$ and DO in TA-33Bpy/PMS system."

Comment 4. What about the consumption of PMS during the process?

Response: We appreciate the helpful comment from Reviewer #3. Following the reviewer's suggestion, the consumption of PMS during the process has been quantified. Relevant revisions are added as follows:

Page 13, line 297,

"To quantitatively verify $^1O_2$ generation in the heterogeneous PMS system, we further conducted PMS consumption, ABDA-based $^1O_2$ quantification, and FFA kinetic measurements. Within 20 min, the PMS consumption reached 50.2%, 23.8%, and 16.4% for TA-33Bpy, TA-22Bpy, and TF-22Bpy, respectively (Fig. S20)."

Supporting Information, Page 26,

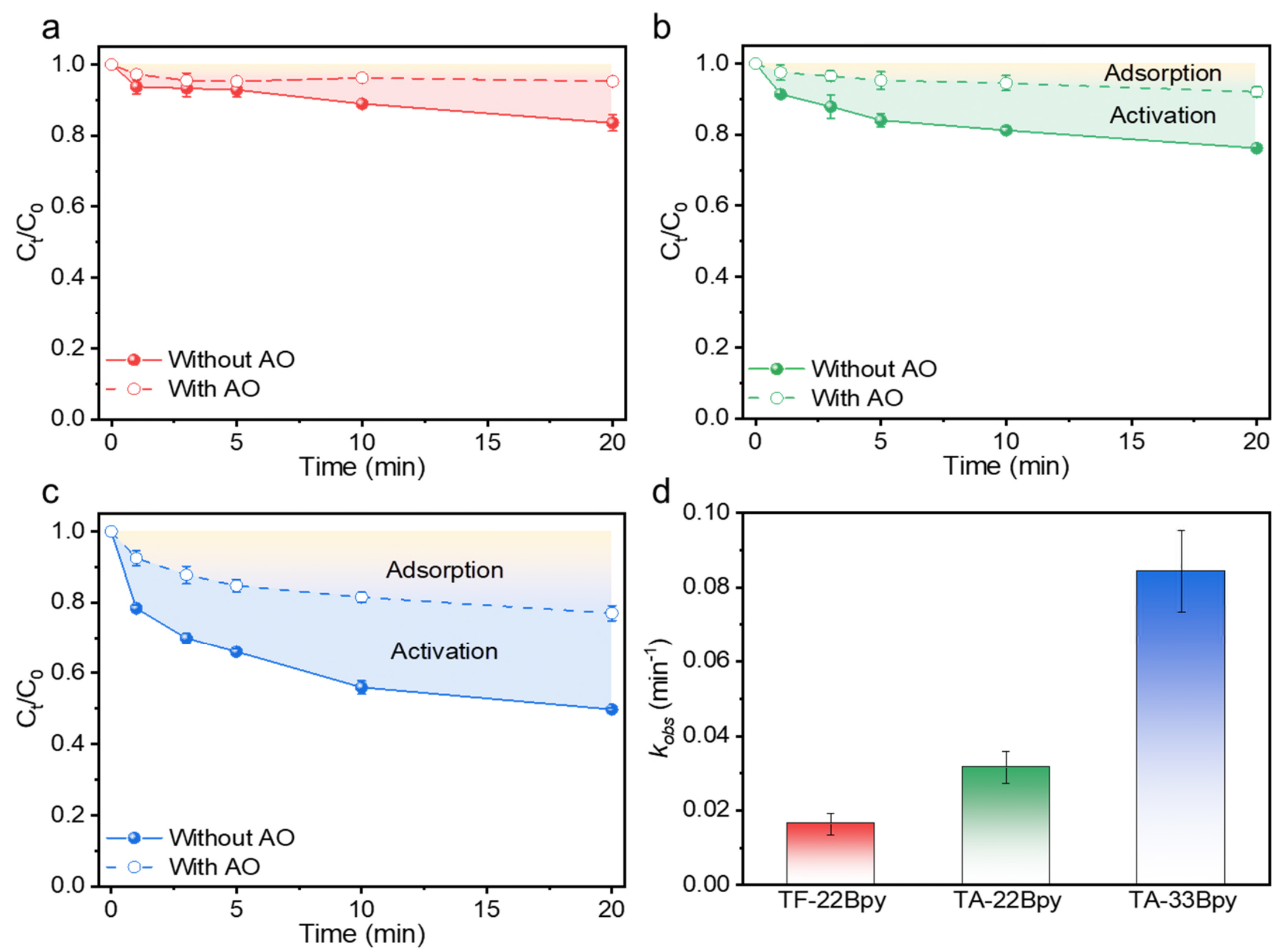

Fig. S20 The consumption and adsorption of PMS by (a) TF-22Bpy, (b) TA-22Bpy and (c) TA-33Bpy. (d) PMS consumption kinetics.

Comment 5. The details of EPR and GOP tests should be provided.

Response: We appreciate the helpful comment from Reviewer #3. Following the reviewer's suggestion, the details of EPR and GOP tests has been added. Relevant revisions are added as follows:

Supporting Information, Page 3,

"Galvanic oxidation process

For the preparation of working electrodes, 5 mg of catalyst was dispersed into a mixture of 0.45 mL of ethanol and 0.05 mL of Nafion® solution, followed by sonication for 3 h. The suspension (200 μL) was uniformly coated onto a graphite plate with dimensions of 20 mm × 20 mm × 3 mm. The plate was then dried in a 60 °C oven. The two graphite plates were separated and placed in two electrolyte chambers with 0.5 M Na_2SO_4 containing 20 ppm BPA and 0.1 g L^{-1} PMS, respectively, and connected by agar salt bridges to maintain charge balance during the reaction process. The current changes during the reaction were monitored in real time using the CHI 760E electrochemical workstation."

Supporting Information, Page 5,

"Electronic paramagnetic resonance (EPR) measurement

To characterize the charge polarization of COFs induced by PMS, we added 250 μL of PMS aqueous solution (0.4 g L^{-1}) to 250 μL of the COFs dispersion solution (0.2 g L^{-1}), followed by vigorously shaken for 5 seconds to ensure complete mixing. After adsorbing the reaction solution with a capillary, the EPR signal was detected by Bruker A300. Similarly, in order to detect TEMP-$^1O_2$ and DMPO-$SO_4^{\bullet-}$/$^{\bullet}OH$, the COF dispersion solutions were prepared containing 50 mM TEMP and 200 mM DMPO, respectively. As for DMPO-$O_2^{\bullet-}$, the COFs (0.2 g L^{-1}) were dispersed into a methanol solution containing 200 mM DMPO."

Comment 6. Some of the figures in the manuscript are blurry, such as Fig. 2a-c and insert of Fig.3a.

Response: We appreciate the helpful comment from Reviewer #3. Following the reviewer's suggestion, figures with higher definition have been provided. Relevant revisions are added as follows:

Page 10,

“

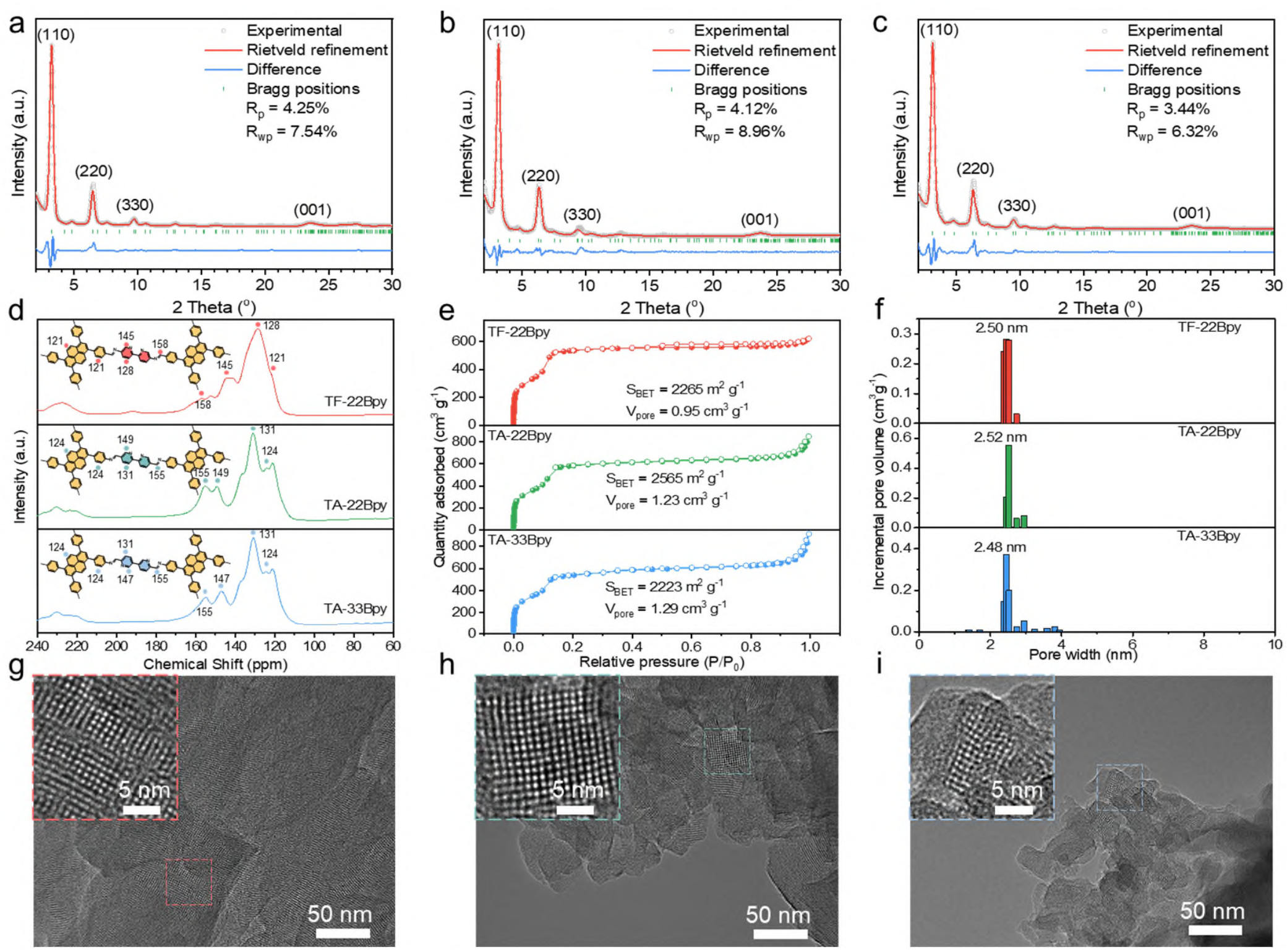

Fig. 2 Characterizations of isomeric COFs. Experimentally obtained PXRD patterns (grey circle), Pawley refined patterns (red curves), their differences (blue curves) and Bragg positions (green verticals) of (a) TF-22Bpy, (b) TA-22Bpy, and (c) TA-33Bpy COF. (Insets: Top and side views of COFs with AA-stacking). (d) Solid-state ^{13}C CP-MAS NMR spectra, (e) N_2 adsorption-desorption isotherms, and (f) pore size distributions of isomeric COFs. TEM and HRTEM images of (g) TF-22Bpy, (h) TA-22Bpy, and (i) TA-33Bpy COFs.”

Page 15,

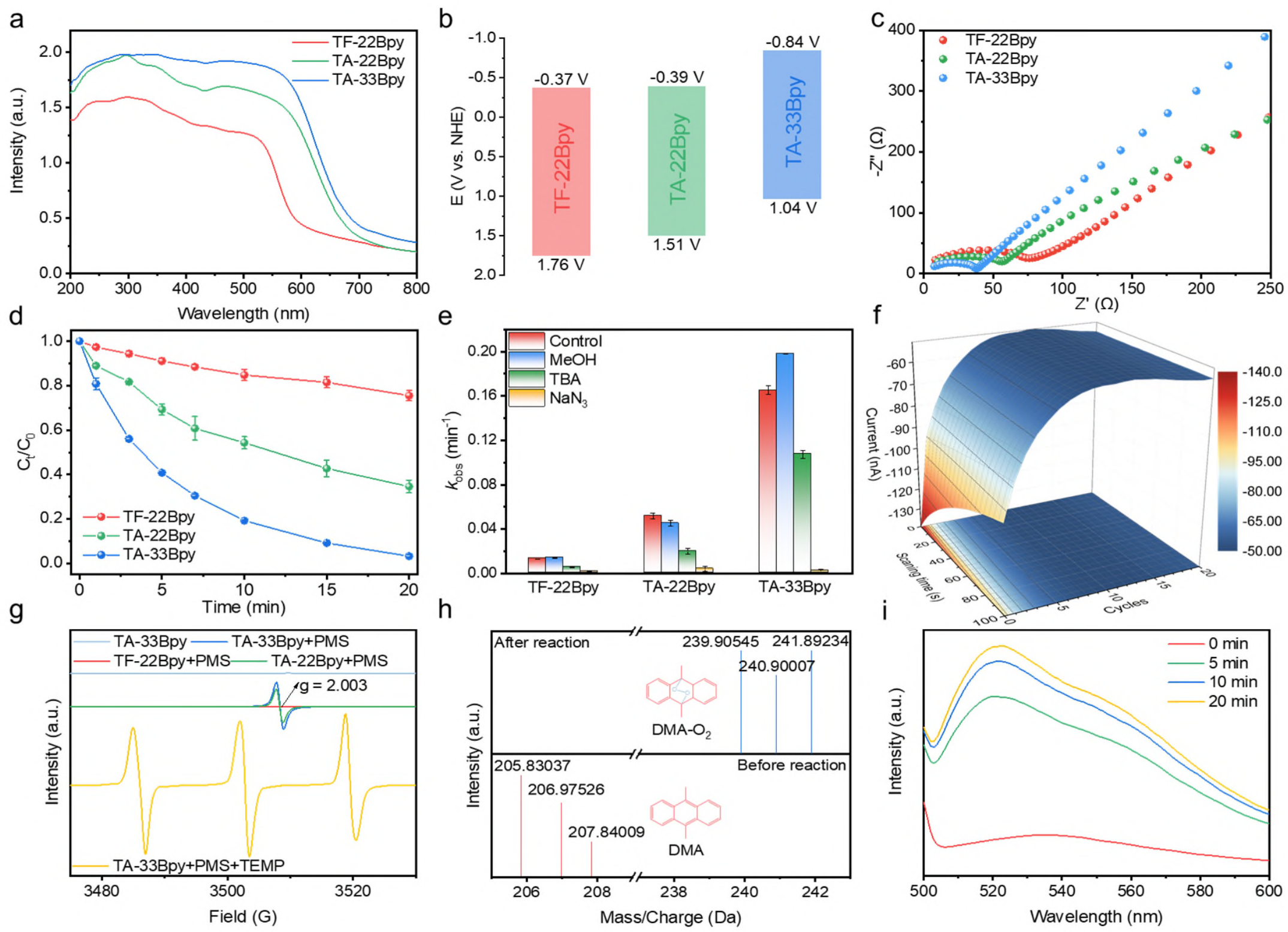

"

Fig. 3 Physicochemical properties and Fenton-like performance. (a) UV–vis–NIR diffuse reflectance spectra, (b) electronic band structures, and (c) electrochemical impedance spectra of the isomeric COFs. (d) BPA removal and (e) quenching experiments of all COFs/PMS system. (f) Scanning electrochemical microscopic analysis of TA-33Bpy/PMS systems. (g) EPR spectra of TA-33Bpy/PMS system using TEMP as trapping agents. (h) HR-MS chromatogram of typical DMA-O_2 from DMA oxidation in TA-33Bpy/PMS system. (i) Fluorescence spectra of SOSG-EP in TA-33Bpy/PMS system."

Comment 7. There are some spelling and grammar mistakes in the text. For example, page 6, line 128, "separation and directional electron transfer between[42,48]" should be revised as "separation and directional electron transfer[42,48]". The authors should check this manuscript carefully.

Response: We appreciate the helpful comments from Reviewer #3. Following the reviewer's suggestion, we have thoroughly rechecked the entire manuscript and corrected the spelling and grammar errors. The manuscript has been carefully revised throughout to improve the accuracy and clarity of the language. Relevant revisions are added as follows:

Page 6, line 132,

"This clear spatial separation of frontier orbitals is the characteristic of a donor-acceptor architecture and is intrinsically favorable for intermolecular charge polarization and electron transfer"

Page 8, line 173,

"As shown in Fig. S5, the emergence of characteristic C=N bond stretching vibration at 1624 (TF-22Bpy), 1627 (TA-22Bpy), and 1608 cm^{-1} (TA-33Bpy) was accompanied by the disappearance of the primary amine ($-NH_2$) and aldehyde (-CHO) signals of the corresponding monomers"

Page 9, line 201,

"SEM images showed that TF-22Bpy adopts an irregular stick-like morphology"
